# GEJ cancers: gastric or esophageal tumors? searching for the answer according to molecular identity

**DOI:** 10.18632/oncotarget.22216

**Published:** 2017-10-31

**Authors:** Williams Fernandes Barra, Fabiano Cordeiro Moreira, Aline Maria Pereira Cruz, André Salim Khayat, Danielle Queiroz Calcagno, Ney Pereira Carneiro dos Santos, Rui Wanderley Mascarenhas Junior, Taíssa Maíra Thomaz Araújo, Geraldo Ishak, Samia Demachki, Rommel Mario Rodríguez Burbano, Ândrea Kely Campos Ribeiro dos Santos, Sidney Emanuel Batista dos Santos, Gregory Joseph Riggins, Paulo Pimentel de Assumpção

**Affiliations:** ^1^ Núcleo de Pesquisas em Oncologia, Universidade Federal do Pará, Brazil; ^2^ Hospital Universitário João de Barros Barreto, Universidade Federal do Pará, Brazil; ^3^ Brain Cancer Biology and Therapy Research Laboratory, Johns Hopkins Medicine, Baltimore, MD, USA

**Keywords:** gastroesophageal junction, 7^th^ UICC, TCGA, molecular fingerprints, expression pattern

## Abstract

The 7th edition of Union for International Cancer Control (UICC) staging system moved gastroesophageal junction (GEJ) cancers from gastric to esophageal group. Since clinical management is strongly influenced by this staging system, we looked at molecular fingerprints of GEJ tumors and compared to gastric and esophageal profiles. We aimed at elucidating whether GEJ cancers cluster with gastric or esophageal groups according to mRNA and microRNA expression pattern, since this might represent tumor identity. The clinical and expression data were downloaded from The Cancer Genome Atlas (TCGA) with 395 stomach, 184 esophagus and 521 colon samples for mRNA analyses and 392 stomach, 175 esophagus and 459 colon samples for microRNA comparisons. Both Principal Component Analysis (PCA) and Heat Map plots were performed in R platform, using Log_2_ transformation of RPKM normalized data. Differential Expression Analysis was also performed in R, using RAW data and the DESeq2 package. The mRNAs and microRNAs were tagged as differentially expressed if they met the following criteria: i) FDR adjusted p-value < 0.05; and ii) |Log_2_ (fold-change)| > 2. Esophagus squamous cell carcinoma (ESCC) clustered apart of the others tumors, while adenocarcinomas (AC) clustered all together according to both mRNAs and microRNAs expression patterns. The HMs of the differentially expressed mRNAs and microRNAs also demonstrated that ESCC belongs to a different group, while AC molecular signature of esophagus looks like AC of the cardia and non cardia regions. Even distal gastric cancers are quite similar to AC of the lower esophagus, demonstrating that esophagus AC relies much closer to gastric cancers than to esophagus cancers. By using robust molecular fingerprints, it was strongly demonstrated that GEJ tumors looks more like gastric cancers than esophageal cancers, despite of tumor heterogeneity.

## INTRODUCTION

The 7th edition of the Union for International Cancer Control–American Joint Committee on Cancer (UICC-AJCC) tumor, node and metastasis (TNM) staging system moved gastroesophageal junction (GEJ) cancers from the gastric staging group to esophageal cancers [1]. This change was based on a review of esophageal cancer staging systems that evaluated a data set of 4,627 patients who were treated by surgical resection without previous or adjuvant therapy; the data set was assembled by the Worldwide Esophageal Cancer Collaboration and analyzed using Random Forest Analysis [2].

Although the application of the 7th UICC-AJCC staging system seems to result in a better prognostic stratification of overall survival compared with the 6th edition for esophageal cancer, according to some reports [3, 4], others have demonstrated that the 7th UICC-AJCC staging system does not demonstrate advantages in the assessment of patient prognosis [5]. The current 8^th^ UICC-AJCC classification kept esophagus and esophagogastric tumors as a same tumor group [6].

According to the current UICC classification, GEJ cancers should more closely resemble esophageal tumors than gastric tumors, especially regarding their clinical and epidemiologic features. In addition, the staging system should favor the best decision regarding medical procedures by applying the rules for esophageal tumor management instead of those for gastric cancers [4]. Furthermore, this modification enhances the burden of esophageal cancer and do not increase the incidence of gastric cancer, thus providing a false perception of better gastric cancer control [7].

Nevertheless, clinical management algorithms and protocols are strongly influenced by the UICC-AJCC staging system, so the current classification has a tremendous impact on medical care. The number of retried lymph nodes necessary for adequate staging differs between gastric and esophageal tumors, and the specific lymph node stations that should be resected vary among these tumors. Thus, if a GEJ tumor is classified as a gastric cancer (by the older classification system), at least 16 lymph nodes are necessary to provide an adequate classification, whereas 12 lymph nodes are sufficient for esophageal cancers, according to the current UICC staging. Moreover, the proportion of positive to negative lymph nodes plays a role as a prognostic factor in gastric cancer. Given that fewer lymph nodes are necessary for staging, a negative impact for this benefit might occur [8].

An intensive debate is required to better define GEJ cancer as a specific entity or both a gastric and esophageal cancer or to keep these tumors classified as gastric or esophageal tumors [8].

The identification of the molecular characteristics of each tumor type can favor its classification and determine molecular targets [9–12].

The mRNAs expression pattern provides the specific tumor’s “molecular fingerprints”, and this information has been thoroughly investigated and reported for the majority of human cancers [13–15]. Moreover, the mRNA expression patterns can also differentiate subtypes or peculiar characteristics of a given tumor, such as aggressiveness or prognosis [15, 16]. It is necessary to consider that only transcribed mRNA will code for the proteins and enzymes that are typically present in tumors [17]. In some cases, these proteins and enzymes are used as tumor markers or even targets for therapy [18, 19]. Thus, it seems reasonable to use the mRNA profile to classify GEJ tumors as either gastric or esophageal cancers.

microRNAs are small non coding RNAs that function as post transcriptional regulators of gene expression. Their main function is to block translation by silencing mRNAs and impeding protein synthesis [20]. Due to been differentially expressed among tissues, microRNAs profiles can provide tissue signatures as previously demonstrated [21–24]. It means that looking at the microRNA is a feasible strategy to identify the origin of a given tissue, as a fingerprint.

There are plenty of reports implicating microRNA expression in human cancers [25–29]. Besides been able to characterize a given cancer, microRNAs profiles also work as markers of specific tumor behaviors as metastasis potential and prognosis [30–33]. As an advantage regarding, microRNAs are more stable and easily recovered from tissues specimens, including fixed samples [34–35]. Due to the specificity of expression in normal and cancer tissues, microRNA emerge as a powerful tool for characterizing GEJ tumors as either gastric or esophagus cancers.

The aims of this study are to enhance decision making regarding the best classification of GEJ cancers among the existing UICC categories. Thus, we provide the molecular fingerprints of these tumors and compared this information with gastric and esophageal molecular profiles to elucidate whether GEJs cluster in the gastric cancer or esophageal cancer groups based on mRNA and microRNA expression patterns because these information might represent a coherent tool for defining tumor identity.

## RESULTS

After dividing gastric cancer samples between cardia (SCA) and non-cardia (SNCA) tumors and esophageal cancers between adenocarcinomas (EA) and squamous cell carcinomas (ES), the number of tumors was defined as shown in Table 1.

**Table 1:** Sample data obtained from TCGA, discriminated by cancer type

Cancer type	mRNA samples	microRNA samples
Stomach non-cardia adenocarcinoma	302	299
Cardia adenocarcinoma	93	93
Esophageal adenocarcinoma	89	87
Esophageal squamous cell carcinoma	95	88
Colon adenocarcinoma	521	459
Total	1100	1026

**Figure 1:**
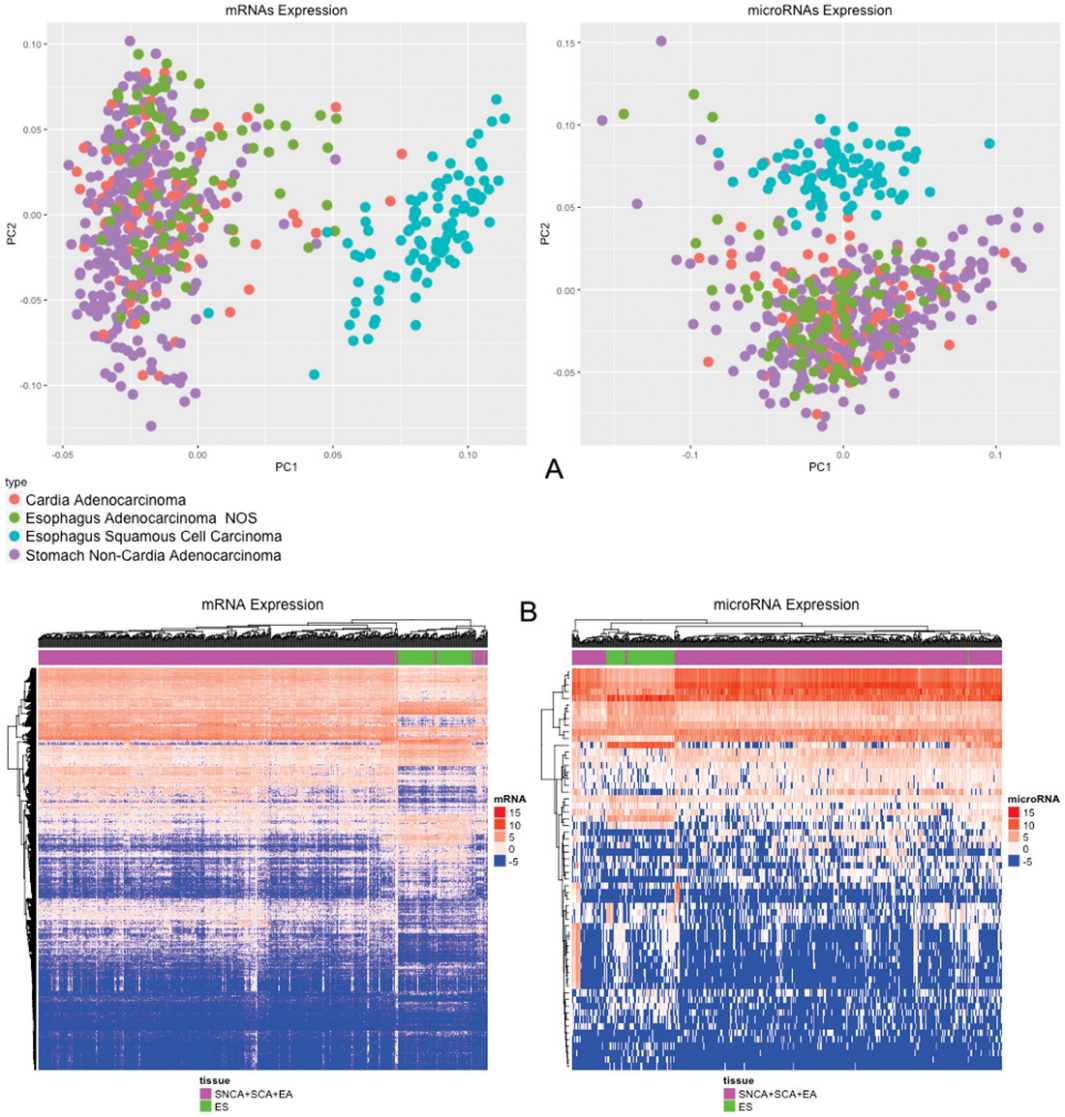
**(A)** PCA of 20,349 mRNAs and 910 microRNAs; **(B)** Heat Map of 2,366 mRNAs and 61 microRNAs differentially expressed (p-value < 0.05; |Log_2_(fold-change)| > 2) comparing non-cardia gastric adenocarcinomas, cardia adenocarcinomas and esophageal adenocarcinomas with esophageal squamous cell carcinomas.

Differential expression (D.E.) analysis was performed by grouping the samples as follows: i) (SNCA+SCA+EA) vs. ES; ii) EA vs. ES; iii) SNCA vs. EA; iv) (SNCA + SCA) vs. EA; and v) (SCA+EA) vs. SNCA. The number of differentially expressed mRNAs was 2366, 2550, 512, 375 and 186 for each analysis respectively. The number of differentially expressed microRNAs was 61, 60, 7, 5 and 2 for each analysis respectively (Table 2; Supplementary Figure 1; Supplementary Table 1). Esophageal squamous cell carcinoma clustered apart of the other tumors, whereas adenocarcinomas clustered together, independent of local origin (Figure 1A). The Heat Map of these analyses also demonstrates that squamous cell carcinoma belongs to a different group of tumors, whereas adenocarcinomas of the esophagus resemble adenocarcinomas of the cardia and non-cardia regions of the stomach (Figure 1B).

**Table 2:** Number of differentially expressed mRNAs and microRNAs in each analysis

Analysis	Number of D. E. mRNAs	Number of D.E. microRNAs
(SNCA+SCA+EA) vs. ES	2366	61
EA vs. ES	2550	60
SNCA vs. EA	512	7
(SNCA + SCA) vs. EA	375	5
(SCA+EA) vs. SNCA	186	2

Tissue discrimination was given as follows: stomach non-cardia adenocarcinoma (SNCA), stomach cardia adenocarcinoma (SCA), esophageal adenocarcinoma (EA) and esophageal squamous cell carcinoma (ES).

**Figure 2:**
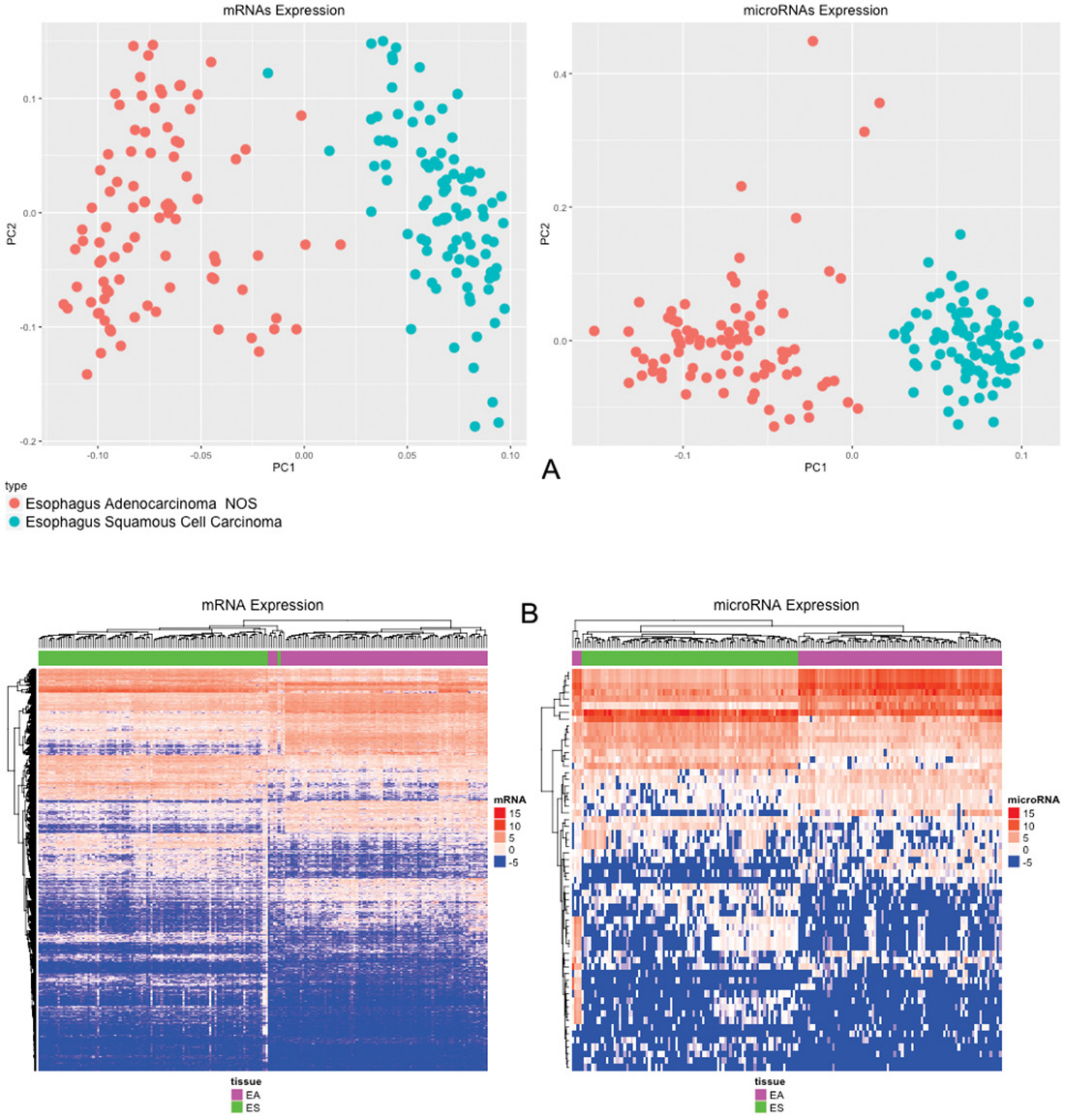
**(A)** PCA of 20,349 mRNAs and 910 microRNAs; **(B)** Heat Map of 2550 mRNAs and 60 microRNAs differentially expressed (p-value < 0.05; |Log_2_(fold-change)| > 2) comparing esophageal adenocarcinomas and esophageal squamous cell carcinomas.

The PCA (Principal Component Analysis) clearly categorized esophageal adenocarcinomas and esophageal squamous cell carcinomas as two different cancers (Figure 2A).

The Heat Map of differentially expressed mRNAs and microRNAs (Figure 2B) strongly demonstrates that esophageal adenocarcinomas and esophageal squamous cell carcinomas are different entities that should not be classified together as a unique tumor, according to the mRNA expression patterns.

**Figure 3:**
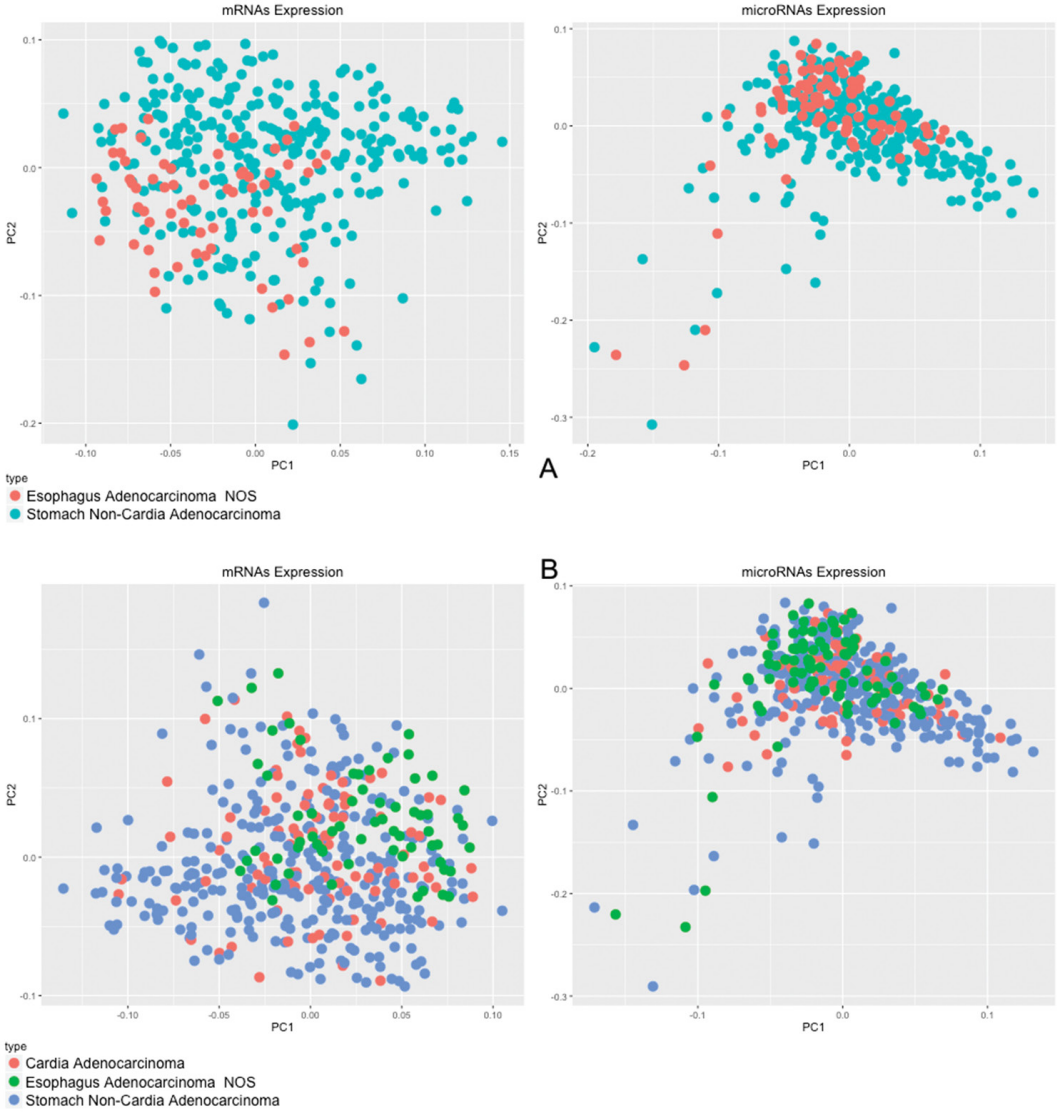
PCA of 20,349 mRNAs and 910 microRNAs In **(A)** comparison between esophagus adenocarcinomas and non-cardia gastric adenocarcinomas; in **(B)** comparison between lower esophagus, gastric cardia region and non-cardia gastric adenocarcinomas.

According to mRNA and microRNA expression profile, even distal tumors of the stomach are quite similar to adenocarcinomas of the lower esophagus, demonstrating that lower esophageal adenocarcinomas are more similar to gastric cancers than esophageal cancers. The PCA plot robustly represents this observation (Figure 3A).

By analyzing adenocarcinomas from the lower esophagus, adenocarcinomas of the gastric cardia region, and adenocarcinomas of the gastric non-cardia region, it was possible to identify that the mRNA and microRNA expression patterns of these tumors are quite similar (Figure 3B). Briefly, based on this molecular fingerprint, adenocarcinomas of the lower esophagus and adenocarcinomas of the cardia region of stomach could be classified as gastric cancers.

Finally, to determine whether the unique differences among these tumors are related to histological type, i.e., adenocarcinoma or squamous cell carcinoma, we also analyzed samples of colon adenocarcinomas and compared these samples with all of the tumors of esophagus and stomach regions previously analyzed.

**Figure 4:**
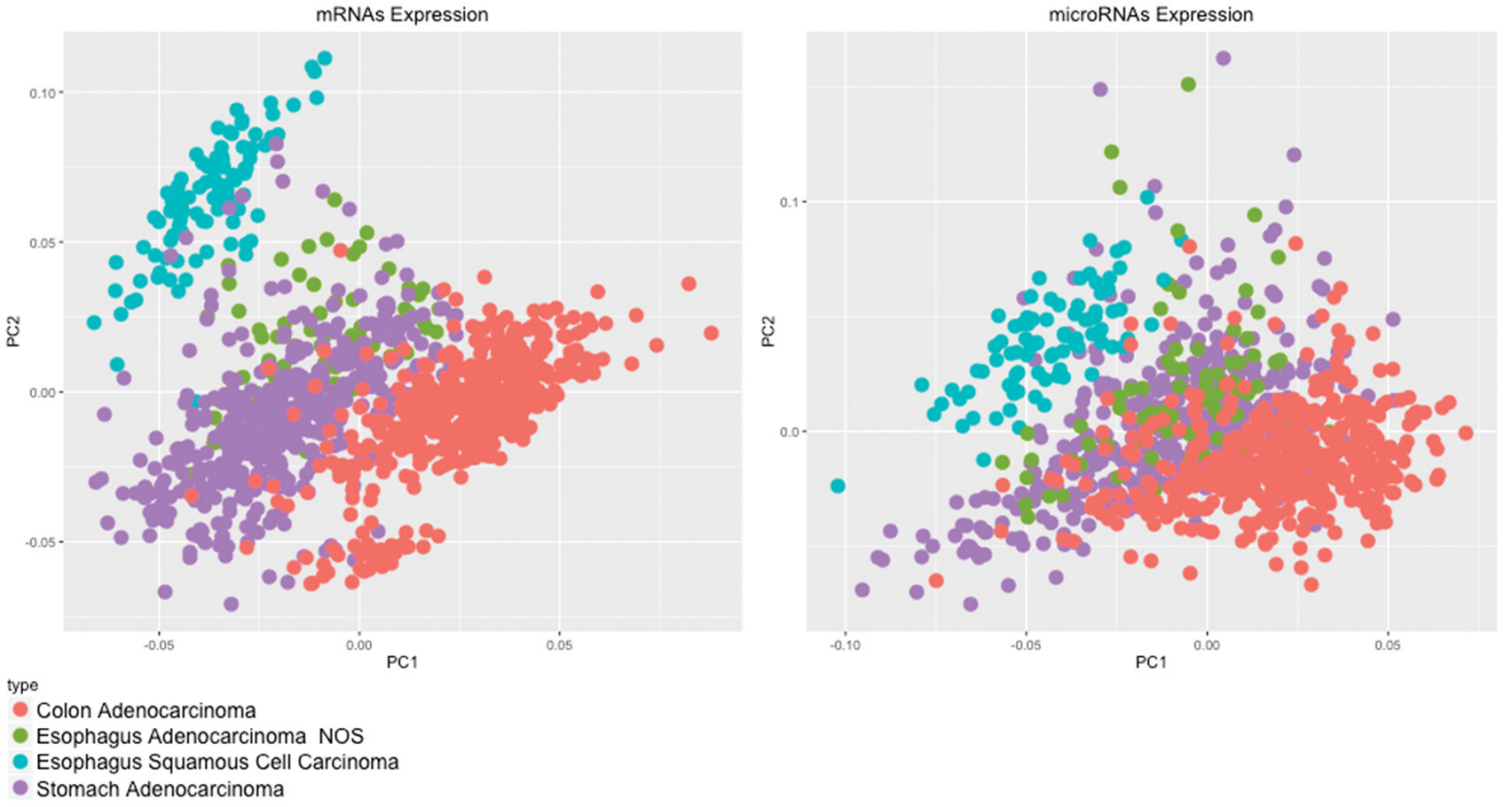
PCA of 20,349 mRNAs and 910 microRNAs from gastric cancer, esophageal squamous cell carcinoma and colon adenocarcinoma samples

The PCA images clearly demonstrated that adenocarcinomas of the colon are different from adenocarcinomas of the stomach and esophagus, thus strengthening the conclusions that GEJ tumors (including cardia cancers and esophageal adenocarcinomas from The Cancer Genome Atlas – TCGA) exhibit mRNA expression patterns similar to that of non-cardia stomach cancers and are extremely different from squamous cell esophagus carcinomas. This finding allows these tumors to be classified as gastric cancers instead of esophageal cancers. In addition, GEJ cancers were distinguished from both esophageal squamous cell carcinomas and colon adenocarcinomas (Figure 4).

## DISCUSSION

Staging GEJ cancer remains a challenge because a special group of tumors integrates this entity, which encompasses two peculiar organs. Nevertheless, the UICC 7^th^ and 8^th^ edition classified GEJ tumors as esophageal cancer. In addition, many clinicians and researchers include these cancers in the group of stomach cancers when reporting clinical and basic research data.

Different approaches have been applied to elucidate the best mode to stage and, consequently, define clinical management in GEJ cancers. Frequently, these approaches take advantage of institutional or even personal case series and compare the 6^th^ and 7^th^ UICC editions to determine which method better reproduces their expected results in terms of surgical and clinical outcomes [3, 7].

Comparing the 6^th^ and 7^th^ UICC staging systems in these case series resulted in conflicting findings due to different approaches and sample peculiarities. Thus, these studies were unable to shed light on this matter [3, 7].

Aiming at removing personal treatment and sample biases, we took advantage of the most important available cancer data bank, named TCGA. We also sought to perform an objective search of information: the genetic features of tumors.

The UICC staging does not differentiate esophageal adenocarcinomas from squamous cell esophageal carcinomas. Thus, for the initial analyses, every esophageal cancer that was available from TCGA, from both histological subtypes, was included.

Since the analyzed data was extracted from TCGA database, and not reviewed case by case by the pathologist author, the topographic accuracy of the tumors might have some misinterpretation, nevertheless the quality control of TCGA registries.

Despite tumor heterogeneity, it was strongly demonstrated that GEJ are much more similar to gastric cancers than they are to esophageal cancers. Using data from every expressed gene (mRNAs from 20,349 genes), the GEJ tumors clustered to gastric cancers instead of esophageal cancers according to the mRNA expression analyses. Regarding microRNAs (910 expressed microRNAs) the results were identical. This preliminary observation suggests that moving GEJ cancers from the stomach to esophageal staging group is not supported by molecular fingerprint analyses.

According to the differential expression analysis, there is a small number of mRNAs and microRNAs differentially expressed when comparing samples from non-cardia gastric adenocarcinomas, cardia adenocarcinomas and esophageal adenocarcinomas, indicating that these cancers have similar expression profile. Otherwise, when comparing these samples with esophageal squamous cell samples, the number of differentially expressed mRNAs and microRNAs increase greatly (Table 2; Supplementary Figure 1). These finds corroborates PCAs and support the suggestion that esophageal and cardia adenocarcinomas expression profiles are more similar to gastric adenocarcinoma than to esophageal cancers.

A possible major inconvenience of this first round of comparison is related to the eventual influence of squamous cell cancers in separating esophageal from cardia cancers. Regarding squamous cell cancers, it was strongly demonstrated, either by mRNAs or microRNAs expression patterns, that this histological type of tumor is significantly different from every group of adenocarcinomas, including non-cardia gastric adenocarcinomas, cardia adenocarcinomas and esophageal adenocarcinomas. Focusing exclusively on esophageal cancers, adenocarcinomas and squamous cell carcinomas of the esophagus are clearly different, thus raising a discussion regarding the traditional staging of these so different tumors as the same entity. In addition to their molecular fingerprints, their histology, epidemiology, risk factors, aggressiveness, and clinical management are completely different [36–38]; thus, this classification should be avoided in clinical practice.

In addition, when comparing esophageal adenocarcinomas to non-cardia gastric adenocarcinomas, no important discriminating differences were observed regarding mRNAs and microRNAs expression patterns. These data reinforce the notion that the placement of GEJ adenocarcinomas in the gastric cancer staging group is much less deleterious than classifying them as esophagus cancers.

Finally, assessing the global data, it could be concluded that the only real difference among these tumors involves the histological type, i.e., adenocarcinoma or squamous cell carcinoma, regardless of the location or organ of origin. To elucidate this notion, we compared the mRNA expression of non-cardia gastric adenocarcinomas, cardia adenocarcinomas and distal esophagus adenocarcinomas with adenocarcinomas of the colon, as well as the microRNAs expression of these tumors. According to the investigated molecular signatures, it was possible to clearly discriminate the organ of origin if colon adenocarcinoma or adenocarcinomas from stomach, esophagus and GEJ adenocarcinomas, thus demonstrating that the issues are much more complex than the tumor histologic type.

The provided data enable the authors to suggest the inclusion of an additional criterion to categorize these tumors – the molecular identity – by UICC. In special, in case of the discussed tumors, it should be reasonable to review the current classification of squamous cell and adenocarcinoma of the esophagus as a unique entity. Adenocarcinoma of GEJ might also be classified in the same gastric cancer staging group, or even as an additional specific tumor category.

In conclusion, by using robust molecular fingerprints of a large series of tumors from the most important available cancer data bank, it was strongly demonstrated that GEJ tumors more closely resemble gastric cancers than esophagus cancers, despite tumor heterogeneity.

## MATERIALS AND METHODS

### Cancer samples

To better estimate the similarities and differences in mRNA expressions patterns, we took advantage of TCGA and downloaded data (http://gdc.cancer.gov) regarding mRNA and microRNA expression, as well as tumor location and histologic types from gastric, esophagus, GEJ and colorectal cancers. Stomach cancers were categorized as cardia cancers (typically reported as GEJ tumors) and non-cardia cancers according to the available TCGA clinical data. Cases without clear definitions of tumor site or histological information were excluded.

Esophageal cancers were also classified as adenocarcinomas, which corresponds to tumors of the lower esophagus (usually included as GEJ tumors), and squamous cell carcinoma, as defined in TCGA data. Additionally, to determine whether mRNA and microRNA expression patterns differentiate adenocarcinomas of the stomach and GEJ from other organ adenocarcinomas instead of only being able to distinguish squamous cell carcinoma from adenocarcinoma, we included colon adenocarcinomas analyses.

### Data analysis

The clinical and expression data used to perform the analysis were downloaded from TCGA. For the mRNA analysis, 395 stomach samples, 184 esophagus samples and 521 colon samples were used (1100 samples in total). For the microRNA analysis, 392 stomach samples, 175 esophagus samples and 459 colon samples were used (1026 samples in total), to avoid outliers, all samples used in microRNAs analysis were also present in mRNA analysis.

For each differential analysis, we excluded samples from non-cancer tissues and then excluded those mRNAs and microRNAs that exhibited no expression in all samples. The main reason for excluding non-cancer tissues adjacent to tumor samples was the possible misinterpretation related to the cancer field effect [39].

Both Principal Component Analysis (PCA) and the Heat Map plots were performed in R platform (http://www.rproject.org) using a Log_2_ transformation of RPKM (Reads Per Kilobase per Million mapped reads) normalized data. Differential expression analysis was also performed in R, using RAW data and the DESeq2 package [40, 41]. To avoid the multiple test problem, Benjamini-Hochberg False Discovery Rate (FDR) corrections were used. The mRNAs and microRNAs were tagged as differentially expressed if they met the following criteria: i) FDR adjusted p-value < 0.05 and ii) |Log_2_(fold-change)| > 2 (expressions greater or lower than four-fold change).

## SUPPLEMENTARY MATERIALS FIGURE AND TABLE





## Abbreviations

UICC-AJCC: Union for International Cancer Control–American Joint Committee on Cancer
TNM: Tumor, Node and Metastasis staging system
GEJ: Gastroesophageal Junction
D.E: Differential Expression
SCA: Stomach Cardia
SNCA: Stomach Non-Cardia
EA: Esophageal Adenocarcinoma
ES: Esophageal Squamous cell carcinoma
TCGA: The Cancer Genome Atlas
PCA: Principal Component Analysis
RPKM: Reads Per Kilobase per Million mapped reads
FDR: False Discovery Rate
