# Supplementary material for: GEJ cancers: gastric or esophageal tumors? searching for the answer according to molecular identity
Source: Oncotarget. 2017 Oct 31;8(61):104286–94. doi: 10.18632/oncotarget.22216 (PMC5732806; doi:10.18632/oncotarget.22216)
Supplement: Supplementary file 1 [file oncotarget-08-104286-s001.pdf]

GEJ cancers: gastric or esophageal tumors? searching for the answer according to molecular identity

SUPPLEMENTARY MATERIALS

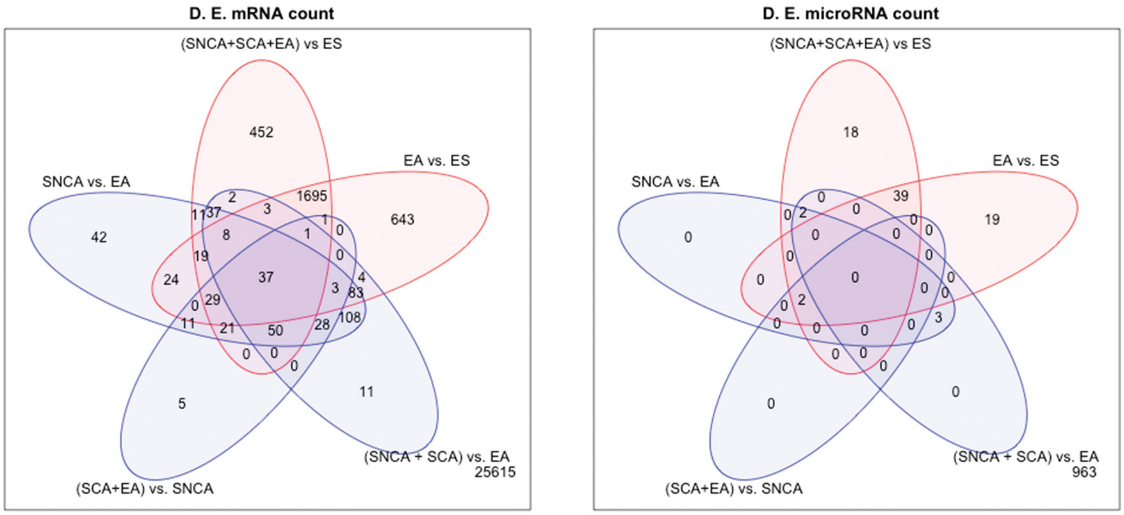

Supplementary Figure 1: Venn diagram with the number of differentially expressed mRNA and miRNAs in each analysis. Tissue discrimination was given as follows: stomach non-cardia adenocarcinoma (SNCA), stomach cardia adenocarcinoma (SCA), esophageal adenocarcinoma (EA) and esophageal squamous cell carcinoma (ES).

Supplementary Table 1: mRNAs and microRNAs differentially expressed in each analysis. Tissue discrimination was given as follows: stomach non-cardia adenocarcinoma (SNCA), stomach cardia adenocarcinoma (SCA), esophageal adenocarcinoma (EA) and esophageal squamous cell carcinoma (ES)

See Supplementary File 1
