# Supplementary material for: GEJ cancers: gastric or esophageal tumors? searching for the answer according to molecular identity
Source: Oncotarget. 2017 Oct 31;8(61):104286–94. doi: 10.18632/oncotarget.22216 (PMC5732806; doi:10.18632/oncotarget.22216)
Supplement: Supplementary file 2 [file oncotarget-08-104286-s002.docx]

Table S1

| **(SNCA+SCA+EA) vs. ES** | | | | | | | |
| --- | --- | --- | --- | --- | --- | --- | --- |
| **mRNAs** | **log2 Fold Change** | **p value** | **p adj** | **microRNAs** | **log2 Fold Change** | **p value** | **p adj** |
| *ABCA4* | -5.272546123 | 6.78E-150 | 1.29E-147 | *hsa-mir-122* | 2.031960156 | 3.88E-06 | 1.61E-05 |
| *ACTL8* | 2.506067044 | 5.76E-13 | 2.59E-12 | *hsa-mir-1224* | 2.74269911 | 5.73E-16 | 5.90E-15 |
| *AGMAT* | 3.159188083 | 1.58E-159 | 3.47E-157 | *hsa-mir-1251* | 2.433486193 | 8.18E-08 | 4.37E-07 |
| *AGT* | 4.81383968 | 3.82E-148 | 6.96E-146 | *hsa-mir-1266* | 2.232140889 | 1.63E-51 | 7.22E-50 |
| *AJAP1* | -2.338051318 | 1.75E-37 | 2.67E-36 | *hsa-mir-1293* | -3.675033577 | 9.28E-50 | 3.67E-48 |
| *AK025975* | 2.592375279 | 5.97E-33 | 7.63E-32 | *hsa-mir-1305* | -2.549406565 | 3.12E-16 | 3.36E-15 |
| *AK054726* | -2.815606064 | 4.84E-12 | 2.04E-11 | *hsa-mir-137* | -2.568970136 | 1.82E-14 | 1.72E-13 |
| *AK056431* | -3.071616642 | 2.01E-50 | 4.72E-49 | *hsa-mir-147b* | 2.556393172 | 3.03E-34 | 7.42E-33 |
| *AK124056* | -2.497568286 | 7.94E-47 | 1.64E-45 | *hsa-mir-149* | -2.793951613 | 7.89E-71 | 5.61E-69 |
| *AK309744* | -2.233836021 | 2.03E-22 | 1.59E-21 | *hsa-mir-153-1* | 2.008856811 | 7.87E-24 | 1.30E-22 |
| *AKR7A3* | 4.765210233 | 5.67E-179 | 1.69E-176 | *hsa-mir-153-2* | 2.742934963 | 7.81E-58 | 4.63E-56 |
| *AKR7L* | 3.600499024 | 1.05E-135 | 1.60E-133 | *hsa-mir-187* | -2.277708956 | 1.73E-17 | 2.01E-16 |
| *AMPD1* | 2.583723918 | 9.18E-33 | 1.17E-31 | *hsa-mir-1910* | -3.28810362 | 2.06E-32 | 4.88E-31 |
| *APOA2* | 4.960491286 | 8.56E-50 | 1.95E-48 | *hsa-mir-192* | 6.723275783 | 0 | 0 |
| *APOBEC4* | 3.195690662 | 1.01E-45 | 2.02E-44 | *hsa-mir-194-1* | 6.637401211 | 0 | 0 |
| *ARTN* | -3.534486832 | 9.59E-95 | 6.34E-93 | *hsa-mir-194-2* | 6.548831238 | 0 | 0 |
| *AX229788* | 2.90988926 | 4.73E-10 | 1.72E-09 | *hsa-mir-203* | -2.571635044 | 5.19E-50 | 2.17E-48 |
| *AX748428* | 3.238813313 | 1.19E-47 | 2.53E-46 | *hsa-mir-205* | -5.910116454 | 3.20E-44 | 1.14E-42 |
| *BC016143.1* | -5.18502073 | 1.49E-136 | 2.29E-134 | *hsa-mir-215* | 8.217892531 | 8.78E-308 | 1.56E-305 |
| *BC016972* | 2.963925392 | 9.06E-98 | 6.34E-96 | *hsa-mir-224* | -2.867373001 | 3.93E-55 | 2.00E-53 |
| *BC030750* | 2.247978388 | 2.23E-111 | 2.03E-109 | *hsa-mir-2276* | 2.221911302 | 3.82E-07 | 1.86E-06 |
| *BC030768* | 2.969994884 | 5.44E-35 | 7.50E-34 | *hsa-mir-302a* | 3.75040682 | 1.80E-05 | 6.79E-05 |
| *BC032040* | -2.957822434 | 2.29E-15 | 1.21E-14 | *hsa-mir-302b* | 3.230741508 | 0.000162211 | 0.000544018 |
| *BC037321* | -2.216644679 | 2.72E-28 | 2.86E-27 | *hsa-mir-3131* | 6.606189154 | 3.89E-39 | 1.15E-37 |
| *BC038769* | -2.039519269 | 1.35E-29 | 1.50E-28 | *hsa-mir-3166* | -2.860734426 | 1.69E-17 | 2.00E-16 |
| *BC042538* | 4.673841702 | 4.14E-34 | 5.56E-33 | *hsa-mir-3171* | -2.04668646 | 0.000264564 | 0.000847319 |
| *BC043541* | -2.037823159 | 8.18E-18 | 4.98E-17 | *hsa-mir-3186* | 2.278177546 | 0.002100511 | 0.005856719 |
| *BC067883* | -2.469810521 | 4.82E-09 | 1.62E-08 | *hsa-mir-3189* | 4.735885265 | 8.29E-21 | 1.23E-19 |
| *BC127868* | -2.331162819 | 1.77E-08 | 5.68E-08 | *hsa-mir-326* | 2.004756503 | 6.42E-47 | 2.40E-45 |
| *BCL2L15* | 5.73117059 | 1.47E-299 | 1.84E-296 | *hsa-mir-34b* | -2.940843285 | 1.58E-81 | 1.25E-79 |
| *BNIPL* | -3.084838003 | 8.14E-37 | 1.21E-35 | *hsa-mir-34c* | -3.215474708 | 8.96E-113 | 1.06E-110 |
| *BRDT* | -2.976237611 | 2.59E-14 | 1.27E-13 | *hsa-mir-3659* | -2.501544187 | 1.02E-07 | 5.39E-07 |
| *C1orf110* | -3.471790861 | 9.68E-25 | 8.60E-24 | *hsa-mir-371* | 4.970869228 | 1.77E-09 | 1.09E-08 |
| *C1orf130* | 3.190978525 | 2.44E-68 | 9.17E-67 | *hsa-mir-372* | 4.304727322 | 1.35E-18 | 1.65E-17 |
| *C1orf180* | 2.750991369 | 2.71E-20 | 1.90E-19 | *hsa-mir-373* | 2.829613795 | 6.26E-05 | 0.000220256 |
| *C1orf65* | -2.208775894 | 2.75E-14 | 1.35E-13 | *hsa-mir-374c* | 3.783771032 | 7.13E-07 | 3.27E-06 |
| *C1orf68* | -4.619724376 | 1.54E-15 | 8.17E-15 | *hsa-mir-375* | 4.378364136 | 1.43E-96 | 1.27E-94 |
| *C4BPA* | 5.943833972 | 6.41E-105 | 5.24E-103 | *hsa-mir-3927* | -2.931277897 | 1.95E-05 | 7.29E-05 |
| *C4BPB* | 6.552646065 | 1.51E-215 | 7.16E-213 | *hsa-mir-3937* | 2.077312254 | 0.011801137 | 0.02706648 |
| *C8A* | 4.157329868 | 3.27E-31 | 3.91E-30 | *hsa-mir-452* | -2.287479468 | 1.33E-54 | 6.29E-53 |
| *CACNA1E* | 2.574567614 | 4.02E-30 | 4.57E-29 | *hsa-mir-490* | 2.040177017 | 2.76E-09 | 1.65E-08 |
| *CAPN8* | 5.904066786 | 6.96E-211 | 3.14E-208 | *hsa-mir-508* | -2.657925556 | 1.82E-29 | 4.04E-28 |
| *CAPN9* | 4.822242994 | 2.70E-85 | 1.46E-83 | *hsa-mir-509-1* | -2.251275181 | 1.65E-15 | 1.63E-14 |
| *CELA3B* | 4.965267245 | 9.55E-66 | 3.33E-64 | *hsa-mir-509-2* | -2.289777581 | 1.85E-16 | 2.06E-15 |
| *CELF3* | 3.205654075 | 3.32E-55 | 8.91E-54 | *hsa-mir-509-3* | -2.276651581 | 1.26E-16 | 1.43E-15 |
| *CELSR2* | -2.649902195 | 2.23E-98 | 1.57E-96 | *hsa-mir-514-1* | -2.672490253 | 1.26E-18 | 1.58E-17 |
| *CFHR2* | 2.150795261 | 0.000901486 | 0.001796765 | *hsa-mir-514-2* | -2.701805544 | 2.19E-19 | 2.88E-18 |
| *CGN* | 2.851003787 | 1.20E-131 | 1.70E-129 | *hsa-mir-514-3* | -3.350815934 | 1.35E-26 | 2.67E-25 |
| *CHIA* | 3.776734212 | 3.47E-12 | 1.48E-11 | *hsa-mir-552* | 7.729138109 | 5.54E-105 | 5.62E-103 |
| *CLCA1* | 7.576686504 | 1.53E-89 | 8.92E-88 | *hsa-mir-559* | 2.029522098 | 1.44E-12 | 1.18E-11 |
| *CLCA2* | -4.826371215 | 2.36E-35 | 3.30E-34 | *hsa-mir-577* | 2.198557438 | 1.10E-27 | 2.29E-26 |
| *CLCA4* | -2.124178753 | 3.21E-08 | 1.01E-07 | *hsa-mir-614* | 2.808750314 | 1.93E-06 | 8.32E-06 |
| *CNR2* | 2.457654054 | 1.86E-21 | 1.39E-20 | *hsa-mir-622* | 2.221666795 | 0.001729553 | 0.004879811 |
| *CR2* | 2.491318609 | 3.74E-18 | 2.32E-17 | *hsa-mir-662* | 2.69621264 | 9.75E-05 | 0.000333438 |
| *CR936677* | -2.830331137 | 1.39E-19 | 9.39E-19 | *hsa-mir-670* | 2.382800722 | 0.006317498 | 0.015760495 |
| *CRABP2* | -2.25774541 | 1.38E-23 | 1.16E-22 | *hsa-mir-708* | -2.05865787 | 2.76E-56 | 1.51E-54 |
| *CRCT1* | -3.06491824 | 5.53E-10 | 2.01E-09 | *hsa-mir-802* | 6.622032291 | 2.44E-25 | 4.45E-24 |
| *CTSE* | 9.705619483 | 0 | 0 | *hsa-mir-876* | -2.28204651 | 9.50E-11 | 6.69E-10 |
| *CYMP* | 3.815091621 | 7.37E-37 | 1.10E-35 | *hsa-mir-934* | 2.293384319 | 9.67E-10 | 6.14E-09 |
| *CYP4Z2P* | -2.144447299 | 4.43E-12 | 1.88E-11 | *hsa-mir-935* | 2.449320508 | 2.25E-26 | 4.32E-25 |
| *DDAH1* | 3.020893342 | 5.94E-229 | 3.40E-226 | *hsa-mir-944* | -6.507092514 | 7.88E-122 | 1.12E-119 |
| *DKFZp686N1631* | -2.742651563 | 5.09E-51 | 1.21E-49 |  |  |  |  |
| *DM075093* | 3.345910126 | 3.38E-94 | 2.22E-92 |  |  |  |  |
| *DMRTA2* | -2.886273162 | 2.91E-18 | 1.81E-17 |  |  |  |  |
| *DNALI1* | 2.540302663 | 6.58E-60 | 2.04E-58 |  |  |  |  |
| *DQ574660.1* | -2.764275292 | 1.22E-14 | 6.15E-14 |  |  |  |  |
| *DQ575010.1* | -2.638719683 | 8.24E-17 | 4.73E-16 |  |  |  |  |
| *DQ575955.1* | -2.061343207 | 2.10E-16 | 1.17E-15 |  |  |  |  |
| *DUSP27* | 6.46296145 | 1.20E-68 | 4.59E-67 |  |  |  |  |
| *EDN2* | -3.038838208 | 8.85E-38 | 1.36E-36 |  |  |  |  |
| *EPHA10* | 3.090275546 | 2.00E-103 | 1.59E-101 |  |  |  |  |
| *EPS8L3* | 6.63989961 | 4.70E-284 | 4.62E-281 |  |  |  |  |
| *ERVMER61-1* | 2.686023598 | 1.61E-07 | 4.74E-07 |  |  |  |  |
| *ETNK2* | -2.080537708 | 4.54E-33 | 5.85E-32 |  |  |  |  |
| *EU250746* | 2.138441423 | 9.68E-15 | 4.89E-14 |  |  |  |  |
| *F5* | 4.683226162 | 1.15E-113 | 1.11E-111 |  |  |  |  |
| *FAM163A* | 2.093479154 | 3.31E-27 | 3.30E-26 |  |  |  |  |
| *FAM177B* | 4.815136985 | 9.63E-80 | 4.72E-78 |  |  |  |  |
| *FCAMR* | 2.583947985 | 1.24E-26 | 1.20E-25 |  |  |  |  |
| *FCGR3B* | 2.617551047 | 1.61E-36 | 2.37E-35 |  |  |  |  |
| *FCRL2* | 2.047644265 | 6.35E-19 | 4.12E-18 |  |  |  |  |
| *FLG2* | -4.701337172 | 8.20E-41 | 1.40E-39 |  |  |  |  |
| *FLG* | -4.756325705 | 6.83E-75 | 2.98E-73 |  |  |  |  |
| *FLJ42875.1* | 4.342839244 | 1.61E-78 | 7.65E-77 |  |  |  |  |
| *FLJ42875.2* | 3.792378927 | 1.82E-93 | 1.17E-91 |  |  |  |  |
| *FMO5* | 3.728321982 | 1.37E-130 | 1.91E-128 |  |  |  |  |
| *FMO6P* | -2.625078 | 9.43E-23 | 7.52E-22 |  |  |  |  |
| *GBP6* | -3.106422159 | 1.98E-19 | 1.33E-18 |  |  |  |  |
| *GCLM* | -2.23753076 | 6.76E-114 | 6.64E-112 |  |  |  |  |
| *GIPC2* | 2.466234957 | 1.83E-62 | 6.05E-61 |  |  |  |  |
| *GJB5* | -2.866351152 | 1.79E-31 | 2.17E-30 |  |  |  |  |
| *GOLT1A* | 4.743659238 | 6.55E-148 | 1.18E-145 |  |  |  |  |
| *GPA33* | 7.345334872 | 8.16E-214 | 3.80E-211 |  |  |  |  |
| *GRHL3* | -2.893849809 | 5.62E-37 | 8.43E-36 |  |  |  |  |
| *GUCA2A* | 7.634774539 | 3.87E-96 | 2.64E-94 |  |  |  |  |
| *GUCA2B* | 5.011824224 | 5.24E-53 | 1.31E-51 |  |  |  |  |
| *HES2* | -3.596369918 | 1.00E-70 | 4.05E-69 |  |  |  |  |
| *HMGCS2* | 7.331427453 | 1.53E-151 | 2.98E-149 |  |  |  |  |
| *HRNR* | -2.258399746 | 2.48E-30 | 2.84E-29 |  |  |  |  |
| *HSD3B1* | 2.506207264 | 3.43E-08 | 1.07E-07 |  |  |  |  |
| *HSD3B2* | 2.19722809 | 4.54E-14 | 2.20E-13 |  |  |  |  |
| *HTR1D* | 3.085694697 | 1.00E-48 | 2.21E-47 |  |  |  |  |
| *IFFO2* | -2.025224717 | 3.07E-67 | 1.12E-65 |  |  |  |  |
| *IGSF3* | -2.543286038 | 1.10E-126 | 1.42E-124 |  |  |  |  |
| *IL12RB2* | -2.968453289 | 6.54E-83 | 3.46E-81 |  |  |  |  |
| *IL20* | -4.25679785 | 1.05E-66 | 3.76E-65 |  |  |  |  |
| *IRF6* | -2.197186975 | 1.89E-118 | 2.02E-116 |  |  |  |  |
| *ITLN1* | 7.574250664 | 6.11E-127 | 7.99E-125 |  |  |  |  |
| *IVL* | -3.269394864 | 6.54E-10 | 2.36E-09 |  |  |  |  |
| *KCNA10* | 2.882579065 | 8.98E-20 | 6.13E-19 |  |  |  |  |
| *KCNA3* | 2.188822948 | 8.07E-37 | 1.20E-35 |  |  |  |  |
| *KCNH1* | -2.971859307 | 1.71E-77 | 7.86E-76 |  |  |  |  |
| *KCNJ10* | 2.203034222 | 2.88E-32 | 3.60E-31 |  |  |  |  |
| *KIAA1324* | 2.446100165 | 2.77E-31 | 3.32E-30 |  |  |  |  |
| *KISS1* | 2.977810457 | 5.66E-43 | 1.03E-41 |  |  |  |  |
| *KLHDC7A* | 3.412627013 | 2.45E-59 | 7.39E-58 |  |  |  |  |
| *KNCN* | 2.044424649 | 6.98E-05 | 0.000158193 |  |  |  |  |
| *KPRP* | -2.684049863 | 8.72E-07 | 2.40E-06 |  |  |  |  |
| *LCE1A* | -4.212420897 | 5.09E-11 | 1.99E-10 |  |  |  |  |
| *LCE1B* | -2.726221878 | 1.91E-09 | 6.64E-09 |  |  |  |  |
| *LCE1C* | -3.954634711 | 8.60E-23 | 6.88E-22 |  |  |  |  |
| *LCE1F* | -5.10070124 | 4.08E-20 | 2.84E-19 |  |  |  |  |
| *LCE2A* | -3.028071415 | 4.02E-05 | 9.36E-05 |  |  |  |  |
| *LCE2B* | -2.9065402 | 0.000242819 | 0.000518025 |  |  |  |  |
| *LCE2C* | -3.678644393 | 3.06E-07 | 8.76E-07 |  |  |  |  |
| *LCE2D* | -3.302621434 | 1.07E-05 | 2.65E-05 |  |  |  |  |
| *LCE3A* | -4.25573344 | 3.84E-10 | 1.41E-09 |  |  |  |  |
| *LCE3C* | -4.342808583 | 2.41E-10 | 9.01E-10 |  |  |  |  |
| *LCE3D* | -5.565103125 | 2.67E-25 | 2.43E-24 |  |  |  |  |
| *LCE3E* | -5.624741567 | 1.09E-22 | 8.64E-22 |  |  |  |  |
| *LCE5A* | -2.888745885 | 4.63E-13 | 2.11E-12 |  |  |  |  |
| *LCE6A* | -2.645830188 | 3.77E-06 | 9.75E-06 |  |  |  |  |
| *LEFTY1* | 6.155944344 | 4.37E-93 | 2.79E-91 |  |  |  |  |
| *LHX8* | -2.718282495 | 3.52E-12 | 1.50E-11 |  |  |  |  |
| *LHX9* | -2.128039146 | 5.06E-09 | 1.70E-08 |  |  |  |  |
| *LOC100129269* | -2.332197393 | 1.37E-28 | 1.47E-27 |  |  |  |  |
| *LOC100133445* | 2.266243009 | 5.67E-141 | 9.38E-139 |  |  |  |  |
| *LOC115110* | 3.12856777 | 1.85E-120 | 2.05E-118 |  |  |  |  |
| *LOC127841* | 3.448866536 | 1.96E-106 | 1.63E-104 |  |  |  |  |
| *LOC149086* | 2.421352069 | 1.90E-14 | 9.40E-14 |  |  |  |  |
| *LOC254099* | 3.731113767 | 2.84E-118 | 3.03E-116 |  |  |  |  |
| *LOC284551* | 2.258109053 | 4.08E-16 | 2.25E-15 |  |  |  |  |
| *LOC339442* | -3.789728644 | 3.50E-15 | 1.82E-14 |  |  |  |  |
| *LOC339535* | -2.486880161 | 9.16E-13 | 4.07E-12 |  |  |  |  |
| *LOC646627* | 3.163014439 | 1.28E-16 | 7.27E-16 |  |  |  |  |
| *LOR* | -4.918195121 | 2.89E-71 | 1.18E-69 |  |  |  |  |
| *LPAR3* | -4.556311571 | 3.63E-78 | 1.72E-76 |  |  |  |  |
| *LRRC38* | -3.07832786 | 3.97E-35 | 5.50E-34 |  |  |  |  |
| *MAB21L3* | -2.069819013 | 1.66E-14 | 8.23E-14 |  |  |  |  |
| *MARK1* | -2.276689874 | 2.66E-49 | 5.94E-48 |  |  |  |  |
| *MFSD4* | 2.360806765 | 1.18E-34 | 1.61E-33 |  |  |  |  |
| *MGC4473* | -3.269242613 | 8.38E-24 | 7.07E-23 |  |  |  |  |
| *MIR1231* | -2.596979027 | 6.85E-53 | 1.71E-51 |  |  |  |  |
| *MIR205HG* | -5.200232257 | 1.35E-25 | 1.25E-24 |  |  |  |  |
| *MIR2682* | -2.344349566 | 7.10E-05 | 0.000160703 |  |  |  |  |
| *MIR4251* | 2.976599413 | 1.39E-08 | 4.49E-08 |  |  |  |  |
| *MIR4252* | -2.290144518 | 4.09E-14 | 1.98E-13 |  |  |  |  |
| *MIR4671* | -3.458014888 | 6.56E-28 | 6.78E-27 |  |  |  |  |
| *MIR548AA1* | -2.187053879 | 3.37E-08 | 1.05E-07 |  |  |  |  |
| *MIR548D1* | -2.187053879 | 3.37E-08 | 1.05E-07 |  |  |  |  |
| *MST1P9* | 2.453661922 | 3.55E-33 | 4.59E-32 |  |  |  |  |
| *MUC1* | 3.138487374 | 2.38E-85 | 1.30E-83 |  |  |  |  |
| *MYBPHL* | 2.196174163 | 1.80E-14 | 8.92E-14 |  |  |  |  |
| *Mir_544.3* | -2.032570145 | 4.72E-11 | 1.86E-10 |  |  |  |  |
| *NBPF7* | 2.268158453 | 4.42E-13 | 2.01E-12 |  |  |  |  |
| *NHLH2* | -2.24247852 | 1.70E-09 | 5.92E-09 |  |  |  |  |
| *NPPB* | 3.018244004 | 6.57E-11 | 2.55E-10 |  |  |  |  |
| *NR0B2* | 8.400196532 | 0 | 0 |  |  |  |  |
| *NR5A2* | 3.261758057 | 1.19E-110 | 1.07E-108 |  |  |  |  |
| *OPRD1* | 2.014739485 | 1.07E-07 | 3.20E-07 |  |  |  |  |
| *OR2T10* | 2.872450527 | 0.000182278 | 0.000393949 |  |  |  |  |
| *PADI3* | -3.508958261 | 7.43E-29 | 8.01E-28 |  |  |  |  |
| *PAX7* | -4.866234676 | 2.05E-28 | 2.17E-27 |  |  |  |  |
| *PDZK1.1* | 3.064033946 | 7.00E-54 | 1.80E-52 |  |  |  |  |
| *PGLYRP3* | -3.685911728 | 1.72E-25 | 1.59E-24 |  |  |  |  |
| *PGLYRP4* | -4.727403736 | 2.53E-61 | 8.16E-60 |  |  |  |  |
| *PIGR* | 6.09703737 | 5.12E-122 | 5.94E-120 |  |  |  |  |
| *PKP1* | -4.927027458 | 2.36E-51 | 5.68E-50 |  |  |  |  |
| *PLA2G2A* | 5.066892111 | 1.60E-72 | 6.69E-71 |  |  |  |  |
| *PLA2G2D* | 2.109787917 | 1.32E-17 | 7.97E-17 |  |  |  |  |
| *PLA2G2E* | 2.052108555 | 1.32E-06 | 3.57E-06 |  |  |  |  |
| *PLCH2* | -2.700041194 | 3.17E-47 | 6.64E-46 |  |  |  |  |
| *PLEKHA6* | 3.463846722 | 2.43E-217 | 1.17E-214 |  |  |  |  |
| *POU3F1* | -3.662778952 | 2.00E-64 | 6.81E-63 |  |  |  |  |
| *PPFIA4* | -2.293750285 | 9.97E-50 | 2.27E-48 |  |  |  |  |
| *PRDM16* | 2.758152489 | 2.87E-72 | 1.20E-70 |  |  |  |  |
| *PROX1-AS1* | 2.072381169 | 4.93E-14 | 2.38E-13 |  |  |  |  |
| *PROX1* | 2.844298336 | 2.50E-36 | 3.62E-35 |  |  |  |  |
| *PRR9* | -3.627063814 | 2.54E-11 | 1.02E-10 |  |  |  |  |
| *PVRL4* | -2.078128094 | 4.93E-43 | 9.02E-42 |  |  |  |  |
| *REG4* | 10.5579019 | 7.33E-282 | 6.95E-279 |  |  |  |  |
| *RGS7* | 3.910266522 | 4.49E-45 | 8.82E-44 |  |  |  |  |
| *RGSL1* | -2.085797797 | 4.16E-10 | 1.52E-09 |  |  |  |  |
| *RHOU* | 2.353358254 | 1.20E-99 | 8.71E-98 |  |  |  |  |
| *RIIAD1* | 2.479182683 | 5.72E-24 | 4.88E-23 |  |  |  |  |
| *RN7SK.10* | -2.5001111 | 8.71E-07 | 2.40E-06 |  |  |  |  |
| *RNF186* | 8.364740198 | 2.05E-259 | 1.48E-256 |  |  |  |  |
| *RORC* | 2.770389437 | 7.92E-53 | 1.97E-51 |  |  |  |  |
| *RPE65* | -2.417793381 | 3.96E-18 | 2.45E-17 |  |  |  |  |
| *RPTN* | -5.607579945 | 1.04E-29 | 1.16E-28 |  |  |  |  |
| *RSPO1* | 2.09474751 | 2.90E-21 | 2.14E-20 |  |  |  |  |
| *RXFP4* | 3.663693391 | 7.53E-70 | 2.94E-68 |  |  |  |  |
| *S100A12* | -3.869504551 | 1.67E-48 | 3.62E-47 |  |  |  |  |
| *S100A2* | -4.165962136 | 1.61E-43 | 3.00E-42 |  |  |  |  |
| *S100A7A* | -4.643833731 | 3.30E-21 | 2.43E-20 |  |  |  |  |
| *S100A7* | -3.046362214 | 7.23E-12 | 3.02E-11 |  |  |  |  |
| *S100A8* | -3.076018446 | 4.85E-22 | 3.73E-21 |  |  |  |  |
| *S100A9* | -2.803207766 | 7.71E-22 | 5.88E-21 |  |  |  |  |
| *SAMD13* | 2.430149392 | 2.46E-65 | 8.52E-64 |  |  |  |  |
| *SCNN1D* | -2.248196955 | 5.77E-52 | 1.41E-50 |  |  |  |  |
| *SELENBP1* | 2.485682199 | 1.57E-70 | 6.29E-69 |  |  |  |  |
| *SFN.1* | -3.056317347 | 1.39E-79 | 6.74E-78 |  |  |  |  |
| *SFN.2* | -2.998334733 | 1.89E-76 | 8.57E-75 |  |  |  |  |
| *SH2D5* | -3.169404862 | 5.44E-52 | 1.33E-50 |  |  |  |  |
| *SLC1A7* | 3.124218288 | 6.06E-63 | 2.02E-61 |  |  |  |  |
| *SLC2A1* | -2.511942378 | 8.17E-69 | 3.12E-67 |  |  |  |  |
| *SLC30A2* | 3.270670355 | 7.33E-31 | 8.63E-30 |  |  |  |  |
| *SLC35F3* | -3.570869857 | 2.92E-51 | 7.01E-50 |  |  |  |  |
| *SLC44A3* | 2.460494041 | 1.34E-133 | 1.98E-131 |  |  |  |  |
| *SLC5A9* | 3.156897541 | 6.67E-53 | 1.67E-51 |  |  |  |  |
| *SLC9C2* | 2.130905859 | 1.54E-15 | 8.17E-15 |  |  |  |  |
| *SMCP* | -3.091356722 | 3.62E-10 | 1.33E-09 |  |  |  |  |
| *SMPDL3B* | 2.754504012 | 8.44E-88 | 4.78E-86 |  |  |  |  |
| *SPRR1A* | -3.225229066 | 5.64E-14 | 2.72E-13 |  |  |  |  |
| *SPRR1B* | -3.529040828 | 1.62E-17 | 9.75E-17 |  |  |  |  |
| *SPRR2A* | -2.401060465 | 7.33E-07 | 2.03E-06 |  |  |  |  |
| *SPRR2B* | -2.947617737 | 5.76E-07 | 1.61E-06 |  |  |  |  |
| *SPRR2C* | -2.276226343 | 0.000214352 | 0.000460223 |  |  |  |  |
| *SPRR2D* | -2.804416417 | 1.38E-09 | 4.85E-09 |  |  |  |  |
| *SPRR2E* | -2.413335096 | 1.90E-05 | 4.57E-05 |  |  |  |  |
| *SPRR2G* | -4.408221507 | 2.00E-16 | 1.12E-15 |  |  |  |  |
| *SPRR4* | -6.757482476 | 2.22E-34 | 3.00E-33 |  |  |  |  |
| *SUSD4* | -2.63133529 | 2.06E-36 | 3.01E-35 |  |  |  |  |
| *SYT14* | -2.628576262 | 7.70E-25 | 6.88E-24 |  |  |  |  |
| *TCHHL1* | -3.703943705 | 5.74E-18 | 3.52E-17 |  |  |  |  |
| *TCHH* | -4.669079518 | 9.27E-132 | 1.31E-129 |  |  |  |  |
| *TMCC2* | -2.22666903 | 1.10E-81 | 5.58E-80 |  |  |  |  |
| *TMEM125* | 2.645801517 | 4.92E-120 | 5.37E-118 |  |  |  |  |
| *TMEM79* | -2.558887268 | 3.99E-76 | 1.78E-74 |  |  |  |  |
| *TMEM82* | 7.263898987 | 3.37E-192 | 1.27E-189 |  |  |  |  |
| *TNNT2* | -2.120541949 | 1.22E-30 | 1.43E-29 |  |  |  |  |
| *TP73* | -2.29006504 | 1.37E-53 | 3.51E-52 |  |  |  |  |
| *TRNP1* | 2.516149155 | 1.91E-54 | 5.02E-53 |  |  |  |  |
| *TSPAN1* | 2.736879762 | 1.10E-52 | 2.75E-51 |  |  |  |  |
| *U6.5* | 3.232465817 | 2.83E-15 | 1.48E-14 |  |  |  |  |
| *U6.8* | 2.215902682 | 8.32E-17 | 4.78E-16 |  |  |  |  |
| *UBXN10* | 2.560727939 | 3.97E-51 | 9.51E-50 |  |  |  |  |
| *VANGL2* | -2.274234233 | 2.10E-42 | 3.75E-41 |  |  |  |  |
| *VWA5B1* | 4.21821588 | 1.95E-39 | 3.17E-38 |  |  |  |  |
| *WNT3A* | -4.9462406 | 3.59E-82 | 1.84E-80 |  |  |  |  |
| *ZP4* | -3.581508775 | 1.69E-15 | 8.95E-15 |  |  |  |  |
| *ZYG11A* | -2.377705605 | 5.73E-29 | 6.20E-28 |  |  |  |  |
| *A1CF* | 7.816993195 | 5.99E-253 | 4.12E-250 |  |  |  |  |
| *ACSL5* | 3.176112239 | 1.33E-138 | 2.13E-136 |  |  |  |  |
| *ADARB2* | -2.193444429 | 4.37E-26 | 4.13E-25 |  |  |  |  |
| *ADRA2A* | 2.867979161 | 1.06E-40 | 1.81E-39 |  |  |  |  |
| *AF086154* | 2.367389587 | 1.52E-14 | 7.59E-14 |  |  |  |  |
| *AGAP11* | -2.731935355 | 5.63E-52 | 1.37E-50 |  |  |  |  |
| *AK128534* | -3.974776126 | 9.74E-44 | 1.83E-42 |  |  |  |  |
| *AK297683* | 3.646982615 | 6.56E-28 | 6.78E-27 |  |  |  |  |
| *AKR1C2* | -4.355048951 | 5.90E-72 | 2.45E-70 |  |  |  |  |
| *AKR1C3* | -2.834747443 | 1.01E-59 | 3.11E-58 |  |  |  |  |
| *AKR1C4* | 3.160422178 | 1.66E-40 | 2.81E-39 |  |  |  |  |
| *ANKRD1* | 2.271409181 | 5.28E-20 | 3.65E-19 |  |  |  |  |
| *ANXA8L1.1* | -3.63247082 | 3.00E-15 | 1.57E-14 |  |  |  |  |
| *ANXA8L1.2* | -3.583306936 | 1.46E-52 | 3.61E-51 |  |  |  |  |
| *ANXA8L2* | -3.346976439 | 7.41E-32 | 9.14E-31 |  |  |  |  |
| *ARMC3* | 2.405474572 | 4.26E-22 | 3.28E-21 |  |  |  |  |
| *ASAH2* | 3.801872132 | 3.99E-41 | 6.89E-40 |  |  |  |  |
| *BC015429* | -2.672922976 | 3.64E-11 | 1.44E-10 |  |  |  |  |
| *BC037970* | -3.499539386 | 1.37E-11 | 5.58E-11 |  |  |  |  |
| *BC051760* | -5.001927629 | 1.18E-48 | 2.58E-47 |  |  |  |  |
| *BC065757* | 2.604731729 | 4.54E-11 | 1.78E-10 |  |  |  |  |
| *BNIP3* | -2.022381748 | 1.96E-42 | 3.51E-41 |  |  |  |  |
| *BTBD16* | 2.00817485 | 4.96E-24 | 4.24E-23 |  |  |  |  |
| *C10orf108* | 4.257670507 | 1.91E-217 | 9.39E-215 |  |  |  |  |
| *C10orf112* | 3.402916106 | 1.86E-29 | 2.05E-28 |  |  |  |  |
| *C10orf136* | 2.364050323 | 1.49E-20 | 1.06E-19 |  |  |  |  |
| *C10orf81* | 3.823591747 | 2.07E-79 | 1.00E-77 |  |  |  |  |
| *C10orf90* | -2.572577949 | 5.73E-24 | 4.88E-23 |  |  |  |  |
| *C10orf99* | -3.448857171 | 8.86E-15 | 4.49E-14 |  |  |  |  |
| *CALHM3* | 2.943473407 | 1.25E-23 | 1.05E-22 |  |  |  |  |
| *CALML3* | -4.466198931 | 4.06E-24 | 3.49E-23 |  |  |  |  |
| *CALML5* | -4.416348175 | 1.67E-21 | 1.25E-20 |  |  |  |  |
| *CALY* | 2.89934625 | 5.35E-29 | 5.81E-28 |  |  |  |  |
| *CDHR1* | -2.992059703 | 3.48E-27 | 3.46E-26 |  |  |  |  |
| *CLRN3* | 6.83448925 | 1.70E-242 | 1.09E-239 |  |  |  |  |
| *COL17A1* | -3.431462925 | 2.59E-55 | 7.00E-54 |  |  |  |  |
| *CPN1* | 3.078145377 | 4.67E-13 | 2.12E-12 |  |  |  |  |
| *CYP17A1* | 3.486922882 | 1.42E-36 | 2.10E-35 |  |  |  |  |
| *CYP26A1* | -5.414413507 | 4.84E-97 | 3.34E-95 |  |  |  |  |
| *CYP2C9* | 2.041699483 | 1.13E-20 | 8.10E-20 |  |  |  |  |
| *DMBT1* | 6.533794417 | 3.65E-113 | 3.48E-111 |  |  |  |  |
| *DRGX* | 5.284686012 | 7.99E-47 | 1.65E-45 |  |  |  |  |
| *DUSP13* | -2.611153177 | 2.83E-18 | 1.76E-17 |  |  |  |  |
| *FAM25A* | -3.635552777 | 4.85E-19 | 3.17E-18 |  |  |  |  |
| *FAM25B.1* | -4.198914712 | 2.21E-17 | 1.31E-16 |  |  |  |  |
| *FAM25B.2* | -4.183515439 | 8.46E-17 | 4.85E-16 |  |  |  |  |
| *FAM25B.3* | -3.565412926 | 8.16E-23 | 6.53E-22 |  |  |  |  |
| *FXYD4* | 4.30191117 | 1.63E-60 | 5.16E-59 |  |  |  |  |
| *GDF10* | 2.402413292 | 3.27E-20 | 2.29E-19 |  |  |  |  |
| *GUCY2GP* | 2.513226684 | 6.31E-21 | 4.59E-20 |  |  |  |  |
| *HABP2* | 7.08967631 | 2.67E-192 | 1.02E-189 |  |  |  |  |
| *HKDC1* | 4.093714056 | 8.35E-115 | 8.41E-113 |  |  |  |  |
| *HMX3* | 2.631494839 | 1.00E-09 | 3.56E-09 |  |  |  |  |
| *HTR7* | -3.649606849 | 4.86E-97 | 3.34E-95 |  |  |  |  |
| *INA* | -2.485505978 | 3.14E-25 | 2.85E-24 |  |  |  |  |
| *ITIH2* | 3.367764062 | 1.06E-42 | 1.91E-41 |  |  |  |  |
| *JAKMIP3* | -2.715881626 | 8.66E-52 | 2.10E-50 |  |  |  |  |
| *LIPF* | 9.289477294 | 2.52E-108 | 2.17E-106 |  |  |  |  |
| *LIPK* | -3.871562022 | 1.41E-21 | 1.06E-20 |  |  |  |  |
| *LOC100128811* | 2.136664352 | 3.17E-13 | 1.46E-12 |  |  |  |  |
| *LOC100507127* | -3.212084563 | 9.90E-32 | 1.21E-30 |  |  |  |  |
| *LOC439990* | 2.107016082 | 4.53E-56 | 1.25E-54 |  |  |  |  |
| *LOC728643* | -2.097952171 | 1.38E-19 | 9.29E-19 |  |  |  |  |
| *LOC731789* | 3.051789037 | 3.65E-18 | 2.26E-17 |  |  |  |  |
| *LOC84856* | -2.06052289 | 6.00E-35 | 8.26E-34 |  |  |  |  |
| *LOXL4* | -2.16227761 | 1.46E-28 | 1.56E-27 |  |  |  |  |
| *LRRC18* | 2.583043839 | 1.50E-27 | 1.52E-26 |  |  |  |  |
| *M1* | 2.212208612 | 5.39E-10 | 1.96E-09 |  |  |  |  |
| *MIR4483* | 2.890213058 | 9.96E-21 | 7.16E-20 |  |  |  |  |
| *MIR4681* | 2.460518965 | 2.36E-06 | 6.22E-06 |  |  |  |  |
| *MIR608* | 3.455112736 | 2.96E-25 | 2.70E-24 |  |  |  |  |
| *MIR936* | -3.539405109 | 2.96E-52 | 7.26E-51 |  |  |  |  |
| *MSMB* | 4.645112742 | 9.55E-49 | 2.10E-47 |  |  |  |  |
| *MYO3A* | -2.594165434 | 2.50E-23 | 2.06E-22 |  |  |  |  |
| *NEUROG3* | 6.918594067 | 1.91E-87 | 1.08E-85 |  |  |  |  |
| *NKX1-2* | -4.124624568 | 1.88E-25 | 1.73E-24 |  |  |  |  |
| *NKX6-2* | 4.392435207 | 7.94E-24 | 6.72E-23 |  |  |  |  |
| *NRAP* | 5.611512222 | 4.06E-124 | 4.96E-122 |  |  |  |  |
| *NRG3* | 2.78049814 | 2.59E-30 | 2.97E-29 |  |  |  |  |
| *O3FAR1* | 3.044743849 | 1.15E-58 | 3.39E-57 |  |  |  |  |
| *OIT3* | 2.373343537 | 4.51E-50 | 1.04E-48 |  |  |  |  |
| *PBLD* | 2.74170412 | 1.08E-92 | 6.84E-91 |  |  |  |  |
| *PITX3* | -2.119523323 | 4.45E-23 | 3.63E-22 |  |  |  |  |
| *PLA2G12B* | 6.336597725 | 4.41E-74 | 1.88E-72 |  |  |  |  |
| *PNLIPRP1* | 2.945768486 | 7.64E-25 | 6.83E-24 |  |  |  |  |
| *PNLIPRP2* | 6.751291767 | 7.65E-88 | 4.36E-86 |  |  |  |  |
| *PNLIPRP3* | -5.32738555 | 1.37E-27 | 1.40E-26 |  |  |  |  |
| *PNLIP* | 2.130869243 | 5.23E-05 | 0.000120268 |  |  |  |  |
| *PPYR1* | 2.031644051 | 4.81E-22 | 3.70E-21 |  |  |  |  |
| *PRLHR* | 3.1635937 | 1.77E-13 | 8.27E-13 |  |  |  |  |
| *PTF1A* | 3.115770187 | 2.82E-10 | 1.05E-09 |  |  |  |  |
| *RBP4* | 3.936453864 | 2.20E-52 | 5.42E-51 |  |  |  |  |
| *SEMA4G* | 3.808539764 | 4.30E-273 | 3.69E-270 |  |  |  |  |
| *SFRP5* | 2.983856478 | 3.11E-27 | 3.11E-26 |  |  |  |  |
| *SFTPA2* | 4.011358907 | 2.14E-55 | 5.80E-54 |  |  |  |  |
| *SLC16A9* | -2.068216349 | 1.63E-23 | 1.36E-22 |  |  |  |  |
| *SLC18A3* | -2.462531269 | 1.94E-08 | 6.22E-08 |  |  |  |  |
| *TACR2* | 2.213763742 | 4.31E-17 | 2.51E-16 |  |  |  |  |
| *TCERG1L* | -2.710240206 | 4.60E-23 | 3.75E-22 |  |  |  |  |
| *TECTB* | 3.587241473 | 7.28E-18 | 4.45E-17 |  |  |  |  |
| *TLX1NB* | 2.217614974 | 6.35E-20 | 4.38E-19 |  |  |  |  |
| *TMEM236.1* | 2.067674064 | 1.19E-12 | 5.27E-12 |  |  |  |  |
| *TMEM72* | 2.84023125 | 6.41E-23 | 5.17E-22 |  |  |  |  |
| *TSPAN15* | 2.190079809 | 5.31E-133 | 7.73E-131 |  |  |  |  |
| *TTC40* | 2.249393748 | 5.30E-24 | 4.52E-23 |  |  |  |  |
| *U6.21* | 2.450042315 | 0.000101745 | 0.000226334 |  |  |  |  |
| *UCN3* | 5.310818025 | 5.20E-43 | 9.51E-42 |  |  |  |  |
| *VAX1* | -4.147412313 | 1.46E-23 | 1.22E-22 |  |  |  |  |
| *ZNF365* | -2.658550067 | 1.19E-38 | 1.88E-37 |  |  |  |  |
| *chromosome_10_open_reading_frame_139* | 2.988697666 | 1.04E-31 | 1.27E-30 |  |  |  |  |
| *tAKR* | -2.368383596 | 5.43E-26 | 5.12E-25 |  |  |  |  |
| *AB231721* | -2.454790803 | 1.19E-09 | 4.21E-09 |  |  |  |  |
| *AB231723* | -2.789472818 | 5.29E-30 | 5.98E-29 |  |  |  |  |
| *AB231761* | 2.877716901 | 6.02E-07 | 1.68E-06 |  |  |  |  |
| *AB429224* | 7.729934744 | 2.00E-304 | 2.62E-301 |  |  |  |  |
| *ABCC8* | 2.328447245 | 9.08E-21 | 6.54E-20 |  |  |  |  |
| *ACY3* | 3.469377611 | 9.83E-114 | 9.58E-112 |  |  |  |  |
| *ADM* | -2.06079777 | 4.11E-50 | 9.54E-49 |  |  |  |  |
| *AF085962* | 2.774256467 | 0.000425122 | 0.000883035 |  |  |  |  |
| *AK056505* | 2.288400023 | 0.000608623 | 0.001239012 |  |  |  |  |
| *AK056982* | -2.226281933 | 1.72E-47 | 3.63E-46 |  |  |  |  |
| *AK091996* | 2.631795351 | 9.05E-51 | 2.13E-49 |  |  |  |  |
| *AK095081* | 7.217690124 | 2.36E-102 | 1.84E-100 |  |  |  |  |
| *AK130852* | 2.302283619 | 0.008750929 | 0.015223461 |  |  |  |  |
| *ALDH3B1* | 2.38045362 | 1.26E-77 | 5.84E-76 |  |  |  |  |
| *ALDH3B2* | -3.330849333 | 1.69E-44 | 3.27E-43 |  |  |  |  |
| *ALX4* | -3.9461896 | 4.24E-59 | 1.27E-57 |  |  |  |  |
| *ANKK1* | -2.463586347 | 2.16E-46 | 4.41E-45 |  |  |  |  |
| *ANO1* | -2.222252856 | 2.94E-46 | 5.97E-45 |  |  |  |  |
| *APOA1* | 6.03162614 | 2.24E-97 | 1.55E-95 |  |  |  |  |
| *APOA4* | 8.005865032 | 5.76E-80 | 2.83E-78 |  |  |  |  |
| *APOA5* | 3.366028188 | 1.01E-26 | 9.80E-26 |  |  |  |  |
| *APOC3* | 6.944891741 | 1.89E-62 | 6.24E-61 |  |  |  |  |
| *ARRB1* | 2.033013965 | 2.19E-104 | 1.77E-102 |  |  |  |  |
| *ASCL2* | 2.472519871 | 9.90E-32 | 1.21E-30 |  |  |  |  |
| *ASRGL1* | 2.222738415 | 3.52E-67 | 1.28E-65 |  |  |  |  |
| *B3GNT6* | 2.392724532 | 8.05E-14 | 3.85E-13 |  |  |  |  |
| *BBOX1* | -2.289978319 | 3.07E-23 | 2.52E-22 |  |  |  |  |
| *BC008359* | -2.996840829 | 4.15E-21 | 3.05E-20 |  |  |  |  |
| *BC016143.2* | -4.674751025 | 1.37E-38 | 2.16E-37 |  |  |  |  |
| *BC021736* | -2.391333483 | 7.04E-11 | 2.73E-10 |  |  |  |  |
| *BC031305* | 2.23339642 | 0.003753903 | 0.006902533 |  |  |  |  |
| *BC031979* | -2.072167768 | 1.58E-19 | 1.06E-18 |  |  |  |  |
| *BC133018* | -2.763687067 | 1.40E-45 | 2.79E-44 |  |  |  |  |
| *BTG4* | -2.927208308 | 1.26E-17 | 7.62E-17 |  |  |  |  |
| *BX649128* | -2.253247033 | 4.00E-63 | 1.34E-61 |  |  |  |  |
| *C11orf41* | -2.089928479 | 9.50E-27 | 9.26E-26 |  |  |  |  |
| *C11orf53* | 2.621179484 | 2.00E-31 | 2.41E-30 |  |  |  |  |
| *C11orf86* | 5.316595512 | 3.40E-76 | 1.53E-74 |  |  |  |  |
| *C11orf87* | -5.822473622 | 7.86E-91 | 4.76E-89 |  |  |  |  |
| *C11orf88* | -2.400893377 | 2.16E-31 | 2.60E-30 |  |  |  |  |
| *C11orf9* | 4.676733796 | 3.66E-290 | 4.20E-287 |  |  |  |  |
| *CALCA* | 2.404355389 | 9.40E-10 | 3.35E-09 |  |  |  |  |
| *CAPN5* | 3.224442705 | 8.22E-153 | 1.61E-150 |  |  |  |  |
| *CARD18* | -4.592468753 | 2.32E-36 | 3.39E-35 |  |  |  |  |
| *CASP5* | 2.702408358 | 7.13E-40 | 1.18E-38 |  |  |  |  |
| *CCKBR* | 2.840952128 | 1.91E-18 | 1.20E-17 |  |  |  |  |
| *CDHR5* | 7.044031045 | 0 | 0 |  |  |  |  |
| *CNTN5* | -3.120991877 | 4.74E-31 | 5.62E-30 |  |  |  |  |
| *CREB3L1* | 3.29892433 | 1.65E-105 | 1.36E-103 |  |  |  |  |
| *DJ031150* | -3.456879917 | 7.82E-24 | 6.62E-23 |  |  |  |  |
| *DKFZP434K028* | 4.507219879 | 4.36E-101 | 3.27E-99 |  |  |  |  |
| *DM119532.2* | 3.652848878 | 2.11E-13 | 9.81E-13 |  |  |  |  |
| *EFCAB4A* | 2.978484058 | 2.63E-132 | 3.75E-130 |  |  |  |  |
| *F2* | 3.602264078 | 4.97E-38 | 7.70E-37 |  |  |  |  |
| *FAM181B* | -2.940885043 | 1.10E-35 | 1.56E-34 |  |  |  |  |
| *FAM55A* | 3.816423177 | 1.31E-25 | 1.21E-24 |  |  |  |  |
| *FAM55B.1* | 2.307103487 | 3.74E-16 | 2.06E-15 |  |  |  |  |
| *FAM55B.2* | 2.657882572 | 1.79E-18 | 1.13E-17 |  |  |  |  |
| *FAM55D* | 2.470358044 | 1.74E-15 | 9.22E-15 |  |  |  |  |
| *FLJ41423* | -3.588467499 | 9.00E-15 | 4.56E-14 |  |  |  |  |
| *FOLR1* | 2.68149205 | 6.63E-20 | 4.56E-19 |  |  |  |  |
| *FOLR3* | -2.281653085 | 7.47E-16 | 4.04E-15 |  |  |  |  |
| *FUT4* | 3.199450657 | 2.18E-198 | 8.71E-196 |  |  |  |  |
| *GAS2* | 2.153892181 | 7.54E-36 | 1.07E-34 |  |  |  |  |
| *GIF* | 6.923023016 | 3.42E-84 | 1.83E-82 |  |  |  |  |
| *GLYATL2* | -2.517120942 | 8.57E-25 | 7.63E-24 |  |  |  |  |
| *GRAMD1B.1* | 3.079891806 | 3.13E-77 | 1.43E-75 |  |  |  |  |
| *GRAMD1B.2* | 2.622055402 | 6.84E-55 | 1.81E-53 |  |  |  |  |
| *GRM5* | -2.510680516 | 6.31E-25 | 5.67E-24 |  |  |  |  |
| *GUCY2E* | -2.987983252 | 4.09E-21 | 3.01E-20 |  |  |  |  |
| *HBD* | 2.690443366 | 5.45E-28 | 5.64E-27 |  |  |  |  |
| *HEPHL1* | -3.361029813 | 9.57E-33 | 1.22E-31 |  |  |  |  |
| *HRASLS5* | 2.097401322 | 2.13E-18 | 1.34E-17 |  |  |  |  |
| *INSC* | 3.006003342 | 7.34E-62 | 2.39E-60 |  |  |  |  |
| *JA429539* | 2.3647371 | 7.63E-43 | 1.39E-41 |  |  |  |  |
| *KCNE3* | 2.565703888 | 2.44E-57 | 6.95E-56 |  |  |  |  |
| *KCNK7* | -2.049529625 | 4.50E-41 | 7.75E-40 |  |  |  |  |
| *KCNQ1* | 2.992927834 | 8.23E-93 | 5.22E-91 |  |  |  |  |
| *KCTD14* | 2.549646797 | 1.46E-64 | 4.98E-63 |  |  |  |  |
| *KRTAP5-1* | 2.214111099 | 6.54E-51 | 1.55E-49 |  |  |  |  |
| *KRTAP5-2* | 2.834173158 | 1.11E-75 | 4.90E-74 |  |  |  |  |
| *KRTAP5-4* | 2.374563085 | 4.34E-20 | 3.01E-19 |  |  |  |  |
| *KRTAP5-5* | 5.193941363 | 4.31E-51 | 1.03E-49 |  |  |  |  |
| *LGALS12* | 2.04370069 | 3.34E-20 | 2.33E-19 |  |  |  |  |
| *LOC100506305* | -3.698304221 | 3.19E-36 | 4.61E-35 |  |  |  |  |
| *LOC283177* | 5.732861912 | 1.89E-93 | 1.22E-91 |  |  |  |  |
| *LOC338651* | 2.727441168 | 4.91E-80 | 2.42E-78 |  |  |  |  |
| *LOC440040* | -2.29001307 | 2.64E-08 | 8.36E-08 |  |  |  |  |
| *LOC643037* | 2.931657776 | 3.35E-16 | 1.85E-15 |  |  |  |  |
| *LRRC55* | -2.344473333 | 3.03E-33 | 3.94E-32 |  |  |  |  |
| *MIR192* | 7.185587728 | 8.05E-78 | 3.75E-76 |  |  |  |  |
| *MIR194-2* | 4.982181794 | 2.17E-24 | 1.90E-23 |  |  |  |  |
| *MIR326* | 2.169344169 | 5.62E-08 | 1.72E-07 |  |  |  |  |
| *MIR483* | 2.01435675 | 0.008029756 | 0.014063197 |  |  |  |  |
| *MIR708* | -2.434876631 | 5.49E-11 | 2.14E-10 |  |  |  |  |
| *MMP10* | -3.12644246 | 1.08E-44 | 2.12E-43 |  |  |  |  |
| *MMP13* | -5.141375641 | 7.04E-92 | 4.38E-90 |  |  |  |  |
| *MOGAT2* | 3.204685293 | 4.33E-31 | 5.15E-30 |  |  |  |  |
| *MRGPRX3* | -4.582509866 | 8.67E-29 | 9.31E-28 |  |  |  |  |
| *MS4A10* | 3.621461998 | 1.39E-25 | 1.28E-24 |  |  |  |  |
| *MS4A12* | 2.401290185 | 1.40E-09 | 4.92E-09 |  |  |  |  |
| *MS4A8B* | 6.531121529 | 9.91E-151 | 1.91E-148 |  |  |  |  |
| *MUC15* | -2.155557514 | 1.55E-08 | 5.00E-08 |  |  |  |  |
| *MUC2* | 7.257929469 | 4.78E-105 | 3.93E-103 |  |  |  |  |
| *MUC5AC* | 7.914378925 | 6.21E-129 | 8.41E-127 |  |  |  |  |
| *MUC5B* | 3.646220937 | 2.06E-35 | 2.89E-34 |  |  |  |  |
| *MUC6* | 7.072099132 | 2.26E-112 | 2.12E-110 |  |  |  |  |
| *Mir_548.8* | -3.033838866 | 3.11E-22 | 2.42E-21 |  |  |  |  |
| *NAV2-AS4* | 2.786353534 | 5.65E-07 | 1.58E-06 |  |  |  |  |
| *NLRP10* | -4.301625361 | 1.55E-28 | 1.65E-27 |  |  |  |  |
| *NLRP6* | 3.42781132 | 6.98E-49 | 1.55E-47 |  |  |  |  |
| *ODZ4.1* | -2.32002861 | 2.37E-49 | 5.33E-48 |  |  |  |  |
| *ODZ4.2* | -2.076729728 | 2.24E-22 | 1.75E-21 |  |  |  |  |
| *OMP* | 3.416702467 | 7.39E-86 | 4.05E-84 |  |  |  |  |
| *OR10A6* | -2.289764435 | 3.20E-05 | 7.53E-05 |  |  |  |  |
| *OR10Q1* | 2.018986782 | 5.93E-08 | 1.81E-07 |  |  |  |  |
| *OR10S1* | -2.532827905 | 0.000107573 | 0.000238353 |  |  |  |  |
| *OR4A47* | 2.119300928 | 0.005318716 | 0.009587498 |  |  |  |  |
| *OR4S1* | 2.115978292 | 0.02549091 | 0.040924475 |  |  |  |  |
| *OR56A3* | -2.692942199 | 1.53E-06 | 4.10E-06 |  |  |  |  |
| *OR56A5* | -2.088541501 | 1.30E-07 | 3.85E-07 |  |  |  |  |
| *OR5AK2* | 2.953248784 | 0.000169197 | 0.000367289 |  |  |  |  |
| *OR5AK4P* | 2.474331414 | 1.89E-11 | 7.66E-11 |  |  |  |  |
| *OR5AN1* | 2.153335447 | 0.016233591 | 0.027008508 |  |  |  |  |
| *P2RX3* | 4.421736108 | 8.12E-59 | 2.41E-57 |  |  |  |  |
| *PC* | -2.138571455 | 3.45E-88 | 1.98E-86 |  |  |  |  |
| *PDZD3* | 4.787747407 | 1.66E-143 | 2.80E-141 |  |  |  |  |
| *PGA3* | 7.129441678 | 2.11E-46 | 4.32E-45 |  |  |  |  |
| *PGA4* | 6.419995296 | 4.00E-36 | 5.77E-35 |  |  |  |  |
| *PGA5* | 5.284121803 | 1.67E-52 | 4.12E-51 |  |  |  |  |
| *PIWIL4* | 2.694846544 | 7.36E-160 | 1.64E-157 |  |  |  |  |
| *PLA2G16* | 2.438420878 | 1.82E-66 | 6.48E-65 |  |  |  |  |
| *PLEKHB1* | 4.092843632 | 2.27E-124 | 2.80E-122 |  |  |  |  |
| *PVRL1* | -2.562945637 | 1.97E-101 | 1.49E-99 |  |  |  |  |
| *RAB38* | -3.604905466 | 7.37E-138 | 1.17E-135 |  |  |  |  |
| *RN7SK.18* | 2.240329366 | 3.32E-07 | 9.49E-07 |  |  |  |  |
| *SCGB2A1* | 3.514076272 | 1.40E-28 | 1.49E-27 |  |  |  |  |
| *SESN3* | -2.51572948 | 1.81E-70 | 7.24E-69 |  |  |  |  |
| *SLC22A11* | 2.353008894 | 8.07E-15 | 4.10E-14 |  |  |  |  |
| *SLC22A20* | -2.839289997 | 6.65E-60 | 2.06E-58 |  |  |  |  |
| *SLC22A25* | -3.095810502 | 1.70E-20 | 1.20E-19 |  |  |  |  |
| *SLC43A1* | 2.801506883 | 4.36E-112 | 4.07E-110 |  |  |  |  |
| *SPATA19* | -3.36144045 | 4.97E-18 | 3.06E-17 |  |  |  |  |
| *SYT13* | 4.563781852 | 1.09E-98 | 7.75E-97 |  |  |  |  |
| *SYTL2* | 2.452833681 | 8.21E-96 | 5.55E-94 |  |  |  |  |
| *SnoMBII_202* | 2.113989248 | 0.003481215 | 0.006434256 |  |  |  |  |
| *TBX10* | 3.601291946 | 3.63E-50 | 8.43E-49 |  |  |  |  |
| *TCN1* | 2.126806622 | 5.47E-16 | 2.98E-15 |  |  |  |  |
| *TMEM151A* | 3.566196198 | 5.21E-54 | 1.35E-52 |  |  |  |  |
| *TMEM45B* | 2.451121639 | 7.90E-61 | 2.52E-59 |  |  |  |  |
| *TMPRSS13* | -2.416615952 | 1.93E-24 | 1.69E-23 |  |  |  |  |
| *TMPRSS5* | 3.223111315 | 1.78E-55 | 4.83E-54 |  |  |  |  |
| *TNNT3* | -2.845578111 | 4.94E-27 | 4.87E-26 |  |  |  |  |
| *TP53AIP1* | -5.169814761 | 7.89E-104 | 6.31E-102 |  |  |  |  |
| *TREH* | 2.816669319 | 7.99E-51 | 1.89E-49 |  |  |  |  |
| *TRIM29* | -2.777095027 | 3.86E-34 | 5.20E-33 |  |  |  |  |
| *TRIM51P4.1* | -2.358718713 | 0.000341944 | 0.000717144 |  |  |  |  |
| *TRNA_Ser.1* | 2.268501874 | 1.22E-49 | 2.76E-48 |  |  |  |  |
| *TRPM5* | 3.541823545 | 2.37E-57 | 6.78E-56 |  |  |  |  |
| *USH1C* | 6.418904272 | 0 | 0 |  |  |  |  |
| *VSIG2* | 5.16597146 | 6.29E-122 | 7.27E-120 |  |  |  |  |
| *A2ML1* | -2.465819611 | 2.81E-10 | 1.04E-09 |  |  |  |  |
| *ADAMTS20* | -3.024874185 | 1.87E-18 | 1.18E-17 |  |  |  |  |
| *AK056228* | -2.987411765 | 4.39E-28 | 4.57E-27 |  |  |  |  |
| *AK095365* | -2.025224226 | 2.11E-18 | 1.32E-17 |  |  |  |  |
| *AK096314* | -2.008942947 | 3.89E-34 | 5.23E-33 |  |  |  |  |
| *AK124066* | 2.170123868 | 7.69E-19 | 4.96E-18 |  |  |  |  |
| *ALX1* | -2.385442964 | 4.72E-08 | 1.46E-07 |  |  |  |  |
| *AMHR2* | 2.033507436 | 3.43E-17 | 2.01E-16 |  |  |  |  |
| *ANKRD33* | 2.722024574 | 2.15E-17 | 1.28E-16 |  |  |  |  |
| *ANP32D* | -4.114658613 | 2.20E-23 | 1.82E-22 |  |  |  |  |
| *APOBEC1* | 6.24393919 | 6.10E-129 | 8.30E-127 |  |  |  |  |
| *AQP2* | 6.498481098 | 2.08E-85 | 1.14E-83 |  |  |  |  |
| *AQP5* | 3.712969259 | 2.40E-39 | 3.88E-38 |  |  |  |  |
| *ART4* | 2.545273471 | 1.69E-24 | 1.49E-23 |  |  |  |  |
| *ASCL4* | -3.04635043 | 1.51E-20 | 1.08E-19 |  |  |  |  |
| *AX746535* | -5.396813535 | 2.17E-89 | 1.26E-87 |  |  |  |  |
| *AX747752* | 2.069375961 | 8.54E-08 | 2.57E-07 |  |  |  |  |
| *AX748157* | 3.075285494 | 4.30E-17 | 2.51E-16 |  |  |  |  |
| *B4GALNT1* | -3.092864351 | 3.62E-90 | 2.17E-88 |  |  |  |  |
| *BC045559* | -2.88967064 | 1.46E-14 | 7.29E-14 |  |  |  |  |
| *BC047090* | -2.190250199 | 1.03E-10 | 3.94E-10 |  |  |  |  |
| *BCL2L14* | 3.72630994 | 7.84E-154 | 1.58E-151 |  |  |  |  |
| *BTBD11* | -2.862451388 | 9.96E-50 | 2.27E-48 |  |  |  |  |
| *C12orf28* | 2.328618085 | 1.21E-26 | 1.18E-25 |  |  |  |  |
| *C12orf36* | 2.203199296 | 4.88E-29 | 5.31E-28 |  |  |  |  |
| *C12orf37* | -2.457361365 | 4.57E-09 | 1.54E-08 |  |  |  |  |
| *C12orf54* | -4.785481992 | 3.00E-60 | 9.40E-59 |  |  |  |  |
| *C12orf56* | -2.299491142 | 6.69E-16 | 3.63E-15 |  |  |  |  |
| *CCDC38* | -2.46416226 | 2.27E-46 | 4.63E-45 |  |  |  |  |
| *CLEC12B* | -2.561054346 | 1.33E-32 | 1.69E-31 |  |  |  |  |
| *CNTN1* | -2.394644946 | 9.08E-21 | 6.54E-20 |  |  |  |  |
| *DNAJC22* | 3.839191576 | 3.60E-224 | 1.90E-221 |  |  |  |  |
| *DPPA3.1* | 2.726022921 | 1.73E-09 | 6.02E-09 |  |  |  |  |
| *DQ590166* | -2.053225527 | 1.45E-20 | 1.03E-19 |  |  |  |  |
| *ENDOU* | -2.200272235 | 2.00E-12 | 8.70E-12 |  |  |  |  |
| *FAIM2* | 2.333969327 | 6.36E-37 | 9.52E-36 |  |  |  |  |
| *FAM101A* | 3.610498802 | 5.80E-121 | 6.51E-119 |  |  |  |  |
| *FAR2* | 2.015519721 | 1.59E-59 | 4.84E-58 |  |  |  |  |
| *FLJ31485* | -2.315680776 | 6.81E-37 | 1.02E-35 |  |  |  |  |
| *FLJ37505* | -2.229853557 | 1.47E-06 | 3.96E-06 |  |  |  |  |
| *FZD10* | -3.168241661 | 7.22E-49 | 1.60E-47 |  |  |  |  |
| *GALNT8* | 4.026642541 | 4.03E-54 | 1.05E-52 |  |  |  |  |
| *GALNT9* | 2.812767755 | 1.17E-29 | 1.30E-28 |  |  |  |  |
| *GLTP* | -2.253131658 | 1.77E-100 | 1.30E-98 |  |  |  |  |
| *GPD1* | 5.37271727 | 4.10E-159 | 8.94E-157 |  |  |  |  |
| *GPRC5A* | 2.482819789 | 1.01E-62 | 3.36E-61 |  |  |  |  |
| *GRIN2B* | 2.440858947 | 9.85E-19 | 6.31E-18 |  |  |  |  |
| *GUCY2C* | 4.773561361 | 9.04E-114 | 8.85E-112 |  |  |  |  |
| *HCAR2* | -2.845319292 | 2.41E-39 | 3.89E-38 |  |  |  |  |
| *HCAR3* | -2.005707121 | 6.15E-20 | 4.25E-19 |  |  |  |  |
| *HNF1A-AS1* | 7.156651649 | 0 | 0 |  |  |  |  |
| *HNF1A* | 4.746189963 | 0 | 0 |  |  |  |  |
| *HRK* | -2.2109999 | 1.62E-16 | 9.11E-16 |  |  |  |  |
| *JA682662* | 2.145199405 | 0.000781253 | 0.001569183 |  |  |  |  |
| *KCNA1* | 2.528763125 | 1.87E-12 | 8.16E-12 |  |  |  |  |
| *KLRB1* | 2.003981742 | 9.34E-44 | 1.75E-42 |  |  |  |  |
| *KLRF2* | -2.850275 | 2.50E-19 | 1.67E-18 |  |  |  |  |
| *KRT18* | 2.357264148 | 4.77E-98 | 3.35E-96 |  |  |  |  |
| *KRT1* | -6.20908037 | 3.60E-76 | 1.62E-74 |  |  |  |  |
| *KRT3* | -3.647010163 | 1.98E-30 | 2.29E-29 |  |  |  |  |
| *KRT4* | -2.00902982 | 1.79E-05 | 4.32E-05 |  |  |  |  |
| *KRT5* | -4.908398232 | 1.52E-35 | 2.14E-34 |  |  |  |  |
| *KRT6A* | -3.926015138 | 3.55E-22 | 2.74E-21 |  |  |  |  |
| *KRT6B* | -3.996816996 | 3.55E-30 | 4.05E-29 |  |  |  |  |
| *KRT6C* | -3.441564422 | 1.13E-14 | 5.71E-14 |  |  |  |  |
| *KRT71* | -2.055627851 | 2.29E-12 | 9.93E-12 |  |  |  |  |
| *KRT74* | -4.652372445 | 6.90E-37 | 1.03E-35 |  |  |  |  |
| *KRT75* | -5.37599553 | 8.60E-31 | 1.01E-29 |  |  |  |  |
| *KRT76* | -4.121938827 | 7.88E-21 | 5.70E-20 |  |  |  |  |
| *KRT77* | -5.562268265 | 7.29E-44 | 1.38E-42 |  |  |  |  |
| *KRT79* | -5.381048287 | 7.63E-77 | 3.48E-75 |  |  |  |  |
| *KRT82* | -3.772325429 | 1.78E-18 | 1.12E-17 |  |  |  |  |
| *KRT84* | -3.339974879 | 7.08E-26 | 6.63E-25 |  |  |  |  |
| *KRT8* | 2.536821256 | 4.49E-95 | 2.98E-93 |  |  |  |  |
| *LGR5* | 2.3644459 | 2.08E-25 | 1.91E-24 |  |  |  |  |
| *LHX5* | -3.325851922 | 2.02E-21 | 1.51E-20 |  |  |  |  |
| *LINC00173* | -2.332022339 | 7.15E-47 | 1.48E-45 |  |  |  |  |
| *LOC100131138* | -2.637067914 | 6.71E-08 | 2.04E-07 |  |  |  |  |
| *LOC100190940* | 5.208699799 | 7.67E-45 | 1.50E-43 |  |  |  |  |
| *LOC100271702* | 2.248323349 | 4.08E-13 | 1.86E-12 |  |  |  |  |
| *LOC100292680* | -5.618648475 | 1.82E-140 | 2.96E-138 |  |  |  |  |
| *LOC255411* | 4.22637343 | 9.71E-55 | 2.57E-53 |  |  |  |  |
| *LOC255480* | -2.467741961 | 1.07E-16 | 6.10E-16 |  |  |  |  |
| *LOC400043* | 2.346301492 | 1.77E-39 | 2.88E-38 |  |  |  |  |
| *LYZ* | 4.379374697 | 9.94E-115 | 9.94E-113 |  |  |  |  |
| *METTL7B* | 3.882664907 | 5.82E-162 | 1.33E-159 |  |  |  |  |
| *MIR4497* | -2.337333408 | 9.29E-36 | 1.32E-34 |  |  |  |  |
| *MIR614* | 2.720333003 | 6.52E-74 | 2.78E-72 |  |  |  |  |
| *MUCL1* | -4.181484065 | 4.70E-36 | 6.76E-35 |  |  |  |  |
| *MYBPC1* | 2.097734365 | 2.34E-10 | 8.74E-10 |  |  |  |  |
| *MYO1A* | 7.625727837 | 0 | 0 |  |  |  |  |
| *Metazoa_SRP.19* | -2.154070398 | 3.01E-12 | 1.29E-11 |  |  |  |  |
| *NDUFA4L2* | -2.934968105 | 4.46E-78 | 2.10E-76 |  |  |  |  |
| *NELL2* | -3.962762909 | 5.70E-103 | 4.46E-101 |  |  |  |  |
| *NFE2* | 2.044591776 | 1.29E-22 | 1.02E-21 |  |  |  |  |
| *NOS1* | -4.04811557 | 1.04E-52 | 2.59E-51 |  |  |  |  |
| *NR1H4* | 5.433856556 | 2.51E-78 | 1.19E-76 |  |  |  |  |
| *NTS* | -4.806843101 | 1.62E-44 | 3.15E-43 |  |  |  |  |
| *NXPH4* | -2.613559778 | 1.56E-35 | 2.20E-34 |  |  |  |  |
| *OR6C2* | -2.328390215 | 1.95E-05 | 4.69E-05 |  |  |  |  |
| *OR6C70* | -3.313689628 | 8.63E-07 | 2.38E-06 |  |  |  |  |
| *OR9K2* | -2.408636814 | 0.000345984 | 0.00072545 |  |  |  |  |
| *PAH* | 2.540502352 | 4.16E-15 | 2.15E-14 |  |  |  |  |
| *PIWIL1* | 4.324945783 | 2.60E-45 | 5.16E-44 |  |  |  |  |
| *PLA2G1B* | 3.02318916 | 2.43E-28 | 2.56E-27 |  |  |  |  |
| *PPP1R1A* | 3.571037548 | 7.11E-40 | 1.17E-38 |  |  |  |  |
| *PRMT8* | -2.291578465 | 2.66E-12 | 1.15E-11 |  |  |  |  |
| *PTHLH* | -5.294006919 | 1.07E-174 | 2.92E-172 |  |  |  |  |
| *PTPRQ* | -2.74404543 | 6.52E-25 | 5.84E-24 |  |  |  |  |
| *PTPRR* | 3.077499064 | 5.88E-64 | 1.99E-62 |  |  |  |  |
| *RASSF9* | -2.109997239 | 7.98E-41 | 1.36E-39 |  |  |  |  |
| *RDH16* | -2.080271678 | 2.78E-30 | 3.18E-29 |  |  |  |  |
| *RND1* | 2.514420936 | 2.19E-68 | 8.23E-67 |  |  |  |  |
| *SDR9C7* | -4.143432122 | 5.91E-22 | 4.53E-21 |  |  |  |  |
| *SLC17A8* | 2.244689601 | 2.26E-11 | 9.09E-11 |  |  |  |  |
| *SLC26A10* | -2.948483039 | 8.32E-89 | 4.83E-87 |  |  |  |  |
| *SLC39A5* | 6.207593379 | 2.89E-211 | 1.32E-208 |  |  |  |  |
| *SLC41A2* | 2.612187064 | 2.57E-129 | 3.54E-127 |  |  |  |  |
| *SLC6A15* | -4.574660647 | 8.47E-43 | 1.54E-41 |  |  |  |  |
| *SLCO1A2* | -2.945312935 | 3.56E-22 | 2.75E-21 |  |  |  |  |
| *SOAT2* | 2.432474871 | 6.46E-17 | 3.74E-16 |  |  |  |  |
| *SVOP* | 2.509823794 | 4.06E-19 | 2.67E-18 |  |  |  |  |
| *TAC3* | 4.64017208 | 3.49E-100 | 2.54E-98 |  |  |  |  |
| *TBX5* | -2.253984884 | 4.20E-16 | 2.31E-15 |  |  |  |  |
| *TESC* | 6.026276045 | 6.18E-260 | 4.59E-257 |  |  |  |  |
| *TMEM132D* | 3.396413401 | 2.04E-37 | 3.09E-36 |  |  |  |  |
| *TRHDE* | 2.274320356 | 2.26E-22 | 1.77E-21 |  |  |  |  |
| *TRPV4* | -2.296497874 | 2.80E-56 | 7.78E-55 |  |  |  |  |
| *TSPAN8* | 8.253670078 | 0 | 0 |  |  |  |  |
| *WDR66* | -3.571224691 | 8.02E-126 | 1.03E-123 |  |  |  |  |
| *ZNF385A* | -2.30465234 | 5.09E-107 | 4.29E-105 |  |  |  |  |
| *AK056689* | 2.329815299 | 4.45E-15 | 2.30E-14 |  |  |  |  |
| *ANKRD26P3* | -3.099210702 | 2.30E-25 | 2.11E-24 |  |  |  |  |
| *ATP12A* | -3.712255844 | 4.31E-25 | 3.90E-24 |  |  |  |  |
| *ATP4B* | 5.793821228 | 2.85E-61 | 9.18E-60 |  |  |  |  |
| *ATP7B* | 2.918509035 | 1.19E-108 | 1.03E-106 |  |  |  |  |
| *AX747962* | 2.215802952 | 0.000239952 | 0.000512187 |  |  |  |  |
| *BASP1P1* | -2.664730513 | 3.26E-09 | 1.11E-08 |  |  |  |  |
| *BC025370* | 3.760181345 | 2.54E-25 | 2.32E-24 |  |  |  |  |
| *BC038727* | 2.147915104 | 2.83E-07 | 8.15E-07 |  |  |  |  |
| *CCDC70.1* | 2.031673311 | 9.35E-11 | 3.60E-10 |  |  |  |  |
| *CCDC70.2* | 2.080735612 | 2.69E-09 | 9.24E-09 |  |  |  |  |
| *CCNA1* | -6.420199204 | 3.02E-148 | 5.53E-146 |  |  |  |  |
| *CDX2* | 4.918600659 | 1.52E-107 | 1.29E-105 |  |  |  |  |
| *DACH1* | 2.310138692 | 1.93E-32 | 2.43E-31 |  |  |  |  |
| *F10* | 3.031869625 | 1.09E-55 | 2.98E-54 |  |  |  |  |
| *F7* | 3.154263501 | 7.49E-35 | 1.03E-33 |  |  |  |  |
| *FAM155A* | 2.171806432 | 3.10E-39 | 4.99E-38 |  |  |  |  |
| *FAM216B* | 3.22729282 | 3.77E-44 | 7.20E-43 |  |  |  |  |
| *GJA3* | -2.868795545 | 1.28E-39 | 2.10E-38 |  |  |  |  |
| *GJB2* | -3.16611068 | 1.29E-73 | 5.45E-72 |  |  |  |  |
| *GJB6* | -4.701644559 | 2.37E-46 | 4.82E-45 |  |  |  |  |
| *GSX1* | 3.252858985 | 2.47E-05 | 5.89E-05 |  |  |  |  |
| *HTR2A* | 2.046782667 | 1.26E-25 | 1.16E-24 |  |  |  |  |
| *MIR3665* | 2.141929773 | 1.57E-24 | 1.38E-23 |  |  |  |  |
| *NEK5* | 2.560220488 | 1.64E-56 | 4.58E-55 |  |  |  |  |
| *OLFM4* | 8.01068905 | 1.93E-144 | 3.31E-142 |  |  |  |  |
| *PDX1* | 5.647269586 | 1.82E-224 | 1.02E-221 |  |  |  |  |
| *PRHOXNB* | 3.8501188 | 6.48E-15 | 3.31E-14 |  |  |  |  |
| *RAB20* | 2.198155258 | 2.81E-168 | 7.08E-166 |  |  |  |  |
| *SLC10A2* | 5.163976876 | 1.78E-40 | 2.99E-39 |  |  |  |  |
| *SLITRK6* | -2.204195557 | 3.89E-20 | 2.70E-19 |  |  |  |  |
| *SOX1* | -4.73791929 | 7.24E-22 | 5.52E-21 |  |  |  |  |
| *TUBA3C* | 2.033615513 | 1.09E-05 | 2.70E-05 |  |  |  |  |
| *AHNAK2* | -2.277543532 | 2.66E-34 | 3.60E-33 |  |  |  |  |
| *AK093301* | 2.882737214 | 4.98E-09 | 1.67E-08 |  |  |  |  |
| *AMN* | 2.822710279 | 2.99E-79 | 1.44E-77 |  |  |  |  |
| *ANG* | 2.797413364 | 4.32E-126 | 5.55E-124 |  |  |  |  |
| *AY748447* | 2.789331545 | 6.67E-10 | 2.40E-09 |  |  |  |  |
| *BC029479* | 2.925117891 | 1.62E-29 | 1.80E-28 |  |  |  |  |
| *BC148240* | 2.133752438 | 0.009197909 | 0.015934504 |  |  |  |  |
| *BC148262* | -3.057912538 | 2.07E-13 | 9.65E-13 |  |  |  |  |
| *BMP4* | 2.216901655 | 2.42E-49 | 5.43E-48 |  |  |  |  |
| *BX161428* | 2.177885588 | 4.07E-19 | 2.67E-18 |  |  |  |  |
| *BX248253* | -2.243898447 | 4.97E-25 | 4.48E-24 |  |  |  |  |
| *C14orf105* | 5.799271195 | 1.59E-86 | 8.78E-85 |  |  |  |  |
| *C14orf162* | -3.426125233 | 1.14E-30 | 1.33E-29 |  |  |  |  |
| *C14orf176* | 2.336853802 | 4.72E-43 | 8.66E-42 |  |  |  |  |
| *C14orf180* | 4.597184575 | 1.56E-19 | 1.05E-18 |  |  |  |  |
| *C14orf184* | 2.74776374 | 2.23E-33 | 2.92E-32 |  |  |  |  |
| *CATSPERB* | 2.571879069 | 1.91E-44 | 3.70E-43 |  |  |  |  |
| *CHGA* | 5.581638387 | 1.62E-68 | 6.12E-67 |  |  |  |  |
| *CLMN* | 2.196656915 | 1.59E-127 | 2.11E-125 |  |  |  |  |
| *CPNE6* | 2.793235096 | 2.22E-29 | 2.45E-28 |  |  |  |  |
| *DEGS2* | 2.179336155 | 1.89E-42 | 3.37E-41 |  |  |  |  |
| *DKFZp686O16217* | 2.446010038 | 5.17E-25 | 4.66E-24 |  |  |  |  |
| *DLK1* | 2.547204126 | 1.11E-11 | 4.56E-11 |  |  |  |  |
| *EFS* | -3.321234378 | 2.49E-117 | 2.59E-115 |  |  |  |  |
| *FAM181A-AS1* | -2.733965691 | 4.94E-10 | 1.80E-09 |  |  |  |  |
| *FAM181A* | -4.041209988 | 4.55E-24 | 3.90E-23 |  |  |  |  |
| *FLJ43390* | 2.072649617 | 6.40E-14 | 3.08E-13 |  |  |  |  |
| *FOXG1* | -3.743941923 | 3.61E-24 | 3.11E-23 |  |  |  |  |
| *FRMD6-AS1* | -2.048766552 | 2.02E-52 | 5.00E-51 |  |  |  |  |
| *FRMD6* | -2.632206884 | 4.51E-90 | 2.69E-88 |  |  |  |  |
| *HSPA2* | -2.012895039 | 1.58E-41 | 2.76E-40 |  |  |  |  |
| *Ig_alpha_1-[alpha]2m* | 3.018969166 | 1.95E-30 | 2.25E-29 |  |  |  |  |
| *JA429831.2* | 2.13877653 | 1.18E-14 | 5.93E-14 |  |  |  |  |
| *LOC100505967* | -2.254043431 | 5.67E-08 | 1.74E-07 |  |  |  |  |
| *LOC283553* | -4.30573146 | 1.08E-89 | 6.35E-88 |  |  |  |  |
| *LOC90925* | 2.826940344 | 6.51E-41 | 1.11E-39 |  |  |  |  |
| *LTB4R2* | -3.088327088 | 3.49E-146 | 6.16E-144 |  |  |  |  |
| *LTB4R* | -3.258076798 | 3.00E-145 | 5.25E-143 |  |  |  |  |
| *MIA2* | 5.831497105 | 3.86E-176 | 1.09E-173 |  |  |  |  |
| *MIR1260A* | -3.892831236 | 1.37E-21 | 1.03E-20 |  |  |  |  |
| *MIR127* | 2.205620247 | 0.001301866 | 0.002546948 |  |  |  |  |
| *MIR136* | 3.366755649 | 1.50E-05 | 3.66E-05 |  |  |  |  |
| *MIR203* | -2.651892335 | 2.44E-11 | 9.80E-11 |  |  |  |  |
| *MIR3545* | -2.503744626 | 4.77E-09 | 1.61E-08 |  |  |  |  |
| *MIR431* | 2.648633636 | 0.000115384 | 0.000254736 |  |  |  |  |
| *MIR432* | 3.056357195 | 8.33E-05 | 0.000187116 |  |  |  |  |
| *NGB* | -3.157030581 | 8.07E-14 | 3.86E-13 |  |  |  |  |
| *NKX2-8* | -2.939577775 | 6.81E-15 | 3.48E-14 |  |  |  |  |
| *OR11H4* | -3.079168761 | 9.70E-18 | 5.89E-17 |  |  |  |  |
| *OR11H6* | -2.617472053 | 6.28E-10 | 2.27E-09 |  |  |  |  |
| *PAX9* | -2.799539339 | 2.99E-32 | 3.73E-31 |  |  |  |  |
| *PPP1R36* | 2.596152683 | 1.65E-77 | 7.61E-76 |  |  |  |  |
| *PPP4R4* | -2.535236473 | 1.86E-31 | 2.24E-30 |  |  |  |  |
| *PTGER2* | 2.812579734 | 9.24E-71 | 3.73E-69 |  |  |  |  |
| *PYGL* | -2.429902534 | 3.17E-58 | 9.21E-57 |  |  |  |  |
| *RNASE10* | -4.876451917 | 1.07E-87 | 6.04E-86 |  |  |  |  |
| *RNASE4* | 2.69782624 | 2.12E-125 | 2.67E-123 |  |  |  |  |
| *RNASE7* | -3.514401571 | 6.26E-22 | 4.79E-21 |  |  |  |  |
| *RTL1* | 2.27203758 | 7.61E-14 | 3.64E-13 |  |  |  |  |
| *SERPINA10* | 2.525613663 | 4.45E-13 | 2.02E-12 |  |  |  |  |
| *SERPINA1* | 4.690987672 | 4.35E-127 | 5.72E-125 |  |  |  |  |
| *SERPINA4* | 7.346326107 | 1.24E-177 | 3.59E-175 |  |  |  |  |
| *SERPINA5* | 4.133298367 | 1.39E-75 | 6.12E-74 |  |  |  |  |
| *SERPINA6* | 3.501869917 | 1.04E-27 | 1.07E-26 |  |  |  |  |
| *SIX6* | -2.104096729 | 0.000305717 | 0.000644448 |  |  |  |  |
| *SLC25A21* | -2.355852879 | 9.62E-43 | 1.74E-41 |  |  |  |  |
| *SLC39A2* | -3.043629193 | 1.35E-18 | 8.53E-18 |  |  |  |  |
| *SLC7A8* | -2.646462735 | 2.98E-92 | 1.87E-90 |  |  |  |  |
| *SSTR1* | 6.140289373 | 1.16E-188 | 4.04E-186 |  |  |  |  |
| *STON2* | -2.713460502 | 3.86E-126 | 4.99E-124 |  |  |  |  |
| *TGM1* | -2.706052481 | 6.80E-17 | 3.93E-16 |  |  |  |  |
| *TMEM63C* | -2.758087953 | 5.58E-31 | 6.62E-30 |  |  |  |  |
| *TRNA_Pseudo.36* | 2.308679428 | 1.74E-07 | 5.11E-07 |  |  |  |  |
| *TRNA_Tyr.3* | 2.830685558 | 2.20E-14 | 1.08E-13 |  |  |  |  |
| *TRNA_Tyr.4* | 3.122065983 | 1.41E-17 | 8.52E-17 |  |  |  |  |
| *TRNA_Tyr.5* | 2.202764237 | 4.95E-08 | 1.52E-07 |  |  |  |  |
| *U6.40* | -3.994273644 | 5.86E-24 | 4.99E-23 |  |  |  |  |
| *U6.41* | -3.163156521 | 6.43E-32 | 7.95E-31 |  |  |  |  |
| *VRTN* | 4.484034737 | 6.77E-63 | 2.25E-61 |  |  |  |  |
| *immunoglobulin_heavy_chain* | 2.156752445 | 2.92E-20 | 2.04E-19 |  |  |  |  |
| *tRNA_Pro.6* | 2.319133103 | 4.37E-11 | 1.72E-10 |  |  |  |  |
| *AJ004954* | 2.211844957 | 3.91E-25 | 3.54E-24 |  |  |  |  |
| *AK056686* | 2.414267806 | 7.58E-58 | 2.18E-56 |  |  |  |  |
| *AK124939* | 2.262885488 | 9.51E-12 | 3.93E-11 |  |  |  |  |
| *AK302238* | -3.157837668 | 1.67E-22 | 1.32E-21 |  |  |  |  |
| *ANPEP* | 4.420397367 | 9.67E-70 | 3.75E-68 |  |  |  |  |
| *AX748237* | -2.179837447 | 4.34E-15 | 2.24E-14 |  |  |  |  |
| *BC024169* | 2.24829169 | 8.93E-05 | 0.000199895 |  |  |  |  |
| *BC037952* | -2.145148595 | 5.54E-08 | 1.70E-07 |  |  |  |  |
| *BC043570* | 3.797966044 | 5.55E-107 | 4.66E-105 |  |  |  |  |
| *BNC1* | -5.816589611 | 2.54E-77 | 1.17E-75 |  |  |  |  |
| *C2CD4A* | 2.187419596 | 7.19E-30 | 8.08E-29 |  |  |  |  |
| *C2CD4B* | 3.085856679 | 4.39E-69 | 1.69E-67 |  |  |  |  |
| *CA12* | -2.635652774 | 1.31E-51 | 3.15E-50 |  |  |  |  |
| *CALML4* | 2.122001693 | 1.25E-117 | 1.32E-115 |  |  |  |  |
| *CERS3* | -4.136048185 | 2.43E-30 | 2.79E-29 |  |  |  |  |
| *CHRNA7* | 2.164411542 | 7.46E-29 | 8.03E-28 |  |  |  |  |
| *CHRNB4* | -2.50659095 | 1.52E-40 | 2.57E-39 |  |  |  |  |
| *CRABP1* | 2.04327233 | 3.42E-10 | 1.26E-09 |  |  |  |  |
| *DQ571370* | 2.030458295 | 0.016736332 | 0.027786091 |  |  |  |  |
| *DQ571691* | 2.144418607 | 0.010434841 | 0.017909235 |  |  |  |  |
| *DQ573543* | 2.031548 | 0.01240687 | 0.02103125 |  |  |  |  |
| *DQ573567* | 2.120410571 | 0.003572204 | 0.006589141 |  |  |  |  |
| *DQ574028* | 2.194897242 | 0.000119134 | 0.000262615 |  |  |  |  |
| *DQ576354* | 2.033724998 | 0.003263141 | 0.006061757 |  |  |  |  |
| *DQ577256* | 2.182586606 | 0.004083142 | 0.007469461 |  |  |  |  |
| *DQ580408* | 2.450139386 | 0.007540234 | 0.013259064 |  |  |  |  |
| *DQ580467* | 2.367350722 | 4.72E-05 | 0.000108914 |  |  |  |  |
| *DQ581622* | 2.653863792 | 0.000198123 | 0.000426946 |  |  |  |  |
| *DQ590438* | 2.060306447 | 0.017948738 | 0.029616549 |  |  |  |  |
| *DQ593269* | 2.130065883 | 0.001774887 | 0.003410477 |  |  |  |  |
| *DQ593762* | 2.269859423 | 5.71E-05 | 0.000130696 |  |  |  |  |
| *DQ597549* | 2.134238915 | 0.004412422 | 0.008047197 |  |  |  |  |
| *DQ600540* | 2.042491759 | 0.003359282 | 0.00622764 |  |  |  |  |
| *DQ600614* | 2.36043999 | 0.00379246 | 0.006968768 |  |  |  |  |
| *DQ600930* | 2.094974875 | 0.000260084 | 0.000552799 |  |  |  |  |
| *DUOX1* | -2.151474952 | 5.88E-26 | 5.53E-25 |  |  |  |  |
| *FAM169B* | 3.83838534 | 1.53E-40 | 2.59E-39 |  |  |  |  |
| *FAM81A* | 2.042337119 | 1.94E-54 | 5.12E-53 |  |  |  |  |
| *FRMD5* | 2.611516173 | 2.53E-81 | 1.28E-79 |  |  |  |  |
| *GABRA5* | -2.193753108 | 1.72E-10 | 6.51E-10 |  |  |  |  |
| *GABRG3* | -2.41384391 | 5.25E-09 | 1.76E-08 |  |  |  |  |
| *GATM* | 2.120966831 | 1.00E-42 | 1.82E-41 |  |  |  |  |
| *GCNT3* | 4.264315091 | 2.66E-109 | 2.32E-107 |  |  |  |  |
| *GOLGA6L1.1* | -2.238239417 | 3.27E-12 | 1.40E-11 |  |  |  |  |
| *HCN4* | 3.553747639 | 2.05E-39 | 3.34E-38 |  |  |  |  |
| *HP11097* | -2.30681314 | 2.23E-23 | 1.84E-22 |  |  |  |  |
| *LIPC* | 3.16435366 | 7.20E-44 | 1.36E-42 |  |  |  |  |
| *LOC145837* | 2.605436422 | 1.90E-29 | 2.09E-28 |  |  |  |  |
| *LOC254559* | -2.698048694 | 2.45E-54 | 6.41E-53 |  |  |  |  |
| *LOC283710* | 2.169295319 | 5.19E-41 | 8.93E-40 |  |  |  |  |
| *LOC283761* | -2.334629234 | 1.06E-13 | 5.03E-13 |  |  |  |  |
| *LTK* | 2.195761566 | 2.99E-31 | 3.59E-30 |  |  |  |  |
| *MKRN3* | -2.16511842 | 5.98E-15 | 3.06E-14 |  |  |  |  |
| *NBEAP1* | -3.354537302 | 3.00E-36 | 4.35E-35 |  |  |  |  |
| *ONECUT1* | 2.759266441 | 2.07E-15 | 1.09E-14 |  |  |  |  |
| *OSTBETA* | 2.859298335 | 1.28E-48 | 2.81E-47 |  |  |  |  |
| *PHGR1* | 10.3045961 | 0 | 0 |  |  |  |  |
| *PLA2G4D* | -2.654428467 | 1.11E-33 | 1.48E-32 |  |  |  |  |
| *PLA2G4E* | -4.219433922 | 6.42E-55 | 1.70E-53 |  |  |  |  |
| *PLIN1* | 2.002383314 | 2.45E-24 | 2.14E-23 |  |  |  |  |
| *PPP1R14D* | 5.937718186 | 3.72E-204 | 1.53E-201 |  |  |  |  |
| *REREP3* | -2.66938578 | 2.63E-27 | 2.63E-26 |  |  |  |  |
| *RHCG* | -2.090951059 | 1.63E-07 | 4.79E-07 |  |  |  |  |
| *RHOV* | -2.712785382 | 5.87E-43 | 1.07E-41 |  |  |  |  |
| *SCG3* | 3.311323717 | 1.47E-39 | 2.41E-38 |  |  |  |  |
| *SH3GL3* | -3.746335547 | 7.14E-36 | 1.02E-34 |  |  |  |  |
| *SLC28A2* | 7.155872013 | 2.43E-110 | 2.16E-108 |  |  |  |  |
| *SMAD6* | 2.127467637 | 1.22E-117 | 1.29E-115 |  |  |  |  |
| *TGM5* | -3.060769811 | 1.28E-16 | 7.27E-16 |  |  |  |  |
| *TRPM1.2* | -2.810101782 | 1.44E-23 | 1.20E-22 |  |  |  |  |
| *abParts.2* | 2.66181679 | 3.29E-22 | 2.55E-21 |  |  |  |  |
| *ABCC1* | -2.059413817 | 1.09E-136 | 1.69E-134 |  |  |  |  |
| *ABCC6P1* | 5.053980284 | 1.01E-125 | 1.28E-123 |  |  |  |  |
| *ABCC6P2* | 3.18302356 | 4.16E-63 | 1.39E-61 |  |  |  |  |
| *ABCC6* | 3.211855066 | 1.97E-106 | 1.64E-104 |  |  |  |  |
| *ACSM2B* | 2.075815152 | 3.56E-07 | 1.01E-06 |  |  |  |  |
| *ACSM3* | 2.14526358 | 1.14E-61 | 3.69E-60 |  |  |  |  |
| *ADAD2* | -3.48760158 | 1.50E-52 | 3.71E-51 |  |  |  |  |
| *AF086125* | 2.06834441 | 1.55E-17 | 9.29E-17 |  |  |  |  |
| *AK055272* | 2.208241989 | 3.18E-26 | 3.04E-25 |  |  |  |  |
| *AK123582* | -4.371996571 | 5.42E-55 | 1.45E-53 |  |  |  |  |
| *ANKS4B* | 6.426397494 | 1.04E-269 | 8.63E-267 |  |  |  |  |
| *ARHGDIG* | 3.472002492 | 3.15E-45 | 6.21E-44 |  |  |  |  |
| *ASPHD1* | 2.7360977 | 5.00E-68 | 1.86E-66 |  |  |  |  |
| *BC041439* | -2.216860981 | 3.09E-05 | 7.27E-05 |  |  |  |  |
| *BCAR4* | 2.228260385 | 3.14E-08 | 9.84E-08 |  |  |  |  |
| *C16orf74* | -2.969428311 | 1.80E-70 | 7.19E-69 |  |  |  |  |
| *C16orf89* | 3.587987477 | 5.22E-53 | 1.31E-51 |  |  |  |  |
| *CAPNS2* | -4.335154756 | 2.46E-33 | 3.21E-32 |  |  |  |  |
| *CBLN1* | 2.639299594 | 3.65E-20 | 2.54E-19 |  |  |  |  |
| *CCDC135* | 2.048758038 | 2.48E-18 | 1.54E-17 |  |  |  |  |
| *CDH3* | -2.692814322 | 1.31E-71 | 5.40E-70 |  |  |  |  |
| *CDH8* | -3.360899684 | 1.88E-75 | 8.26E-74 |  |  |  |  |
| *CES1P1* | -3.488794204 | 3.97E-20 | 2.76E-19 |  |  |  |  |
| *CES1P2* | -3.849603487 | 3.88E-21 | 2.85E-20 |  |  |  |  |
| *CES1* | -3.888433839 | 1.31E-48 | 2.86E-47 |  |  |  |  |
| *CHST4* | 3.170344975 | 1.79E-34 | 2.44E-33 |  |  |  |  |
| *CHST5* | 4.334484575 | 2.00E-90 | 1.20E-88 |  |  |  |  |
| *CLDN6* | 4.545129993 | 3.68E-39 | 5.92E-38 |  |  |  |  |
| *CLDN9* | 2.557503728 | 2.78E-29 | 3.05E-28 |  |  |  |  |
| *CLEC19A* | 2.08743058 | 2.93E-12 | 1.26E-11 |  |  |  |  |
| *CNGB1* | -5.568799827 | 4.68E-130 | 6.50E-128 |  |  |  |  |
| *CTRB1* | 3.897508325 | 4.24E-16 | 2.33E-15 |  |  |  |  |
| *CTRB2* | 2.970788069 | 1.84E-10 | 6.94E-10 |  |  |  |  |
| *DPEP1* | 6.811588552 | 2.10E-127 | 2.78E-125 |  |  |  |  |
| *DQ573285* | -4.312245443 | 3.24E-18 | 2.01E-17 |  |  |  |  |
| *ERN2* | 4.955532332 | 5.33E-179 | 1.61E-176 |  |  |  |  |
| *FA2H* | 3.439316555 | 7.90E-134 | 1.17E-131 |  |  |  |  |
| *FLJ26245* | -2.013515651 | 0.000985773 | 0.001956561 |  |  |  |  |
| *FOXC2* | -2.206373594 | 3.66E-36 | 5.28E-35 |  |  |  |  |
| *GP2* | 7.636693024 | 4.51E-124 | 5.48E-122 |  |  |  |  |
| *GPRC5B* | 2.375500108 | 7.98E-50 | 1.82E-48 |  |  |  |  |
| *HAS3* | -2.761172499 | 1.77E-114 | 1.76E-112 |  |  |  |  |
| *HP* | 2.466202651 | 6.03E-22 | 4.62E-21 |  |  |  |  |
| *HS3ST6* | -2.424049925 | 1.37E-11 | 5.61E-11 |  |  |  |  |
| *HSD17B2* | 3.645363467 | 5.61E-79 | 2.70E-77 |  |  |  |  |
| *IGFALS* | 4.217410099 | 3.45E-60 | 1.08E-58 |  |  |  |  |
| *IGH.2* | 2.140307179 | 9.42E-12 | 3.90E-11 |  |  |  |  |
| *IGH.5* | 3.253039017 | 1.81E-10 | 6.81E-10 |  |  |  |  |
| *IGH.7* | 2.136462999 | 3.13E-08 | 9.83E-08 |  |  |  |  |
| *IGH.8* | 3.315766393 | 2.28E-08 | 7.27E-08 |  |  |  |  |
| *IGH.9* | 2.134553025 | 0.000275272 | 0.000583502 |  |  |  |  |
| *IL17C* | 2.83847691 | 8.54E-30 | 9.56E-29 |  |  |  |  |
| *IRF8* | 2.677621942 | 6.16E-107 | 5.16E-105 |  |  |  |  |
| *IRX3* | -2.957137015 | 9.94E-44 | 1.86E-42 |  |  |  |  |
| *IRX6* | -4.004778962 | 1.53E-30 | 1.78E-29 |  |  |  |  |
| *KREMEN2* | -3.231111205 | 6.17E-76 | 2.74E-74 |  |  |  |  |
| *LOC146336* | 3.076297274 | 2.95E-22 | 2.29E-21 |  |  |  |  |
| *LOC440335* | 2.891935283 | 4.81E-81 | 2.41E-79 |  |  |  |  |
| *LOC643714* | 3.697485843 | 7.99E-36 | 1.14E-34 |  |  |  |  |
| *LRRC36* | 2.524923941 | 3.32E-50 | 7.73E-49 |  |  |  |  |
| *MIR1910* | -2.266212384 | 4.49E-21 | 3.29E-20 |  |  |  |  |
| *MIR3182* | -2.053415813 | 2.89E-06 | 7.56E-06 |  |  |  |  |
| *MIR548AE2* | -2.535697561 | 3.44E-11 | 1.37E-10 |  |  |  |  |
| *MIR662* | 2.939795694 | 4.69E-13 | 2.13E-12 |  |  |  |  |
| *MSLNL* | 2.560030435 | 2.47E-20 | 1.74E-19 |  |  |  |  |
| *MSLN* | 3.906997865 | 1.90E-60 | 6.00E-59 |  |  |  |  |
| *MT1G* | 2.402070319 | 2.04E-30 | 2.35E-29 |  |  |  |  |
| *MT1H* | 2.757387162 | 4.19E-31 | 4.99E-30 |  |  |  |  |
| *NDRG4* | -3.666278862 | 6.74E-123 | 7.99E-121 |  |  |  |  |
| *NECAB2* | -3.243115347 | 8.15E-59 | 2.42E-57 |  |  |  |  |
| *NOD2* | -2.391576167 | 2.60E-72 | 1.08E-70 |  |  |  |  |
| *PDIA2* | 4.419916146 | 9.80E-72 | 4.06E-70 |  |  |  |  |
| *PDILT* | 3.803372026 | 2.11E-16 | 1.18E-15 |  |  |  |  |
| *PLA2G10* | 4.16532457 | 6.36E-129 | 8.57E-127 |  |  |  |  |
| *PRSS30P* | 3.056421559 | 9.22E-49 | 2.03E-47 |  |  |  |  |
| *PRSS33* | 5.559097216 | 2.10E-99 | 1.51E-97 |  |  |  |  |
| *PYDC1* | -2.031869399 | 1.81E-09 | 6.28E-09 |  |  |  |  |
| *QPRT* | 2.259972046 | 1.83E-29 | 2.02E-28 |  |  |  |  |
| *RPL3L* | -3.695629389 | 4.43E-50 | 1.03E-48 |  |  |  |  |
| *SCNN1G* | -2.457779658 | 7.00E-17 | 4.04E-16 |  |  |  |  |
| *SLC6A2* | -3.611424388 | 1.03E-31 | 1.26E-30 |  |  |  |  |
| *SLC7A5* | -2.139670885 | 5.52E-56 | 1.52E-54 |  |  |  |  |
| *SMPD3* | 3.397139806 | 5.72E-115 | 5.78E-113 |  |  |  |  |
| *SOX8* | 2.037064386 | 4.52E-23 | 3.69E-22 |  |  |  |  |
| *SULT1A2* | 2.263515671 | 3.39E-39 | 5.45E-38 |  |  |  |  |
| *TEPP* | -2.289012442 | 1.49E-21 | 1.12E-20 |  |  |  |  |
| *TMC5* | 5.361254512 | 3.65E-284 | 3.72E-281 |  |  |  |  |
| *TMED6* | 2.165128268 | 5.52E-45 | 1.08E-43 |  |  |  |  |
| *TNFRSF17* | 2.150615615 | 3.05E-28 | 3.20E-27 |  |  |  |  |
| *TOX3* | 5.007692045 | 1.03E-173 | 2.75E-171 |  |  |  |  |
| *TPSG1* | 2.376965313 | 1.92E-33 | 2.52E-32 |  |  |  |  |
| *TRIM72.1* | 3.009001519 | 1.16E-23 | 9.70E-23 |  |  |  |  |
| *UMOD* | 2.741738546 | 8.36E-13 | 3.73E-12 |  |  |  |  |
| *VAT1L* | 2.156468682 | 1.56E-49 | 3.53E-48 |  |  |  |  |
| *VHDJH* | 2.899281131 | 2.98E-21 | 2.20E-20 |  |  |  |  |
| *ZG16B* | 3.441697741 | 1.75E-58 | 5.13E-57 |  |  |  |  |
| *ZG16* | 5.00943364 | 3.93E-57 | 1.11E-55 |  |  |  |  |
| *tRNA_Pro.15* | -2.05419996 | 1.78E-07 | 5.21E-07 |  |  |  |  |
| *AATK* | 2.61276326 | 3.64E-74 | 1.55E-72 |  |  |  |  |
| *ABCA8* | 2.210968483 | 2.46E-23 | 2.02E-22 |  |  |  |  |
| *AK055254* | -2.294496862 | 3.00E-37 | 4.52E-36 |  |  |  |  |
| *AK127460* | -4.37836453 | 1.26E-117 | 1.33E-115 |  |  |  |  |
| *AK296148* | -3.312901648 | 4.96E-39 | 7.92E-38 |  |  |  |  |
| *AK301679* | -3.638094864 | 4.59E-19 | 3.00E-18 |  |  |  |  |
| *ALDH3A1* | -3.652109994 | 4.20E-58 | 1.21E-56 |  |  |  |  |
| *ALOX12B* | -3.35694977 | 1.67E-43 | 3.09E-42 |  |  |  |  |
| *ALOX12P2* | -3.000819964 | 7.08E-47 | 1.47E-45 |  |  |  |  |
| *ALOX12* | -2.706112676 | 6.10E-57 | 1.72E-55 |  |  |  |  |
| *ALOX15B* | -2.54853911 | 2.15E-36 | 3.13E-35 |  |  |  |  |
| *ALOXE3* | -3.498188359 | 3.62E-60 | 1.13E-58 |  |  |  |  |
| *APOH* | 5.030773899 | 1.48E-59 | 4.50E-58 |  |  |  |  |
| *ARL4D* | -2.7482652 | 1.51E-52 | 3.74E-51 |  |  |  |  |
| *ASGR1* | 2.143235997 | 2.87E-32 | 3.59E-31 |  |  |  |  |
| *ATP2A3* | 2.487009511 | 6.04E-76 | 2.69E-74 |  |  |  |  |
| *AX747630* | 3.464348605 | 7.89E-30 | 8.84E-29 |  |  |  |  |
| *AX748345* | 2.704393682 | 1.28E-92 | 8.04E-91 |  |  |  |  |
| *AXIN2* | 2.637811327 | 2.61E-45 | 5.16E-44 |  |  |  |  |
| *BC037347* | -2.665202059 | 1.07E-23 | 8.97E-23 |  |  |  |  |
| *BC040189* | -2.514085275 | 6.14E-11 | 2.39E-10 |  |  |  |  |
| *BC043554* | 5.261750803 | 3.23E-56 | 8.96E-55 |  |  |  |  |
| *BC044939* | 4.649032066 | 2.84E-160 | 6.41E-158 |  |  |  |  |
| *BC046191* | -2.661335841 | 4.18E-39 | 6.69E-38 |  |  |  |  |
| *C17orf102* | 2.193790444 | 4.27E-06 | 1.10E-05 |  |  |  |  |
| *C17orf110* | 5.50322927 | 1.43E-269 | 1.15E-266 |  |  |  |  |
| *C17orf28* | 3.47114329 | 2.80E-179 | 8.56E-177 |  |  |  |  |
| *C17orf78* | 2.403788953 | 1.09E-21 | 8.23E-21 |  |  |  |  |
| *CA4* | 5.912164667 | 9.72E-80 | 4.75E-78 |  |  |  |  |
| *CCL15* | 2.261275168 | 1.05E-36 | 1.55E-35 |  |  |  |  |
| *CCL23* | 2.405773595 | 1.28E-38 | 2.02E-37 |  |  |  |  |
| *CDK5R1* | -2.312775627 | 1.98E-76 | 8.98E-75 |  |  |  |  |
| *CORO6* | -2.85531231 | 1.77E-74 | 7.63E-73 |  |  |  |  |
| *DNAH17* | -4.284518694 | 0 | 0 |  |  |  |  |
| *ENPP7* | 3.157109135 | 3.78E-30 | 4.30E-29 |  |  |  |  |
| *EVPLL* | -2.214697615 | 2.28E-22 | 1.78E-21 |  |  |  |  |
| *FAM211A* | 2.807128867 | 8.16E-145 | 1.42E-142 |  |  |  |  |
| *FGF11* | -2.86452658 | 3.87E-78 | 1.82E-76 |  |  |  |  |
| *FOXJ1* | 2.486064668 | 6.66E-20 | 4.59E-19 |  |  |  |  |
| *FOXN1* | -4.608209452 | 7.09E-61 | 2.27E-59 |  |  |  |  |
| *GIP* | 2.369928204 | 1.50E-11 | 6.10E-11 |  |  |  |  |
| *GLP2R* | 2.540370164 | 1.00E-25 | 9.31E-25 |  |  |  |  |
| *GRAPL* | 2.105742454 | 4.15E-12 | 1.76E-11 |  |  |  |  |
| *GSDMB* | 3.220570924 | 1.99E-85 | 1.09E-83 |  |  |  |  |
| *HAP1* | -2.905105332 | 2.56E-35 | 3.58E-34 |  |  |  |  |
| *HCRT* | 2.734377644 | 6.59E-24 | 5.60E-23 |  |  |  |  |
| *HNF1B* | 6.658227552 | 6.03E-289 | 6.63E-286 |  |  |  |  |
| *HOXB-AS3* | 2.878927458 | 3.07E-96 | 2.10E-94 |  |  |  |  |
| *HOXB13* | 2.22741216 | 3.44E-23 | 2.82E-22 |  |  |  |  |
| *HOXB5* | 2.506848285 | 7.34E-83 | 3.87E-81 |  |  |  |  |
| *HOXB6* | 2.964083205 | 7.19E-90 | 4.26E-88 |  |  |  |  |
| *HOXB8* | 3.108847788 | 8.07E-41 | 1.38E-39 |  |  |  |  |
| *HOXB9* | 2.264526451 | 3.90E-31 | 4.66E-30 |  |  |  |  |
| *HS3ST3A1* | -2.136710715 | 1.09E-47 | 2.32E-46 |  |  |  |  |
| *IKZF3* | 2.09475796 | 1.04E-30 | 1.21E-29 |  |  |  |  |
| *KCNH6* | 3.778879884 | 1.17E-43 | 2.18E-42 |  |  |  |  |
| *KRT10* | -2.350846853 | 1.20E-56 | 3.37E-55 |  |  |  |  |
| *KRT13* | -2.132320623 | 1.55E-06 | 4.15E-06 |  |  |  |  |
| *KRT14* | -5.418143837 | 6.87E-40 | 1.14E-38 |  |  |  |  |
| *KRT15* | -4.201295231 | 3.95E-58 | 1.14E-56 |  |  |  |  |
| *KRT16P2* | -2.349811533 | 2.10E-11 | 8.47E-11 |  |  |  |  |
| *KRT16P3* | -3.464760829 | 4.25E-17 | 2.48E-16 |  |  |  |  |
| *KRT16* | -4.798070519 | 2.32E-47 | 4.88E-46 |  |  |  |  |
| *KRT17* | -4.668952126 | 1.42E-71 | 5.85E-70 |  |  |  |  |
| *KRT20* | 8.75815962 | 4.03E-163 | 9.38E-161 |  |  |  |  |
| *KRT24* | -2.502921079 | 9.49E-07 | 2.60E-06 |  |  |  |  |
| *KRT31* | -4.156287617 | 6.83E-17 | 3.95E-16 |  |  |  |  |
| *KRT32* | -2.625424066 | 2.50E-11 | 1.00E-10 |  |  |  |  |
| *KRT33A* | -2.389482494 | 1.30E-07 | 3.84E-07 |  |  |  |  |
| *KRT33B* | -2.103599097 | 6.30E-08 | 1.92E-07 |  |  |  |  |
| *KRT34* | -3.857440163 | 4.81E-27 | 4.75E-26 |  |  |  |  |
| *KRT35* | -2.135204681 | 3.17E-07 | 9.06E-07 |  |  |  |  |
| *KRT37* | -3.594871808 | 2.79E-16 | 1.55E-15 |  |  |  |  |
| *KRT38* | -2.184002466 | 3.34E-06 | 8.68E-06 |  |  |  |  |
| *KRT40* | 2.409439274 | 2.42E-12 | 1.05E-11 |  |  |  |  |
| *KRT42P* | -4.899209907 | 1.06E-95 | 7.11E-94 |  |  |  |  |
| *KRT9* | -3.096066088 | 2.20E-30 | 2.54E-29 |  |  |  |  |
| *KRTAP3-3* | 2.278433283 | 1.64E-05 | 3.97E-05 |  |  |  |  |
| *LINC00483* | 6.325510307 | 3.03E-204 | 1.26E-201 |  |  |  |  |
| *LOC100289255* | 5.014100261 | 1.22E-121 | 1.39E-119 |  |  |  |  |
| *LOC100505782* | -2.564113507 | 2.38E-11 | 9.57E-11 |  |  |  |  |
| *LOC339240* | -4.375320422 | 2.68E-59 | 8.07E-58 |  |  |  |  |
| *MARCH10* | -2.06011942 | 2.72E-19 | 1.81E-18 |  |  |  |  |
| *MFSD6L* | 2.508432299 | 3.89E-37 | 5.85E-36 |  |  |  |  |
| *MIR2117* | -4.986491855 | 2.27E-50 | 5.32E-49 |  |  |  |  |
| *MIR657* | 2.579748841 | 3.87E-20 | 2.69E-19 |  |  |  |  |
| *MYADML2* | 2.695287252 | 6.01E-32 | 7.45E-31 |  |  |  |  |
| *MYH4* | 2.607971093 | 1.77E-15 | 9.38E-15 |  |  |  |  |
| *MYO15B* | 2.348960943 | 3.84E-80 | 1.90E-78 |  |  |  |  |
| *NGFR* | -2.30420554 | 3.85E-21 | 2.83E-20 |  |  |  |  |
| *NOS2* | 3.226845005 | 8.45E-44 | 1.59E-42 |  |  |  |  |
| *NOTUM* | 2.607647952 | 2.66E-23 | 2.19E-22 |  |  |  |  |
| *OSBPL7* | 2.057396559 | 2.32E-87 | 1.30E-85 |  |  |  |  |
| *OTOP2* | -2.361188943 | 2.73E-13 | 1.26E-12 |  |  |  |  |
| *OTOP3* | -2.565835418 | 1.19E-12 | 5.27E-12 |  |  |  |  |
| *PNMT* | 2.322605343 | 3.88E-20 | 2.70E-19 |  |  |  |  |
| *PPP1R1B* | 5.903005165 | 1.04E-116 | 1.07E-114 |  |  |  |  |
| *PRAC* | 2.146466796 | 0.000666772 | 0.001350789 |  |  |  |  |
| *PRR15L* | 4.796061427 | 1.22E-285 | 1.29E-282 |  |  |  |  |
| *PYY2* | -3.763413148 | 3.27E-86 | 1.79E-84 |  |  |  |  |
| *RAB37* | 2.373364863 | 1.21E-67 | 4.43E-66 |  |  |  |  |
| *RNF157* | 2.212037909 | 8.51E-49 | 1.88E-47 |  |  |  |  |
| *RNF222* | -2.569148949 | 1.76E-16 | 9.91E-16 |  |  |  |  |
| *RTN4RL1* | -2.129015838 | 1.37E-22 | 1.09E-21 |  |  |  |  |
| *SDK2* | -2.337712755 | 1.47E-36 | 2.17E-35 |  |  |  |  |
| *SLC13A2* | 5.901300031 | 3.00E-77 | 1.38E-75 |  |  |  |  |
| *SLC47A2* | -4.379644604 | 1.16E-156 | 2.48E-154 |  |  |  |  |
| *SLC52A1* | -2.483973809 | 2.09E-43 | 3.86E-42 |  |  |  |  |
| *SOST* | -6.899358615 | 2.48E-108 | 2.14E-106 |  |  |  |  |
| *SOX15* | -4.395181489 | 8.06E-111 | 7.27E-109 |  |  |  |  |
| *SPACA3* | 2.244435041 | 1.36E-13 | 6.42E-13 |  |  |  |  |
| *SPATA22* | -2.196775211 | 4.15E-36 | 5.98E-35 |  |  |  |  |
| *SPEM1* | -2.805483309 | 6.16E-78 | 2.88E-76 |  |  |  |  |
| *SPNS3* | 2.668143396 | 7.56E-83 | 3.98E-81 |  |  |  |  |
| *ST6GALNAC1* | 2.833650803 | 4.67E-56 | 1.29E-54 |  |  |  |  |
| *ST6GALNAC2* | -2.797744606 | 1.65E-88 | 9.53E-87 |  |  |  |  |
| *TM4SF5* | 9.094416896 | 0 | 0 |  |  |  |  |
| *TMEM100* | 2.804352205 | 3.19E-40 | 5.33E-39 |  |  |  |  |
| *TNFRSF13B* | 2.270671507 | 1.20E-22 | 9.51E-22 |  |  |  |  |
| *TRIM16L* | -2.53479108 | 8.22E-92 | 5.09E-90 |  |  |  |  |
| *TRPV3* | -2.263024741 | 3.59E-38 | 5.60E-37 |  |  |  |  |
| *TSPAN10* | -2.07116257 | 2.97E-51 | 7.14E-50 |  |  |  |  |
| *TTLL6* | 5.055225424 | 5.98E-129 | 8.18E-127 |  |  |  |  |
| *TUSC5* | 2.91840159 | 4.71E-18 | 2.90E-17 |  |  |  |  |
| *USH1G* | -3.618007649 | 4.48E-29 | 4.88E-28 |  |  |  |  |
| *UTS2R* | 2.820775012 | 1.16E-27 | 1.19E-26 |  |  |  |  |
| *VTN* | 4.256231768 | 2.26E-71 | 9.22E-70 |  |  |  |  |
| *WNK4* | 2.116066655 | 1.18E-27 | 1.21E-26 |  |  |  |  |
| *ZNF750* | -3.553922569 | 8.66E-34 | 1.15E-32 |  |  |  |  |
| *ACAA2* | 2.106238001 | 9.47E-83 | 4.96E-81 |  |  |  |  |
| *AK093940* | -2.378024505 | 7.81E-32 | 9.62E-31 |  |  |  |  |
| *AK127787* | -2.090149502 | 2.59E-57 | 7.38E-56 |  |  |  |  |
| *BC042382* | -3.661130768 | 3.85E-42 | 6.83E-41 |  |  |  |  |
| *BC047599* | 2.558701007 | 2.00E-22 | 1.56E-21 |  |  |  |  |
| *BOD1L2* | 3.109443123 | 1.84E-05 | 4.44E-05 |  |  |  |  |
| *C18orf23* | -3.639238728 | 2.04E-32 | 2.57E-31 |  |  |  |  |
| *C18orf26* | -2.348182464 | 2.04E-05 | 4.90E-05 |  |  |  |  |
| *C18orf42* | 2.26576099 | 2.84E-11 | 1.13E-10 |  |  |  |  |
| *CABYR* | -2.67611441 | 8.91E-63 | 2.96E-61 |  |  |  |  |
| *CCDC68* | 2.694962589 | 1.18E-89 | 6.93E-88 |  |  |  |  |
| *CNDP1* | 3.897623062 | 3.54E-55 | 9.49E-54 |  |  |  |  |
| *DCC* | -2.32007614 | 5.09E-29 | 5.53E-28 |  |  |  |  |
| *DSC1* | -5.697183218 | 9.05E-138 | 1.42E-135 |  |  |  |  |
| *DSC2* | -2.395350422 | 3.30E-48 | 7.14E-47 |  |  |  |  |
| *DSC3* | -5.276047112 | 3.78E-68 | 1.41E-66 |  |  |  |  |
| *DSG1* | -4.600668305 | 3.26E-38 | 5.09E-37 |  |  |  |  |
| *DSG3* | -3.939898282 | 1.58E-34 | 2.15E-33 |  |  |  |  |
| *FHOD3* | -2.020795061 | 6.96E-39 | 1.11E-37 |  |  |  |  |
| *GATA6* | 4.001461807 | 2.05E-224 | 1.13E-221 |  |  |  |  |
| *KC6* | -3.173029889 | 1.48E-16 | 8.37E-16 |  |  |  |  |
| *LAMA1* | -2.097006843 | 1.42E-19 | 9.59E-19 |  |  |  |  |
| *LOC284215* | 3.59486847 | 4.23E-27 | 4.19E-26 |  |  |  |  |
| *LOC728606* | 2.395557955 | 3.36E-14 | 1.64E-13 |  |  |  |  |
| *LOC729950* | 2.442887526 | 5.82E-50 | 1.34E-48 |  |  |  |  |
| *LPIN2* | 2.047782284 | 6.28E-101 | 4.68E-99 |  |  |  |  |
| *MC2R* | 2.357792766 | 4.33E-18 | 2.67E-17 |  |  |  |  |
| *MEP1B* | 3.56794426 | 5.71E-43 | 1.04E-41 |  |  |  |  |
| *NETO1* | -3.071409742 | 1.80E-34 | 2.45E-33 |  |  |  |  |
| *NOL4* | 2.17442956 | 6.31E-15 | 3.22E-14 |  |  |  |  |
| *ONECUT2* | 4.180611006 | 1.10E-120 | 1.22E-118 |  |  |  |  |
| *PARD6G* | -2.863219749 | 3.28E-154 | 6.68E-152 |  |  |  |  |
| *RNF165* | -3.221159978 | 1.54E-63 | 5.18E-62 |  |  |  |  |
| *SALL3* | -4.854296624 | 2.64E-29 | 2.90E-28 |  |  |  |  |
| *SCARNA17* | 2.001785965 | 2.38E-53 | 6.03E-52 |  |  |  |  |
| *SERPINB11* | -2.856372078 | 1.08E-07 | 3.22E-07 |  |  |  |  |
| *SERPINB12* | -4.535410449 | 6.35E-22 | 4.85E-21 |  |  |  |  |
| *SERPINB13* | -3.628067458 | 1.83E-12 | 7.97E-12 |  |  |  |  |
| *SERPINB3* | -3.10289632 | 2.74E-16 | 1.52E-15 |  |  |  |  |
| *SIGLEC15* | 2.046284308 | 4.01E-33 | 5.17E-32 |  |  |  |  |
| *SLC14A2* | 2.142281745 | 2.80E-18 | 1.74E-17 |  |  |  |  |
| *SNORA73* | -3.017262125 | 6.24E-16 | 3.39E-15 |  |  |  |  |
| *ST8SIA3* | 3.758515732 | 4.51E-27 | 4.46E-26 |  |  |  |  |
| *SYT4* | 3.019884178 | 1.32E-19 | 8.95E-19 |  |  |  |  |
| *TNFRSF11A* | 2.866601353 | 2.23E-82 | 1.15E-80 |  |  |  |  |
| *TTR* | 7.227049029 | 8.51E-103 | 6.65E-101 |  |  |  |  |
| *U6.66* | -2.787602703 | 1.92E-13 | 8.95E-13 |  |  |  |  |
| *U7.15* | -2.249824897 | 4.10E-08 | 1.27E-07 |  |  |  |  |
| *ACER1* | -2.310837092 | 2.27E-11 | 9.12E-11 |  |  |  |  |
| *ATP4A* | 3.646096096 | 6.72E-25 | 6.02E-24 |  |  |  |  |
| *B3GNT3* | 2.834097976 | 3.70E-110 | 3.27E-108 |  |  |  |  |
| *BC034929* | 2.657634229 | 1.85E-84 | 9.93E-83 |  |  |  |  |
| *BC068609* | 2.453573828 | 2.95E-12 | 1.27E-11 |  |  |  |  |
| *C19orf21* | 3.756815642 | 9.04E-186 | 3.07E-183 |  |  |  |  |
| *C19orf45* | 2.456164493 | 1.74E-45 | 3.46E-44 |  |  |  |  |
| *C19orf69* | 3.780245279 | 5.52E-43 | 1.01E-41 |  |  |  |  |
| *C19orf77* | 8.079969213 | 0 | 0 |  |  |  |  |
| *C3P1* | 5.794083136 | 1.48E-69 | 5.71E-68 |  |  |  |  |
| *CACNG6* | 3.534662998 | 4.32E-25 | 3.91E-24 |  |  |  |  |
| *CACNG8* | 2.08821257 | 1.69E-27 | 1.71E-26 |  |  |  |  |
| *CASP14* | -8.004282816 | 2.02E-93 | 1.30E-91 |  |  |  |  |
| *CCL25* | 8.545222511 | 9.16E-125 | 1.13E-122 |  |  |  |  |
| *CD79A* | 2.203511439 | 4.21E-24 | 3.62E-23 |  |  |  |  |
| *CDC42EP5* | 3.155219501 | 2.81E-103 | 2.22E-101 |  |  |  |  |
| *CEACAM18* | 5.955112034 | 6.97E-75 | 3.03E-73 |  |  |  |  |
| *CEACAM19* | -2.620910198 | 1.58E-68 | 5.98E-67 |  |  |  |  |
| *CEACAM20* | 4.475682976 | 1.01E-43 | 1.89E-42 |  |  |  |  |
| *CEACAM5* | 2.820317901 | 1.96E-31 | 2.37E-30 |  |  |  |  |
| *CEACAM6* | 2.282786622 | 1.38E-23 | 1.15E-22 |  |  |  |  |
| *CGB5* | 2.26530656 | 1.35E-10 | 5.15E-10 |  |  |  |  |
| *CGB8* | -2.07054362 | 1.59E-21 | 1.19E-20 |  |  |  |  |
| *CLC* | 3.127079622 | 1.32E-23 | 1.10E-22 |  |  |  |  |
| *CNFN* | -2.365240974 | 5.94E-14 | 2.86E-13 |  |  |  |  |
| *CNN1* | 2.045151127 | 8.06E-15 | 4.10E-14 |  |  |  |  |
| *CREB3L3* | 7.461987315 | 1.98E-179 | 6.11E-177 |  |  |  |  |
| *CYP2B6* | 7.45414552 | 1.59E-163 | 3.76E-161 |  |  |  |  |
| *CYP2B7P1* | 3.104769413 | 1.16E-39 | 1.91E-38 |  |  |  |  |
| *CYP4F11* | -3.555481711 | 4.55E-61 | 1.46E-59 |  |  |  |  |
| *CYP4F22* | -3.041343805 | 4.17E-31 | 4.98E-30 |  |  |  |  |
| *CYP4F2* | 4.332535095 | 7.57E-54 | 1.95E-52 |  |  |  |  |
| *DKFZp434J0226* | -2.40021805 | 6.73E-24 | 5.72E-23 |  |  |  |  |
| *DMKN* | -2.654443118 | 2.36E-36 | 3.43E-35 |  |  |  |  |
| *DPF1* | -2.68927697 | 1.69E-59 | 5.14E-58 |  |  |  |  |
| *EFNA2* | 4.859544479 | 1.25E-140 | 2.05E-138 |  |  |  |  |
| *FAM83E* | 2.39986162 | 8.84E-60 | 2.72E-58 |  |  |  |  |
| *FBXO27* | -2.68501951 | 2.01E-53 | 5.11E-52 |  |  |  |  |
| *FCGBP* | 3.614224931 | 3.13E-47 | 6.56E-46 |  |  |  |  |
| *FCGRT* | 2.023780699 | 1.54E-107 | 1.30E-105 |  |  |  |  |
| *FFAR3.2* | 2.236197689 | 4.92E-08 | 1.52E-07 |  |  |  |  |
| *FOXA3* | 6.257732741 | 0 | 0 |  |  |  |  |
| *GDF15* | 3.606393027 | 3.77E-110 | 3.33E-108 |  |  |  |  |
| *GNA15* | -2.54851418 | 2.90E-70 | 1.15E-68 |  |  |  |  |
| *HIF3A* | 2.07237046 | 1.90E-18 | 1.19E-17 |  |  |  |  |
| *HPN* | 3.593777573 | 1.39E-38 | 2.19E-37 |  |  |  |  |
| *HSH2D* | 2.11258297 | 3.70E-116 | 3.77E-114 |  |  |  |  |
| *HSPB6* | 3.146454399 | 1.56E-34 | 2.13E-33 |  |  |  |  |
| *ICAM5* | -2.074259785 | 2.70E-37 | 4.08E-36 |  |  |  |  |
| *IGFL1* | -4.506371688 | 7.38E-36 | 1.05E-34 |  |  |  |  |
| *IGFL3* | -4.897238195 | 4.64E-53 | 1.17E-51 |  |  |  |  |
| *IGSF23* | 3.477754661 | 6.02E-44 | 1.14E-42 |  |  |  |  |
| *IZUMO2* | 3.120715427 | 1.94E-15 | 1.02E-14 |  |  |  |  |
| *JSRP1* | 2.66540928 | 3.18E-40 | 5.31E-39 |  |  |  |  |
| *KLC3* | -2.831468341 | 7.79E-75 | 3.38E-73 |  |  |  |  |
| *KLK14* | -3.482162737 | 7.30E-49 | 1.61E-47 |  |  |  |  |
| *KLK15* | 3.009380019 | 3.29E-19 | 2.18E-18 |  |  |  |  |
| *KLK1* | 2.841318349 | 1.95E-38 | 3.06E-37 |  |  |  |  |
| *KLK3* | 3.006915514 | 2.57E-18 | 1.60E-17 |  |  |  |  |
| *KLK5* | -2.538794201 | 9.82E-09 | 3.23E-08 |  |  |  |  |
| *KLK8* | -2.51667552 | 8.59E-19 | 5.53E-18 |  |  |  |  |
| *KLK9* | -3.420071023 | 8.14E-32 | 1.00E-30 |  |  |  |  |
| *KRTDAP* | -4.916450458 | 1.60E-26 | 1.54E-25 |  |  |  |  |
| *LGALS4* | 9.153759886 | 0 | 0 |  |  |  |  |
| *LGALS7B* | -4.427287411 | 7.55E-42 | 1.33E-40 |  |  |  |  |
| *LGALS7* | -4.371268298 | 9.83E-23 | 7.83E-22 |  |  |  |  |
| *LIM2* | -2.653155603 | 1.51E-12 | 6.61E-12 |  |  |  |  |
| *LOC100507003* | 2.036512499 | 3.43E-10 | 1.26E-09 |  |  |  |  |
| *LOC113230* | 2.283356061 | 1.36E-74 | 5.87E-73 |  |  |  |  |
| *LOC646862* | -2.565831301 | 1.50E-16 | 8.46E-16 |  |  |  |  |
| *LYPD3* | -3.339073127 | 3.54E-37 | 5.35E-36 |  |  |  |  |
| *LYPD5* | -2.170343739 | 7.73E-47 | 1.60E-45 |  |  |  |  |
| *MADCAM1* | 3.086313418 | 2.59E-40 | 4.35E-39 |  |  |  |  |
| *MIA* | 4.069971684 | 7.45E-56 | 2.04E-54 |  |  |  |  |
| *MIR3189* | 4.211736016 | 1.85E-58 | 5.41E-57 |  |  |  |  |
| *MIR519A1* | 2.276543972 | 0.000768558 | 0.001544926 |  |  |  |  |
| *MIR7-3HG* | 3.21652957 | 7.14E-19 | 4.62E-18 |  |  |  |  |
| *Mir_324.4* | 3.462454428 | 1.99E-16 | 1.12E-15 |  |  |  |  |
| *NANOS3* | 2.435009274 | 1.05E-35 | 1.49E-34 |  |  |  |  |
| *NKPD1* | -2.742167278 | 1.41E-57 | 4.05E-56 |  |  |  |  |
| *NPHS1* | 2.56732481 | 1.89E-27 | 1.91E-26 |  |  |  |  |
| *NTF4* | -3.295212089 | 1.66E-64 | 5.68E-63 |  |  |  |  |
| *ONECUT3* | 5.39836361 | 5.45E-65 | 1.88E-63 |  |  |  |  |
| *OR7A5* | -3.945429863 | 3.17E-26 | 3.03E-25 |  |  |  |  |
| *OR7C1* | -2.465756515 | 1.67E-12 | 7.29E-12 |  |  |  |  |
| *PALM3* | 3.280608572 | 2.12E-59 | 6.43E-58 |  |  |  |  |
| *PAPL* | -3.498484012 | 9.53E-31 | 1.12E-29 |  |  |  |  |
| *PDE4C* | 2.870485824 | 9.50E-60 | 2.91E-58 |  |  |  |  |
| *PLAC2* | -3.647027631 | 8.01E-50 | 1.83E-48 |  |  |  |  |
| *PRG1* | -2.874001557 | 1.13E-18 | 7.21E-18 |  |  |  |  |
| *PRKCG* | 5.062522584 | 1.94E-111 | 1.77E-109 |  |  |  |  |
| *PRODH2* | 3.385670842 | 7.23E-21 | 5.24E-20 |  |  |  |  |
| *PSG4* | -3.396081748 | 2.88E-19 | 1.91E-18 |  |  |  |  |
| *PSG5* | -2.84249171 | 5.72E-13 | 2.58E-12 |  |  |  |  |
| *PTPRH* | 3.196677841 | 9.40E-105 | 7.67E-103 |  |  |  |  |
| *RDH8* | 2.601241125 | 6.78E-20 | 4.66E-19 |  |  |  |  |
| *RGL3* | 2.552893812 | 5.81E-42 | 1.02E-40 |  |  |  |  |
| *RHPN2* | 3.348688491 | 4.10E-185 | 1.36E-182 |  |  |  |  |
| *RYR1* | -3.085941305 | 2.45E-76 | 1.10E-74 |  |  |  |  |
| *S1PR5* | -3.992995026 | 5.43E-157 | 1.17E-154 |  |  |  |  |
| *SBSN* | -3.756198824 | 6.85E-19 | 4.44E-18 |  |  |  |  |
| *SHD* | 4.206192 | 2.60E-93 | 1.66E-91 |  |  |  |  |
| *SLC1A6* | -3.148965001 | 3.55E-16 | 1.96E-15 |  |  |  |  |
| *SLC5A5* | 3.539459349 | 4.31E-36 | 6.20E-35 |  |  |  |  |
| *SLC7A9* | 3.900427598 | 9.98E-59 | 2.94E-57 |  |  |  |  |
| *SPIB* | 3.129308531 | 7.05E-40 | 1.17E-38 |  |  |  |  |
| *SULT2A1* | 5.588746883 | 4.40E-43 | 8.08E-42 |  |  |  |  |
| *THEG* | -2.229663325 | 5.85E-10 | 2.12E-09 |  |  |  |  |
| *TJP3* | 3.137703523 | 1.31E-118 | 1.40E-116 |  |  |  |  |
| *TM6SF2* | 3.136807287 | 1.29E-48 | 2.83E-47 |  |  |  |  |
| *TMEM150B* | 3.816739094 | 2.49E-133 | 3.66E-131 |  |  |  |  |
| *TNNT1* | -2.017273484 | 2.14E-17 | 1.28E-16 |  |  |  |  |
| *TSKS* | -3.083344376 | 1.42E-47 | 3.01E-46 |  |  |  |  |
| *UCA1* | 2.827831254 | 3.11E-28 | 3.26E-27 |  |  |  |  |
| *UNC13A* | 2.515109788 | 5.05E-36 | 7.24E-35 |  |  |  |  |
| *VSIG10L* | -2.224995686 | 1.07E-24 | 9.45E-24 |  |  |  |  |
| *VSTM2B* | 3.072175809 | 8.50E-12 | 3.53E-11 |  |  |  |  |
| *ZNF69* | 2.997555196 | 5.42E-149 | 1.01E-146 |  |  |  |  |
| *ZNF812* | -3.134255635 | 2.30E-27 | 2.31E-26 |  |  |  |  |
| *ABCA12* | -2.616637956 | 3.33E-28 | 3.49E-27 |  |  |  |  |
| *ABCG5* | 3.223287467 | 3.21E-57 | 9.10E-56 |  |  |  |  |
| *ABCG8* | 4.689077574 | 2.87E-66 | 1.02E-64 |  |  |  |  |
| *ACMSD* | 2.18395408 | 1.15E-27 | 1.18E-26 |  |  |  |  |
| *ADAM23* | -3.792999907 | 2.26E-104 | 1.82E-102 |  |  |  |  |
| *ADD2* | -2.664004306 | 5.17E-38 | 8.00E-37 |  |  |  |  |
| *AGXT* | 3.413884185 | 4.80E-38 | 7.45E-37 |  |  |  |  |
| *AK125871* | 2.048238349 | 5.85E-38 | 9.03E-37 |  |  |  |  |
| *AK127124* | -3.485466586 | 1.10E-46 | 2.26E-45 |  |  |  |  |
| *AK127400* | 2.118336943 | 3.96E-12 | 1.68E-11 |  |  |  |  |
| *AK311291* | -2.834689108 | 4.12E-14 | 2.00E-13 |  |  |  |  |
| *ALPI* | 7.96501074 | 8.65E-137 | 1.34E-134 |  |  |  |  |
| *ALPPL2* | 6.022165346 | 4.53E-81 | 2.27E-79 |  |  |  |  |
| *ALPP* | 3.852161873 | 6.12E-38 | 9.43E-37 |  |  |  |  |
| *APOB* | 6.141100633 | 3.95E-84 | 2.11E-82 |  |  |  |  |
| *AQP12A* | 3.995486773 | 4.57E-23 | 3.72E-22 |  |  |  |  |
| *AQP12B* | 3.749921602 | 4.53E-25 | 4.09E-24 |  |  |  |  |
| *ARHGEF4* | -3.463549558 | 9.26E-78 | 4.30E-76 |  |  |  |  |
| *ASPRV1* | -2.304404763 | 1.14E-55 | 3.11E-54 |  |  |  |  |
| *AX746677* | 4.058831388 | 3.65E-48 | 7.89E-47 |  |  |  |  |
| *AX746725* | -2.344574891 | 1.95E-22 | 1.53E-21 |  |  |  |  |
| *B3GNT7* | 2.788881332 | 9.76E-67 | 3.51E-65 |  |  |  |  |
| *BC016143.6* | -6.369012492 | 4.11E-47 | 8.58E-46 |  |  |  |  |
| *BC016831* | 5.298288957 | 1.66E-23 | 1.38E-22 |  |  |  |  |
| *BC040861* | -2.049838129 | 4.62E-22 | 3.56E-21 |  |  |  |  |
| *BC051708* | -2.352357869 | 8.27E-44 | 1.56E-42 |  |  |  |  |
| *BC051759* | -3.458844959 | 1.36E-64 | 4.66E-63 |  |  |  |  |
| *C2orf40* | 2.632000544 | 1.11E-18 | 7.08E-18 |  |  |  |  |
| *C2orf70* | 2.07139505 | 1.75E-30 | 2.03E-29 |  |  |  |  |
| *C2orf71* | -3.061740702 | 1.40E-24 | 1.23E-23 |  |  |  |  |
| *C2orf72* | 5.098691649 | 3.90E-238 | 2.38E-235 |  |  |  |  |
| *C2orf82* | 3.015325674 | 5.49E-67 | 1.99E-65 |  |  |  |  |
| *C2orf89* | 3.310858488 | 1.80E-76 | 8.16E-75 |  |  |  |  |
| *CCDC108* | 3.218522834 | 8.43E-60 | 2.60E-58 |  |  |  |  |
| *CCDC140* | -2.731371133 | 9.37E-05 | 0.000209108 |  |  |  |  |
| *CFC1B* | 4.879217126 | 8.84E-13 | 3.93E-12 |  |  |  |  |
| *CIB4* | 3.320715083 | 2.55E-28 | 2.68E-27 |  |  |  |  |
| *CLIP4* | -2.005474093 | 2.59E-54 | 6.77E-53 |  |  |  |  |
| *CPS1-IT1* | 4.080480321 | 8.75E-16 | 4.72E-15 |  |  |  |  |
| *CPS1* | 4.996120087 | 5.60E-70 | 2.19E-68 |  |  |  |  |
| *CRYGD* | 2.559663617 | 7.40E-08 | 2.24E-07 |  |  |  |  |
| *CTNNA2* | 2.382439893 | 1.43E-15 | 7.59E-15 |  |  |  |  |
| *CXCR7* | -2.459403251 | 5.37E-87 | 2.97E-85 |  |  |  |  |
| *CYP27C1* | -3.494252116 | 3.08E-78 | 1.46E-76 |  |  |  |  |
| *DAPL1* | -3.249886157 | 5.74E-26 | 5.40E-25 |  |  |  |  |
| *DLX1* | -2.758151041 | 2.42E-27 | 2.42E-26 |  |  |  |  |
| *DLX2* | -2.453375121 | 2.79E-20 | 1.96E-19 |  |  |  |  |
| *DNAH6* | 2.203340012 | 1.38E-46 | 2.83E-45 |  |  |  |  |
| *DNAJB3* | -2.68459968 | 2.83E-24 | 2.46E-23 |  |  |  |  |
| *DPP4* | 2.253267575 | 1.54E-40 | 2.60E-39 |  |  |  |  |
| *ECEL1P2* | 2.414107416 | 4.88E-13 | 2.21E-12 |  |  |  |  |
| *EN1* | -6.458972551 | 1.14E-96 | 7.84E-95 |  |  |  |  |
| *EPCAM* | 2.17207332 | 2.80E-91 | 1.72E-89 |  |  |  |  |
| *FABP1* | 10.24641987 | 6.08E-179 | 1.80E-176 |  |  |  |  |
| *FAM123C* | 3.608986548 | 6.84E-30 | 7.70E-29 |  |  |  |  |
| *FEV* | 5.036641867 | 3.97E-57 | 1.12E-55 |  |  |  |  |
| *FOXI3* | -5.582322861 | 4.00E-29 | 4.37E-28 |  |  |  |  |
| *FRZB* | 2.451600125 | 5.97E-49 | 1.32E-47 |  |  |  |  |
| *FZD5* | 2.224507537 | 1.46E-116 | 1.51E-114 |  |  |  |  |
| *GAL3ST2* | 2.3884902 | 1.46E-24 | 1.29E-23 |  |  |  |  |
| *GALM* | 2.464780899 | 5.29E-163 | 1.22E-160 |  |  |  |  |
| *GALNT14* | -2.415023137 | 9.16E-31 | 1.08E-29 |  |  |  |  |
| *GCG* | 4.18347559 | 9.77E-17 | 5.59E-16 |  |  |  |  |
| *GCKR* | 2.968080257 | 2.99E-48 | 6.49E-47 |  |  |  |  |
| *GKN1* | 9.43849525 | 8.67E-113 | 8.25E-111 |  |  |  |  |
| *GKN2* | 8.703037519 | 1.26E-101 | 9.59E-100 |  |  |  |  |
| *GLI2* | -2.136216788 | 2.21E-45 | 4.39E-44 |  |  |  |  |
| *GPAT2.1* | 2.071560389 | 1.60E-09 | 5.60E-09 |  |  |  |  |
| *GPBAR1* | 2.921052398 | 1.46E-76 | 6.64E-75 |  |  |  |  |
| *GPC1* | -2.787993033 | 3.11E-123 | 3.74E-121 |  |  |  |  |
| *GPR148* | 2.142576107 | 2.03E-06 | 5.38E-06 |  |  |  |  |
| *GPR1* | -2.152898225 | 4.30E-28 | 4.47E-27 |  |  |  |  |
| *GPR35* | 5.189716643 | 0 | 0 |  |  |  |  |
| *GRHL1* | -2.271887958 | 8.42E-48 | 1.80E-46 |  |  |  |  |
| *HNMT* | 2.146079401 | 5.01E-123 | 5.96E-121 |  |  |  |  |
| *HOXD10* | -3.296217246 | 8.10E-28 | 8.34E-27 |  |  |  |  |
| *HOXD11* | -3.214269337 | 1.22E-13 | 5.79E-13 |  |  |  |  |
| *HOXD13* | -2.691150439 | 1.64E-09 | 5.74E-09 |  |  |  |  |
| *IGKV* | 2.225827893 | 2.19E-14 | 1.08E-13 |  |  |  |  |
| *IHH* | 8.728910891 | 0 | 0 |  |  |  |  |
| *IL1A* | -3.750861507 | 6.01E-66 | 2.11E-64 |  |  |  |  |
| *IL1F10* | -4.263673481 | 6.37E-20 | 4.39E-19 |  |  |  |  |
| *IL36B* | -3.593584954 | 9.46E-21 | 6.81E-20 |  |  |  |  |
| *IL36G* | -4.930309711 | 1.46E-42 | 2.62E-41 |  |  |  |  |
| *IL36RN* | -4.121126662 | 2.34E-21 | 1.74E-20 |  |  |  |  |
| *IQCA1* | -2.82856593 | 1.16E-71 | 4.80E-70 |  |  |  |  |
| *KCNJ3* | 5.375297022 | 1.10E-90 | 6.62E-89 |  |  |  |  |
| *LINC00486* | -3.193280259 | 2.31E-25 | 2.12E-24 |  |  |  |  |
| *LOC100286922* | -2.891136156 | 5.41E-27 | 5.33E-26 |  |  |  |  |
| *LOC100507334* | 2.385889715 | 2.25E-11 | 9.08E-11 |  |  |  |  |
| *LOC150622* | 3.465931076 | 6.41E-23 | 5.17E-22 |  |  |  |  |
| *LOC200726* | -3.146556075 | 9.52E-10 | 3.39E-09 |  |  |  |  |
| *LOC200772* | 3.19110502 | 4.90E-27 | 4.83E-26 |  |  |  |  |
| *LOC285000* | 3.366091443 | 1.14E-27 | 1.17E-26 |  |  |  |  |
| *LOC285084* | -3.332830856 | 1.52E-27 | 1.55E-26 |  |  |  |  |
| *LOC348761* | 2.799552222 | 5.32E-54 | 1.37E-52 |  |  |  |  |
| *LOC388942* | 4.017371808 | 1.43E-32 | 1.80E-31 |  |  |  |  |
| *LOC389043* | 2.650693359 | 5.63E-13 | 2.54E-12 |  |  |  |  |
| *LOC440925* | 2.941364379 | 9.35E-51 | 2.20E-49 |  |  |  |  |
| *LOC727982* | -2.823115909 | 2.36E-08 | 7.51E-08 |  |  |  |  |
| *LRP1B* | -2.726898528 | 1.67E-32 | 2.11E-31 |  |  |  |  |
| *MGAT4A* | 2.090994197 | 3.13E-83 | 1.67E-81 |  |  |  |  |
| *MIR149* | -2.35364999 | 1.87E-12 | 8.16E-12 |  |  |  |  |
| *MIR3131* | 7.518635621 | 2.19E-54 | 5.74E-53 |  |  |  |  |
| *MIR559* | 2.064347846 | 5.16E-29 | 5.60E-28 |  |  |  |  |
| *MLPH* | 3.472147934 | 1.83E-101 | 1.39E-99 |  |  |  |  |
| *MOGAT1* | 2.253127643 | 2.63E-15 | 1.37E-14 |  |  |  |  |
| *MSGN1* | -3.193189248 | 5.03E-15 | 2.59E-14 |  |  |  |  |
| *MYO7B* | 5.5912418 | 2.60E-176 | 7.43E-174 |  |  |  |  |
| *Mir_584.21* | -2.262126703 | 1.06E-28 | 1.13E-27 |  |  |  |  |
| *NAT8B* | 3.274190052 | 1.39E-44 | 2.71E-43 |  |  |  |  |
| *NAT8* | 2.341232923 | 2.16E-19 | 1.44E-18 |  |  |  |  |
| *NEU2* | -4.254052425 | 2.02E-25 | 1.85E-24 |  |  |  |  |
| *NEU4* | 3.304877329 | 9.77E-47 | 2.02E-45 |  |  |  |  |
| *NEUROD1* | 3.653968958 | 6.13E-26 | 5.76E-25 |  |  |  |  |
| *NMUR1* | 2.21751225 | 1.57E-42 | 2.82E-41 |  |  |  |  |
| *NOSTRIN* | 3.112279867 | 3.70E-185 | 1.24E-182 |  |  |  |  |
| *OSBPL6* | -2.192988216 | 2.71E-47 | 5.68E-46 |  |  |  |  |
| *PAX3* | -3.497464731 | 1.05E-14 | 5.28E-14 |  |  |  |  |
| *PCDP1* | 4.173720899 | 4.64E-73 | 1.95E-71 |  |  |  |  |
| *PDE11A* | 2.279339157 | 4.80E-33 | 6.16E-32 |  |  |  |  |
| *PKDCC* | 3.2525938 | 2.28E-112 | 2.14E-110 |  |  |  |  |
| *POMC* | -2.598631348 | 2.02E-39 | 3.29E-38 |  |  |  |  |
| *PRLH* | 2.317214726 | 1.06E-06 | 2.89E-06 |  |  |  |  |
| *RAB17* | 2.912211077 | 8.19E-138 | 1.29E-135 |  |  |  |  |
| *RAD51AP2* | -2.123542657 | 7.23E-25 | 6.47E-24 |  |  |  |  |
| *REG1A* | 8.841574005 | 2.58E-154 | 5.29E-152 |  |  |  |  |
| *REG1B* | 7.91934474 | 2.86E-76 | 1.29E-74 |  |  |  |  |
| *REG3A* | 9.717017572 | 2.63E-142 | 4.38E-140 |  |  |  |  |
| *REG3G* | 6.47675791 | 3.40E-44 | 6.48E-43 |  |  |  |  |
| *RN7SK.51* | -3.361201872 | 2.90E-16 | 1.61E-15 |  |  |  |  |
| *SCN9A* | -3.108877151 | 4.63E-55 | 1.24E-53 |  |  |  |  |
| *SCTR* | 3.565539047 | 4.64E-43 | 8.51E-42 |  |  |  |  |
| *SH2D6* | 2.028911363 | 1.01E-27 | 1.04E-26 |  |  |  |  |
| *SLC19A3* | 4.567676785 | 6.02E-90 | 3.58E-88 |  |  |  |  |
| *SLC40A1* | 2.215090993 | 4.70E-78 | 2.21E-76 |  |  |  |  |
| *SLC4A10* | 2.05710291 | 2.10E-26 | 2.03E-25 |  |  |  |  |
| *SLC9A4* | 2.613569131 | 2.53E-19 | 1.68E-18 |  |  |  |  |
| *SMYD1* | 4.016099434 | 2.27E-24 | 1.98E-23 |  |  |  |  |
| *SP5* | 4.475619389 | 1.58E-92 | 9.92E-91 |  |  |  |  |
| *ST6GAL2* | -2.418003935 | 2.16E-27 | 2.17E-26 |  |  |  |  |
| *SULT1C2P1* | 4.926709535 | 3.31E-87 | 1.84E-85 |  |  |  |  |
| *SULT1C2* | 6.73915687 | 1.59E-220 | 7.97E-218 |  |  |  |  |
| *SULT1C3* | 5.621850016 | 1.01E-57 | 2.89E-56 |  |  |  |  |
| *TCF23* | 3.192155265 | 7.42E-18 | 4.53E-17 |  |  |  |  |
| *TM4SF20* | 8.902351947 | 2.29E-224 | 1.24E-221 |  |  |  |  |
| *TRIM54* | 3.522421044 | 2.96E-44 | 5.68E-43 |  |  |  |  |
| *VIL1* | 6.651149611 | 2.93E-254 | 2.07E-251 |  |  |  |  |
| *VIT* | -2.474272883 | 6.19E-26 | 5.82E-25 |  |  |  |  |
| *VSNL1* | -2.205477092 | 3.10E-25 | 2.81E-24 |  |  |  |  |
| *VWA3B* | 2.038303869 | 2.59E-35 | 3.62E-34 |  |  |  |  |
| *abParts.4* | 2.321257826 | 2.30E-22 | 1.79E-21 |  |  |  |  |
| *AF143870* | 4.861197092 | 9.60E-112 | 8.86E-110 |  |  |  |  |
| *AK056267* | 3.62427689 | 4.23E-11 | 1.67E-10 |  |  |  |  |
| *AK125594* | 4.548016451 | 2.15E-38 | 3.36E-37 |  |  |  |  |
| *APCDD1L* | -2.804981037 | 2.37E-47 | 4.99E-46 |  |  |  |  |
| *AX747171* | -4.574422689 | 5.24E-94 | 3.42E-92 |  |  |  |  |
| *AX747649* | 2.665296493 | 7.56E-37 | 1.13E-35 |  |  |  |  |
| *BANF2* | 3.36403797 | 1.60E-16 | 9.00E-16 |  |  |  |  |
| *BC027448* | -3.76624933 | 5.80E-52 | 1.41E-50 |  |  |  |  |
| *BC071794* | 5.352417651 | 5.04E-148 | 9.12E-146 |  |  |  |  |
| *BC141903* | 2.649011184 | 1.76E-33 | 2.31E-32 |  |  |  |  |
| *BCAS1* | 4.090498471 | 2.60E-95 | 1.73E-93 |  |  |  |  |
| *BIRC7* | 2.538695464 | 3.52E-30 | 4.01E-29 |  |  |  |  |
| *BMP7* | -2.232433617 | 1.08E-25 | 1.01E-24 |  |  |  |  |
| *BPIFA2* | 4.307016824 | 1.94E-33 | 2.55E-32 |  |  |  |  |
| *BPIFB1* | 5.09799219 | 1.24E-50 | 2.92E-49 |  |  |  |  |
| *BPIFB2* | 4.5716164 | 2.90E-28 | 3.05E-27 |  |  |  |  |
| *C20orf197* | -3.311820462 | 4.39E-63 | 1.47E-61 |  |  |  |  |
| *C20orf202* | 2.086761563 | 5.78E-50 | 1.33E-48 |  |  |  |  |
| *C20orf85* | 3.157314824 | 6.00E-16 | 3.27E-15 |  |  |  |  |
| *CDH26* | -3.251721652 | 2.57E-64 | 8.76E-63 |  |  |  |  |
| *CDH4* | -2.000049113 | 4.11E-28 | 4.29E-27 |  |  |  |  |
| *CYP24A1* | -3.196790231 | 7.31E-23 | 5.87E-22 |  |  |  |  |
| *DUSP15* | 2.0430282 | 1.93E-27 | 1.94E-26 |  |  |  |  |
| *EDN3* | 2.739039898 | 3.72E-27 | 3.70E-26 |  |  |  |  |
| *FAM83C* | -4.140603906 | 1.58E-28 | 1.68E-27 |  |  |  |  |
| *FER1L4.1* | 2.549590285 | 1.26E-43 | 2.35E-42 |  |  |  |  |
| *FER1L4.2* | 2.475535201 | 5.28E-40 | 8.77E-39 |  |  |  |  |
| *FOXA2* | 4.844211107 | 8.43E-156 | 1.76E-153 |  |  |  |  |
| *GATA5* | 5.889261432 | 4.74E-83 | 2.51E-81 |  |  |  |  |
| *HNF4A* | 6.55181193 | 0 | 0 |  |  |  |  |
| *INSM1* | 4.432642924 | 9.67E-61 | 3.09E-59 |  |  |  |  |
| *KCNG1* | -2.380416941 | 1.36E-31 | 1.66E-30 |  |  |  |  |
| *KCNK15* | 2.424848841 | 2.70E-30 | 3.08E-29 |  |  |  |  |
| *KCNQ2.1* | 5.103163247 | 1.47E-33 | 1.95E-32 |  |  |  |  |
| *KCNQ2.2* | 2.85202327 | 3.18E-24 | 2.75E-23 |  |  |  |  |
| *LBP* | 2.571853334 | 6.98E-21 | 5.07E-20 |  |  |  |  |
| *LINC00261* | 7.158054589 | 6.08E-156 | 1.28E-153 |  |  |  |  |
| *LINC00494* | 3.315877634 | 1.37E-48 | 2.98E-47 |  |  |  |  |
| *LOC100127888* | 2.065557797 | 2.22E-38 | 3.47E-37 |  |  |  |  |
| *LOC149773* | -2.416154488 | 2.22E-29 | 2.44E-28 |  |  |  |  |
| *LOC149950* | 2.084708052 | 0.000596991 | 0.001216412 |  |  |  |  |
| *LOC339593* | -2.218305634 | 3.54E-08 | 1.11E-07 |  |  |  |  |
| *LOC63930* | 2.368734646 | 1.78E-12 | 7.78E-12 |  |  |  |  |
| *LOC643406* | 2.516928202 | 6.20E-15 | 3.17E-14 |  |  |  |  |
| *MACROD2* | 2.845883204 | 2.00E-49 | 4.51E-48 |  |  |  |  |
| *MIR1257* | 2.017680675 | 5.51E-07 | 1.54E-06 |  |  |  |  |
| *MIR3646* | 5.403633761 | 4.52E-33 | 5.81E-32 |  |  |  |  |
| *MIR4756* | 3.071433567 | 9.43E-11 | 3.63E-10 |  |  |  |  |
| *MYT1* | 2.358359773 | 8.75E-21 | 6.31E-20 |  |  |  |  |
| *NFATC2* | 2.053174102 | 1.36E-74 | 5.86E-73 |  |  |  |  |
| *NKX2-4* | -3.323138141 | 8.78E-07 | 2.41E-06 |  |  |  |  |
| *NTSR1* | 3.159018096 | 1.27E-33 | 1.68E-32 |  |  |  |  |
| *PAX1* | -2.725210692 | 2.77E-10 | 1.03E-09 |  |  |  |  |
| *PCK1* | 5.368273415 | 4.49E-91 | 2.74E-89 |  |  |  |  |
| *R3HDML* | 5.103561215 | 3.37E-125 | 4.21E-123 |  |  |  |  |
| *SEMG2* | 5.422748256 | 3.20E-66 | 1.13E-64 |  |  |  |  |
| *SGK2* | 3.78203202 | 1.73E-120 | 1.92E-118 |  |  |  |  |
| *SLC17A9* | 2.68002754 | 4.05E-71 | 1.64E-69 |  |  |  |  |
| *SRMS* | 2.251702282 | 1.00E-38 | 1.58E-37 |  |  |  |  |
| *TFAP2C* | -3.17939196 | 2.72E-55 | 7.35E-54 |  |  |  |  |
| *TGM6* | -2.829919989 | 4.98E-09 | 1.67E-08 |  |  |  |  |
| *THBD* | -2.609555142 | 5.82E-101 | 4.35E-99 |  |  |  |  |
| *TMEM74B* | 2.129987201 | 1.33E-42 | 2.39E-41 |  |  |  |  |
| *TNNC2* | 2.338994493 | 3.35E-30 | 3.82E-29 |  |  |  |  |
| *WFDC12* | -3.627005094 | 5.45E-32 | 6.76E-31 |  |  |  |  |
| *WFDC5* | -3.832948543 | 1.16E-24 | 1.03E-23 |  |  |  |  |
| *XKR7* | 2.414354326 | 2.98E-12 | 1.28E-11 |  |  |  |  |
| *AIRE* | 2.37993691 | 9.95E-24 | 8.37E-23 |  |  |  |  |
| *B3GALT5* | 3.262226246 | 6.00E-50 | 1.38E-48 |  |  |  |  |
| *CBR3* | -2.104620738 | 2.16E-76 | 9.76E-75 |  |  |  |  |
| *CHODL* | -3.39027102 | 8.11E-51 | 1.91E-49 |  |  |  |  |
| *CLDN17* | -2.530431469 | 0.000215509 | 0.000462455 |  |  |  |  |
| *CLDN8* | -3.850692575 | 1.06E-15 | 5.68E-15 |  |  |  |  |
| *CYP4F29P* | -2.061762724 | 1.43E-11 | 5.82E-11 |  |  |  |  |
| *DQ586768.5* | -2.055386151 | 0.000245218 | 0.00052286 |  |  |  |  |
| *DQ588725* | 3.642391747 | 1.85E-16 | 1.04E-15 |  |  |  |  |
| *DQ590668* | 3.886472455 | 2.47E-12 | 1.07E-11 |  |  |  |  |
| *DQ601137* | 3.791946567 | 7.98E-12 | 3.33E-11 |  |  |  |  |
| *DSCAM* | -2.203468538 | 9.16E-16 | 4.94E-15 |  |  |  |  |
| *DSCR4* | 2.827872204 | 5.52E-07 | 1.55E-06 |  |  |  |  |
| *DSCR8* | 2.274722169 | 4.58E-05 | 0.000105827 |  |  |  |  |
| *FAM3B* | 2.22799576 | 1.90E-22 | 1.49E-21 |  |  |  |  |
| *KCNE2* | 3.937451184 | 2.77E-53 | 7.00E-52 |  |  |  |  |
| *KCNJ15* | -2.179268964 | 4.54E-26 | 4.29E-25 |  |  |  |  |
| *KRTAP10-4* | 3.271669203 | 3.83E-11 | 1.51E-10 |  |  |  |  |
| *KRTAP10-5* | 2.349248593 | 0.000501318 | 0.001031794 |  |  |  |  |
| *KRTAP13-2* | -2.226564351 | 1.08E-05 | 2.67E-05 |  |  |  |  |
| *KRTAP19-1* | -7.595431718 | 9.10E-44 | 1.71E-42 |  |  |  |  |
| *KRTAP21-2* | -2.386267431 | 0.000406466 | 0.000845562 |  |  |  |  |
| *LINC00114* | 3.650652802 | 3.89E-33 | 5.03E-32 |  |  |  |  |
| *LOC284837* | -2.347573847 | 3.00E-73 | 1.27E-71 |  |  |  |  |
| *MIR125B2* | -2.494503472 | 4.31E-07 | 1.22E-06 |  |  |  |  |
| *MIR3197* | 2.487798597 | 1.05E-16 | 5.98E-16 |  |  |  |  |
| *RSPH1* | 3.740346697 | 1.64E-183 | 5.36E-181 |  |  |  |  |
| *SLC37A1* | 2.456997564 | 1.44E-132 | 2.08E-130 |  |  |  |  |
| *TFF1* | 8.0802826 | 7.84E-175 | 2.18E-172 |  |  |  |  |
| *TFF2* | 9.03952958 | 1.40E-173 | 3.71E-171 |  |  |  |  |
| *TFF3* | 4.386353092 | 2.04E-71 | 8.35E-70 |  |  |  |  |
| *TIAM1* | -2.208597906 | 2.18E-47 | 4.60E-46 |  |  |  |  |
| *TMPRSS15* | 3.027859891 | 7.19E-18 | 4.39E-17 |  |  |  |  |
| *TMPRSS2* | 3.683543448 | 2.13E-122 | 2.49E-120 |  |  |  |  |
| *TMPRSS3* | 4.530119126 | 1.36E-153 | 2.73E-151 |  |  |  |  |
| *U3.6* | 3.25737866 | 1.49E-27 | 1.52E-26 |  |  |  |  |
| *AK097787* | 3.053126939 | 7.76E-20 | 5.31E-19 |  |  |  |  |
| *AK131325* | 2.171094251 | 1.38E-15 | 7.34E-15 |  |  |  |  |
| *BAIAP2L2* | 2.294759569 | 3.98E-71 | 1.61E-69 |  |  |  |  |
| *BC035867* | -3.321831016 | 1.03E-17 | 6.23E-17 |  |  |  |  |
| *BC038197* | 2.288393327 | 4.76E-06 | 1.22E-05 |  |  |  |  |
| *BC089413* | 2.512000767 | 1.56E-18 | 9.84E-18 |  |  |  |  |
| *BPIFC* | -3.529812644 | 3.31E-22 | 2.56E-21 |  |  |  |  |
| *CECR2* | -3.196309764 | 5.26E-52 | 1.28E-50 |  |  |  |  |
| *CELSR1* | -2.353396813 | 1.78E-79 | 8.66E-78 |  |  |  |  |
| *CR936633* | 3.816674512 | 7.62E-20 | 5.22E-19 |  |  |  |  |
| *DQ571461.1* | -2.316551405 | 3.00E-05 | 7.08E-05 |  |  |  |  |
| *FLJ46257* | -3.124710222 | 7.73E-20 | 5.30E-19 |  |  |  |  |
| *GAL3ST1* | 5.116809989 | 1.02E-243 | 6.69E-241 |  |  |  |  |
| *GGT2* | -3.36299121 | 7.36E-11 | 2.85E-10 |  |  |  |  |
| *GSC2* | -3.313514822 | 9.85E-14 | 4.69E-13 |  |  |  |  |
| *GSTTP2* | 2.16629212 | 7.37E-09 | 2.44E-08 |  |  |  |  |
| *IL17REL* | 2.856143295 | 5.83E-33 | 7.46E-32 |  |  |  |  |
| *ISX* | 7.148757193 | 1.48E-78 | 7.05E-77 |  |  |  |  |
| *KLHDC7B* | -3.166853412 | 3.16E-54 | 8.21E-53 |  |  |  |  |
| *LGALS2* | 4.325927838 | 3.59E-91 | 2.20E-89 |  |  |  |  |
| *LOC284865* | 3.656944951 | 8.42E-43 | 1.53E-41 |  |  |  |  |
| *MGAT3* | 2.43800778 | 4.31E-64 | 1.46E-62 |  |  |  |  |
| *MIR650* | 2.513393938 | 3.78E-24 | 3.26E-23 |  |  |  |  |
| *MPPED1* | -3.925511592 | 1.61E-36 | 2.37E-35 |  |  |  |  |
| *PANX2* | -3.631707641 | 5.07E-70 | 1.99E-68 |  |  |  |  |
| *POM121L4P* | -2.343480344 | 5.62E-07 | 1.57E-06 |  |  |  |  |
| *SGSM1* | 2.004964515 | 4.94E-36 | 7.09E-35 |  |  |  |  |
| *SLC5A1* | 2.143174239 | 6.02E-31 | 7.12E-30 |  |  |  |  |
| *SNORA50.2* | 2.190720715 | 6.69E-11 | 2.60E-10 |  |  |  |  |
| *TBX1* | -2.842395741 | 3.39E-44 | 6.48E-43 |  |  |  |  |
| *UPK3A* | 4.520475907 | 3.29E-59 | 9.87E-58 |  |  |  |  |
| *WNT7B* | -3.34105523 | 7.04E-50 | 1.61E-48 |  |  |  |  |
| *A4GNT* | 4.954098748 | 8.23E-59 | 2.44E-57 |  |  |  |  |
| *AADACL2* | -4.602502187 | 8.83E-52 | 2.14E-50 |  |  |  |  |
| *ABCC5* | -2.831177407 | 3.11E-164 | 7.50E-162 |  |  |  |  |
| *ADIPOQ* | 4.682619354 | 1.29E-25 | 1.19E-24 |  |  |  |  |
| *AF279780* | -2.074140021 | 7.88E-18 | 4.81E-17 |  |  |  |  |
| *AK056252* | -2.346801806 | 4.73E-26 | 4.47E-25 |  |  |  |  |
| *AK091265* | 5.325925097 | 9.83E-195 | 3.81E-192 |  |  |  |  |
| *AK092143* | -2.618005779 | 8.54E-26 | 7.96E-25 |  |  |  |  |
| *AK092619* | -3.512145842 | 4.08E-47 | 8.52E-46 |  |  |  |  |
| *AK124973* | -3.337885976 | 9.94E-101 | 7.35E-99 |  |  |  |  |
| *AK128202* | -2.76781468 | 6.19E-15 | 3.17E-14 |  |  |  |  |
| *AK304483* | -2.938603197 | 6.72E-18 | 4.11E-17 |  |  |  |  |
| *ANKUB1* | -2.868703361 | 4.85E-15 | 2.50E-14 |  |  |  |  |
| *APOD* | 2.622995801 | 2.43E-30 | 2.79E-29 |  |  |  |  |
| *ARHGEF26-AS1* | -2.115169125 | 1.53E-18 | 9.68E-18 |  |  |  |  |
| *ARL14* | 3.632854702 | 9.90E-74 | 4.21E-72 |  |  |  |  |
| *ATP13A5* | -5.618563211 | 8.67E-76 | 3.85E-74 |  |  |  |  |
| *AX746877.1* | -3.748201024 | 3.21E-49 | 7.17E-48 |  |  |  |  |
| *AX746877.2* | -3.544343948 | 2.83E-55 | 7.64E-54 |  |  |  |  |
| *BC036236* | -3.319082442 | 2.46E-24 | 2.14E-23 |  |  |  |  |
| *BC038725* | 2.276401687 | 2.73E-08 | 8.62E-08 |  |  |  |  |
| *C3orf32* | 2.426723448 | 1.87E-44 | 3.63E-43 |  |  |  |  |
| *C3orf72* | -3.137066536 | 7.76E-27 | 7.59E-26 |  |  |  |  |
| *C3orf74* | -4.282684706 | 2.95E-55 | 7.93E-54 |  |  |  |  |
| *C3orf79* | -3.153638766 | 6.34E-23 | 5.12E-22 |  |  |  |  |
| *CACNA1D* | 2.53761768 | 4.73E-63 | 1.58E-61 |  |  |  |  |
| *CADPS* | 4.527459761 | 1.38E-82 | 7.15E-81 |  |  |  |  |
| *CAMKV* | 2.419803056 | 1.08E-18 | 6.87E-18 |  |  |  |  |
| *CCK* | 3.248976474 | 2.40E-33 | 3.13E-32 |  |  |  |  |
| *CCR2* | 2.088594885 | 4.27E-35 | 5.93E-34 |  |  |  |  |
| *CCR9* | 2.593591395 | 4.10E-40 | 6.83E-39 |  |  |  |  |
| *CCRL1* | 2.006281551 | 2.00E-35 | 2.81E-34 |  |  |  |  |
| *CCRL2* | 2.560213549 | 9.05E-122 | 1.04E-119 |  |  |  |  |
| *CHDH* | 3.277367181 | 2.75E-175 | 7.71E-173 |  |  |  |  |
| *CHST13* | 3.470384083 | 4.43E-58 | 1.28E-56 |  |  |  |  |
| *CHST2* | -2.091449153 | 3.43E-54 | 8.92E-53 |  |  |  |  |
| *CIDEC* | 2.29501155 | 5.13E-59 | 1.53E-57 |  |  |  |  |
| *CLDN18* | 8.778892166 | 1.73E-279 | 1.59E-276 |  |  |  |  |
| *CLDN1* | -2.085941132 | 3.13E-45 | 6.19E-44 |  |  |  |  |
| *COL7A1* | -2.905270346 | 8.92E-70 | 3.47E-68 |  |  |  |  |
| *CPB1* | 2.788094119 | 1.01E-16 | 5.76E-16 |  |  |  |  |
| *CSTA* | -2.841063886 | 6.80E-22 | 5.19E-21 |  |  |  |  |
| *DQ571917* | -4.21708873 | 5.57E-21 | 4.06E-20 |  |  |  |  |
| *DUSP7* | -2.010600139 | 5.14E-110 | 4.51E-108 |  |  |  |  |
| *ESRG* | -7.68505488 | 1.85E-122 | 2.16E-120 |  |  |  |  |
| *FAM19A4* | 2.424083161 | 1.35E-11 | 5.52E-11 |  |  |  |  |
| *FAM3D* | 3.016333217 | 9.64E-41 | 1.65E-39 |  |  |  |  |
| *FETUB* | -5.470386673 | 3.77E-43 | 6.93E-42 |  |  |  |  |
| *FLJ22763* | 4.83139082 | 1.26E-41 | 2.19E-40 |  |  |  |  |
| *FOXL2* | -3.75487637 | 4.45E-38 | 6.91E-37 |  |  |  |  |
| *GHRL* | 2.473347978 | 2.62E-35 | 3.65E-34 |  |  |  |  |
| *GLYCTK* | 2.640430061 | 1.67E-151 | 3.24E-149 |  |  |  |  |
| *GP9* | 2.009711975 | 8.97E-12 | 3.72E-11 |  |  |  |  |
| *GPR128* | 5.964407165 | 8.97E-126 | 1.14E-123 |  |  |  |  |
| *GPR149* | -5.33848938 | 1.90E-28 | 2.02E-27 |  |  |  |  |
| *GPR156* | -2.700674078 | 3.39E-43 | 6.24E-42 |  |  |  |  |
| *GPR160* | 2.730956171 | 1.05E-100 | 7.78E-99 |  |  |  |  |
| *GPR87* | -4.348344487 | 3.86E-39 | 6.18E-38 |  |  |  |  |
| *GRK7* | -2.428650896 | 4.56E-46 | 9.22E-45 |  |  |  |  |
| *HGD* | 5.457793868 | 2.04E-189 | 7.38E-187 |  |  |  |  |
| *HHLA2* | 6.112025455 | 2.11E-169 | 5.42E-167 |  |  |  |  |
| *HRG* | -2.174492951 | 1.59E-11 | 6.46E-11 |  |  |  |  |
| *IGSF11* | -2.990608731 | 6.77E-45 | 1.33E-43 |  |  |  |  |
| *IL17RB* | 2.979902708 | 2.36E-129 | 3.26E-127 |  |  |  |  |
| *IL1RAP* | -2.425150768 | 3.73E-111 | 3.40E-109 |  |  |  |  |
| *IL20RB* | -3.970430797 | 4.83E-80 | 2.39E-78 |  |  |  |  |
| *ITIH1* | 2.950021137 | 2.10E-30 | 2.42E-29 |  |  |  |  |
| *JA611300* | -2.064253977 | 0.000360352 | 0.000754082 |  |  |  |  |
| *KBTBD12* | 3.411447193 | 1.37E-47 | 2.91E-46 |  |  |  |  |
| *KNG1* | 2.261237671 | 2.66E-20 | 1.87E-19 |  |  |  |  |
| *LEPREL1* | -2.301272968 | 5.72E-43 | 1.04E-41 |  |  |  |  |
| *LIPH* | 2.487575778 | 5.82E-55 | 1.55E-53 |  |  |  |  |
| *LOC201617* | 2.929942016 | 2.32E-49 | 5.22E-48 |  |  |  |  |
| *LOC344887* | -5.798681826 | 3.57E-163 | 8.39E-161 |  |  |  |  |
| *LOC401109* | -4.71994411 | 3.52E-92 | 2.20E-90 |  |  |  |  |
| *LRRC2* | 2.729082581 | 4.44E-52 | 1.08E-50 |  |  |  |  |
| *LRRC31* | 5.636046186 | 1.32E-144 | 2.29E-142 |  |  |  |  |
| *LRRIQ4* | 2.378626211 | 2.14E-39 | 3.48E-38 |  |  |  |  |
| *LTF* | 2.685462905 | 8.96E-21 | 6.46E-20 |  |  |  |  |
| *MECOM* | 2.282801228 | 9.12E-78 | 4.24E-76 |  |  |  |  |
| *MIR135A1* | 2.291781073 | 7.56E-47 | 1.57E-45 |  |  |  |  |
| *MIR4273.1* | 2.309627892 | 0.00016724 | 0.000363269 |  |  |  |  |
| *MIR944* | -5.22287521 | 3.38E-75 | 1.48E-73 |  |  |  |  |
| *MUC13* | 6.099036634 | 1.17E-190 | 4.33E-188 |  |  |  |  |
| *MYRIP* | 2.166500227 | 6.49E-32 | 8.02E-31 |  |  |  |  |
| *Mir_384.4* | -3.410035561 | 1.80E-12 | 7.87E-12 |  |  |  |  |
| *Mir_584.29* | 2.615248903 | 2.19E-05 | 5.23E-05 |  |  |  |  |
| *NR1I2* | 6.596773002 | 4.18E-189 | 1.49E-186 |  |  |  |  |
| *P2RY1* | -3.206026544 | 5.08E-78 | 2.39E-76 |  |  |  |  |
| *PHLDB2* | -2.111553224 | 2.30E-49 | 5.17E-48 |  |  |  |  |
| *PLA1A* | 2.929306616 | 1.46E-70 | 5.86E-69 |  |  |  |  |
| *PLCH1* | 2.224071185 | 1.26E-66 | 4.50E-65 |  |  |  |  |
| *PLS1* | 2.783695194 | 7.02E-134 | 1.05E-131 |  |  |  |  |
| *PPARG* | 2.933039091 | 1.87E-108 | 1.62E-106 |  |  |  |  |
| *PVRL3* | 2.20194896 | 3.42E-65 | 1.18E-63 |  |  |  |  |
| *PYDC2* | -2.348307492 | 2.17E-05 | 5.20E-05 |  |  |  |  |
| *RBP2* | 6.485169719 | 1.23E-94 | 8.09E-93 |  |  |  |  |
| *RETNLB* | 2.963757022 | 3.56E-22 | 2.75E-21 |  |  |  |  |
| *RN7SK.71* | -2.101502999 | 5.27E-33 | 6.76E-32 |  |  |  |  |
| *ROPN1* | -2.351131834 | 2.33E-10 | 8.71E-10 |  |  |  |  |
| *RTP1* | -2.544110592 | 2.48E-13 | 1.15E-12 |  |  |  |  |
| *RTP3* | -3.453871498 | 2.05E-15 | 1.08E-14 |  |  |  |  |
| *SEMA3B* | 2.353123483 | 4.16E-70 | 1.64E-68 |  |  |  |  |
| *SI* | 9.3785898 | 8.00E-175 | 2.20E-172 |  |  |  |  |
| *SLC2A2* | 4.133016041 | 9.90E-38 | 1.52E-36 |  |  |  |  |
| *SLC38A3* | 2.039309251 | 6.39E-15 | 3.27E-14 |  |  |  |  |
| *SLC6A11* | -4.020713913 | 6.96E-30 | 7.83E-29 |  |  |  |  |
| *SLC6A20* | 5.567966248 | 2.61E-166 | 6.47E-164 |  |  |  |  |
| *SOX14* | 4.059084432 | 2.08E-27 | 2.09E-26 |  |  |  |  |
| *SOX2-OT* | -4.020628644 | 1.99E-99 | 1.43E-97 |  |  |  |  |
| *SOX2* | -2.847894963 | 1.81E-27 | 1.83E-26 |  |  |  |  |
| *SPSB4* | -2.909864903 | 1.35E-58 | 3.97E-57 |  |  |  |  |
| *SST* | 5.762209224 | 3.85E-59 | 1.15E-57 |  |  |  |  |
| *SYNPR* | 5.013278336 | 6.28E-60 | 1.95E-58 |  |  |  |  |
| *TDGF1* | 5.777821284 | 8.64E-108 | 7.35E-106 |  |  |  |  |
| *TGM4* | -2.985943718 | 6.44E-42 | 1.13E-40 |  |  |  |  |
| *TM4SF19* | -3.125026247 | 2.25E-121 | 2.53E-119 |  |  |  |  |
| *TM4SF4* | 8.101318189 | 1.58E-172 | 4.13E-170 |  |  |  |  |
| *TMEM207* | 2.133401544 | 0.000207097 | 0.000445167 |  |  |  |  |
| *TMEM40* | -2.952834585 | 2.14E-19 | 1.43E-18 |  |  |  |  |
| *TMEM45A* | -2.918599084 | 1.93E-87 | 1.09E-85 |  |  |  |  |
| *TNNC1* | 4.977967247 | 1.10E-138 | 1.77E-136 |  |  |  |  |
| *TP63* | -6.486372301 | 1.89E-128 | 2.54E-126 |  |  |  |  |
| *TPRG1* | -2.474310768 | 1.02E-36 | 1.52E-35 |  |  |  |  |
| *TRIM71* | 2.286541588 | 4.84E-11 | 1.90E-10 |  |  |  |  |
| *U6.110* | -2.16678566 | 1.34E-13 | 6.33E-13 |  |  |  |  |
| *UCN2* | -2.719942829 | 6.38E-46 | 1.29E-44 |  |  |  |  |
| *UPK1B* | -2.368633344 | 7.63E-13 | 3.41E-12 |  |  |  |  |
| *VILL* | 3.613703729 | 2.88E-148 | 5.31E-146 |  |  |  |  |
| *WNT7A* | -3.902537918 | 1.51E-31 | 1.84E-30 |  |  |  |  |
| *ZBED2* | -3.019920893 | 4.41E-51 | 1.05E-49 |  |  |  |  |
| *ZIC4* | -2.048666124 | 2.57E-06 | 6.76E-06 |  |  |  |  |
| *AB059369* | 3.758089247 | 2.03E-32 | 2.56E-31 |  |  |  |  |
| *ADH1B* | 2.324255636 | 1.09E-18 | 6.93E-18 |  |  |  |  |
| *ADH1C* | 2.50064789 | 1.33E-19 | 8.99E-19 |  |  |  |  |
| *ADH4* | 5.552414455 | 2.68E-85 | 1.46E-83 |  |  |  |  |
| *ADH6* | 4.447129457 | 1.81E-97 | 1.26E-95 |  |  |  |  |
| *ADH7* | -4.56024279 | 4.87E-23 | 3.96E-22 |  |  |  |  |
| *AFM* | 2.05744948 | 3.29E-05 | 7.74E-05 |  |  |  |  |
| *AFP* | 2.420617672 | 2.75E-14 | 1.35E-13 |  |  |  |  |
| *AGXT2L1* | 2.533942114 | 1.16E-13 | 5.51E-13 |  |  |  |  |
| *AK001394* | -2.041321879 | 2.18E-43 | 4.04E-42 |  |  |  |  |
| *AK026379* | 2.073695681 | 8.19E-50 | 1.87E-48 |  |  |  |  |
| *AK093264* | 2.210135843 | 7.70E-07 | 2.13E-06 |  |  |  |  |
| *AK095285* | -2.222447816 | 3.58E-10 | 1.32E-09 |  |  |  |  |
| *AK124272* | 3.507558697 | 1.76E-23 | 1.46E-22 |  |  |  |  |
| *ALB* | 3.733324284 | 2.93E-37 | 4.43E-36 |  |  |  |  |
| *AMTN* | -4.601002142 | 1.14E-27 | 1.16E-26 |  |  |  |  |
| *ANXA10* | 3.305571893 | 4.32E-32 | 5.38E-31 |  |  |  |  |
| *ARHGEF38* | 4.964049075 | 4.38E-248 | 2.94E-245 |  |  |  |  |
| *ATOH1* | 8.259175217 | 3.03E-123 | 3.65E-121 |  |  |  |  |
| *AX748249* | 3.165669784 | 2.99E-53 | 7.55E-52 |  |  |  |  |
| *BC016361* | -3.234282723 | 1.26E-34 | 1.73E-33 |  |  |  |  |
| *BC031092* | -2.941869158 | 1.83E-19 | 1.23E-18 |  |  |  |  |
| *BC035172* | -2.449099459 | 7.89E-09 | 2.61E-08 |  |  |  |  |
| *BC041902* | -2.409871121 | 2.95E-11 | 1.18E-10 |  |  |  |  |
| *BC042823* | 2.582961907 | 4.85E-44 | 9.22E-43 |  |  |  |  |
| *BC070495* | -3.599684548 | 3.88E-37 | 5.84E-36 |  |  |  |  |
| *C1QTNF7* | 2.12371225 | 1.01E-26 | 9.86E-26 |  |  |  |  |
| *C4orf17* | -3.148387721 | 1.65E-12 | 7.23E-12 |  |  |  |  |
| *C4orf19* | 3.192323241 | 4.49E-111 | 4.07E-109 |  |  |  |  |
| *CCKAR* | 3.007008524 | 3.46E-15 | 1.80E-14 |  |  |  |  |
| *CHRNA9* | -4.864640036 | 3.41E-70 | 1.35E-68 |  |  |  |  |
| *CLDN24* | -3.281191387 | 5.12E-48 | 1.10E-46 |  |  |  |  |
| *CPLX1* | 2.139064939 | 1.19E-51 | 2.88E-50 |  |  |  |  |
| *CSN1S1* | 2.283505233 | 0.000301241 | 0.000635511 |  |  |  |  |
| *CWH43* | -2.792154066 | 1.13E-09 | 4.00E-09 |  |  |  |  |
| *CXCL2* | 2.105652854 | 7.25E-36 | 1.03E-34 |  |  |  |  |
| *CXCL3* | 3.545005258 | 8.03E-92 | 4.98E-90 |  |  |  |  |
| *CXCL5* | 4.014753673 | 1.14E-45 | 2.28E-44 |  |  |  |  |
| *DMP1* | -2.120240701 | 9.18E-17 | 5.26E-16 |  |  |  |  |
| *DOK7* | 2.161624961 | 9.54E-38 | 1.47E-36 |  |  |  |  |
| *DQ583161.2* | 2.411925671 | 9.19E-06 | 2.29E-05 |  |  |  |  |
| *DQ590589.20* | 2.814241363 | 7.08E-06 | 1.78E-05 |  |  |  |  |
| *DRD5* | -2.181829008 | 4.62E-19 | 3.02E-18 |  |  |  |  |
| *DSPP* | -3.130156077 | 3.66E-27 | 3.64E-26 |  |  |  |  |
| *ENAM* | 2.514183515 | 4.64E-24 | 3.98E-23 |  |  |  |  |
| *EPGN* | -2.742227238 | 1.07E-11 | 4.41E-11 |  |  |  |  |
| *F11* | 3.601851671 | 3.04E-30 | 3.47E-29 |  |  |  |  |
| *FABP2* | 7.594776325 | 5.96E-120 | 6.45E-118 |  |  |  |  |
| *FGA* | 4.955563469 | 1.95E-44 | 3.78E-43 |  |  |  |  |
| *FGB* | 4.014333142 | 2.00E-27 | 2.01E-26 |  |  |  |  |
| *FGFBP1* | -3.552594088 | 3.02E-36 | 4.37E-35 |  |  |  |  |
| *FGFBP2* | -6.049379695 | 1.08E-166 | 2.70E-164 |  |  |  |  |
| *FGG* | 3.208015967 | 4.83E-15 | 2.49E-14 |  |  |  |  |
| *FLJ35424* | 4.769803681 | 6.75E-27 | 6.62E-26 |  |  |  |  |
| *FRG2.1* | -2.482614553 | 4.41E-05 | 0.000102206 |  |  |  |  |
| *FTLP10* | -2.283630114 | 0.000349751 | 0.00073279 |  |  |  |  |
| *GABRA2* | 2.813043164 | 3.27E-28 | 3.42E-27 |  |  |  |  |
| *GABRA4* | 2.131134255 | 1.55E-12 | 6.78E-12 |  |  |  |  |
| *GALNTL6* | 3.035889609 | 2.32E-29 | 2.55E-28 |  |  |  |  |
| *GBA3* | 4.991488278 | 8.43E-64 | 2.85E-62 |  |  |  |  |
| *GC* | 8.059497392 | 2.50E-116 | 2.55E-114 |  |  |  |  |
| *GLRA3* | -2.077327917 | 1.31E-13 | 6.17E-13 |  |  |  |  |
| *GPM6A* | -2.28412682 | 1.49E-17 | 8.96E-17 |  |  |  |  |
| *GPR78* | -2.228309562 | 2.23E-08 | 7.09E-08 |  |  |  |  |
| *GPRIN3* | 2.456237805 | 1.76E-64 | 6.01E-63 |  |  |  |  |
| *HAND2* | 2.216070863 | 1.90E-13 | 8.89E-13 |  |  |  |  |
| *HMX1* | -3.794374458 | 3.13E-15 | 1.63E-14 |  |  |  |  |
| *HPGD* | 2.004712856 | 8.64E-28 | 8.89E-27 |  |  |  |  |
| *IGJ* | 3.662086806 | 2.96E-60 | 9.30E-59 |  |  |  |  |
| *KIAA1211* | 2.412380372 | 1.83E-97 | 1.27E-95 |  |  |  |  |
| *LOC100505989* | 5.801481192 | 7.70E-123 | 9.04E-121 |  |  |  |  |
| *LOC100507096* | 2.978671438 | 1.73E-06 | 4.61E-06 |  |  |  |  |
| *LOC285419* | 2.685900363 | 2.25E-84 | 1.21E-82 |  |  |  |  |
| *LOC285547* | 2.870066137 | 2.23E-07 | 6.48E-07 |  |  |  |  |
| *LOC402160* | 2.03321161 | 3.09E-33 | 4.01E-32 |  |  |  |  |
| *LOC644145* | 2.268152141 | 6.28E-08 | 1.92E-07 |  |  |  |  |
| *LRAT* | -3.920880984 | 6.22E-48 | 1.33E-46 |  |  |  |  |
| *LRRC66* | 4.931095822 | 1.68E-168 | 4.27E-166 |  |  |  |  |
| *MGC45800* | -2.169440955 | 1.43E-18 | 9.05E-18 |  |  |  |  |
| *MGC4836* | -3.502841508 | 1.24E-40 | 2.10E-39 |  |  |  |  |
| *MIR548I2* | 3.684173435 | 5.51E-41 | 9.44E-40 |  |  |  |  |
| *MTNR1A* | 2.307417771 | 2.48E-19 | 1.65E-18 |  |  |  |  |
| *MTTP* | 5.182134637 | 1.27E-69 | 4.89E-68 |  |  |  |  |
| *NBLA00301* | 2.544348943 | 1.88E-16 | 1.06E-15 |  |  |  |  |
| *NKX6-1* | -2.200650938 | 2.69E-14 | 1.32E-13 |  |  |  |  |
| *NPY2R* | 2.089278909 | 3.84E-05 | 8.96E-05 |  |  |  |  |
| *NR3C2* | 2.106659511 | 2.50E-49 | 5.60E-48 |  |  |  |  |
| *ODAM* | 4.390402585 | 1.60E-62 | 5.30E-61 |  |  |  |  |
| *PF4* | 4.475068126 | 1.01E-69 | 3.90E-68 |  |  |  |  |
| *PITX2* | -2.887208589 | 2.06E-32 | 2.60E-31 |  |  |  |  |
| *PLAC8* | 2.480639034 | 5.57E-40 | 9.25E-39 |  |  |  |  |
| *PPP2R2C* | -3.44317125 | 2.51E-39 | 4.05E-38 |  |  |  |  |
| *PROM1* | 4.391222895 | 1.33E-104 | 1.08E-102 |  |  |  |  |
| *PSAPL1* | 2.487443204 | 2.64E-17 | 1.56E-16 |  |  |  |  |
| *PTPN13* | -3.006028478 | 3.14E-74 | 1.35E-72 |  |  |  |  |
| *QRFPR* | -5.40822751 | 2.88E-87 | 1.61E-85 |  |  |  |  |
| *RN7SK.87* | 2.169902213 | 0.000511053 | 0.001050572 |  |  |  |  |
| *S100P* | 2.648038261 | 1.07E-41 | 1.88E-40 |  |  |  |  |
| *SCRG1* | 2.412258857 | 4.84E-17 | 2.82E-16 |  |  |  |  |
| *SHISA3* | 2.525606374 | 3.14E-26 | 3.00E-25 |  |  |  |  |
| *SHROOM3* | 2.183942602 | 9.66E-85 | 5.21E-83 |  |  |  |  |
| *SLC10A6* | -2.861376792 | 2.05E-52 | 5.06E-51 |  |  |  |  |
| *SLC4A4* | 5.091948174 | 6.02E-161 | 1.37E-158 |  |  |  |  |
| *SLC7A11* | -2.052943036 | 3.00E-35 | 4.18E-34 |  |  |  |  |
| *SPOCK3* | -3.429265563 | 6.74E-28 | 6.96E-27 |  |  |  |  |
| *STATH* | -2.157491786 | 1.48E-05 | 3.62E-05 |  |  |  |  |
| *SULT1B1* | 4.097774895 | 5.74E-81 | 2.87E-79 |  |  |  |  |
| *SYT14L* | -2.153308297 | 5.78E-10 | 2.09E-09 |  |  |  |  |
| *TMEM154* | -2.543632797 | 3.77E-66 | 1.33E-64 |  |  |  |  |
| *TMPRSS11A* | -2.524164361 | 9.23E-07 | 2.53E-06 |  |  |  |  |
| *TMPRSS11BNL* | -2.495083041 | 2.67E-05 | 6.32E-05 |  |  |  |  |
| *TMPRSS11D* | -3.263595629 | 6.46E-15 | 3.30E-14 |  |  |  |  |
| *TMPRSS11E* | -2.765137246 | 8.82E-11 | 3.40E-10 |  |  |  |  |
| *TMPRSS11F* | -2.695776989 | 1.81E-08 | 5.79E-08 |  |  |  |  |
| *U6.122* | 2.701754249 | 4.22E-10 | 1.54E-09 |  |  |  |  |
| *UCHL1* | -3.464805695 | 2.03E-57 | 5.80E-56 |  |  |  |  |
| *UGT2A1* | -2.113921341 | 6.13E-11 | 2.39E-10 |  |  |  |  |
| *UGT2A3* | 8.160249786 | 3.60E-140 | 5.82E-138 |  |  |  |  |
| *UGT2B10.1* | 3.580458939 | 5.76E-12 | 2.42E-11 |  |  |  |  |
| *UGT2B11* | 3.060444166 | 1.40E-18 | 8.90E-18 |  |  |  |  |
| *UGT2B15* | 6.503466618 | 1.74E-121 | 1.97E-119 |  |  |  |  |
| *UGT2B17* | 3.503918479 | 3.66E-24 | 3.16E-23 |  |  |  |  |
| *UGT2B7* | 5.595499249 | 6.31E-108 | 5.38E-106 |  |  |  |  |
| *AB074188* | 2.577610264 | 2.46E-30 | 2.82E-29 |  |  |  |  |
| *ADRB2* | -2.37100167 | 7.96E-45 | 1.56E-43 |  |  |  |  |
| *ARSI* | -2.29803278 | 9.25E-44 | 1.74E-42 |  |  |  |  |
| *ATP10B* | 2.586275121 | 2.46E-40 | 4.14E-39 |  |  |  |  |
| *AX746723* | -2.056763934 | 1.05E-05 | 2.59E-05 |  |  |  |  |
| *AX747345* | -3.634216646 | 1.12E-20 | 8.02E-20 |  |  |  |  |
| *BC032469* | 5.440842888 | 1.06E-69 | 4.09E-68 |  |  |  |  |
| *BC032795* | 3.587685004 | 1.68E-110 | 1.50E-108 |  |  |  |  |
| *BC034612* | 2.491302338 | 3.45E-14 | 1.68E-13 |  |  |  |  |
| *BC034636* | -5.502749022 | 7.88E-79 | 3.78E-77 |  |  |  |  |
| *BC038535* | -4.009172086 | 8.23E-29 | 8.85E-28 |  |  |  |  |
| *BC042046* | -2.07129415 | 7.71E-14 | 3.69E-13 |  |  |  |  |
| *BC127870* | 2.216950501 | 4.46E-46 | 9.02E-45 |  |  |  |  |
| *BTNL3* | 6.561477663 | 2.52E-133 | 3.69E-131 |  |  |  |  |
| *BTNL8* | 7.118700576 | 7.16E-240 | 4.47E-237 |  |  |  |  |
| *C5orf52* | 3.563598391 | 1.49E-37 | 2.28E-36 |  |  |  |  |
| *C6* | 4.680600088 | 7.98E-70 | 3.11E-68 |  |  |  |  |
| *C7* | 2.644640339 | 1.79E-21 | 1.33E-20 |  |  |  |  |
| *C9* | -2.385143846 | 2.85E-20 | 2.00E-19 |  |  |  |  |
| *CARTPT* | 2.853992879 | 4.27E-11 | 1.68E-10 |  |  |  |  |
| *CCL28* | 2.084441165 | 9.76E-33 | 1.24E-31 |  |  |  |  |
| *CCNI2* | 3.215755985 | 1.41E-159 | 3.12E-157 |  |  |  |  |
| *CDH18* | -2.438732896 | 3.96E-10 | 1.45E-09 |  |  |  |  |
| *CDHR2* | 6.195728108 | 2.41E-207 | 1.03E-204 |  |  |  |  |
| *CDX1* | 5.511816368 | 1.15E-97 | 8.05E-96 |  |  |  |  |
| *CTNND2* | 3.549203775 | 1.30E-39 | 2.13E-38 |  |  |  |  |
| *CTXN3* | -2.892858351 | 1.08E-14 | 5.45E-14 |  |  |  |  |
| *CXCL14* | -2.194128676 | 1.09E-31 | 1.33E-30 |  |  |  |  |
| *CYSTM1* | 3.46521087 | 1.01E-163 | 2.42E-161 |  |  |  |  |
| *DL490294* | 2.890161905 | 3.85E-07 | 1.09E-06 |  |  |  |  |
| *DQ576909* | 2.026368146 | 2.54E-07 | 7.32E-07 |  |  |  |  |
| *DRD1* | 2.87577308 | 7.51E-30 | 8.43E-29 |  |  |  |  |
| *ENC1* | 2.495073553 | 8.81E-131 | 1.24E-128 |  |  |  |  |
| *FAM134B* | 2.098547477 | 2.04E-45 | 4.05E-44 |  |  |  |  |
| *FAT2* | -5.365767315 | 1.15E-82 | 6.01E-81 |  |  |  |  |
| *FBN2* | -2.775274724 | 1.67E-66 | 5.96E-65 |  |  |  |  |
| *FBXL21* | -2.439486176 | 3.23E-08 | 1.01E-07 |  |  |  |  |
| *FGF10* | 3.096721978 | 7.80E-39 | 1.24E-37 |  |  |  |  |
| *FGF18* | 2.523508337 | 3.89E-54 | 1.01E-52 |  |  |  |  |
| *FGFR4* | 3.498403453 | 1.82E-143 | 3.05E-141 |  |  |  |  |
| *FSTL4* | -2.472757935 | 5.34E-18 | 3.28E-17 |  |  |  |  |
| *FST* | -3.008054576 | 6.25E-50 | 1.43E-48 |  |  |  |  |
| *GAPT* | 2.076151237 | 9.70E-29 | 1.04E-27 |  |  |  |  |
| *GHR.2* | -2.040504923 | 3.76E-07 | 1.07E-06 |  |  |  |  |
| *GPR98* | -2.175636967 | 3.64E-22 | 2.81E-21 |  |  |  |  |
| *HAVCR1* | 5.037085998 | 4.57E-67 | 1.66E-65 |  |  |  |  |
| *HMP19* | 2.231683183 | 1.69E-17 | 1.01E-16 |  |  |  |  |
| *HSPB3* | -2.459373999 | 1.62E-13 | 7.62E-13 |  |  |  |  |
| *HTR4* | 3.055325154 | 3.91E-24 | 3.37E-23 |  |  |  |  |
| *IL31RA* | -2.486157481 | 1.57E-30 | 1.82E-29 |  |  |  |  |
| *IQGAP2* | 3.037292595 | 2.63E-120 | 2.89E-118 |  |  |  |  |
| *IRX1* | -5.020473232 | 2.82E-57 | 8.02E-56 |  |  |  |  |
| *IRX4* | -5.869087474 | 3.48E-52 | 8.53E-51 |  |  |  |  |
| *ISL1* | 3.016392298 | 3.84E-49 | 8.54E-48 |  |  |  |  |
| *LOC340074* | -2.524749454 | 2.97E-08 | 9.35E-08 |  |  |  |  |
| *LOC389332* | 5.20423135 | 7.32E-94 | 4.77E-92 |  |  |  |  |
| *LOC643201* | 2.475295651 | 2.53E-31 | 3.04E-30 |  |  |  |  |
| *MCC* | -2.466456986 | 8.76E-96 | 5.90E-94 |  |  |  |  |
| *MIR4277* | -2.395299098 | 1.63E-07 | 4.78E-07 |  |  |  |  |
| *MIR4456* | 2.012591291 | 0.023360557 | 0.037760053 |  |  |  |  |
| *MIR4803* | -2.196558161 | 1.33E-06 | 3.61E-06 |  |  |  |  |
| *MZB1* | 2.029715967 | 1.28E-24 | 1.13E-23 |  |  |  |  |
| *Metazoa_SRP.85* | 3.246040889 | 3.87E-39 | 6.20E-38 |  |  |  |  |
| *NEUROG1* | -2.770314438 | 6.83E-07 | 1.90E-06 |  |  |  |  |
| *NIPAL4* | -4.031515001 | 6.30E-66 | 2.21E-64 |  |  |  |  |
| *NME5* | 2.100177562 | 7.41E-34 | 9.90E-33 |  |  |  |  |
| *NMUR2* | 4.919494445 | 1.15E-57 | 3.31E-56 |  |  |  |  |
| *NPR3* | -2.700630361 | 4.33E-40 | 7.19E-39 |  |  |  |  |
| *NPY6R* | 2.360896092 | 1.49E-17 | 8.99E-17 |  |  |  |  |
| *ODZ2* | -5.573348156 | 1.09E-121 | 1.24E-119 |  |  |  |  |
| *PLCXD3* | 2.455978953 | 6.57E-23 | 5.30E-22 |  |  |  |  |
| *PLEKHG4B* | -2.763467966 | 4.61E-30 | 5.23E-29 |  |  |  |  |
| *RAB3C* | 3.299452749 | 1.11E-49 | 2.53E-48 |  |  |  |  |
| *RGS7BP* | 2.188425134 | 2.90E-35 | 4.03E-34 |  |  |  |  |
| *SLC12A2* | 2.35299863 | 1.13E-77 | 5.24E-76 |  |  |  |  |
| *SLC34A1* | -2.734899001 | 4.38E-33 | 5.64E-32 |  |  |  |  |
| *SLC6A18* | 2.775614956 | 4.21E-15 | 2.18E-14 |  |  |  |  |
| *SLC6A19* | 8.837249109 | 2.85E-180 | 8.92E-178 |  |  |  |  |
| *SLC6A7* | 2.887327702 | 1.35E-37 | 2.07E-36 |  |  |  |  |
| *SLCO6A1* | -2.242749661 | 4.53E-08 | 1.40E-07 |  |  |  |  |
| *SNCB* | -3.31000159 | 1.07E-42 | 1.93E-41 |  |  |  |  |
| *SNORA40.4* | -3.889838285 | 2.21E-23 | 1.83E-22 |  |  |  |  |
| *SNORA47* | 2.947267595 | 1.93E-33 | 2.53E-32 |  |  |  |  |
| *SOWAHA* | 3.944528381 | 4.73E-118 | 5.03E-116 |  |  |  |  |
| *SPINK13* | 2.098988089 | 3.34E-15 | 1.74E-14 |  |  |  |  |
| *SPINK1* | 8.997204261 | 0 | 0 |  |  |  |  |
| *SPINK5* | -2.266486108 | 2.02E-14 | 9.99E-14 |  |  |  |  |
| *SPINK6* | -5.819773762 | 8.05E-52 | 1.95E-50 |  |  |  |  |
| *THBS4* | 2.258600688 | 1.06E-16 | 6.04E-16 |  |  |  |  |
| *TLX3* | -4.289704031 | 1.42E-16 | 8.06E-16 |  |  |  |  |
| *TRPC7* | 2.65087598 | 1.41E-26 | 1.37E-25 |  |  |  |  |
| *TSLP* | -2.375250117 | 1.72E-44 | 3.35E-43 |  |  |  |  |
| *TSSK1B* | -2.333150663 | 1.80E-24 | 1.58E-23 |  |  |  |  |
| *UNC5A* | 2.849661566 | 3.76E-37 | 5.66E-36 |  |  |  |  |
| *AK024998* | 4.07384917 | 3.09E-90 | 1.85E-88 |  |  |  |  |
| *AK056584* | -3.607952964 | 2.05E-60 | 6.48E-59 |  |  |  |  |
| *AK123416* | 4.590498758 | 1.22E-56 | 3.42E-55 |  |  |  |  |
| *AK126334* | -6.079005083 | 9.13E-65 | 3.14E-63 |  |  |  |  |
| *ARG1* | -2.270886847 | 1.22E-40 | 2.07E-39 |  |  |  |  |
| *AX747250* | 2.115029201 | 3.08E-16 | 1.71E-15 |  |  |  |  |
| *BC040308* | -5.764839355 | 3.46E-103 | 2.73E-101 |  |  |  |  |
| *BC047626* | 2.301465849 | 2.26E-30 | 2.60E-29 |  |  |  |  |
| *BC067243* | 2.422542533 | 0.000150125 | 0.000327828 |  |  |  |  |
| *BC070061* | -3.287778054 | 6.84E-26 | 6.41E-25 |  |  |  |  |
| *BTN1A1* | 3.179177131 | 1.07E-48 | 2.35E-47 |  |  |  |  |
| *C6orf10* | -3.763091419 | 8.73E-32 | 1.07E-30 |  |  |  |  |
| *C6orf123* | 3.303541141 | 4.20E-83 | 2.23E-81 |  |  |  |  |
| *C6orf222* | 8.569021099 | 0 | 0 |  |  |  |  |
| *C6orf223* | 2.138318909 | 1.54E-23 | 1.28E-22 |  |  |  |  |
| *C6orf58* | 5.431083204 | 6.68E-58 | 1.93E-56 |  |  |  |  |
| *CAGE1* | -2.541658679 | 3.51E-55 | 9.42E-54 |  |  |  |  |
| *CD109* | -2.792045979 | 1.05E-75 | 4.65E-74 |  |  |  |  |
| *CDSN* | -4.277603456 | 7.49E-96 | 5.07E-94 |  |  |  |  |
| *CFB* | 2.474064953 | 3.66E-79 | 1.76E-77 |  |  |  |  |
| *CGA* | 2.466663842 | 1.58E-17 | 9.49E-17 |  |  |  |  |
| *CLDN20* | -2.539167813 | 2.08E-81 | 1.05E-79 |  |  |  |  |
| *CLIC5* | 3.473919915 | 2.43E-98 | 1.71E-96 |  |  |  |  |
| *CLPS* | 2.938005994 | 3.52E-18 | 2.18E-17 |  |  |  |  |
| *CLVS2* | 2.86989333 | 1.56E-09 | 5.45E-09 |  |  |  |  |
| *COL9A1* | 3.623093967 | 1.29E-68 | 4.91E-67 |  |  |  |  |
| *DCDC2* | 4.686425126 | 4.27E-88 | 2.44E-86 |  |  |  |  |
| *DLK2* | -2.678924794 | 1.05E-88 | 6.09E-87 |  |  |  |  |
| *DPCR1* | 7.485836122 | 1.96E-117 | 2.04E-115 |  |  |  |  |
| *DSP* | -2.501053746 | 1.33E-89 | 7.78E-88 |  |  |  |  |
| *DST* | -2.063592433 | 1.06E-69 | 4.12E-68 |  |  |  |  |
| *ELOVL4* | -2.40878303 | 2.48E-44 | 4.78E-43 |  |  |  |  |
| *FRMD1* | 2.095305642 | 9.11E-15 | 4.61E-14 |  |  |  |  |
| *FUT9* | 6.308081786 | 3.72E-89 | 2.17E-87 |  |  |  |  |
| *GJA1* | -2.945278531 | 1.74E-113 | 1.67E-111 |  |  |  |  |
| *GLP1R* | 2.515268977 | 1.70E-20 | 1.20E-19 |  |  |  |  |
| *GMDS* | 2.640637251 | 1.07E-126 | 1.40E-124 |  |  |  |  |
| *GSTA2* | 4.173839278 | 2.63E-39 | 4.24E-38 |  |  |  |  |
| *HDGFL1* | -3.035391002 | 1.99E-07 | 5.81E-07 |  |  |  |  |
| *HTR1B* | 2.51294207 | 2.61E-37 | 3.95E-36 |  |  |  |  |
| *IL17A* | 2.443956435 | 4.89E-18 | 3.01E-17 |  |  |  |  |
| *IYD* | 6.783769843 | 3.96E-264 | 3.11E-261 |  |  |  |  |
| *KAAG1* | 4.037507445 | 1.46E-57 | 4.18E-56 |  |  |  |  |
| *KCNK16* | 4.362926715 | 2.75E-17 | 1.62E-16 |  |  |  |  |
| *KCNK5* | 2.819475223 | 2.51E-95 | 1.68E-93 |  |  |  |  |
| *KCNQ5* | -3.068257818 | 2.20E-55 | 5.98E-54 |  |  |  |  |
| *KHDC1L* | -3.790150644 | 1.05E-30 | 1.23E-29 |  |  |  |  |
| *KIAA0319* | -2.043244698 | 5.82E-25 | 5.23E-24 |  |  |  |  |
| *KIAA1244* | 2.242212146 | 8.44E-74 | 3.59E-72 |  |  |  |  |
| *KLHL32* | 2.553003435 | 1.97E-39 | 3.20E-38 |  |  |  |  |
| *LGSN* | 3.197419313 | 4.32E-20 | 3.00E-19 |  |  |  |  |
| *LINC00240* | -2.119036132 | 3.87E-46 | 7.83E-45 |  |  |  |  |
| *LINC00473* | 2.460346917 | 1.34E-22 | 1.07E-21 |  |  |  |  |
| *LINC00602* | 2.251069233 | 3.68E-15 | 1.91E-14 |  |  |  |  |
| *LOC100422737* | 4.584366904 | 3.23E-67 | 1.18E-65 |  |  |  |  |
| *LOC100507203* | 5.31982771 | 2.23E-68 | 8.37E-67 |  |  |  |  |
| *LOC154092* | -2.772686185 | 2.29E-46 | 4.66E-45 |  |  |  |  |
| *LY6G6C* | -3.188803012 | 2.75E-33 | 3.57E-32 |  |  |  |  |
| *MDGA1* | -2.268743698 | 2.84E-53 | 7.18E-52 |  |  |  |  |
| *MEP1A* | 6.985659073 | 2.66E-137 | 4.16E-135 |  |  |  |  |
| *MLIP* | -2.972322236 | 3.32E-39 | 5.34E-38 |  |  |  |  |
| *MLN* | 3.95096382 | 5.59E-19 | 3.64E-18 |  |  |  |  |
| *Metazoa_SRP.91* | -2.514997937 | 1.26E-34 | 1.72E-33 |  |  |  |  |
| *NKAIN2* | -2.135088139 | 4.62E-14 | 2.23E-13 |  |  |  |  |
| *NR2E1* | -2.481893354 | 2.27E-16 | 1.27E-15 |  |  |  |  |
| *PAQR8* | 2.689231144 | 2.14E-114 | 2.13E-112 |  |  |  |  |
| *PBOV1* | 2.075874611 | 4.31E-22 | 3.32E-21 |  |  |  |  |
| *PERP* | -2.566046452 | 3.06E-102 | 2.36E-100 |  |  |  |  |
| *PGC* | 8.750699925 | 5.36E-136 | 8.19E-134 |  |  |  |  |
| *PI16* | 2.080847469 | 1.46E-15 | 7.76E-15 |  |  |  |  |
| *PNLDC1* | -2.723912691 | 2.32E-25 | 2.12E-24 |  |  |  |  |
| *PNPLA1* | -2.01557592 | 2.78E-23 | 2.28E-22 |  |  |  |  |
| *POPDC3* | -3.089869612 | 4.52E-36 | 6.50E-35 |  |  |  |  |
| *POU3F2* | -2.524358398 | 3.25E-15 | 1.69E-14 |  |  |  |  |
| *PPP1R14C* | -3.489020508 | 2.64E-52 | 6.50E-51 |  |  |  |  |
| *PRDM13* | -2.256326801 | 1.60E-06 | 4.28E-06 |  |  |  |  |
| *PRL* | 2.419960956 | 1.37E-16 | 7.76E-16 |  |  |  |  |
| *RAET1E* | -2.254289497 | 1.32E-22 | 1.04E-21 |  |  |  |  |
| *RAET1G* | -2.399290106 | 4.28E-35 | 5.93E-34 |  |  |  |  |
| *RAET1L* | -2.496020016 | 2.11E-22 | 1.65E-21 |  |  |  |  |
| *RFX6* | 4.57618461 | 1.10E-47 | 2.34E-46 |  |  |  |  |
| *RNF217* | -2.818353926 | 8.66E-101 | 6.42E-99 |  |  |  |  |
| *SAMD5* | 2.038773188 | 4.31E-36 | 6.20E-35 |  |  |  |  |
| *SCAND3* | -2.178500169 | 2.97E-16 | 1.65E-15 |  |  |  |  |
| *SCGN* | 6.091879928 | 1.01E-66 | 3.63E-65 |  |  |  |  |
| *SCML4* | 2.611906887 | 2.17E-56 | 6.05E-55 |  |  |  |  |
| *SFTA2* | 4.52630109 | 2.41E-67 | 8.81E-66 |  |  |  |  |
| *SH3BGRL2* | 2.389116243 | 2.83E-106 | 2.35E-104 |  |  |  |  |
| *SIM1* | -2.081465224 | 9.18E-06 | 2.28E-05 |  |  |  |  |
| *SLC17A1* | 2.766494088 | 2.90E-13 | 1.34E-12 |  |  |  |  |
| *SLC17A4* | 8.06982706 | 1.49E-156 | 3.15E-154 |  |  |  |  |
| *SLC35D3* | 3.454390047 | 1.74E-34 | 2.37E-33 |  |  |  |  |
| *SLC44A4* | 6.114279029 | 3.95E-292 | 4.72E-289 |  |  |  |  |
| *SPDEF* | 3.416277003 | 1.33E-53 | 3.40E-52 |  |  |  |  |
| *STL* | -2.787668102 | 3.54E-82 | 1.82E-80 |  |  |  |  |
| *TAAR1* | 4.3743101 | 3.91E-31 | 4.66E-30 |  |  |  |  |
| *TAAR3* | 4.331430707 | 3.63E-76 | 1.62E-74 |  |  |  |  |
| *TBX18* | -2.249902737 | 7.28E-26 | 6.81E-25 |  |  |  |  |
| *TCP10L2* | 4.929529441 | 2.72E-36 | 3.95E-35 |  |  |  |  |
| *TCP10* | 4.059761596 | 2.69E-31 | 3.24E-30 |  |  |  |  |
| *TFAP2A* | -2.053827064 | 9.87E-43 | 1.78E-41 |  |  |  |  |
| *TFAP2B* | -2.359311747 | 3.29E-07 | 9.40E-07 |  |  |  |  |
| *TINAG* | 4.147849996 | 2.45E-49 | 5.48E-48 |  |  |  |  |
| *TPD52L1* | -2.264025488 | 1.55E-40 | 2.62E-39 |  |  |  |  |
| *TRDN* | -2.330546473 | 4.99E-11 | 1.96E-10 |  |  |  |  |
| *TREML2* | 2.588054006 | 2.45E-35 | 3.43E-34 |  |  |  |  |
| *TREML3P* | -2.505854 | 1.12E-27 | 1.14E-26 |  |  |  |  |
| *TRIM10* | 4.144935067 | 1.40E-149 | 2.65E-147 |  |  |  |  |
| *TRIM15* | 5.225484844 | 1.81E-200 | 7.33E-198 |  |  |  |  |
| *TRIM31* | 5.71889086 | 5.58E-222 | 2.84E-219 |  |  |  |  |
| *TRIM40* | 5.227306456 | 8.60E-115 | 8.63E-113 |  |  |  |  |
| *TRNA_Ile.18* | -2.00716113 | 1.15E-35 | 1.62E-34 |  |  |  |  |
| *TSPO2* | 2.923801617 | 2.50E-70 | 9.96E-69 |  |  |  |  |
| *TTLL2* | 2.360237955 | 5.20E-30 | 5.89E-29 |  |  |  |  |
| *UBD* | 3.019059153 | 4.33E-55 | 1.16E-53 |  |  |  |  |
| *UNC5CL* | 3.615278075 | 2.95E-149 | 5.51E-147 |  |  |  |  |
| *VIP* | 2.361775518 | 9.97E-18 | 6.05E-17 |  |  |  |  |
| *VNN1* | 3.526877612 | 2.66E-57 | 7.56E-56 |  |  |  |  |
| *VNN2* | 2.646644647 | 1.80E-53 | 4.59E-52 |  |  |  |  |
| *Y_RNA.77* | -3.464691158 | 2.34E-18 | 1.46E-17 |  |  |  |  |
| *AB074160* | 2.2248411 | 2.39E-10 | 8.92E-10 |  |  |  |  |
| *ABCA13* | -4.024237291 | 1.37E-82 | 7.13E-81 |  |  |  |  |
| *ABHD11-AS1* | 3.627232806 | 8.96E-113 | 8.49E-111 |  |  |  |  |
| *ABP1* | 4.615232186 | 8.41E-147 | 1.49E-144 |  |  |  |  |
| *ACHE* | 3.44883353 | 1.77E-66 | 6.30E-65 |  |  |  |  |
| *ADAP1* | 2.468551255 | 3.14E-144 | 5.33E-142 |  |  |  |  |
| *AGMO* | 2.581207075 | 4.77E-29 | 5.19E-28 |  |  |  |  |
| *AGR2* | 4.971189641 | 4.80E-153 | 9.50E-151 |  |  |  |  |
| *AGR3* | 7.514070696 | 6.15E-277 | 5.46E-274 |  |  |  |  |
| *AK096803* | 5.899671386 | 1.11E-197 | 4.35E-195 |  |  |  |  |
| *AK097470* | -2.565522965 | 3.72E-15 | 1.93E-14 |  |  |  |  |
| *AK123474* | -2.051536336 | 1.08E-29 | 1.20E-28 |  |  |  |  |
| *AKR1B15* | -2.654969131 | 4.82E-19 | 3.15E-18 |  |  |  |  |
| *ASB4* | 3.257441676 | 4.06E-23 | 3.32E-22 |  |  |  |  |
| *AX746871.2* | 2.118888849 | 9.77E-07 | 2.67E-06 |  |  |  |  |
| *AZGP1* | 2.739481827 | 3.05E-32 | 3.81E-31 |  |  |  |  |
| *BC018166* | -5.212451477 | 3.23E-36 | 4.68E-35 |  |  |  |  |
| *BC034444* | 5.338490322 | 2.44E-58 | 7.10E-57 |  |  |  |  |
| *BC038570* | 2.41542378 | 5.80E-88 | 3.31E-86 |  |  |  |  |
| *BC040865* | 2.781428252 | 6.04E-32 | 7.47E-31 |  |  |  |  |
| *BC087859* | -2.005004453 | 3.29E-31 | 3.94E-30 |  |  |  |  |
| *BHLHA15* | 2.387090939 | 2.50E-42 | 4.45E-41 |  |  |  |  |
| *BX538274* | 6.523167496 | 7.50E-123 | 8.85E-121 |  |  |  |  |
| *CCL24* | 2.505919971 | 3.51E-33 | 4.54E-32 |  |  |  |  |
| *CFTR* | 5.950401822 | 1.21E-164 | 2.94E-162 |  |  |  |  |
| *CHN2* | 2.75643695 | 2.50E-110 | 2.22E-108 |  |  |  |  |
| *CHRM2* | 3.08655795 | 3.56E-20 | 2.48E-19 |  |  |  |  |
| *CLDN15* | 2.493525726 | 3.02E-45 | 5.98E-44 |  |  |  |  |
| *CLDN3* | 4.905748488 | 3.88E-134 | 5.87E-132 |  |  |  |  |
| *CPA1* | 2.375038289 | 2.59E-11 | 1.04E-10 |  |  |  |  |
| *CPA2* | 4.119220032 | 6.98E-51 | 1.65E-49 |  |  |  |  |
| *CPA4* | -4.029452502 | 3.21E-45 | 6.32E-44 |  |  |  |  |
| *CYP2W1* | 4.572498952 | 1.28E-62 | 4.25E-61 |  |  |  |  |
| *CYP3A4* | 4.92740628 | 3.15E-74 | 1.35E-72 |  |  |  |  |
| *CYP3A7* | 3.565576994 | 1.19E-101 | 9.12E-100 |  |  |  |  |
| *DDC* | 4.85955396 | 2.60E-120 | 2.86E-118 |  |  |  |  |
| *DJ051769* | 2.72913044 | 7.78E-21 | 5.62E-20 |  |  |  |  |
| *DLX5* | -4.175320278 | 6.65E-78 | 3.11E-76 |  |  |  |  |
| *DPY19L2P1* | -2.68338271 | 1.13E-31 | 1.38E-30 |  |  |  |  |
| *DQ578920* | 3.887477433 | 1.09E-11 | 4.48E-11 |  |  |  |  |
| *DQ583756* | 4.600039451 | 1.05E-111 | 9.63E-110 |  |  |  |  |
| *EGFR* | -2.260431162 | 7.55E-38 | 1.16E-36 |  |  |  |  |
| *EPO* | -4.228828901 | 4.63E-73 | 1.95E-71 |  |  |  |  |
| *EVX1* | 5.091244329 | 3.09E-57 | 8.77E-56 |  |  |  |  |
| *FAM221A* | 2.02274388 | 1.15E-66 | 4.13E-65 |  |  |  |  |
| *FAM71F1* | -2.45623728 | 4.56E-21 | 3.34E-20 |  |  |  |  |
| *FSCN1* | -3.012886052 | 6.57E-112 | 6.08E-110 |  |  |  |  |
| *GHRHR* | 4.433141588 | 2.09E-46 | 4.27E-45 |  |  |  |  |
| *GLI3* | -2.235040564 | 6.22E-55 | 1.65E-53 |  |  |  |  |
| *GPNMB* | -2.545712202 | 4.11E-62 | 1.35E-60 |  |  |  |  |
| *GU228584* | -2.258641995 | 1.22E-38 | 1.93E-37 |  |  |  |  |
| *HEPACAM2* | 6.025342002 | 5.72E-93 | 3.64E-91 |  |  |  |  |
| *HOTTIP* | 5.189658758 | 1.69E-106 | 1.41E-104 |  |  |  |  |
| *HOXA11-AS* | 2.511670198 | 3.17E-28 | 3.32E-27 |  |  |  |  |
| *HOXA13* | 3.312699247 | 9.41E-55 | 2.49E-53 |  |  |  |  |
| *ICA1* | 2.916954903 | 3.13E-155 | 6.46E-153 |  |  |  |  |
| *IGFBP1* | 4.806290311 | 2.11E-60 | 6.67E-59 |  |  |  |  |
| *KLRG2* | -3.637368336 | 6.98E-39 | 1.11E-37 |  |  |  |  |
| *LHFPL3* | 3.831702023 | 3.97E-48 | 8.56E-47 |  |  |  |  |
| *LOC100124692* | 6.627197895 | 3.20E-187 | 1.10E-184 |  |  |  |  |
| *LOC100129427* | 7.537135228 | 6.54E-174 | 1.76E-171 |  |  |  |  |
| *LOC100131257* | 2.922216762 | 5.43E-53 | 1.36E-51 |  |  |  |  |
| *LOC100506895* | -3.452312098 | 8.62E-83 | 4.53E-81 |  |  |  |  |
| *LOC285889* | 2.196340665 | 4.04E-14 | 1.96E-13 |  |  |  |  |
| *LOC645591* | 2.076783234 | 7.83E-08 | 2.37E-07 |  |  |  |  |
| *LOC723809* | 4.045393376 | 2.57E-57 | 7.32E-56 |  |  |  |  |
| *LOC93432* | 6.434095298 | 5.55E-158 | 1.20E-155 |  |  |  |  |
| *LRRC4* | -3.437075857 | 5.44E-102 | 4.19E-100 |  |  |  |  |
| *MACC1* | 2.124921797 | 4.59E-63 | 1.53E-61 |  |  |  |  |
| *MGAM* | 3.058344967 | 6.11E-55 | 1.63E-53 |  |  |  |  |
| *MIR3147* | 2.4511016 | 3.09E-05 | 7.28E-05 |  |  |  |  |
| *MIR3666* | -2.778301998 | 4.05E-09 | 1.37E-08 |  |  |  |  |
| *MIR595* | 3.690161173 | 6.60E-12 | 2.76E-11 |  |  |  |  |
| *MLXIPL* | 2.888812854 | 1.25E-45 | 2.49E-44 |  |  |  |  |
| *MNX1* | 2.668869737 | 1.55E-80 | 7.73E-79 |  |  |  |  |
| *MOGAT3* | 7.007885452 | 1.60E-189 | 5.85E-187 |  |  |  |  |
| *MOXD2P* | 3.104014526 | 9.84E-20 | 6.70E-19 |  |  |  |  |
| *MUC12* | 5.095260593 | 2.02E-87 | 1.13E-85 |  |  |  |  |
| *MUC17* | 8.077277141 | 9.41E-165 | 2.31E-162 |  |  |  |  |
| *MUC3A* | 6.062132472 | 2.73E-207 | 1.16E-204 |  |  |  |  |
| *Mir_340.10* | 2.104862981 | 3.81E-09 | 1.29E-08 |  |  |  |  |
| *NCF1.1* | 2.209823539 | 1.62E-05 | 3.93E-05 |  |  |  |  |
| *NPC1L1* | 5.022657931 | 1.13E-101 | 8.67E-100 |  |  |  |  |
| *NPSR1* | 3.51052104 | 7.57E-28 | 7.80E-27 |  |  |  |  |
| *PAX4* | 5.781367225 | 3.61E-42 | 6.41E-41 |  |  |  |  |
| *PDK4* | 2.711191007 | 7.16E-42 | 1.26E-40 |  |  |  |  |
| *PPP1R9A* | 2.621247447 | 2.59E-39 | 4.19E-38 |  |  |  |  |
| *PRR15* | 3.241825677 | 4.94E-134 | 7.42E-132 |  |  |  |  |
| *PRSS1* | 7.576367174 | 7.32E-121 | 8.18E-119 |  |  |  |  |
| *PRSS3P2* | 7.912431257 | 4.64E-111 | 4.20E-109 |  |  |  |  |
| *PTPRN2* | 3.938018007 | 6.94E-125 | 8.63E-123 |  |  |  |  |
| *PTPRZ1* | -3.879456024 | 2.28E-65 | 7.92E-64 |  |  |  |  |
| *RAB19* | 2.451393984 | 8.65E-83 | 4.54E-81 |  |  |  |  |
| *SHH* | 2.747116896 | 3.39E-42 | 6.03E-41 |  |  |  |  |
| *SLC26A3* | 7.332618199 | 4.30E-116 | 4.36E-114 |  |  |  |  |
| *SNORD93* | -2.280343411 | 8.47E-42 | 1.49E-40 |  |  |  |  |
| *SPAM1* | 3.066520891 | 2.39E-12 | 1.03E-11 |  |  |  |  |
| *STEAP1B* | -3.628429123 | 1.64E-99 | 1.18E-97 |  |  |  |  |
| *TAC1* | 3.047211648 | 6.29E-15 | 3.22E-14 |  |  |  |  |
| *TBX20* | -2.265122382 | 2.11E-10 | 7.90E-10 |  |  |  |  |
| *TCRB.3* | 2.040243221 | 2.43E-25 | 2.22E-24 |  |  |  |  |
| *TMEM139* | 4.408631121 | 1.32E-182 | 4.28E-180 |  |  |  |  |
| *TMEM176A* | 2.813423602 | 1.08E-94 | 7.12E-93 |  |  |  |  |
| *TMEM176B* | 2.24924068 | 3.98E-81 | 2.00E-79 |  |  |  |  |
| *TMEM229A* | 5.44608485 | 1.94E-75 | 8.52E-74 |  |  |  |  |
| *TRBV29-1* | 2.374840862 | 3.28E-33 | 4.25E-32 |  |  |  |  |
| *TRBV5-1* | 2.018738205 | 9.54E-30 | 1.07E-28 |  |  |  |  |
| *TRIM50* | 2.435460607 | 3.73E-20 | 2.59E-19 |  |  |  |  |
| *TSL-A* | 2.292279915 | 4.03E-30 | 4.58E-29 |  |  |  |  |
| *TSPAN12* | 2.63108773 | 1.05E-118 | 1.13E-116 |  |  |  |  |
| *VGF* | 2.123503394 | 3.14E-24 | 2.72E-23 |  |  |  |  |
| *VSTM2A* | 3.550370565 | 4.09E-22 | 3.15E-21 |  |  |  |  |
| *ZAN* | -3.278927222 | 1.01E-36 | 1.50E-35 |  |  |  |  |
| *ADAM28* | 2.443532096 | 2.08E-49 | 4.69E-48 |  |  |  |  |
| *ADCY8* | -4.642964139 | 3.17E-32 | 3.96E-31 |  |  |  |  |
| *AK001351* | -3.332796946 | 1.11E-53 | 2.85E-52 |  |  |  |  |
| *AK024242* | -2.958064039 | 8.20E-37 | 1.22E-35 |  |  |  |  |
| *AK057332* | 2.112466522 | 1.62E-14 | 8.04E-14 |  |  |  |  |
| *AK307207* | 2.705882201 | 2.39E-31 | 2.88E-30 |  |  |  |  |
| *AK308605* | -3.523920895 | 2.10E-38 | 3.29E-37 |  |  |  |  |
| *ANXA13* | 6.553892097 | 1.68E-181 | 5.37E-179 |  |  |  |  |
| *AX747124.2* | -2.010753062 | 0.000176463 | 0.000382246 |  |  |  |  |
| *AX747544* | 3.344465671 | 9.28E-33 | 1.18E-31 |  |  |  |  |
| *AX748380* | 2.501595189 | 4.49E-30 | 5.10E-29 |  |  |  |  |
| *BAALC* | -2.677712562 | 2.58E-33 | 3.36E-32 |  |  |  |  |
| *BAI1* | -3.280588797 | 1.52E-73 | 6.45E-72 |  |  |  |  |
| *BC017578* | 3.497109207 | 2.58E-41 | 4.47E-40 |  |  |  |  |
| *BC030294* | 2.401702044 | 4.35E-09 | 1.47E-08 |  |  |  |  |
| *BC038546* | -3.182358666 | 1.07E-19 | 7.30E-19 |  |  |  |  |
| *BLK* | 2.063328124 | 2.09E-16 | 1.17E-15 |  |  |  |  |
| *C8orf31* | 2.626443425 | 2.20E-54 | 5.76E-53 |  |  |  |  |
| *C8orf49* | 4.431550269 | 1.25E-84 | 6.72E-83 |  |  |  |  |
| *C8orf56* | -2.409657997 | 3.28E-19 | 2.17E-18 |  |  |  |  |
| *CA13* | 2.173899457 | 2.12E-74 | 9.09E-73 |  |  |  |  |
| *CA1* | 6.205826658 | 1.45E-91 | 8.92E-90 |  |  |  |  |
| *CA3* | 2.310250658 | 4.13E-47 | 8.62E-46 |  |  |  |  |
| *CA8* | 3.047536626 | 5.88E-52 | 1.43E-50 |  |  |  |  |
| *CDH17* | 7.554887879 | 3.57E-260 | 2.73E-257 |  |  |  |  |
| *CLDN23* | 3.105218042 | 7.68E-88 | 4.36E-86 |  |  |  |  |
| *CRH* | 3.296468236 | 1.10E-07 | 3.28E-07 |  |  |  |  |
| *DEFA5* | 6.615119105 | 3.25E-38 | 5.07E-37 |  |  |  |  |
| *DEFA6* | 7.628755992 | 2.43E-54 | 6.37E-53 |  |  |  |  |
| *DEPTOR* | 2.698523696 | 4.22E-128 | 5.63E-126 |  |  |  |  |
| *DQ580489* | -2.92008941 | 3.92E-11 | 1.55E-10 |  |  |  |  |
| *DUSP4* | 2.619236006 | 1.04E-74 | 4.50E-73 |  |  |  |  |
| *EFCAB1* | -3.207238032 | 5.38E-51 | 1.28E-49 |  |  |  |  |
| *FABP12* | -2.671583755 | 2.19E-11 | 8.83E-11 |  |  |  |  |
| *FABP4* | -3.108066964 | 5.33E-30 | 6.03E-29 |  |  |  |  |
| *FABP5* | -3.695920739 | 4.93E-104 | 3.95E-102 |  |  |  |  |
| *FABP9* | -2.71611048 | 6.89E-17 | 3.98E-16 |  |  |  |  |
| *FAM150A* | 2.595683743 | 2.34E-38 | 3.66E-37 |  |  |  |  |
| *FAM83A* | -3.340273202 | 6.69E-36 | 9.55E-35 |  |  |  |  |
| *FER1L6-AS1* | 2.306099831 | 5.97E-13 | 2.68E-12 |  |  |  |  |
| *FER1L6* | 2.627219735 | 8.57E-23 | 6.86E-22 |  |  |  |  |
| *FGF20* | 4.109555076 | 2.53E-29 | 2.78E-28 |  |  |  |  |
| *FGL1* | 3.948605296 | 9.24E-34 | 1.23E-32 |  |  |  |  |
| *FZD6* | -2.249091693 | 1.35E-123 | 1.63E-121 |  |  |  |  |
| *GATA4* | 4.7036808 | 7.60E-101 | 5.65E-99 |  |  |  |  |
| *GPT* | 2.642112809 | 2.20E-44 | 4.24E-43 |  |  |  |  |
| *GSDMC* | -4.27482268 | 2.56E-79 | 1.24E-77 |  |  |  |  |
| *HEY1* | -2.189662369 | 5.88E-73 | 2.46E-71 |  |  |  |  |
| *HNF4G* | 4.589610566 | 6.64E-210 | 2.90E-207 |  |  |  |  |
| *KBTBD11* | 3.205198761 | 4.46E-112 | 4.15E-110 |  |  |  |  |
| *LGI3* | -4.74827073 | 2.58E-67 | 9.42E-66 |  |  |  |  |
| *LINC00588* | 2.061808988 | 0.017636108 | 0.029144415 |  |  |  |  |
| *LOC100130231* | 2.211929473 | 1.75E-31 | 2.12E-30 |  |  |  |  |
| *LOC100130298* | 2.074558647 | 4.47E-30 | 5.08E-29 |  |  |  |  |
| *LOC100131726* | -3.785341286 | 5.50E-47 | 1.14E-45 |  |  |  |  |
| *LOC100288181* | -2.159460872 | 3.88E-24 | 3.34E-23 |  |  |  |  |
| *LOC100505659* | 5.044357169 | 5.65E-27 | 5.56E-26 |  |  |  |  |
| *LOC157381* | -3.442660755 | 1.97E-27 | 1.98E-26 |  |  |  |  |
| *LOC731779* | 3.357850573 | 7.92E-26 | 7.39E-25 |  |  |  |  |
| *LRP12* | -2.253391333 | 9.72E-96 | 6.54E-94 |  |  |  |  |
| *LY6D* | -3.705028498 | 1.52E-25 | 1.40E-24 |  |  |  |  |
| *LY6K* | -2.35569667 | 2.56E-19 | 1.70E-18 |  |  |  |  |
| *MTSS1* | -2.592596795 | 1.17E-82 | 6.09E-81 |  |  |  |  |
| *Metazoa_SRP.103* | -2.449126161 | 4.60E-05 | 0.000106215 |  |  |  |  |
| *NAT2* | 5.600107487 | 1.03E-98 | 7.34E-97 |  |  |  |  |
| *NDRG1* | -2.434356369 | 1.14E-99 | 8.30E-98 |  |  |  |  |
| *NEFL* | -5.845988318 | 2.08E-99 | 1.49E-97 |  |  |  |  |
| *NEFM* | -4.188767041 | 6.71E-70 | 2.62E-68 |  |  |  |  |
| *NKX2-6* | -3.015038028 | 2.69E-07 | 7.76E-07 |  |  |  |  |
| *NKX6-3* | 5.254403515 | 3.11E-44 | 5.95E-43 |  |  |  |  |
| *NPBWR1* | -4.00902347 | 7.89E-29 | 8.49E-28 |  |  |  |  |
| *NRG1* | -2.852750126 | 9.87E-45 | 1.93E-43 |  |  |  |  |
| *PNMA2* | 2.468989601 | 1.03E-42 | 1.85E-41 |  |  |  |  |
| *PSCA* | 3.746914413 | 2.09E-36 | 3.06E-35 |  |  |  |  |
| *RALYL* | 2.167173079 | 8.62E-10 | 3.08E-09 |  |  |  |  |
| *RGS20* | -3.807231664 | 3.31E-68 | 1.24E-66 |  |  |  |  |
| *RIMS2* | -2.702389914 | 8.67E-24 | 7.31E-23 |  |  |  |  |
| *SGCZ* | -2.071508219 | 1.08E-07 | 3.24E-07 |  |  |  |  |
| *SLC10A5* | 2.230426033 | 2.09E-53 | 5.29E-52 |  |  |  |  |
| *SLC18A1* | 4.814563628 | 1.45E-60 | 4.62E-59 |  |  |  |  |
| *SLURP1* | -2.108994364 | 1.10E-05 | 2.71E-05 |  |  |  |  |
| *SNAI2* | -2.767399485 | 2.60E-109 | 2.28E-107 |  |  |  |  |
| *SNX31* | -2.84047394 | 2.12E-26 | 2.05E-25 |  |  |  |  |
| *STAR* | -2.285053924 | 2.54E-53 | 6.42E-52 |  |  |  |  |
| *STMN2* | 2.705250744 | 1.21E-25 | 1.12E-24 |  |  |  |  |
| *TDH* | -2.041811281 | 9.33E-23 | 7.45E-22 |  |  |  |  |
| *TNFRSF10C* | 2.540827974 | 1.18E-66 | 4.22E-65 |  |  |  |  |
| *TNFRSF11B* | 2.735813355 | 1.31E-41 | 2.30E-40 |  |  |  |  |
| *TTPA* | 3.335724977 | 2.09E-41 | 3.63E-40 |  |  |  |  |
| *TUSC3* | -2.096166637 | 2.79E-44 | 5.36E-43 |  |  |  |  |
| *U3.12* | -3.220559568 | 4.65E-25 | 4.20E-24 |  |  |  |  |
| *XKR5* | -2.255444876 | 1.06E-35 | 1.50E-34 |  |  |  |  |
| *XKR9* | 2.339085407 | 3.34E-46 | 6.77E-45 |  |  |  |  |
| *ZMAT4* | -2.328566049 | 1.08E-11 | 4.44E-11 |  |  |  |  |
| *AK131516* | -2.155888912 | 6.16E-05 | 0.00014033 |  |  |  |  |
| *ALDOB* | 8.383896943 | 1.33E-233 | 7.94E-231 |  |  |  |  |
| *AMBP* | 4.404709463 | 2.66E-85 | 1.45E-83 |  |  |  |  |
| *ANKRD18B* | -2.460407974 | 4.41E-19 | 2.89E-18 |  |  |  |  |
| *ANXA1* | -2.254679888 | 4.66E-30 | 5.29E-29 |  |  |  |  |
| *AQP3* | -2.002871141 | 2.49E-20 | 1.75E-19 |  |  |  |  |
| *AQP7* | 2.89722147 | 3.34E-63 | 1.12E-61 |  |  |  |  |
| *AY343892* | 2.181954043 | 2.94E-10 | 1.09E-09 |  |  |  |  |
| *AY343902* | 2.836005056 | 5.76E-06 | 1.46E-05 |  |  |  |  |
| *BAAT* | 5.372013415 | 2.06E-88 | 1.19E-86 |  |  |  |  |
| *BARX1* | 2.349816626 | 1.44E-21 | 1.08E-20 |  |  |  |  |
| *BC016143.9* | -2.391022425 | 9.49E-32 | 1.16E-30 |  |  |  |  |
| *BC037833* | 2.921143484 | 1.11E-41 | 1.94E-40 |  |  |  |  |
| *BC039180* | -2.52524796 | 3.13E-41 | 5.41E-40 |  |  |  |  |
| *BC065763* | 2.809163231 | 1.45E-19 | 9.79E-19 |  |  |  |  |
| *BICD2* | -2.596718269 | 5.86E-224 | 3.04E-221 |  |  |  |  |
| *C8G* | 2.538953468 | 2.79E-31 | 3.35E-30 |  |  |  |  |
| *C9orf135* | 3.335598139 | 8.08E-17 | 4.64E-16 |  |  |  |  |
| *C9orf152* | 4.845932337 | 8.22E-171 | 2.13E-168 |  |  |  |  |
| *CACNA1B* | -2.796786998 | 1.25E-25 | 1.16E-24 |  |  |  |  |
| *CELP* | 2.081869903 | 1.09E-11 | 4.49E-11 |  |  |  |  |
| *CNTNAP3B.2* | -2.298679303 | 3.25E-35 | 4.51E-34 |  |  |  |  |
| *CNTNAP3* | -2.779865905 | 2.36E-43 | 4.36E-42 |  |  |  |  |
| *DEC1* | -2.89570425 | 2.95E-31 | 3.53E-30 |  |  |  |  |
| *DIRAS2* | -3.660448076 | 9.99E-37 | 1.48E-35 |  |  |  |  |
| *DL491118* | 2.17702949 | 0.000181204 | 0.000391868 |  |  |  |  |
| *DMRT1* | -2.328059152 | 2.22E-13 | 1.03E-12 |  |  |  |  |
| *DMRT2* | -4.874190283 | 3.89E-62 | 1.28E-60 |  |  |  |  |
| *DMRT3* | -2.888302403 | 6.31E-25 | 5.66E-24 |  |  |  |  |
| *DQ571524.3* | 2.326509253 | 2.74E-05 | 6.48E-05 |  |  |  |  |
| *DQ574810.2* | -2.727524237 | 3.39E-10 | 1.25E-09 |  |  |  |  |
| *DQ585850.2* | -2.857889761 | 1.04E-08 | 3.40E-08 |  |  |  |  |
| *DQ586551* | -2.184712481 | 4.81E-21 | 3.52E-20 |  |  |  |  |
| *DQ587539.18* | -2.583467951 | 1.75E-14 | 8.68E-14 |  |  |  |  |
| *DQ587955* | -2.260684577 | 8.67E-07 | 2.38E-06 |  |  |  |  |
| *DQ590442.1* | -2.862346391 | 2.31E-27 | 2.32E-26 |  |  |  |  |
| *DQ590589.21* | -2.145949442 | 1.02E-05 | 2.52E-05 |  |  |  |  |
| *DQ594696.2* | -2.588679916 | 2.15E-06 | 5.68E-06 |  |  |  |  |
| *DQ596206.4* | -2.977975363 | 3.07E-24 | 2.66E-23 |  |  |  |  |
| *DQ597117.3* | -2.826594782 | 8.69E-10 | 3.10E-09 |  |  |  |  |
| *ELAVL2* | -2.543744642 | 4.84E-28 | 5.03E-27 |  |  |  |  |
| *ENTPD8* | 4.611896197 | 1.13E-113 | 1.09E-111 |  |  |  |  |
| *FBP1* | 2.620439401 | 8.84E-80 | 4.34E-78 |  |  |  |  |
| *FBP2* | 2.150640822 | 1.33E-29 | 1.47E-28 |  |  |  |  |
| *FOXB2* | -2.465661371 | 0.00045286 | 0.000937181 |  |  |  |  |
| *FOXE1* | -6.048821165 | 2.62E-54 | 6.84E-53 |  |  |  |  |
| *FREM1* | 2.032056792 | 8.46E-20 | 5.78E-19 |  |  |  |  |
| *GCNT1* | 2.789290255 | 4.13E-90 | 2.47E-88 |  |  |  |  |
| *GFI1B* | 3.983270225 | 9.72E-60 | 2.98E-58 |  |  |  |  |
| *GNA14* | 2.179438553 | 2.56E-54 | 6.68E-53 |  |  |  |  |
| *GOLM1* | 2.378864985 | 1.23E-93 | 7.93E-92 |  |  |  |  |
| *GPR144* | -2.195584083 | 1.21E-16 | 6.88E-16 |  |  |  |  |
| *INSL4* | 3.059469153 | 8.09E-17 | 4.65E-16 |  |  |  |  |
| *KIAA1045* | -2.51043297 | 5.63E-31 | 6.67E-30 |  |  |  |  |
| *KIF12* | 3.937821714 | 8.26E-147 | 1.48E-144 |  |  |  |  |
| *LCN15* | 4.896756997 | 2.38E-33 | 3.11E-32 |  |  |  |  |
| *LCN2* | 2.628695172 | 6.97E-30 | 7.83E-29 |  |  |  |  |
| *LHX2* | -3.53927182 | 2.50E-82 | 1.29E-80 |  |  |  |  |
| *LINGO2* | -2.216872357 | 2.66E-17 | 1.57E-16 |  |  |  |  |
| *LOC286238* | -2.668376 | 3.37E-07 | 9.63E-07 |  |  |  |  |
| *LOC286359* | 2.517663898 | 1.49E-07 | 4.39E-07 |  |  |  |  |
| *LOC440173* | -2.711947417 | 1.37E-29 | 1.52E-28 |  |  |  |  |
| *LRRC19* | 2.919023174 | 9.68E-43 | 1.75E-41 |  |  |  |  |
| *LRRC26* | 3.280688987 | 9.14E-59 | 2.70E-57 |  |  |  |  |
| *MIR31* | -2.435063112 | 6.31E-07 | 1.76E-06 |  |  |  |  |
| *MIR3621* | 2.887795737 | 1.68E-16 | 9.45E-16 |  |  |  |  |
| *MORN5* | 2.804061847 | 7.59E-18 | 4.63E-17 |  |  |  |  |
| *NTRK2* | -4.327418278 | 2.36E-81 | 1.19E-79 |  |  |  |  |
| *OGN* | 2.782943146 | 8.41E-28 | 8.66E-27 |  |  |  |  |
| *OLFM1* | -2.118178141 | 5.12E-34 | 6.87E-33 |  |  |  |  |
| *ORM1* | 6.128401521 | 4.13E-94 | 2.70E-92 |  |  |  |  |
| *PIP5K1B* | 4.437268748 | 6.09E-231 | 3.57E-228 |  |  |  |  |
| *PRKACG* | 2.38582783 | 4.67E-19 | 3.05E-18 |  |  |  |  |
| *PRSS3* | 2.852717605 | 4.67E-62 | 1.53E-60 |  |  |  |  |
| *PTGDS* | 2.232545872 | 7.86E-32 | 9.67E-31 |  |  |  |  |
| *PTPRD* | -2.039312112 | 1.39E-28 | 1.49E-27 |  |  |  |  |
| *RMRP* | 2.446868131 | 7.88E-36 | 1.12E-34 |  |  |  |  |
| *RNF183* | 2.094421405 | 1.54E-28 | 1.64E-27 |  |  |  |  |
| *SLC1A1* | 2.436159443 | 3.95E-59 | 1.18E-57 |  |  |  |  |
| *SPINK4* | 8.765174659 | 2.10E-181 | 6.64E-179 |  |  |  |  |
| *TMEM8C* | 2.58726025 | 2.38E-07 | 6.88E-07 |  |  |  |  |
| *TNC* | -2.696838787 | 3.85E-51 | 9.24E-50 |  |  |  |  |
| *TNFSF15* | 2.02659903 | 3.09E-68 | 1.16E-66 |  |  |  |  |
| *TXNDC8* | -2.219858669 | 8.68E-13 | 3.87E-12 |  |  |  |  |
| *FLJ44838* | 2.377126562 | 1.48E-06 | 3.97E-06 |  |  |  |  |
| *ACE2* | 2.196998489 | 1.36E-22 | 1.08E-21 |  |  |  |  |
| *AFF2* | -2.386008645 | 1.70E-33 | 2.24E-32 |  |  |  |  |
| *AGTR2* | 2.183925847 | 1.53E-06 | 4.10E-06 |  |  |  |  |
| *AK056105* | 2.14533815 | 1.43E-07 | 4.23E-07 |  |  |  |  |
| *AK123758* | 4.302544956 | 1.17E-49 | 2.66E-48 |  |  |  |  |
| *AKAP4* | 4.804621629 | 1.16E-36 | 1.71E-35 |  |  |  |  |
| *AMELX* | 3.657343879 | 5.43E-34 | 7.28E-33 |  |  |  |  |
| *ARHGAP6* | 2.217373102 | 7.43E-56 | 2.04E-54 |  |  |  |  |
| *ARSE* | 4.769562625 | 6.41E-189 | 2.26E-186 |  |  |  |  |
| *ARX* | 3.459666816 | 5.10E-42 | 9.02E-41 |  |  |  |  |
| *BEX1* | 2.542462418 | 1.20E-15 | 6.41E-15 |  |  |  |  |
| *CAPN6* | 2.168239928 | 2.35E-17 | 1.39E-16 |  |  |  |  |
| *CD40LG* | 2.353314937 | 2.72E-46 | 5.52E-45 |  |  |  |  |
| *CITED1* | 2.250177628 | 2.37E-33 | 3.10E-32 |  |  |  |  |
| *CLCN4* | 2.009955579 | 4.32E-40 | 7.19E-39 |  |  |  |  |
| *CLDN2* | 7.244079774 | 2.01E-210 | 8.94E-208 |  |  |  |  |
| *COL4A5* | -2.621227733 | 1.02E-55 | 2.78E-54 |  |  |  |  |
| *COL4A6* | -3.074465764 | 9.54E-49 | 2.10E-47 |  |  |  |  |
| *CPXCR1* | -2.235576084 | 2.36E-05 | 5.62E-05 |  |  |  |  |
| *CXorf61* | 2.977408715 | 1.37E-16 | 7.76E-16 |  |  |  |  |
| *DCAF12L1* | 2.86590706 | 4.14E-09 | 1.40E-08 |  |  |  |  |
| *DCX* | 3.034062542 | 7.56E-30 | 8.48E-29 |  |  |  |  |
| *DGKK* | 2.132398652 | 3.20E-07 | 9.16E-07 |  |  |  |  |
| *DKFZp686D0853* | 2.918018085 | 2.63E-32 | 3.30E-31 |  |  |  |  |
| *DUSP9* | -2.097746564 | 1.85E-13 | 8.63E-13 |  |  |  |  |
| *FAM9C* | 2.984294892 | 9.18E-18 | 5.58E-17 |  |  |  |  |
| *FOXR2* | 2.36557139 | 0.001307741 | 0.002557169 |  |  |  |  |
| *GABRE* | -2.875865253 | 8.74E-44 | 1.65E-42 |  |  |  |  |
| *GABRQ* | -3.290550478 | 3.31E-33 | 4.29E-32 |  |  |  |  |
| *GJB1* | 7.905156784 | 0 | 0 |  |  |  |  |
| *GLOD5* | 3.720057501 | 5.77E-120 | 6.27E-118 |  |  |  |  |
| *GPR112* | 2.559876259 | 1.06E-13 | 5.03E-13 |  |  |  |  |
| *GPR119* | 2.450615884 | 2.84E-06 | 7.42E-06 |  |  |  |  |
| *GPR50* | -4.934564135 | 4.73E-32 | 5.88E-31 |  |  |  |  |
| *H2BFM* | -2.447265296 | 8.80E-07 | 2.42E-06 |  |  |  |  |
| *HEPH* | 2.437640214 | 9.20E-63 | 3.05E-61 |  |  |  |  |
| *HS6ST2* | -2.886678554 | 9.89E-32 | 1.21E-30 |  |  |  |  |
| *IL2RG* | 2.386519374 | 2.87E-60 | 9.03E-59 |  |  |  |  |
| *KLF8* | -2.095926616 | 3.82E-43 | 7.02E-42 |  |  |  |  |
| *LHFPL1* | -2.336203298 | 1.23E-20 | 8.84E-20 |  |  |  |  |
| *LONRF3* | 2.274291037 | 8.67E-62 | 2.82E-60 |  |  |  |  |
| *MAGEA11* | -2.558552454 | 8.33E-08 | 2.51E-07 |  |  |  |  |
| *MIR452* | -2.081543622 | 4.87E-07 | 1.37E-06 |  |  |  |  |
| *MIR4767* | 2.089056501 | 6.15E-46 | 1.24E-44 |  |  |  |  |
| *MUM1L1* | -2.526183642 | 5.09E-17 | 2.96E-16 |  |  |  |  |
| *NAP1L2* | -2.000331912 | 6.63E-23 | 5.34E-22 |  |  |  |  |
| *NOX1* | 4.374082756 | 1.29E-68 | 4.89E-67 |  |  |  |  |
| *NR0B1* | -5.894216675 | 2.98E-40 | 4.99E-39 |  |  |  |  |
| *NUDT11* | -3.254042626 | 1.07E-82 | 5.56E-81 |  |  |  |  |
| *NXF3* | 4.58332201 | 6.62E-68 | 2.45E-66 |  |  |  |  |
| *OTC* | 7.609607814 | 2.54E-114 | 2.50E-112 |  |  |  |  |
| *PAGE1* | 2.68359425 | 1.90E-08 | 6.09E-08 |  |  |  |  |
| *PCDH19* | -3.25840859 | 1.22E-58 | 3.58E-57 |  |  |  |  |
| *PCYT1B* | -2.684566398 | 2.26E-35 | 3.16E-34 |  |  |  |  |
| *REPS2* | 2.059193284 | 8.14E-71 | 3.29E-69 |  |  |  |  |
| *RGN* | 2.961401203 | 4.61E-46 | 9.31E-45 |  |  |  |  |
| *RHOXF2* | 2.517437069 | 0.001992875 | 0.003806732 |  |  |  |  |
| *RN7SK.128* | 2.20643542 | 0.006064476 | 0.010838009 |  |  |  |  |
| *SERPINA7* | 3.325090005 | 2.40E-07 | 6.93E-07 |  |  |  |  |
| *SMPX* | 2.829291644 | 3.36E-17 | 1.97E-16 |  |  |  |  |
| *SPANXC* | -3.626633406 | 1.06E-08 | 3.46E-08 |  |  |  |  |
| *SPANXE* | -3.660366158 | 1.77E-10 | 6.67E-10 |  |  |  |  |
| *SSX2.1* | 2.967764331 | 0.001698613 | 0.003273064 |  |  |  |  |
| *SSX4B* | 4.091444013 | 3.97E-08 | 1.23E-07 |  |  |  |  |
| *SSX4* | 2.828525551 | 7.83E-05 | 0.000176202 |  |  |  |  |
| *SYP* | 2.053361133 | 4.36E-30 | 4.95E-29 |  |  |  |  |
| *TNMD* | 2.13161363 | 2.31E-12 | 1.00E-11 |  |  |  |  |
| *TRNA_Ile.19* | -2.108435947 | 7.36E-12 | 3.07E-11 |  |  |  |  |
| *VSIG1* | 5.777929036 | 2.39E-102 | 1.85E-100 |  |  |  |  |
| *XAGE3* | 2.819860655 | 5.91E-10 | 2.14E-09 |  |  |  |  |
| *XG* | -2.005658973 | 1.95E-18 | 1.23E-17 |  |  |  |  |
| *XPNPEP2* | 4.083045622 | 3.15E-61 | 1.01E-59 |  |  |  |  |
| *ZIC3* | -2.925737547 | 2.66E-08 | 8.43E-08 |  |  |  |  |
| *ZNF185* | -2.158200356 | 1.44E-30 | 1.68E-29 |  |  |  |  |
| *BC041884* | -3.155308735 | 1.39E-07 | 4.11E-07 |  |  |  |  |
| *DAZ1* | 6.336755632 | 2.32E-24 | 2.03E-23 |  |  |  |  |
| *DAZ2* | 6.266986305 | 3.21E-22 | 2.49E-21 |  |  |  |  |
| *DAZ4* | 5.202831409 | 1.94E-14 | 9.63E-14 |  |  |  |  |
| *IGL@.2* | 2.064452499 | 7.44E-10 | 2.67E-09 |  |  |  |  |
| *RBMY2EP* | 2.69093549 | 0.000246494 | 0.000525499 |  |  |  |  |
| *SRY* | -2.378056472 | 1.24E-05 | 3.04E-05 |  |  |  |  |
| *TBL1Y* | -2.482247098 | 2.08E-17 | 1.24E-16 |  |  |  |  |
| *TTTY13* | 2.763636903 | 8.00E-05 | 0.000179989 |  |  |  |  |
| *TTTY16* | -3.073513943 | 1.46E-08 | 4.71E-08 |  |  |  |  |
| **EA vs. ES** | | | | | | | |

| **mRNAs** | **log2 Fold Change** | **p value** | **p adj** | **microRNAs** | **log2 Fold Change** | **p value** | **p adj** |
| --- | --- | --- | --- | --- | --- | --- | --- |
| *AADACL3* | 2.153034744 | 0.000232 | 0.0006197 | *hsa-mir-122* | 2.524410668 | 1.47E-08 | 9.50E-08 |
| *ABCA4* | 4.580290407 | 1.18E-45 | 5.33E-44 | *hsa-mir-1224* | 2.756629245 | 1.51E-10 | 1.28E-09 |
| *ACTL8* | -2.764747557 | 1.46E-10 | 8.49E-10 | *hsa-mir-1257* | 2.251202253 | 0.000543479 | 0.00168255 |
| *AGMAT* | -2.958078062 | 2.19E-103 | 6.25E-101 | *hsa-mir-1266* | 2.670262128 | 3.82E-37 | 1.62E-35 |
| *AGT* | -4.763306984 | 2.08E-89 | 4.15E-87 | *hsa-mir-1293* | -3.211810944 | 3.85E-30 | 1.00E-28 |
| *AJAP1* | 2.482451146 | 3.10E-20 | 3.85E-19 | *hsa-mir-1305* | -3.124716075 | 3.71E-17 | 5.13E-16 |
| *AK025975* | -3.170330312 | 2.18E-29 | 4.63E-28 | *hsa-mir-137* | -3.107223886 | 2.12E-15 | 2.66E-14 |
| *AK054708* | -2.63555062 | 9.62E-25 | 1.59E-23 | *hsa-mir-147b* | 2.485845114 | 4.22E-31 | 1.36E-29 |
| *AK054726* | 4.466709012 | 1.40E-30 | 3.15E-29 | *hsa-mir-149* | -2.724813996 | 8.90E-39 | 4.31E-37 |
| *AK056431* | 2.555315783 | 1.15E-18 | 1.28E-17 | *hsa-mir-187* | -2.887030875 | 1.44E-22 | 2.50E-21 |
| *AK057554* | 2.68615431 | 0.000645879 | 0.001600323 | *hsa-mir-1910* | -3.683369716 | 1.32E-23 | 2.49E-22 |
| *AK094607* | 2.770092225 | 1.13E-08 | 5.38E-08 | *hsa-mir-192* | 6.955990782 | 0 | 0 |
| *AK124056* | 3.4911473 | 9.13E-67 | 8.64E-65 | *hsa-mir-194-1* | 6.967244582 | 0 | 0 |
| *AK125078* | 2.134178116 | 8.71E-05 | 0.000249738 | *hsa-mir-194-2* | 6.922436997 | 0 | 0 |
| *AKR7A3* | -4.396502484 | 2.94E-101 | 7.68E-99 | *hsa-mir-203* | -2.065636077 | 2.48E-17 | 3.66E-16 |
| *AKR7L* | -3.9102472 | 8.36E-120 | 3.07E-117 | *hsa-mir-205* | -4.942493486 | 3.39E-56 | 2.88E-54 |
| *AMPD1* | -2.06397683 | 6.73E-14 | 5.30E-13 | *hsa-mir-206* | 2.367180257 | 2.75E-11 | 2.49E-10 |
| *ANKRD45* | -2.153090821 | 5.36E-14 | 4.25E-13 | *hsa-mir-2117* | -2.261174535 | 0.005596303 | 0.013648537 |
| *ANKRD65* | 2.018710284 | 8.63E-24 | 1.34E-22 | *hsa-mir-215* | 9.038740421 | 1.06E-256 | 1.79E-254 |
| *APOA2* | -3.097436993 | 1.35E-16 | 1.31E-15 | *hsa-mir-224* | -2.750060003 | 2.58E-37 | 1.17E-35 |
| *APOBEC4* | -2.660940548 | 1.95E-15 | 1.73E-14 | *hsa-mir-3131* | 7.0309072 | 1.09E-39 | 5.66E-38 |
| *ARTN* | 3.767171206 | 5.54E-78 | 7.79E-76 | *hsa-mir-3166* | -3.129367512 | 7.61E-09 | 5.27E-08 |
| *ASTN1* | 2.62669308 | 3.46E-14 | 2.79E-13 | *hsa-mir-3189* | 4.667149789 | 1.89E-16 | 2.46E-15 |
| *AX229788* | -2.783231947 | 1.09E-07 | 4.62E-07 | *hsa-mir-338* | 2.043974087 | 1.74E-40 | 9.84E-39 |
| *AX748428* | -3.640507935 | 2.56E-33 | 6.69E-32 | *hsa-mir-34b* | -3.184071293 | 4.89E-37 | 1.95E-35 |
| *BC016143.1* | 5.121850592 | 1.23E-80 | 1.92E-78 | *hsa-mir-34c* | -3.242281904 | 1.47E-42 | 9.08E-41 |
| *BC016972* | -2.757512565 | 4.27E-47 | 2.09E-45 | *hsa-mir-3659* | -2.212295673 | 0.000374706 | 0.001221398 |
| *BC030750* | -2.356920925 | 3.50E-71 | 3.84E-69 | *hsa-mir-371* | 4.327063095 | 1.11E-06 | 5.56E-06 |
| *BC030768* | -2.549674241 | 3.81E-17 | 3.87E-16 | *hsa-mir-372* | 3.714814281 | 6.87E-15 | 8.32E-14 |
| *BC032040* | 2.956960082 | 1.21E-21 | 1.65E-20 | *hsa-mir-373* | 2.3126982 | 0.004653583 | 0.01155725 |
| *BC037321* | 2.159387848 | 2.00E-14 | 1.64E-13 | *hsa-mir-375* | 4.773586663 | 1.71E-65 | 1.66E-63 |
| *BC038769* | 2.444306194 | 4.85E-36 | 1.45E-34 | *hsa-mir-3927* | -3.155463169 | 1.89E-06 | 9.00E-06 |
| *BC041341* | 2.05808828 | 0.009292764 | 0.018379849 | *hsa-mir-452* | -2.14083954 | 6.52E-29 | 1.52E-27 |
| *BC041441* | 2.117005244 | 2.84E-05 | 8.74E-05 | *hsa-mir-488* | -2.611510601 | 5.59E-06 | 2.45E-05 |
| *BC042538* | -4.301615359 | 2.64E-21 | 3.50E-20 | *hsa-mir-514-1* | -2.325753883 | 3.65E-09 | 2.64E-08 |
| *BC127868* | 2.160809319 | 0.000101498 | 0.000288028 | *hsa-mir-514-2* | -2.27810404 | 2.99E-09 | 2.23E-08 |
| *BCL2L15* | -5.850223093 | 6.58E-173 | 5.77E-170 | *hsa-mir-514-3* | -2.793403946 | 6.60E-13 | 6.88E-12 |
| *BRDT* | 3.198728779 | 5.92E-10 | 3.24E-09 | *hsa-mir-516b-1* | -2.010171105 | 0.000104079 | 0.000379385 |
| *C1orf110* | 3.061253118 | 2.65E-13 | 1.98E-12 | *hsa-mir-517a* | -2.460837548 | 5.31E-08 | 3.13E-07 |
| *C1orf130* | -3.454874221 | 5.98E-46 | 2.76E-44 | *hsa-mir-517b* | -2.456536485 | 5.49E-08 | 3.21E-07 |
| *C1orf180* | -2.669430582 | 2.18E-12 | 1.50E-11 | *hsa-mir-517c* | -2.081858598 | 0.000848116 | 0.002544347 |
| *C1orf65* | 2.215822126 | 6.46E-14 | 5.10E-13 | *hsa-mir-518b* | -2.163165616 | 1.23E-07 | 6.95E-07 |
| *C1orf68* | 4.243744621 | 4.83E-19 | 5.55E-18 | *hsa-mir-518c* | -2.081584753 | 2.32E-06 | 1.09E-05 |
| *C1orf81* | -2.081673827 | 4.43E-21 | 5.80E-20 | *hsa-mir-518e* | -2.044080083 | 0.000238164 | 0.000795445 |
| *C4BPA* | -6.164095123 | 5.71E-61 | 4.55E-59 | *hsa-mir-518f* | -2.691052732 | 1.65E-06 | 7.95E-06 |
| *C4BPB* | -7.067559307 | 5.70E-122 | 2.15E-119 | *hsa-mir-520g* | -2.197566273 | 3.32E-05 | 0.000128559 |
| *C8A* | -3.274524681 | 1.52E-11 | 9.68E-11 | *hsa-mir-526b* | -2.591140058 | 1.22E-12 | 1.26E-11 |
| *CACNA1E* | -2.080547532 | 1.79E-13 | 1.36E-12 | *hsa-mir-552* | 7.971844006 | 6.16E-80 | 6.96E-78 |
| *CAMK2N1* | -2.010389232 | 1.08E-30 | 2.45E-29 | *hsa-mir-559* | 2.714259871 | 4.19E-17 | 5.68E-16 |
| *CAPN8* | -5.912000744 | 1.88E-87 | 3.62E-85 | *hsa-mir-577* | 2.407108645 | 4.27E-21 | 7.07E-20 |
| *CAPN9* | -4.56844988 | 6.47E-53 | 3.78E-51 | *hsa-mir-592* | 2.351445835 | 6.46E-31 | 1.91E-29 |
| *CD55* | -2.204501664 | 3.48E-38 | 1.15E-36 | *hsa-mir-614* | 2.39621327 | 0.000489781 | 0.00155007 |
| *CELA3B* | -4.227877804 | 2.86E-33 | 7.44E-32 | *hsa-mir-7-2* | 2.58146155 | 2.90E-27 | 6.15E-26 |
| *CELF3* | -2.640426276 | 3.31E-33 | 8.57E-32 | *hsa-mir-7-3* | 2.768621714 | 5.70E-31 | 1.76E-29 |
| *CELSR2* | 2.15917085 | 4.77E-45 | 2.12E-43 | *hsa-mir-708* | -2.547094805 | 5.26E-50 | 3.96E-48 |
| *CGN* | -3.284220283 | 1.27E-99 | 3.16E-97 | *hsa-mir-802* | 5.501173433 | 1.36E-13 | 1.54E-12 |
| *CHIA* | 4.997379752 | 1.52E-09 | 7.96E-09 | *hsa-mir-873* | -2.19987184 | 1.50E-06 | 7.28E-06 |
| *CLCA1* | -6.567165359 | 4.99E-52 | 2.86E-50 | *hsa-mir-876* | -2.730171131 | 6.42E-07 | 3.32E-06 |
| *CLCA2* | 3.277800411 | 2.30E-17 | 2.36E-16 | *hsa-mir-934* | 2.376515745 | 2.09E-08 | 1.30E-07 |
| *COL11A1* | 2.106872729 | 2.96E-10 | 1.67E-09 | *hsa-mir-944* | -5.512271746 | 1.48E-83 | 2.01E-81 |
| *CR936677* | 2.385681264 | 7.60E-13 | 5.46E-12 |  |  |  |  |
| *CR936711* | 2.405951602 | 7.04E-07 | 2.70E-06 |  |  |  |  |
| *CRP* | -2.624118657 | 4.38E-10 | 2.43E-09 |  |  |  |  |
| *CTSE* | -9.491569625 | 3.51E-169 | 2.72E-166 |  |  |  |  |
| *CYMP* | -3.750087792 | 9.50E-26 | 1.67E-24 |  |  |  |  |
| *CYP4Z2P* | 2.369481297 | 5.43E-10 | 2.99E-09 |  |  |  |  |
| *DDAH1* | -2.942928707 | 1.98E-90 | 4.07E-88 |  |  |  |  |
| *DM075093* | -3.062365476 | 4.30E-55 | 2.74E-53 |  |  |  |  |
| *DMRTA2* | 2.294268765 | 6.58E-11 | 3.95E-10 |  |  |  |  |
| *DNALI1* | -2.295232973 | 1.37E-31 | 3.28E-30 |  |  |  |  |
| *DQ574660.1* | 2.863660489 | 7.97E-08 | 3.44E-07 |  |  |  |  |
| *DQ575010.1* | 2.827898855 | 9.93E-09 | 4.77E-08 |  |  |  |  |
| *DUSP27* | -6.07966074 | 1.71E-41 | 6.52E-40 |  |  |  |  |
| *EDARADD* | 2.115150686 | 4.39E-86 | 8.05E-84 |  |  |  |  |
| *EDN2* | 3.403545397 | 1.12E-30 | 2.54E-29 |  |  |  |  |
| *EPHA10* | -3.266115742 | 1.60E-47 | 7.91E-46 |  |  |  |  |
| *EPS8L3* | -8.784671862 | 2.65E-220 | 5.14E-217 |  |  |  |  |
| *ETNK2* | 2.46508685 | 4.21E-38 | 1.38E-36 |  |  |  |  |
| *F5* | -4.834946949 | 1.17E-71 | 1.33E-69 |  |  |  |  |
| *FAM177B* | -4.309296616 | 3.73E-35 | 1.07E-33 |  |  |  |  |
| *FCAMR* | -2.303033238 | 6.19E-11 | 3.72E-10 |  |  |  |  |
| *FLG2* | 3.230982273 | 9.67E-17 | 9.48E-16 |  |  |  |  |
| *FLG* | 3.307342238 | 4.59E-25 | 7.74E-24 |  |  |  |  |
| *FLJ31662* | 2.479644702 | 0.00011445 | 0.000322525 |  |  |  |  |
| *FLJ42875.1* | -4.457551503 | 7.93E-52 | 4.51E-50 |  |  |  |  |
| *FLJ42875.2* | -3.622073439 | 1.75E-48 | 9.01E-47 |  |  |  |  |
| *FMO5* | -4.072118696 | 1.36E-131 | 6.48E-129 |  |  |  |  |
| *FMO6P* | 3.625083792 | 1.32E-25 | 2.30E-24 |  |  |  |  |
| *FMO9P* | 2.297654305 | 1.55E-09 | 8.09E-09 |  |  |  |  |
| *FOXD3* | 2.733814639 | 7.96E-20 | 9.64E-19 |  |  |  |  |
| *GBP1P1* | 2.20011323 | 1.18E-33 | 3.13E-32 |  |  |  |  |
| *GBP6* | 2.240990116 | 1.42E-09 | 7.48E-09 |  |  |  |  |
| *GCLM* | 2.008864747 | 4.07E-29 | 8.57E-28 |  |  |  |  |
| *GIPC2* | -2.455294746 | 7.79E-29 | 1.62E-27 |  |  |  |  |
| *GJB5* | 2.413721252 | 9.73E-20 | 1.17E-18 |  |  |  |  |
| *GOLT1A* | -4.584244417 | 5.10E-70 | 5.41E-68 |  |  |  |  |
| *GPA33* | -7.575953476 | 1.03E-163 | 7.54E-161 |  |  |  |  |
| *GSTM3* | 2.116082769 | 1.92E-15 | 1.70E-14 |  |  |  |  |
| *GUCA2A* | -7.797424079 | 2.43E-70 | 2.61E-68 |  |  |  |  |
| *GUCA2B* | -4.553765165 | 2.72E-26 | 4.94E-25 |  |  |  |  |
| *HAO2* | -2.229699632 | 2.12E-15 | 1.88E-14 |  |  |  |  |
| *HES2* | 2.942535316 | 1.60E-33 | 4.23E-32 |  |  |  |  |
| *HMGB4* | 2.824670865 | 0.000123774 | 0.000346751 |  |  |  |  |
| *HMGCS2* | -7.343163259 | 1.92E-76 | 2.60E-74 |  |  |  |  |
| *HSD3B2* | -2.040375257 | 1.75E-08 | 8.19E-08 |  |  |  |  |
| *HTR1D* | -3.089392828 | 6.46E-33 | 1.66E-31 |  |  |  |  |
| *IFI16* | 2.066877791 | 6.11E-60 | 4.65E-58 |  |  |  |  |
| *IGFN1* | 2.099706429 | 3.20E-12 | 2.18E-11 |  |  |  |  |
| *IGSF3* | 2.170457339 | 6.12E-60 | 4.65E-58 |  |  |  |  |
| *IL12RB2* | 2.709268701 | 1.44E-30 | 3.23E-29 |  |  |  |  |
| *IL20* | 3.225130613 | 7.86E-27 | 1.48E-25 |  |  |  |  |
| *ITLN1* | -7.16449287 | 1.47E-83 | 2.52E-81 |  |  |  |  |
| *KCNA10* | -2.922506328 | 2.22E-12 | 1.53E-11 |  |  |  |  |
| *KCNH1* | 2.887632104 | 5.78E-31 | 1.34E-29 |  |  |  |  |
| *KIAA1324* | -2.297313314 | 1.04E-15 | 9.46E-15 |  |  |  |  |
| *KISS1* | -2.548027317 | 9.83E-16 | 8.93E-15 |  |  |  |  |
| *KLHDC7A* | -3.592888429 | 1.70E-30 | 3.81E-29 |  |  |  |  |
| *LCE1A* | 3.376868694 | 6.96E-07 | 2.67E-06 |  |  |  |  |
| *LCE1B* | 3.631330875 | 6.87E-18 | 7.33E-17 |  |  |  |  |
| *LCE1C* | 4.402940901 | 1.03E-34 | 2.90E-33 |  |  |  |  |
| *LCE1D* | 2.382183311 | 0.000358127 | 0.00092709 |  |  |  |  |
| *LCE1F* | 4.485402753 | 1.23E-19 | 1.47E-18 |  |  |  |  |
| *LCE2A* | 3.028430111 | 1.17E-05 | 3.80E-05 |  |  |  |  |
| *LCE2B* | 3.375252995 | 2.67E-07 | 1.08E-06 |  |  |  |  |
| *LCE2D* | 2.42254766 | 0.000541223 | 0.001359362 |  |  |  |  |
| *LCE3A* | 3.305460512 | 1.13E-09 | 5.98E-09 |  |  |  |  |
| *LCE3C* | 4.746954221 | 1.89E-12 | 1.31E-11 |  |  |  |  |
| *LCE3D* | 4.404932204 | 5.45E-18 | 5.83E-17 |  |  |  |  |
| *LCE3E* | 3.980577039 | 2.34E-15 | 2.07E-14 |  |  |  |  |
| *LCE5A* | 2.560026668 | 7.27E-09 | 3.55E-08 |  |  |  |  |
| *LCE6A* | 2.800788866 | 3.44E-05 | 0.000104704 |  |  |  |  |
| *LEFTY1* | -5.842177761 | 2.05E-76 | 2.77E-74 |  |  |  |  |
| *LELP1* | 3.25311544 | 1.02E-08 | 4.87E-08 |  |  |  |  |
| *LHX9* | 2.104102414 | 1.43E-07 | 5.97E-07 |  |  |  |  |
| *LINC00303* | 2.253254964 | 1.60E-07 | 6.65E-07 |  |  |  |  |
| *LOC100129269* | 3.462982143 | 4.40E-38 | 1.44E-36 |  |  |  |  |
| *LOC100133445* | -2.038040913 | 3.59E-57 | 2.45E-55 |  |  |  |  |
| *LOC115110* | -3.309396093 | 4.61E-67 | 4.42E-65 |  |  |  |  |
| *LOC127841* | -3.736304753 | 4.00E-62 | 3.34E-60 |  |  |  |  |
| *LOC149086* | -2.575377863 | 1.02E-11 | 6.62E-11 |  |  |  |  |
| *LOC254099* | -4.236366833 | 1.06E-71 | 1.20E-69 |  |  |  |  |
| *LOC284551* | -2.488557423 | 3.24E-14 | 2.62E-13 |  |  |  |  |
| *LOC284688* | 2.042284726 | 2.44E-09 | 1.25E-08 |  |  |  |  |
| *LOC339442* | 4.024373313 | 3.42E-17 | 3.48E-16 |  |  |  |  |
| *LOC339535* | 2.481064089 | 1.25E-09 | 6.63E-09 |  |  |  |  |
| *LOC440704* | 2.653955394 | 0.001614913 | 0.003728601 |  |  |  |  |
| *LOC641515* | 2.099936692 | 3.39E-19 | 3.92E-18 |  |  |  |  |
| *LOC646627* | -3.312928815 | 1.18E-12 | 8.28E-12 |  |  |  |  |
| *LOR* | 4.758751523 | 4.32E-39 | 1.49E-37 |  |  |  |  |
| *LPAR3* | 3.664785184 | 1.24E-41 | 4.77E-40 |  |  |  |  |
| *LRRC38* | 4.198304614 | 8.10E-41 | 3.01E-39 |  |  |  |  |
| *MARK1* | 2.064623815 | 2.17E-32 | 5.40E-31 |  |  |  |  |
| *METTL11B* | 2.405439164 | 1.45E-19 | 1.73E-18 |  |  |  |  |
| *MFSD4* | -2.53561582 | 9.19E-28 | 1.81E-26 |  |  |  |  |
| *MGC4473* | 4.413111835 | 3.62E-31 | 8.43E-30 |  |  |  |  |
| *MIR1231* | 2.202573611 | 2.33E-23 | 3.52E-22 |  |  |  |  |
| *MIR205HG* | 4.692129485 | 4.56E-38 | 1.49E-36 |  |  |  |  |
| *MIR4251* | -3.052714043 | 2.33E-06 | 8.33E-06 |  |  |  |  |
| *MIR4422* | 2.168302209 | 0.013975217 | 0.026567582 |  |  |  |  |
| *MIR4671* | 3.654096457 | 5.19E-14 | 4.13E-13 |  |  |  |  |
| *MIR556* | -2.632270119 | 9.25E-10 | 4.95E-09 |  |  |  |  |
| *MLLT11* | 2.084448298 | 2.91E-24 | 4.67E-23 |  |  |  |  |
| *MST1P9* | -2.351404981 | 2.70E-25 | 4.61E-24 |  |  |  |  |
| *MTMR11* | -2.126831517 | 1.47E-55 | 9.54E-54 |  |  |  |  |
| *MUC1* | -3.130091569 | 3.37E-57 | 2.31E-55 |  |  |  |  |
| *Mir_584.3* | 2.362187052 | 0.003450738 | 0.007464784 |  |  |  |  |
| *NBPF7* | -2.741494904 | 2.06E-11 | 1.30E-10 |  |  |  |  |
| *NGF* | 2.686829852 | 3.80E-31 | 8.82E-30 |  |  |  |  |
| *NPPB* | -2.056850995 | 0.000307321 | 0.000804461 |  |  |  |  |
| *NR0B2* | -8.483979611 | 4.63E-203 | 7.40E-200 |  |  |  |  |
| *NR5A2* | -3.627219725 | 5.40E-92 | 1.15E-89 |  |  |  |  |
| *OR10K2* | 2.530189791 | 0.001902691 | 0.004331196 |  |  |  |  |
| *OR2L13* | 2.274655716 | 1.46E-05 | 4.68E-05 |  |  |  |  |
| *OR2L1P* | 2.222171003 | 0.001762305 | 0.004040406 |  |  |  |  |
| *OR2L2* | 2.27172452 | 0.000673229 | 0.001664447 |  |  |  |  |
| *OR2L3* | 2.188239937 | 0.010346117 | 0.020282344 |  |  |  |  |
| *OR6K2* | 2.890702517 | 0.001570037 | 0.003634565 |  |  |  |  |
| *OR6Y1* | 3.281041579 | 0.000157611 | 0.000433585 |  |  |  |  |
| *PADI3* | 4.098621695 | 7.74E-37 | 2.40E-35 |  |  |  |  |
| *PAPPA2* | 2.14235691 | 7.95E-12 | 5.20E-11 |  |  |  |  |
| *PAX7* | 4.872884054 | 8.28E-20 | 1.00E-18 |  |  |  |  |
| *PCP4L1* | 2.244020664 | 1.15E-12 | 8.11E-12 |  |  |  |  |
| *PDPN* | 2.010804031 | 1.21E-28 | 2.49E-27 |  |  |  |  |
| *PDZK1.1* | -3.384902957 | 9.16E-46 | 4.16E-44 |  |  |  |  |
| *PGBD5* | 2.614142447 | 2.07E-25 | 3.56E-24 |  |  |  |  |
| *PGLYRP3* | 2.268600906 | 4.10E-10 | 2.28E-09 |  |  |  |  |
| *PGLYRP4* | 3.674082232 | 2.11E-30 | 4.70E-29 |  |  |  |  |
| *PIGR* | -6.492578187 | 4.72E-73 | 5.70E-71 |  |  |  |  |
| *PKP1* | 3.470854705 | 5.01E-27 | 9.55E-26 |  |  |  |  |
| *PLA2G2A* | -4.593738509 | 1.59E-36 | 4.88E-35 |  |  |  |  |
| *PLA2G2E* | -2.241144845 | 8.11E-05 | 0.000233587 |  |  |  |  |
| *PLCH2* | 2.138496648 | 4.90E-21 | 6.40E-20 |  |  |  |  |
| *PLD5* | 4.184261287 | 5.62E-22 | 7.81E-21 |  |  |  |  |
| *PLEKHA6* | -3.698300231 | 2.17E-95 | 4.92E-93 |  |  |  |  |
| *POU3F1* | 3.39872375 | 3.53E-29 | 7.45E-28 |  |  |  |  |
| *PPFIA4* | 2.365346135 | 1.99E-34 | 5.50E-33 |  |  |  |  |
| *PRAMEF1* | 2.515566843 | 0.00661562 | 0.013527015 |  |  |  |  |
| *PRAMEF2* | 2.069208155 | 0.009015094 | 0.017877513 |  |  |  |  |
| *PRAMEF4* | 2.601399792 | 0.000225372 | 0.000603078 |  |  |  |  |
| *PRAMEF8.1* | 2.83968017 | 0.000606035 | 0.001507789 |  |  |  |  |
| *PRDM16* | -2.897394606 | 1.09E-39 | 3.88E-38 |  |  |  |  |
| *PROX1-AS1* | -2.499080732 | 4.47E-12 | 3.00E-11 |  |  |  |  |
| *PROX1* | -2.752597647 | 1.31E-24 | 2.16E-23 |  |  |  |  |
| *PRR9* | 5.714825827 | 5.60E-30 | 1.23E-28 |  |  |  |  |
| *PRSS38* | 2.38158781 | 4.23E-10 | 2.35E-09 |  |  |  |  |
| *RBSG4* | 2.431432203 | 8.30E-08 | 3.57E-07 |  |  |  |  |
| *REG4* | -10.52910635 | 4.14E-153 | 2.74E-150 |  |  |  |  |
| *RGS7* | -2.979006487 | 4.93E-17 | 4.97E-16 |  |  |  |  |
| *RHOU* | -2.180777604 | 1.23E-51 | 6.98E-50 |  |  |  |  |
| *RN7SK.10* | 4.048302282 | 3.36E-13 | 2.49E-12 |  |  |  |  |
| *RNF186* | -8.188854785 | 1.02E-150 | 6.42E-148 |  |  |  |  |
| *RORC* | -2.873919061 | 1.31E-28 | 2.70E-27 |  |  |  |  |
| *RPE65* | 2.230336704 | 5.33E-10 | 2.94E-09 |  |  |  |  |
| *RPTN* | 4.050392669 | 1.15E-15 | 1.04E-14 |  |  |  |  |
| *RXFP4* | -3.567778765 | 1.73E-37 | 5.52E-36 |  |  |  |  |
| *S100A12* | 2.405876092 | 3.56E-12 | 2.41E-11 |  |  |  |  |
| *S100A2* | 2.8715727 | 1.05E-19 | 1.26E-18 |  |  |  |  |
| *S100A7A* | 2.863451065 | 7.19E-10 | 3.90E-09 |  |  |  |  |
| *SAMD13* | -2.170183585 | 6.02E-31 | 1.39E-29 |  |  |  |  |
| *SEC16B* | -2.119275846 | 1.11E-44 | 4.85E-43 |  |  |  |  |
| *SELENBP1* | -2.548213616 | 1.34E-32 | 3.36E-31 |  |  |  |  |
| *SFN.1* | 2.418225969 | 1.87E-31 | 4.43E-30 |  |  |  |  |
| *SFN.2* | 2.428520026 | 6.04E-32 | 1.47E-30 |  |  |  |  |
| *SH2D5* | 2.705198149 | 7.14E-27 | 1.35E-25 |  |  |  |  |
| *SHCBP1L* | 2.079265074 | 7.37E-05 | 0.000213715 |  |  |  |  |
| *SLAMF9* | 2.009698641 | 2.52E-14 | 2.06E-13 |  |  |  |  |
| *SLC1A7* | -3.177054428 | 7.82E-41 | 2.91E-39 |  |  |  |  |
| *SLC2A1* | 2.212437497 | 1.42E-35 | 4.16E-34 |  |  |  |  |
| *SLC30A10* | -2.326402919 | 4.25E-12 | 2.86E-11 |  |  |  |  |
| *SLC30A2* | -3.312498452 | 4.74E-20 | 5.82E-19 |  |  |  |  |
| *SLC35F3* | 4.326786552 | 2.00E-55 | 1.29E-53 |  |  |  |  |
| *SLC44A3* | -2.450466852 | 1.37E-65 | 1.27E-63 |  |  |  |  |
| *SLC5A9* | -2.866658754 | 8.88E-33 | 2.26E-31 |  |  |  |  |
| *SMCP* | 4.942196434 | 1.43E-20 | 1.81E-19 |  |  |  |  |
| *SMPDL3B* | -2.763876643 | 2.09E-40 | 7.63E-39 |  |  |  |  |
| *SPRR2G* | 2.878149488 | 1.94E-08 | 9.04E-08 |  |  |  |  |
| *SPRR4* | 5.582373883 | 1.30E-30 | 2.94E-29 |  |  |  |  |
| *SUSD4* | 2.635769553 | 1.45E-26 | 2.69E-25 |  |  |  |  |
| *SYT14* | 2.274265895 | 2.30E-13 | 1.73E-12 |  |  |  |  |
| *TCHHL1* | 4.328788208 | 7.00E-25 | 1.17E-23 |  |  |  |  |
| *TCHH* | 4.242227922 | 3.85E-46 | 1.80E-44 |  |  |  |  |
| *TMCC2* | 2.512709037 | 9.40E-62 | 7.71E-60 |  |  |  |  |
| *TMEM125* | -2.685456308 | 1.82E-57 | 1.26E-55 |  |  |  |  |
| *TMEM63A* | -2.071633874 | 2.67E-88 | 5.25E-86 |  |  |  |  |
| *TMEM82* | -6.45581928 | 1.66E-99 | 4.09E-97 |  |  |  |  |
| *TNFRSF18* | 2.263585545 | 1.73E-32 | 4.32E-31 |  |  |  |  |
| *TP73* | 2.103342407 | 2.96E-30 | 6.56E-29 |  |  |  |  |
| *TRNA_Asn.24* | -2.536451922 | 0.001560226 | 0.003613085 |  |  |  |  |
| *TRNA_Pseudo.19* | 2.732767483 | 7.59E-05 | 0.000219539 |  |  |  |  |
| *TRNA_Val.4* | 2.135862148 | 0.005404999 | 0.011241174 |  |  |  |  |
| *TRNP1* | -2.527204452 | 2.25E-35 | 6.52E-34 |  |  |  |  |
| *TSPAN1* | -2.69945831 | 2.55E-28 | 5.17E-27 |  |  |  |  |
| *U6.5* | -3.795158651 | 1.78E-19 | 2.11E-18 |  |  |  |  |
| *U6.8* | -2.713349431 | 3.71E-16 | 3.47E-15 |  |  |  |  |
| *UBXN10* | -2.767028418 | 3.71E-32 | 9.15E-31 |  |  |  |  |
| *VANGL2* | 2.555064781 | 1.51E-43 | 6.29E-42 |  |  |  |  |
| *VWA5B1* | -4.209251968 | 7.97E-30 | 1.74E-28 |  |  |  |  |
| *WNT2B* | 2.125042808 | 2.80E-28 | 5.66E-27 |  |  |  |  |
| *WNT3A* | 5.220546434 | 5.55E-104 | 1.60E-101 |  |  |  |  |
| *WNT9A* | 2.722996538 | 6.50E-46 | 3.00E-44 |  |  |  |  |
| *Y_RNA.7* | 2.108346589 | 0.01767953 | 0.032779287 |  |  |  |  |
| *ZBTB8B* | 2.205472104 | 6.30E-08 | 2.75E-07 |  |  |  |  |
| *ZP4* | 4.116398505 | 1.71E-17 | 1.78E-16 |  |  |  |  |
| *ZYG11A* | 2.695007872 | 1.16E-28 | 2.39E-27 |  |  |  |  |
| *A1CF* | -7.927467066 | 1.98E-187 | 2.24E-184 |  |  |  |  |
| *ACSL5* | -3.328919845 | 3.06E-69 | 3.15E-67 |  |  |  |  |
| *ADARB2-AS1* | 2.122303342 | 1.41E-08 | 6.67E-08 |  |  |  |  |
| *ADARB2* | 2.175181358 | 6.27E-14 | 4.95E-13 |  |  |  |  |
| *ADRA2A* | -2.627808448 | 3.50E-20 | 4.33E-19 |  |  |  |  |
| *AF086154* | -2.34333353 | 2.97E-08 | 1.35E-07 |  |  |  |  |
| *AGAP11* | 2.017247065 | 7.16E-15 | 6.09E-14 |  |  |  |  |
| *AK128534* | 2.842461161 | 3.18E-22 | 4.50E-21 |  |  |  |  |
| *AK297683* | -2.713172628 | 5.42E-11 | 3.29E-10 |  |  |  |  |
| *AKR1C2* | 4.332576994 | 1.06E-47 | 5.31E-46 |  |  |  |  |
| *AKR1C3* | 2.969507293 | 3.43E-28 | 6.91E-27 |  |  |  |  |
| *AKR1C4* | -3.147357551 | 7.17E-26 | 1.28E-24 |  |  |  |  |
| *ANKRD1* | -2.251228297 | 2.12E-14 | 1.73E-13 |  |  |  |  |
| *ANKRD2* | 2.311960489 | 4.99E-24 | 7.87E-23 |  |  |  |  |
| *ANKRD30A* | 2.012908531 | 0.000257643 | 0.000683174 |  |  |  |  |
| *ANXA8L1.1* | 2.832024665 | 8.85E-17 | 8.71E-16 |  |  |  |  |
| *ANXA8L1.2* | 3.119588421 | 8.36E-39 | 2.83E-37 |  |  |  |  |
| *ANXA8L2* | 2.782877979 | 3.83E-23 | 5.74E-22 |  |  |  |  |
| *ARMC3* | -2.754010135 | 1.83E-17 | 1.90E-16 |  |  |  |  |
| *ASAH2* | -2.964951032 | 1.44E-20 | 1.83E-19 |  |  |  |  |
| *BC036309* | 2.14335316 | 0.006074945 | 0.012519492 |  |  |  |  |
| *BC037970* | 6.398931537 | 9.08E-33 | 2.31E-31 |  |  |  |  |
| *BC039000* | 2.702044717 | 1.67E-10 | 9.65E-10 |  |  |  |  |
| *BC051760* | 3.987894188 | 1.68E-31 | 4.00E-30 |  |  |  |  |
| *BC065757* | -2.802625316 | 7.44E-07 | 2.84E-06 |  |  |  |  |
| *BC132944* | 2.250808464 | 0.000138619 | 0.000385319 |  |  |  |  |
| *BNIP3* | 2.585181924 | 5.68E-44 | 2.41E-42 |  |  |  |  |
| *BTBD16* | -2.031213242 | 1.12E-17 | 1.18E-16 |  |  |  |  |
| *C10orf108* | -4.367827273 | 1.54E-114 | 5.09E-112 |  |  |  |  |
| *C10orf112* | -2.308209908 | 1.32E-09 | 6.99E-09 |  |  |  |  |
| *C10orf122* | 2.285234586 | 0.000297854 | 0.000781788 |  |  |  |  |
| *C10orf136* | -2.047672695 | 1.49E-09 | 7.82E-09 |  |  |  |  |
| *C10orf81* | -3.749298069 | 1.93E-33 | 5.06E-32 |  |  |  |  |
| *C10orf90* | 2.530222059 | 3.14E-16 | 2.97E-15 |  |  |  |  |
| *C10orf91* | 2.234438219 | 3.08E-31 | 7.18E-30 |  |  |  |  |
| *C10orf99* | 2.218782906 | 7.34E-07 | 2.80E-06 |  |  |  |  |
| *CALHM3* | -3.420973671 | 5.70E-20 | 6.95E-19 |  |  |  |  |
| *CALML3* | 3.147654106 | 1.69E-15 | 1.51E-14 |  |  |  |  |
| *CALML5* | 3.373007535 | 3.62E-14 | 2.91E-13 |  |  |  |  |
| *CDHR1* | 3.017330846 | 1.80E-20 | 2.27E-19 |  |  |  |  |
| *CHAT* | 3.130257731 | 8.95E-10 | 4.80E-09 |  |  |  |  |
| *CLRN3* | -8.720161848 | 3.50E-246 | 1.06E-242 |  |  |  |  |
| *COL17A1* | 3.097216688 | 3.01E-31 | 7.03E-30 |  |  |  |  |
| *CYP26A1* | 4.84051778 | 3.45E-37 | 1.09E-35 |  |  |  |  |
| *CYP2C9* | -2.271732982 | 4.64E-13 | 3.39E-12 |  |  |  |  |
| *CYP2E1* | -2.203331657 | 3.42E-14 | 2.76E-13 |  |  |  |  |
| *DMBT1* | -6.927893157 | 1.42E-70 | 1.54E-68 |  |  |  |  |
| *DNAJC12* | -2.069249559 | 3.95E-16 | 3.70E-15 |  |  |  |  |
| *DPYSL4* | 2.153915157 | 1.23E-16 | 1.19E-15 |  |  |  |  |
| *DRGX* | -4.97826233 | 4.50E-25 | 7.60E-24 |  |  |  |  |
| *DUSP13* | 2.645910825 | 8.26E-17 | 8.16E-16 |  |  |  |  |
| *FAM25A* | 2.305436869 | 1.35E-07 | 5.64E-07 |  |  |  |  |
| *FAM25B.1* | 2.845731034 | 1.85E-09 | 9.62E-09 |  |  |  |  |
| *FAM25B.2* | 2.986328292 | 1.23E-09 | 6.50E-09 |  |  |  |  |
| *FAM25B.3* | 2.971372353 | 4.17E-14 | 3.34E-13 |  |  |  |  |
| *FLJ41350* | 2.228907465 | 3.25E-05 | 9.93E-05 |  |  |  |  |
| *FXYD4* | -4.099649557 | 5.02E-37 | 1.57E-35 |  |  |  |  |
| *GUCY2GP* | -2.127051263 | 4.91E-08 | 2.18E-07 |  |  |  |  |
| *H2AFY2* | 2.254978804 | 1.99E-35 | 5.79E-34 |  |  |  |  |
| *HABP2* | -6.847185454 | 1.36E-130 | 6.25E-128 |  |  |  |  |
| *HKDC1* | -4.567328156 | 4.31E-62 | 3.57E-60 |  |  |  |  |
| *HPSE2* | 2.032317349 | 6.60E-11 | 3.96E-10 |  |  |  |  |
| *HTR7* | 3.571783984 | 2.38E-44 | 1.03E-42 |  |  |  |  |
| *INA* | 3.487104734 | 4.59E-28 | 9.20E-27 |  |  |  |  |
| *ITIH2* | -2.593839159 | 1.68E-23 | 2.57E-22 |  |  |  |  |
| *JAKMIP3* | 2.53368938 | 1.18E-20 | 1.50E-19 |  |  |  |  |
| *KCNK18* | 2.168809217 | 0.000902935 | 0.002187758 |  |  |  |  |
| *KCNMA1* | 2.262433921 | 4.50E-18 | 4.85E-17 |  |  |  |  |
| *LIPF* | -7.13635709 | 3.26E-50 | 1.78E-48 |  |  |  |  |
| *LIPJ* | 2.002832116 | 1.06E-10 | 6.25E-10 |  |  |  |  |
| *LIPK* | 2.628138636 | 6.47E-11 | 3.88E-10 |  |  |  |  |
| *LOC100507127* | 2.257213411 | 8.16E-11 | 4.85E-10 |  |  |  |  |
| *LOC170425* | -2.390050158 | 1.44E-20 | 1.82E-19 |  |  |  |  |
| *LOC439990* | -2.285769413 | 1.10E-31 | 2.65E-30 |  |  |  |  |
| *LOC728978* | 2.129985162 | 8.36E-17 | 8.26E-16 |  |  |  |  |
| *LOC731789* | -3.550303166 | 7.45E-17 | 7.39E-16 |  |  |  |  |
| *LOC84856* | 2.001194047 | 5.69E-19 | 6.50E-18 |  |  |  |  |
| *LOXL4* | 2.316466022 | 4.70E-18 | 5.05E-17 |  |  |  |  |
| *LRIT2* | 2.477420929 | 1.83E-10 | 1.06E-09 |  |  |  |  |
| *M1* | -2.285172027 | 1.69E-08 | 7.90E-08 |  |  |  |  |
| *MIR4483* | -3.192735862 | 8.54E-19 | 9.64E-18 |  |  |  |  |
| *MIR4675* | 3.112064335 | 9.71E-07 | 3.65E-06 |  |  |  |  |
| *MIR4681* | -2.789230326 | 9.04E-07 | 3.41E-06 |  |  |  |  |
| *MIR608* | -3.754164374 | 5.40E-26 | 9.65E-25 |  |  |  |  |
| *MIR936* | 3.103648166 | 1.19E-28 | 2.45E-27 |  |  |  |  |
| *MSMB* | -2.306015487 | 2.43E-10 | 1.38E-09 |  |  |  |  |
| *MTRNR2L5* | 2.176599917 | 0.002800533 | 0.006178275 |  |  |  |  |
| *MYO3A* | 2.312469244 | 4.16E-10 | 2.31E-09 |  |  |  |  |
| *Mir_544.7* | 2.683039419 | 0.002763279 | 0.006105014 |  |  |  |  |
| *Mir_548.5* | -2.012080204 | 3.31E-05 | 0.00010102 |  |  |  |  |
| *NEUROG3* | -7.59176446 | 3.17E-69 | 3.25E-67 |  |  |  |  |
| *NKX1-2* | 4.487232154 | 4.62E-34 | 1.25E-32 |  |  |  |  |
| *NRAP* | -5.327975776 | 1.56E-71 | 1.76E-69 |  |  |  |  |
| *NRG3* | -2.42343082 | 1.78E-15 | 1.59E-14 |  |  |  |  |
| *O3FAR1* | -3.473895115 | 1.82E-46 | 8.65E-45 |  |  |  |  |
| *OIT3* | -2.160229945 | 1.09E-30 | 2.48E-29 |  |  |  |  |
| *PBLD* | -2.042859274 | 7.84E-56 | 5.14E-54 |  |  |  |  |
| *PITX3* | 2.549593938 | 1.57E-20 | 1.98E-19 |  |  |  |  |
| *PLA2G12B* | -5.59094912 | 4.26E-45 | 1.90E-43 |  |  |  |  |
| *PNLIPRP1* | -2.362728643 | 5.64E-11 | 3.41E-10 |  |  |  |  |
| *PNLIPRP2* | -5.84241263 | 3.14E-39 | 1.09E-37 |  |  |  |  |
| *PNLIPRP3* | 3.595614983 | 1.54E-12 | 1.08E-11 |  |  |  |  |
| *PPAPDC1A* | 2.344949126 | 8.16E-19 | 9.23E-18 |  |  |  |  |
| *PPYR1* | -2.618287799 | 2.94E-24 | 4.70E-23 |  |  |  |  |
| *RBP4* | -3.917204566 | 5.50E-32 | 1.34E-30 |  |  |  |  |
| *RN7SK.13* | 2.023810328 | 0.014441557 | 0.027366025 |  |  |  |  |
| *SEMA4G* | -3.899589409 | 1.21E-192 | 1.64E-189 |  |  |  |  |
| *SFTPA2* | -3.642456494 | 4.69E-29 | 9.81E-28 |  |  |  |  |
| *SLC18A3* | 2.950266569 | 8.07E-09 | 3.92E-08 |  |  |  |  |
| *SORCS3* | 2.319836587 | 4.68E-08 | 2.08E-07 |  |  |  |  |
| *TCERG1L* | 2.480481647 | 5.04E-12 | 3.36E-11 |  |  |  |  |
| *TECTB* | -3.533355165 | 3.59E-16 | 3.37E-15 |  |  |  |  |
| *TLX1NB* | -3.116661037 | 3.77E-19 | 4.34E-18 |  |  |  |  |
| *TMEM236.1* | -2.27963247 | 6.94E-11 | 4.15E-10 |  |  |  |  |
| *TMEM72* | -2.238766817 | 1.29E-08 | 6.10E-08 |  |  |  |  |
| *TSPAN15* | -2.124034206 | 1.82E-55 | 1.17E-53 |  |  |  |  |
| *TTC40* | -2.320603743 | 1.58E-16 | 1.53E-15 |  |  |  |  |
| *U6.21* | -2.203027199 | 0.004802785 | 0.010086776 |  |  |  |  |
| *UCN3* | -4.930344379 | 9.22E-21 | 1.19E-19 |  |  |  |  |
| *VAX1* | 5.268831188 | 4.37E-39 | 1.51E-37 |  |  |  |  |
| *ZNF239* | -2.048791194 | 2.65E-34 | 7.29E-33 |  |  |  |  |
| *AB231721* | 2.21369558 | 7.24E-06 | 2.42E-05 |  |  |  |  |
| *AB231723* | 2.457178601 | 5.91E-19 | 6.73E-18 |  |  |  |  |
| *AB231784* | 2.270303057 | 0.001160195 | 0.002752892 |  |  |  |  |
| *AB429224* | -7.86611498 | 3.24E-181 | 3.25E-178 |  |  |  |  |
| *ABCC8* | -2.107695351 | 2.02E-09 | 1.05E-08 |  |  |  |  |
| *ACY3* | -3.059995284 | 4.77E-54 | 2.91E-52 |  |  |  |  |
| *AF085962* | -2.782735315 | 0.001805948 | 0.004129663 |  |  |  |  |
| *AK056982* | 3.00797936 | 2.40E-47 | 1.19E-45 |  |  |  |  |
| *AK091996* | -2.778563919 | 5.54E-34 | 1.50E-32 |  |  |  |  |
| *AK095081* | -7.816237183 | 1.81E-78 | 2.61E-76 |  |  |  |  |
| *AK128059* | 2.993993101 | 1.21E-08 | 5.77E-08 |  |  |  |  |
| *AK294973* | 2.232967227 | 8.70E-09 | 4.21E-08 |  |  |  |  |
| *ALDH3B1* | -2.46516771 | 1.29E-48 | 6.71E-47 |  |  |  |  |
| *ALDH3B2* | 2.445841369 | 1.07E-19 | 1.28E-18 |  |  |  |  |
| *ALX4* | 4.022768 | 1.20E-40 | 4.45E-39 |  |  |  |  |
| *ANO1* | 2.038301448 | 2.83E-19 | 3.30E-18 |  |  |  |  |
| *APOA1* | -4.376663996 | 8.95E-54 | 5.40E-52 |  |  |  |  |
| *APOA4* | -6.69578453 | 1.73E-33 | 4.55E-32 |  |  |  |  |
| *APOA5* | -2.042817134 | 5.88E-08 | 2.59E-07 |  |  |  |  |
| *APOC3* | -5.176556245 | 3.16E-25 | 5.37E-24 |  |  |  |  |
| *ASCL2* | -2.190881768 | 1.04E-17 | 1.10E-16 |  |  |  |  |
| *ASRGL1* | -2.224294337 | 2.04E-33 | 5.34E-32 |  |  |  |  |
| *AX747648* | -2.227725893 | 2.11E-46 | 1.00E-44 |  |  |  |  |
| *BC008359* | 3.279825242 | 1.65E-23 | 2.52E-22 |  |  |  |  |
| *BC016143.2* | 4.264325464 | 1.74E-27 | 3.38E-26 |  |  |  |  |
| *BC021736* | 3.17190305 | 2.64E-16 | 2.52E-15 |  |  |  |  |
| *BC031955* | 2.65765912 | 2.10E-09 | 1.09E-08 |  |  |  |  |
| *BC031979* | 2.57143029 | 3.71E-21 | 4.88E-20 |  |  |  |  |
| *BC133018* | 2.150309815 | 1.89E-16 | 1.81E-15 |  |  |  |  |
| *BTG4* | 4.252668724 | 6.92E-37 | 2.15E-35 |  |  |  |  |
| *BX649128* | 2.397767352 | 8.01E-46 | 3.67E-44 |  |  |  |  |
| *C11orf53* | -2.670897586 | 2.98E-16 | 2.81E-15 |  |  |  |  |
| *C11orf86* | -4.529648462 | 1.59E-30 | 3.56E-29 |  |  |  |  |
| *C11orf87* | 4.973511842 | 1.45E-40 | 5.33E-39 |  |  |  |  |
| *C11orf88* | 2.993685231 | 3.30E-27 | 6.33E-26 |  |  |  |  |
| *C11orf9* | -5.036702093 | 3.52E-167 | 2.65E-164 |  |  |  |  |
| *CABP4* | -2.21843965 | 5.32E-24 | 8.37E-23 |  |  |  |  |
| *CALCA* | -2.585734454 | 9.74E-07 | 3.66E-06 |  |  |  |  |
| *CAPN5* | -3.386406439 | 2.48E-80 | 3.82E-78 |  |  |  |  |
| *CARD18* | 3.470213539 | 1.29E-20 | 1.64E-19 |  |  |  |  |
| *CASP5* | -2.99839431 | 3.72E-36 | 1.12E-34 |  |  |  |  |
| *CDHR5* | -7.011220024 | 1.92E-275 | 8.70E-272 |  |  |  |  |
| *CNTN5* | 2.638754296 | 1.00E-14 | 8.43E-14 |  |  |  |  |
| *CREB3L1* | -3.493811061 | 7.24E-72 | 8.37E-70 |  |  |  |  |
| *DJ031150* | 2.743931976 | 7.89E-12 | 5.17E-11 |  |  |  |  |
| *DKFZP434K028* | -4.940660694 | 2.94E-76 | 3.96E-74 |  |  |  |  |
| *DM119532.2* | -4.774032764 | 1.17E-20 | 1.50E-19 |  |  |  |  |
| *DTX4* | -2.074154615 | 4.69E-59 | 3.44E-57 |  |  |  |  |
| *EFCAB4A* | -2.916019604 | 1.67E-57 | 1.16E-55 |  |  |  |  |
| *F2* | -3.371796699 | 5.64E-24 | 8.86E-23 |  |  |  |  |
| *FAM181B* | 4.252126766 | 2.35E-48 | 1.20E-46 |  |  |  |  |
| *FAM55A* | -3.658888084 | 2.22E-17 | 2.29E-16 |  |  |  |  |
| *FAM55B.2* | -2.414060113 | 1.38E-09 | 7.25E-09 |  |  |  |  |
| *FGF3* | 2.687513827 | 1.66E-10 | 9.61E-10 |  |  |  |  |
| *FLJ41423* | 4.210478817 | 7.73E-24 | 1.20E-22 |  |  |  |  |
| *FOLR1* | -2.003336627 | 1.54E-07 | 6.42E-07 |  |  |  |  |
| *FOLR3* | 3.503091218 | 1.18E-24 | 1.95E-23 |  |  |  |  |
| *FUT4* | -3.532368541 | 1.34E-135 | 6.59E-133 |  |  |  |  |
| *GAL3ST3* | 2.440313634 | 1.07E-09 | 5.68E-09 |  |  |  |  |
| *GIF* | -5.679562775 | 2.12E-42 | 8.46E-41 |  |  |  |  |
| *GLYATL2* | 2.426381668 | 1.32E-12 | 9.29E-12 |  |  |  |  |
| *GRAMD1B.1* | -2.769426961 | 2.94E-29 | 6.23E-28 |  |  |  |  |
| *GRAMD1B.2* | -3.086502005 | 8.10E-46 | 3.71E-44 |  |  |  |  |
| *GRM5* | 3.167426795 | 1.73E-26 | 3.19E-25 |  |  |  |  |
| *GUCY2E* | 3.733686291 | 2.14E-20 | 2.69E-19 |  |  |  |  |
| *HBE1* | 2.134454405 | 1.24E-05 | 4.02E-05 |  |  |  |  |
| *INSC* | -3.164963519 | 2.35E-44 | 1.02E-42 |  |  |  |  |
| *JA429539* | -2.538437069 | 3.57E-32 | 8.81E-31 |  |  |  |  |
| *KCNE3* | -2.651625534 | 1.95E-27 | 3.80E-26 |  |  |  |  |
| *KCNQ1* | -2.9866508 | 3.41E-58 | 2.43E-56 |  |  |  |  |
| *KCTD14* | -2.273235159 | 6.00E-27 | 1.14E-25 |  |  |  |  |
| *KIRREL3-AS3* | 2.046371378 | 0.001538868 | 0.003566969 |  |  |  |  |
| *KRTAP5-1* | -2.645856354 | 1.14E-38 | 3.83E-37 |  |  |  |  |
| *KRTAP5-2* | -2.767805658 | 7.26E-38 | 2.36E-36 |  |  |  |  |
| *KRTAP5-4* | -2.453619849 | 8.10E-14 | 6.33E-13 |  |  |  |  |
| *KRTAP5-5* | -5.567055417 | 8.91E-46 | 4.06E-44 |  |  |  |  |
| *LMO1* | 4.56643863 | 3.30E-48 | 1.68E-46 |  |  |  |  |
| *LOC100506305* | 2.909200924 | 9.68E-24 | 1.50E-22 |  |  |  |  |
| *LOC283177* | -6.212303792 | 6.92E-60 | 5.23E-58 |  |  |  |  |
| *LOC338651* | -2.692233232 | 1.76E-41 | 6.72E-40 |  |  |  |  |
| *LOC440040* | 3.66219731 | 5.35E-18 | 5.74E-17 |  |  |  |  |
| *LOC643037* | -2.499490697 | 9.28E-08 | 3.96E-07 |  |  |  |  |
| *MIR192* | -7.715045874 | 1.07E-80 | 1.68E-78 |  |  |  |  |
| *MIR194-2* | -5.937755922 | 2.57E-32 | 6.38E-31 |  |  |  |  |
| *MIR326* | -2.618010365 | 1.43E-09 | 7.51E-09 |  |  |  |  |
| *MIR708* | 3.205616819 | 2.44E-10 | 1.39E-09 |  |  |  |  |
| *MMP10* | 2.70246893 | 1.63E-20 | 2.06E-19 |  |  |  |  |
| *MMP13* | 5.119707556 | 3.58E-55 | 2.29E-53 |  |  |  |  |
| *MMP7* | -2.232336589 | 3.98E-12 | 2.69E-11 |  |  |  |  |
| *MOGAT2* | -2.600819448 | 4.42E-11 | 2.70E-10 |  |  |  |  |
| *MPPED2* | 2.18907846 | 2.76E-20 | 3.43E-19 |  |  |  |  |
| *MRGPRX2* | 2.90804942 | 2.19E-11 | 1.37E-10 |  |  |  |  |
| *MRGPRX3* | 4.445505735 | 8.43E-29 | 1.75E-27 |  |  |  |  |
| *MS4A10* | -2.197292902 | 6.55E-07 | 2.52E-06 |  |  |  |  |
| *MS4A8B* | -5.842511916 | 6.17E-58 | 4.33E-56 |  |  |  |  |
| *MUC2* | -7.614585688 | 2.39E-79 | 3.58E-77 |  |  |  |  |
| *MUC5AC* | -8.018037006 | 5.73E-77 | 7.91E-75 |  |  |  |  |
| *MUC5B* | -3.610503614 | 6.40E-20 | 7.79E-19 |  |  |  |  |
| *MUC6* | -6.565052781 | 6.16E-63 | 5.31E-61 |  |  |  |  |
| *MYOD1* | 2.480439473 | 9.69E-05 | 0.000275652 |  |  |  |  |
| *Mir_548.8* | 2.506586162 | 8.71E-10 | 4.67E-09 |  |  |  |  |
| *NLRP10* | 4.471518856 | 1.56E-32 | 3.91E-31 |  |  |  |  |
| *NLRP6* | -3.407551039 | 8.59E-36 | 2.54E-34 |  |  |  |  |
| *ODZ4.1* | 2.776980625 | 3.78E-42 | 1.49E-40 |  |  |  |  |
| *ODZ4.2* | 2.897989177 | 2.10E-24 | 3.39E-23 |  |  |  |  |
| *OMP* | -3.736729199 | 1.09E-53 | 6.56E-52 |  |  |  |  |
| *OR10A3* | 2.61874161 | 1.21E-06 | 4.51E-06 |  |  |  |  |
| *OR10A6* | 2.225212339 | 8.91E-05 | 0.00025499 |  |  |  |  |
| *OR10AG1* | 2.151420426 | 0.00584996 | 0.012102659 |  |  |  |  |
| *OR10S1* | 4.575272634 | 1.97E-12 | 1.36E-11 |  |  |  |  |
| *OR4C12* | 2.069040846 | 0.022251872 | 0.040299624 |  |  |  |  |
| *OR4C3* | 2.0805532 | 0.017258699 | 0.032062491 |  |  |  |  |
| *OR4D11* | 2.008327346 | 0.016578809 | 0.03095182 |  |  |  |  |
| *OR4D5* | 2.193107337 | 0.008286849 | 0.016582244 |  |  |  |  |
| *OR52E4* | 2.435760349 | 0.003051455 | 0.00667707 |  |  |  |  |
| *OR52J3* | 2.116564537 | 0.02743609 | 0.048580943 |  |  |  |  |
| *OR52L1* | 2.14696448 | 8.24E-06 | 2.73E-05 |  |  |  |  |
| *OR52W1* | -2.136262071 | 7.29E-12 | 4.79E-11 |  |  |  |  |
| *OR56A1* | 2.982851019 | 1.22E-06 | 4.54E-06 |  |  |  |  |
| *OR56A3* | 3.103451504 | 3.66E-06 | 1.28E-05 |  |  |  |  |
| *OR56A4* | 2.982978437 | 1.01E-06 | 3.78E-06 |  |  |  |  |
| *OR56A5* | 2.253371274 | 2.52E-07 | 1.02E-06 |  |  |  |  |
| *OR5A1* | 3.2862077 | 0.000133566 | 0.000372225 |  |  |  |  |
| *OR5AK4P* | -2.396722039 | 2.37E-07 | 9.66E-07 |  |  |  |  |
| *OR5AP2* | 2.59681273 | 0.000383114 | 0.000986883 |  |  |  |  |
| *OR5J2* | 3.008731858 | 0.000559163 | 0.001399117 |  |  |  |  |
| *OR5P3* | 2.168136994 | 0.000132954 | 0.000370595 |  |  |  |  |
| *OR5T3* | 2.659169138 | 0.007709983 | 0.015550478 |  |  |  |  |
| *OR5W2* | 2.357601344 | 0.022144261 | 0.040129127 |  |  |  |  |
| *OR8H1* | 2.930412118 | 0.001293006 | 0.003041195 |  |  |  |  |
| *OR8K1* | 2.847195168 | 0.001694806 | 0.003895845 |  |  |  |  |
| *OR8K5* | 2.604591414 | 0.005146109 | 0.010745518 |  |  |  |  |
| *OR9G9* | 2.220378366 | 0.026000572 | 0.046304677 |  |  |  |  |
| *OR9Q2* | 2.006056898 | 0.02826716 | 0.049821948 |  |  |  |  |
| *P2RX3* | -3.813369558 | 5.00E-24 | 7.88E-23 |  |  |  |  |
| *PDZD3* | -4.729072718 | 1.06E-92 | 2.28E-90 |  |  |  |  |
| *PGA3* | 6.256756511 | 3.26E-15 | 2.85E-14 |  |  |  |  |
| *PGA4* | 4.251305089 | 1.61E-07 | 6.67E-07 |  |  |  |  |
| *PGA5* | -4.491666907 | 4.20E-23 | 6.25E-22 |  |  |  |  |
| *PIWIL4* | -2.956692794 | 1.38E-111 | 4.37E-109 |  |  |  |  |
| *PJCG1* | 2.667390482 | 8.55E-05 | 0.000245514 |  |  |  |  |
| *PLA2G16* | -2.476209273 | 3.19E-31 | 7.43E-30 |  |  |  |  |
| *PLEKHB1* | -4.239294063 | 3.52E-77 | 4.88E-75 |  |  |  |  |
| *PVRL1* | 2.125968209 | 1.02E-44 | 4.47E-43 |  |  |  |  |
| *RAB38* | 3.021567883 | 1.07E-54 | 6.66E-53 |  |  |  |  |
| *RN7SK.18* | -2.351435164 | 2.22E-06 | 7.94E-06 |  |  |  |  |
| *SCGB2A2* | 2.494045148 | 0.000399783 | 0.001027096 |  |  |  |  |
| *SESN3* | 2.625133584 | 9.83E-48 | 4.94E-46 |  |  |  |  |
| *SLC22A11* | -2.339125721 | 1.75E-10 | 1.01E-09 |  |  |  |  |
| *SLC22A20* | 2.465976948 | 2.16E-30 | 4.81E-29 |  |  |  |  |
| *SLC22A25* | 3.105336126 | 5.32E-16 | 4.93E-15 |  |  |  |  |
| *SLC43A1* | -2.822151492 | 7.09E-59 | 5.14E-57 |  |  |  |  |
| *SPATA19* | 2.736669292 | 3.72E-12 | 2.52E-11 |  |  |  |  |
| *SPTBN2.2* | 2.110854287 | 1.21E-11 | 7.76E-11 |  |  |  |  |
| *SYT13* | -4.338346096 | 4.98E-46 | 2.32E-44 |  |  |  |  |
| *SYTL2* | -2.458242034 | 5.74E-55 | 3.62E-53 |  |  |  |  |
| *TBX10* | -2.88586472 | 1.00E-21 | 1.37E-20 |  |  |  |  |
| *TMEM151A* | -3.281517438 | 1.27E-32 | 3.20E-31 |  |  |  |  |
| *TMEM45B* | -2.614550716 | 2.28E-29 | 4.85E-28 |  |  |  |  |
| *TMPRSS5* | -3.030773733 | 4.04E-47 | 1.98E-45 |  |  |  |  |
| *TNNT3* | 4.472197489 | 1.77E-46 | 8.46E-45 |  |  |  |  |
| *TP53AIP1* | 4.248488967 | 5.81E-61 | 4.62E-59 |  |  |  |  |
| *TPH1* | -2.052058094 | 3.73E-19 | 4.30E-18 |  |  |  |  |
| *TREH* | -2.023979958 | 3.57E-18 | 3.87E-17 |  |  |  |  |
| *TRIM29* | 2.01092143 | 1.50E-16 | 1.46E-15 |  |  |  |  |
| *TRIM48* | 2.470385617 | 0.003549192 | 0.007659465 |  |  |  |  |
| *TRIM49B* | 3.960770212 | 2.83E-08 | 1.29E-07 |  |  |  |  |
| *TRIM49C* | 2.900433908 | 5.47E-05 | 0.000161519 |  |  |  |  |
| *TRIM49* | 3.382757517 | 1.04E-05 | 3.41E-05 |  |  |  |  |
| *TRIM51P4.1* | 2.076650056 | 0.001369635 | 0.003206438 |  |  |  |  |
| *TRIM64B* | 3.539526404 | 3.50E-05 | 0.000106363 |  |  |  |  |
| *TRNA_Ser.1* | -2.051400639 | 4.02E-26 | 7.23E-25 |  |  |  |  |
| *TRPM5* | -4.012674502 | 2.94E-45 | 1.31E-43 |  |  |  |  |
| *U6.28* | 2.615996986 | 0.00053998 | 0.001356491 |  |  |  |  |
| *USH1C* | -6.33850179 | 6.03E-123 | 2.34E-120 |  |  |  |  |
| *VSIG2* | -4.792388313 | 2.84E-64 | 2.54E-62 |  |  |  |  |
| *WT1-AS* | 2.563880144 | 8.07E-10 | 4.34E-09 |  |  |  |  |
| *ADAMTS20* | 2.59513442 | 1.02E-13 | 7.93E-13 |  |  |  |  |
| *AK056228* | 3.227389051 | 1.41E-28 | 2.89E-27 |  |  |  |  |
| *AK094733* | 2.056579274 | 5.37E-07 | 2.09E-06 |  |  |  |  |
| *AK096314* | 2.057494625 | 3.16E-25 | 5.36E-24 |  |  |  |  |
| *AK125333* | 2.873906625 | 1.49E-09 | 7.83E-09 |  |  |  |  |
| *ANKRD33* | -2.0167538 | 1.99E-06 | 7.20E-06 |  |  |  |  |
| *ANP32D* | 4.451664714 | 1.05E-15 | 9.52E-15 |  |  |  |  |
| *APOBEC1* | -6.074296394 | 7.35E-66 | 6.84E-64 |  |  |  |  |
| *AQP2* | -6.636874563 | 2.06E-54 | 1.27E-52 |  |  |  |  |
| *AQP5* | -3.626605756 | 1.21E-22 | 1.75E-21 |  |  |  |  |
| *AQP6* | -2.508497608 | 5.92E-25 | 9.89E-24 |  |  |  |  |
| *ASCL4* | 4.392162694 | 6.99E-27 | 1.32E-25 |  |  |  |  |
| *AX746535* | 5.112365034 | 4.75E-42 | 1.86E-40 |  |  |  |  |
| *B4GALNT1* | 2.972361908 | 1.38E-42 | 5.55E-41 |  |  |  |  |
| *BC043551* | 3.641925794 | 5.18E-08 | 2.29E-07 |  |  |  |  |
| *BC045559* | 3.745152019 | 5.59E-26 | 9.97E-25 |  |  |  |  |
| *BC047615* | 2.25033759 | 0.000128176 | 0.000358015 |  |  |  |  |
| *BC053951* | 2.496751526 | 1.47E-07 | 6.13E-07 |  |  |  |  |
| *BCL2L14* | -3.530517951 | 6.82E-69 | 6.94E-67 |  |  |  |  |
| *BTBD11* | 2.02943434 | 4.32E-18 | 4.67E-17 |  |  |  |  |
| *C12orf12* | 2.291557508 | 0.000153436 | 0.000423085 |  |  |  |  |
| *C12orf28* | -2.282559343 | 6.47E-20 | 7.86E-19 |  |  |  |  |
| *C12orf36* | -2.208151159 | 2.33E-16 | 2.23E-15 |  |  |  |  |
| *C12orf37* | 2.98323811 | 3.47E-11 | 2.14E-10 |  |  |  |  |
| *C12orf54* | 3.623545894 | 1.49E-27 | 2.92E-26 |  |  |  |  |
| *CCDC38* | 2.572171171 | 1.84E-25 | 3.17E-24 |  |  |  |  |
| *CLEC12B* | 2.653301991 | 8.67E-21 | 1.12E-19 |  |  |  |  |
| *CLEC2A* | 2.436104198 | 4.62E-05 | 0.000137713 |  |  |  |  |
| *CLEC2B* | 2.243939406 | 2.21E-37 | 7.01E-36 |  |  |  |  |
| *CNTN1* | 3.594154625 | 1.60E-33 | 4.23E-32 |  |  |  |  |
| *CSRP2* | 2.057829392 | 9.98E-38 | 3.22E-36 |  |  |  |  |
| *DNAJC22* | -3.806054399 | 5.60E-84 | 9.76E-82 |  |  |  |  |
| *DQ590166* | 2.979937752 | 7.66E-13 | 5.50E-12 |  |  |  |  |
| *DQ591569* | 2.611593707 | 8.12E-06 | 2.70E-05 |  |  |  |  |
| *EPYC* | 3.161546914 | 2.08E-15 | 1.85E-14 |  |  |  |  |
| *F379* | 2.578983878 | 0.000220303 | 0.000590853 |  |  |  |  |
| *FAIM2* | -2.373617675 | 3.42E-20 | 4.24E-19 |  |  |  |  |
| *FAM101A* | -3.801899512 | 2.26E-81 | 3.64E-79 |  |  |  |  |
| *FAR2* | -2.159300893 | 3.10E-41 | 1.17E-39 |  |  |  |  |
| *FLJ31485* | 2.276162293 | 7.13E-25 | 1.19E-23 |  |  |  |  |
| *FLJ37505* | 3.258706511 | 1.00E-08 | 4.82E-08 |  |  |  |  |
| *FZD10* | 2.891820997 | 1.59E-35 | 4.65E-34 |  |  |  |  |
| *GALNT4* | -2.217112905 | 1.03E-84 | 1.85E-82 |  |  |  |  |
| *GALNT8* | -3.730560384 | 1.60E-32 | 4.01E-31 |  |  |  |  |
| *GPD1* | -5.273773799 | 7.31E-115 | 2.45E-112 |  |  |  |  |
| *GPRC5A* | -2.72910451 | 7.80E-39 | 2.65E-37 |  |  |  |  |
| *GRIN2B* | -2.54194117 | 5.71E-13 | 4.15E-12 |  |  |  |  |
| *GUCY2C* | -5.054847346 | 6.60E-110 | 2.04E-107 |  |  |  |  |
| *HCAR2* | 2.292802539 | 8.83E-20 | 1.07E-18 |  |  |  |  |
| *HNF1A-AS1* | -7.711865158 | 3.58E-223 | 7.48E-220 |  |  |  |  |
| *HNF1A* | -4.796640207 | 0 | 0 |  |  |  |  |
| *HRK* | 2.547010247 | 3.72E-18 | 4.03E-17 |  |  |  |  |
| *IAPP* | 2.067546313 | 2.54E-08 | 1.17E-07 |  |  |  |  |
| *IGFBP6* | 2.292290726 | 1.06E-24 | 1.75E-23 |  |  |  |  |
| *KCNA1* | 2.031551555 | 3.11E-07 | 1.25E-06 |  |  |  |  |
| *KCNC2* | -2.212198853 | 4.48E-06 | 1.54E-05 |  |  |  |  |
| *KLRF2* | 3.402898405 | 2.38E-15 | 2.10E-14 |  |  |  |  |
| *KRT18* | -2.100284705 | 4.75E-48 | 2.41E-46 |  |  |  |  |
| *KRT1* | 5.381505874 | 6.11E-39 | 2.09E-37 |  |  |  |  |
| *KRT3* | 2.41979261 | 5.66E-12 | 3.76E-11 |  |  |  |  |
| *KRT5* | 3.553386367 | 9.86E-21 | 1.27E-19 |  |  |  |  |
| *KRT6A* | 2.205064159 | 4.38E-08 | 1.95E-07 |  |  |  |  |
| *KRT6B* | 2.275858304 | 2.71E-09 | 1.39E-08 |  |  |  |  |
| *KRT71* | 2.377373516 | 8.93E-15 | 7.53E-14 |  |  |  |  |
| *KRT74* | 5.433770374 | 1.07E-92 | 2.29E-90 |  |  |  |  |
| *KRT75* | 4.258417662 | 6.62E-24 | 1.04E-22 |  |  |  |  |
| *KRT76* | 3.852850908 | 1.38E-20 | 1.75E-19 |  |  |  |  |
| *KRT77* | 6.446233218 | 2.05E-57 | 1.41E-55 |  |  |  |  |
| *KRT79* | 3.802105516 | 1.06E-29 | 2.30E-28 |  |  |  |  |
| *KRT82* | 5.575110491 | 4.15E-42 | 1.63E-40 |  |  |  |  |
| *KRT84* | 2.937216755 | 9.26E-19 | 1.04E-17 |  |  |  |  |
| *KRT85* | 2.798402979 | 8.38E-12 | 5.47E-11 |  |  |  |  |
| *KRT8* | -2.333320644 | 6.82E-36 | 2.03E-34 |  |  |  |  |
| *LGR5* | -2.046647003 | 2.65E-11 | 1.65E-10 |  |  |  |  |
| *LHX5* | 4.607591651 | 8.49E-36 | 2.52E-34 |  |  |  |  |
| *LINC00173* | 2.495044544 | 7.16E-29 | 1.49E-27 |  |  |  |  |
| *LINC00592* | 2.616295066 | 4.23E-32 | 1.04E-30 |  |  |  |  |
| *LOC100131138* | 3.022316002 | 5.33E-09 | 2.64E-08 |  |  |  |  |
| *LOC100190940* | -5.102927497 | 1.70E-38 | 5.67E-37 |  |  |  |  |
| *LOC100292680* | 5.27995281 | 8.94E-51 | 4.95E-49 |  |  |  |  |
| *LOC255411* | -2.98679529 | 9.86E-19 | 1.11E-17 |  |  |  |  |
| *LOC440117* | 2.053505503 | 0.00394007 | 0.008426037 |  |  |  |  |
| *LYZ* | -4.048278895 | 3.46E-61 | 2.76E-59 |  |  |  |  |
| *METTL7B* | -3.740640391 | 6.32E-73 | 7.56E-71 |  |  |  |  |
| *MIR1293* | -2.015571597 | 6.14E-16 | 5.66E-15 |  |  |  |  |
| *MIR4497* | 2.15477275 | 5.72E-22 | 7.94E-21 |  |  |  |  |
| *MIR614* | -3.185295814 | 1.46E-53 | 8.72E-52 |  |  |  |  |
| *MIR920* | 2.15045764 | 0.00058458 | 0.001457079 |  |  |  |  |
| *MUC19.1* | 2.380416183 | 0.009815659 | 0.019327397 |  |  |  |  |
| *MUCL1* | 4.31690887 | 1.73E-32 | 4.32E-31 |  |  |  |  |
| *MYBPC1* | -2.114602349 | 3.26E-06 | 1.14E-05 |  |  |  |  |
| *MYO1A* | -7.496234699 | 5.12E-247 | 1.74E-243 |  |  |  |  |
| *Mir_544.11* | 2.642526044 | 0.000741239 | 0.001821648 |  |  |  |  |
| *NANOGNB* | 2.151688205 | 6.81E-05 | 0.000198419 |  |  |  |  |
| *NAV3* | 2.097309215 | 6.87E-21 | 8.92E-20 |  |  |  |  |
| *NDUFA4L2* | 2.89908199 | 2.97E-34 | 8.13E-33 |  |  |  |  |
| *NELL2* | 4.070449063 | 2.33E-61 | 1.89E-59 |  |  |  |  |
| *NOS1* | 5.48273036 | 6.71E-65 | 6.08E-63 |  |  |  |  |
| *NR1H4* | -5.015344404 | 1.75E-37 | 5.60E-36 |  |  |  |  |
| *NTS* | 5.090007437 | 5.51E-32 | 1.34E-30 |  |  |  |  |
| *NXPH4* | 3.560671228 | 2.11E-65 | 1.95E-63 |  |  |  |  |
| *OR6C2* | 3.135327573 | 7.94E-09 | 3.86E-08 |  |  |  |  |
| *OR6C3* | 2.427047993 | 0.008670386 | 0.017260713 |  |  |  |  |
| *OR6C65* | 3.399904674 | 1.67E-05 | 5.33E-05 |  |  |  |  |
| *OR6C68* | 2.174755407 | 0.009418512 | 0.018608239 |  |  |  |  |
| *OR6C70* | 5.15917897 | 3.13E-19 | 3.63E-18 |  |  |  |  |
| *OR6C75* | 2.774924421 | 6.69E-05 | 0.000195215 |  |  |  |  |
| *OR8S1* | 2.44713184 | 5.35E-06 | 1.83E-05 |  |  |  |  |
| *OR9K2* | 4.379834782 | 1.65E-11 | 1.05E-10 |  |  |  |  |
| *PAH* | -2.587223233 | 9.66E-13 | 6.88E-12 |  |  |  |  |
| *PIWIL1* | -4.510104621 | 1.82E-31 | 4.33E-30 |  |  |  |  |
| *PKP2* | -2.100422981 | 1.05E-42 | 4.27E-41 |  |  |  |  |
| *PRMT8* | 3.628988437 | 1.66E-23 | 2.53E-22 |  |  |  |  |
| *PTHLH* | 4.370297097 | 8.99E-55 | 5.60E-53 |  |  |  |  |
| *PTPRQ* | 2.39619479 | 1.39E-14 | 1.16E-13 |  |  |  |  |
| *PTPRR* | -2.878756593 | 1.83E-34 | 5.10E-33 |  |  |  |  |
| *RDH16* | 2.170361413 | 1.54E-24 | 2.51E-23 |  |  |  |  |
| *RND1* | -2.565366473 | 1.00E-40 | 3.72E-39 |  |  |  |  |
| *RPSAP52* | 2.218522856 | 6.40E-17 | 6.37E-16 |  |  |  |  |
| *SDR9C7* | 2.652570823 | 8.09E-10 | 4.35E-09 |  |  |  |  |
| *SLC26A10* | 2.760714665 | 1.43E-38 | 4.78E-37 |  |  |  |  |
| *SLC39A5* | -5.746447289 | 2.91E-171 | 2.33E-168 |  |  |  |  |
| *SLC41A2* | -2.6662342 | 4.18E-72 | 4.90E-70 |  |  |  |  |
| *SLC6A15* | 3.719877695 | 2.86E-24 | 4.59E-23 |  |  |  |  |
| *SLCO1A2* | 2.332636762 | 6.29E-12 | 4.16E-11 |  |  |  |  |
| *SYT10* | 3.032864875 | 1.41E-11 | 9.01E-11 |  |  |  |  |
| *TAC3* | -3.508939487 | 1.17E-43 | 4.93E-42 |  |  |  |  |
| *TESC* | -5.801003531 | 5.60E-160 | 3.90E-157 |  |  |  |  |
| *TMEM132D* | -3.127515991 | 1.50E-17 | 1.56E-16 |  |  |  |  |
| *TRHDE* | -2.060457638 | 1.55E-10 | 8.98E-10 |  |  |  |  |
| *TSPAN19* | 2.321821792 | 8.48E-08 | 3.64E-07 |  |  |  |  |
| *TSPAN8* | -8.149590212 | 1.57E-225 | 3.89E-222 |  |  |  |  |
| *WDR66* | 3.239899245 | 3.00E-76 | 4.01E-74 |  |  |  |  |
| *WSCD2* | 2.864841321 | 7.43E-18 | 7.91E-17 |  |  |  |  |
| *Y_RNA.18* | 2.597849361 | 1.09E-05 | 3.55E-05 |  |  |  |  |
| *ZNF385A* | 2.400728507 | 5.07E-72 | 5.89E-70 |  |  |  |  |
| *contactin_1* | 3.308331061 | 7.22E-18 | 7.70E-17 |  |  |  |  |
| *ANKRD26P3* | 4.130645221 | 9.35E-38 | 3.02E-36 |  |  |  |  |
| *ATP12A* | 2.490138457 | 1.04E-09 | 5.55E-09 |  |  |  |  |
| *ATP4B* | -2.236302051 | 4.20E-13 | 3.08E-12 |  |  |  |  |
| *ATP7B* | -3.078726773 | 4.03E-84 | 7.06E-82 |  |  |  |  |
| *BC025370* | -2.939512881 | 7.45E-09 | 3.64E-08 |  |  |  |  |
| *BC038727* | -2.03423957 | 3.88E-05 | 0.000117128 |  |  |  |  |
| *BC043519* | -2.144264325 | 5.22E-13 | 3.81E-12 |  |  |  |  |
| *CCDC70.1* | -2.168525962 | 1.08E-08 | 5.14E-08 |  |  |  |  |
| *CCDC70.2* | -2.284436934 | 4.91E-09 | 2.44E-08 |  |  |  |  |
| *CCNA1* | 5.673466392 | 3.63E-58 | 2.58E-56 |  |  |  |  |
| *CDX2* | -4.816282772 | 4.18E-51 | 2.32E-49 |  |  |  |  |
| *DACH1* | -2.756098017 | 1.85E-29 | 3.95E-28 |  |  |  |  |
| *DCT* | 2.019664642 | 6.14E-10 | 3.35E-09 |  |  |  |  |
| *F10* | -2.372804194 | 9.16E-36 | 2.71E-34 |  |  |  |  |
| *F7* | -2.701819304 | 2.36E-24 | 3.80E-23 |  |  |  |  |
| *FAM155A* | -2.323276534 | 8.09E-31 | 1.85E-29 |  |  |  |  |
| *FAM216B* | -2.092519312 | 1.03E-12 | 7.31E-12 |  |  |  |  |
| *FGF14-IT1* | 2.466285224 | 7.41E-09 | 3.62E-08 |  |  |  |  |
| *GJA3* | 2.827873949 | 1.30E-32 | 3.28E-31 |  |  |  |  |
| *GJB2* | 2.332551506 | 1.81E-19 | 2.13E-18 |  |  |  |  |
| *GJB6* | 3.36037526 | 2.01E-22 | 2.88E-21 |  |  |  |  |
| *LOC100507240* | 2.563225809 | 2.62E-33 | 6.81E-32 |  |  |  |  |
| *MAB21L1* | 2.592932974 | 5.19E-19 | 5.94E-18 |  |  |  |  |
| *MIR2276* | -2.095613699 | 1.37E-06 | 5.07E-06 |  |  |  |  |
| *Metazoa_SRP.20* | 2.794971899 | 0.000715513 | 0.00176241 |  |  |  |  |
| *NEK5* | -2.569571262 | 4.32E-41 | 1.63E-39 |  |  |  |  |
| *OLFM4* | -8.303595359 | 2.82E-103 | 7.89E-101 |  |  |  |  |
| *OXGR1* | 2.008626543 | 2.96E-11 | 1.83E-10 |  |  |  |  |
| *PDX1* | -5.671992266 | 2.54E-88 | 5.04E-86 |  |  |  |  |
| *POU4F1* | 3.707999753 | 3.23E-23 | 4.86E-22 |  |  |  |  |
| *PRHOXNB* | -3.700594596 | 7.27E-11 | 4.34E-10 |  |  |  |  |
| *SERTM1* | 3.115778596 | 1.63E-08 | 7.67E-08 |  |  |  |  |
| *SHISA2* | 2.074746635 | 2.39E-13 | 1.79E-12 |  |  |  |  |
| *SLC10A2* | -3.290373923 | 3.21E-11 | 1.98E-10 |  |  |  |  |
| *SLITRK6* | 2.1048133 | 1.12E-12 | 7.90E-12 |  |  |  |  |
| *SOX1* | 7.27203652 | 3.12E-45 | 1.39E-43 |  |  |  |  |
| *SPACA7* | 2.085674043 | 0.015604144 | 0.029337821 |  |  |  |  |
| *SPG20OS* | 2.029760077 | 1.31E-21 | 1.78E-20 |  |  |  |  |
| *AHNAK2* | 2.148076797 | 1.80E-22 | 2.58E-21 |  |  |  |  |
| *AK093301* | -2.843037556 | 2.35E-06 | 8.38E-06 |  |  |  |  |
| *AK125397* | 2.530127501 | 2.93E-15 | 2.57E-14 |  |  |  |  |
| *AMN* | -2.600590595 | 8.34E-48 | 4.20E-46 |  |  |  |  |
| *ANG* | -2.413400102 | 1.92E-68 | 1.91E-66 |  |  |  |  |
| *AY748447* | -2.235967484 | 0.000519545 | 0.00130818 |  |  |  |  |
| *BC029479* | -2.962813499 | 4.63E-18 | 4.98E-17 |  |  |  |  |
| *BC034423* | 2.856038784 | 8.39E-06 | 2.78E-05 |  |  |  |  |
| *BC148262* | 3.341049971 | 3.03E-19 | 3.51E-18 |  |  |  |  |
| *BMP4* | -2.137711401 | 7.19E-26 | 1.28E-24 |  |  |  |  |
| *BX248253* | 3.521366827 | 2.51E-36 | 7.59E-35 |  |  |  |  |
| *C14orf105* | -5.3858826 | 2.10E-50 | 1.16E-48 |  |  |  |  |
| *C14orf162* | 3.306495736 | 3.07E-25 | 5.23E-24 |  |  |  |  |
| *C14orf177* | 2.811542582 | 0.000164631 | 0.000451253 |  |  |  |  |
| *C14orf180* | -3.440171045 | 1.32E-07 | 5.55E-07 |  |  |  |  |
| *C14orf184* | -3.467512193 | 4.67E-33 | 1.20E-31 |  |  |  |  |
| *C14orf23* | 3.325975952 | 6.75E-14 | 5.31E-13 |  |  |  |  |
| *C14orf39* | 2.814774561 | 9.68E-13 | 6.89E-12 |  |  |  |  |
| *CATSPERB* | -3.159149344 | 1.55E-46 | 7.40E-45 |  |  |  |  |
| *CHGA* | -4.09822629 | 1.23E-25 | 2.15E-24 |  |  |  |  |
| *CLMN* | -2.508155125 | 2.46E-90 | 5.03E-88 |  |  |  |  |
| *EFS* | 3.288832467 | 8.06E-69 | 8.11E-67 |  |  |  |  |
| *FAM181A-AS1* | 3.844247684 | 2.68E-16 | 2.55E-15 |  |  |  |  |
| *FAM181A* | 5.300117616 | 4.08E-35 | 1.17E-33 |  |  |  |  |
| *FLRT2* | 2.347216594 | 5.17E-25 | 8.68E-24 |  |  |  |  |
| *FOXG1* | 3.567488614 | 8.51E-26 | 1.50E-24 |  |  |  |  |
| *FRMD6-AS1* | 2.028033571 | 1.15E-38 | 3.87E-37 |  |  |  |  |
| *FRMD6* | 2.59175366 | 2.88E-58 | 2.07E-56 |  |  |  |  |
| *GPHB5* | 3.797386865 | 5.55E-09 | 2.75E-08 |  |  |  |  |
| *GPR33* | 2.202145722 | 2.72E-10 | 1.54E-09 |  |  |  |  |
| *HSPA2* | 2.362899324 | 7.97E-37 | 2.46E-35 |  |  |  |  |
| *IL25* | 4.219967859 | 1.58E-15 | 1.41E-14 |  |  |  |  |
| *Ig_alpha_1-[alpha]2m* | -2.347060496 | 1.28E-10 | 7.47E-10 |  |  |  |  |
| *LOC100129345* | 2.122740221 | 0.002308035 | 0.005171994 |  |  |  |  |
| *LOC100505967* | 2.634326617 | 2.17E-12 | 1.49E-11 |  |  |  |  |
| *LOC283553* | 4.117453176 | 2.01E-67 | 1.96E-65 |  |  |  |  |
| *LOC90925* | -2.076529525 | 2.97E-12 | 2.03E-11 |  |  |  |  |
| *LTB4R2* | 2.318733984 | 8.70E-46 | 3.96E-44 |  |  |  |  |
| *LTB4R* | 2.281269634 | 6.47E-41 | 2.42E-39 |  |  |  |  |
| *MIA2* | -6.087271936 | 1.88E-101 | 5.01E-99 |  |  |  |  |
| *MIR1260A* | 4.80584991 | 1.21E-22 | 1.75E-21 |  |  |  |  |
| *Mir_548.16* | 2.158859583 | 0.009842721 | 0.019373088 |  |  |  |  |
| *NGB* | 5.348414562 | 1.88E-43 | 7.79E-42 |  |  |  |  |
| *NKX2-8* | 3.005589848 | 2.45E-20 | 3.06E-19 |  |  |  |  |
| *OR11H2* | 2.604854189 | 0.00654265 | 0.013392935 |  |  |  |  |
| *OR11H4* | 3.627855385 | 1.79E-17 | 1.86E-16 |  |  |  |  |
| *OR11H6* | 2.429564122 | 6.05E-08 | 2.65E-07 |  |  |  |  |
| *OR4K2* | 2.671473545 | 0.000186925 | 0.000507548 |  |  |  |  |
| *OR4K5* | 2.489733822 | 0.004018865 | 0.008582382 |  |  |  |  |
| *OTX2* | 3.886032018 | 6.19E-14 | 4.88E-13 |  |  |  |  |
| *PPP1R36* | -2.595546476 | 1.44E-51 | 8.08E-50 |  |  |  |  |
| *PPP4R4* | 2.563474322 | 6.08E-22 | 8.43E-21 |  |  |  |  |
| *PRIMA1* | 2.969379152 | 1.71E-33 | 4.51E-32 |  |  |  |  |
| *PTGER2* | -2.479001183 | 1.58E-33 | 4.19E-32 |  |  |  |  |
| *RNASE10* | 4.531516136 | 1.03E-43 | 4.36E-42 |  |  |  |  |
| *RNASE11* | 2.318423124 | 0.005323934 | 0.011089556 |  |  |  |  |
| *RNASE4* | -2.406415639 | 2.52E-64 | 2.27E-62 |  |  |  |  |
| *SERPINA10* | -2.785064471 | 6.85E-12 | 4.51E-11 |  |  |  |  |
| *SERPINA12* | 2.33205322 | 2.99E-08 | 1.36E-07 |  |  |  |  |
| *SERPINA1* | -4.750424727 | 1.37E-98 | 3.30E-96 |  |  |  |  |
| *SERPINA4* | -6.628027735 | 1.54E-85 | 2.80E-83 |  |  |  |  |
| *SERPINA5* | -3.602904205 | 6.68E-42 | 2.60E-40 |  |  |  |  |
| *SERPINA6* | -2.292205342 | 7.83E-10 | 4.22E-09 |  |  |  |  |
| *SERPINA9* | 2.135638977 | 1.89E-06 | 6.84E-06 |  |  |  |  |
| *SIX6* | 3.426855704 | 4.85E-08 | 2.15E-07 |  |  |  |  |
| *SLC7A8* | 2.849768238 | 5.71E-57 | 3.88E-55 |  |  |  |  |
| *SSTR1* | -6.822417631 | 5.32E-116 | 1.81E-113 |  |  |  |  |
| *STON2* | 2.284171761 | 1.29E-53 | 7.71E-52 |  |  |  |  |
| *TRNA_Pseudo.36* | -2.362021762 | 1.12E-06 | 4.18E-06 |  |  |  |  |
| *TRNA_Thr.2* | -2.042870554 | 1.71E-06 | 6.23E-06 |  |  |  |  |
| *TRNA_Tyr.3* | -2.689814698 | 6.30E-09 | 3.10E-08 |  |  |  |  |
| *TRNA_Tyr.4* | -2.710093507 | 4.06E-12 | 2.74E-11 |  |  |  |  |
| *U6.40* | 3.73525465 | 6.80E-12 | 4.48E-11 |  |  |  |  |
| *U6.41* | 3.129694595 | 8.76E-23 | 1.28E-21 |  |  |  |  |
| *VRTN* | -3.501943131 | 1.42E-21 | 1.93E-20 |  |  |  |  |
| *tRNA_Pro.6* | -2.080188752 | 2.33E-07 | 9.48E-07 |  |  |  |  |
| *AK056686* | -2.854682578 | 6.24E-53 | 3.65E-51 |  |  |  |  |
| *AK094352* | 2.36650142 | 4.90E-11 | 2.98E-10 |  |  |  |  |
| *AK097829* | 2.835013839 | 4.29E-06 | 1.48E-05 |  |  |  |  |
| *AK302238* | 3.104764329 | 4.50E-11 | 2.74E-10 |  |  |  |  |
| *ALDH1A2* | -2.275338019 | 4.07E-14 | 3.26E-13 |  |  |  |  |
| *ANPEP* | -3.933677899 | 2.08E-46 | 9.86E-45 |  |  |  |  |
| *BC024169* | -2.404082228 | 0.000125326 | 0.000350738 |  |  |  |  |
| *BC036442* | -2.086037216 | 6.85E-08 | 2.98E-07 |  |  |  |  |
| *BC037952* | 2.732633172 | 5.26E-11 | 3.19E-10 |  |  |  |  |
| *BC043257* | 3.286350393 | 5.91E-20 | 7.21E-19 |  |  |  |  |
| *BC043570* | -3.940921629 | 1.41E-80 | 2.20E-78 |  |  |  |  |
| *BNC1* | 4.397918666 | 3.39E-37 | 1.07E-35 |  |  |  |  |
| *C15orf59* | 2.027612654 | 4.48E-16 | 4.18E-15 |  |  |  |  |
| *C2CD4A* | -2.626274777 | 8.45E-25 | 1.40E-23 |  |  |  |  |
| *C2CD4B* | -3.347596302 | 1.10E-47 | 5.50E-46 |  |  |  |  |
| *CA12* | 2.503408198 | 2.55E-34 | 7.00E-33 |  |  |  |  |
| *CALML4* | -2.057052155 | 3.71E-95 | 8.33E-93 |  |  |  |  |
| *CCDC33* | -2.349648778 | 2.87E-11 | 1.78E-10 |  |  |  |  |
| *CERS3* | 2.56361048 | 1.82E-12 | 1.26E-11 |  |  |  |  |
| *CHRNB4* | 2.749793446 | 3.90E-39 | 1.35E-37 |  |  |  |  |
| *DQ571347* | -2.315016846 | 0.001475743 | 0.003434131 |  |  |  |  |
| *DQ574028* | -2.636295412 | 2.11E-05 | 6.62E-05 |  |  |  |  |
| *DQ575076* | -2.344834469 | 6.08E-05 | 0.00017836 |  |  |  |  |
| *DQ576468* | -2.01620681 | 0.009143692 | 0.018117982 |  |  |  |  |
| *DQ578058* | -2.523518379 | 2.09E-08 | 9.71E-08 |  |  |  |  |
| *DQ579313* | -2.212878538 | 0.001799461 | 0.004116213 |  |  |  |  |
| *DQ580467* | -3.120455638 | 1.54E-08 | 7.24E-08 |  |  |  |  |
| *DQ581622* | -2.168507921 | 0.004688928 | 0.009878969 |  |  |  |  |
| *DQ583728* | -2.842744316 | 1.70E-06 | 6.20E-06 |  |  |  |  |
| *DQ584918* | -2.768795949 | 4.32E-09 | 2.16E-08 |  |  |  |  |
| *DQ589659* | -2.306082773 | 0.000341769 | 0.000887365 |  |  |  |  |
| *DQ593762* | -2.878717478 | 2.42E-06 | 8.60E-06 |  |  |  |  |
| *DQ594004* | -2.164247349 | 0.000468302 | 0.001187853 |  |  |  |  |
| *DQ597717* | -2.543642201 | 7.46E-07 | 2.85E-06 |  |  |  |  |
| *DQ598082* | -2.444236008 | 0.000606518 | 0.001508714 |  |  |  |  |
| *DQ598122* | -2.125471422 | 0.001385036 | 0.003238308 |  |  |  |  |
| *DQ599537* | -2.132354603 | 0.004016984 | 0.00857904 |  |  |  |  |
| *DQ599802* | -2.333952277 | 0.001399752 | 0.003271025 |  |  |  |  |
| *DQ600930* | -2.961199267 | 7.78E-07 | 2.96E-06 |  |  |  |  |
| *DUOX2* | -2.666675544 | 5.15E-24 | 8.10E-23 |  |  |  |  |
| *DUOXA2* | -2.226979714 | 9.41E-20 | 1.13E-18 |  |  |  |  |
| *FAM169B* | -3.714988364 | 7.60E-22 | 1.05E-20 |  |  |  |  |
| *FAM81A* | -2.020423998 | 4.54E-31 | 1.06E-29 |  |  |  |  |
| *FRMD5* | -2.730926998 | 5.78E-55 | 3.63E-53 |  |  |  |  |
| *GABRA5* | 3.976008917 | 1.63E-20 | 2.06E-19 |  |  |  |  |
| *GABRG3* | 3.563508055 | 8.06E-12 | 5.27E-11 |  |  |  |  |
| *GATM* | -2.420371127 | 2.92E-33 | 7.60E-32 |  |  |  |  |
| *GCNT3* | -4.788059077 | 7.74E-76 | 1.01E-73 |  |  |  |  |
| *GOLGA6L2* | 2.827345509 | 9.87E-17 | 9.68E-16 |  |  |  |  |
| *GOLGA6L6* | 2.724690326 | 8.26E-07 | 3.13E-06 |  |  |  |  |
| *HCN4* | -2.793446639 | 4.73E-15 | 4.08E-14 |  |  |  |  |
| *HDGFRP3* | 2.119204746 | 9.50E-45 | 4.16E-43 |  |  |  |  |
| *L12234* | -2.017509741 | 1.71E-14 | 1.41E-13 |  |  |  |  |
| *LIPC* | -2.929586143 | 1.61E-25 | 2.79E-24 |  |  |  |  |
| *LOC100144604* | -2.421421497 | 2.46E-13 | 1.84E-12 |  |  |  |  |
| *LOC145837* | -2.790780792 | 7.75E-24 | 1.21E-22 |  |  |  |  |
| *LOC254559* | 2.603597564 | 8.70E-26 | 1.54E-24 |  |  |  |  |
| *LOC283685* | 2.63564565 | 1.22E-12 | 8.61E-12 |  |  |  |  |
| *LOC283761* | 2.42702146 | 1.05E-16 | 1.03E-15 |  |  |  |  |
| *LOC390660.1* | 2.392788496 | 2.14E-05 | 6.71E-05 |  |  |  |  |
| *LOC648809* | 2.138387924 | 8.11E-12 | 5.30E-11 |  |  |  |  |
| *LTK* | -2.294519514 | 7.13E-21 | 9.24E-20 |  |  |  |  |
| *MYO5C* | -2.144109428 | 1.20E-39 | 4.27E-38 |  |  |  |  |
| *NBEAP1* | 4.356728869 | 4.21E-40 | 1.52E-38 |  |  |  |  |
| *NF1P2.2* | 3.096577948 | 9.61E-06 | 3.16E-05 |  |  |  |  |
| *OR4M2* | 2.771730456 | 0.000901813 | 0.00218546 |  |  |  |  |
| *OR4N4* | 2.182685149 | 0.00085714 | 0.002084421 |  |  |  |  |
| *OSTBETA* | -2.425122198 | 5.33E-26 | 9.53E-25 |  |  |  |  |
| *PHGR1* | -9.706889128 | 4.67E-173 | 4.22E-170 |  |  |  |  |
| *PLA2G4E* | 2.937679247 | 2.81E-19 | 3.27E-18 |  |  |  |  |
| *PPP1R14D* | -5.883167891 | 2.52E-137 | 1.29E-134 |  |  |  |  |
| *REREP3* | 2.402144299 | 7.80E-11 | 4.64E-10 |  |  |  |  |
| *RGMA* | 2.457066098 | 7.65E-27 | 1.44E-25 |  |  |  |  |
| *RHOV* | 2.05585477 | 2.51E-22 | 3.57E-21 |  |  |  |  |
| *SCG3* | -2.057776159 | 2.26E-11 | 1.42E-10 |  |  |  |  |
| *SH3GL3* | 3.400702029 | 5.96E-25 | 9.96E-24 |  |  |  |  |
| *SLC28A2* | -6.811046062 | 1.78E-73 | 2.17E-71 |  |  |  |  |
| *SMAD6* | -2.091952127 | 7.48E-69 | 7.56E-67 |  |  |  |  |
| *TMEM202* | 3.20536181 | 6.85E-08 | 2.98E-07 |  |  |  |  |
| *TRPM1.2* | 2.77682064 | 1.99E-21 | 2.67E-20 |  |  |  |  |
| *ABCC6P1* | -5.062746428 | 1.07E-78 | 1.56E-76 |  |  |  |  |
| *ABCC6P2* | -2.660291662 | 2.26E-26 | 4.13E-25 |  |  |  |  |
| *ABCC6* | -3.278577006 | 3.34E-76 | 4.45E-74 |  |  |  |  |
| *ACSM3* | -2.328083082 | 8.42E-45 | 3.70E-43 |  |  |  |  |
| *ADAD2* | 3.207717949 | 1.59E-36 | 4.88E-35 |  |  |  |  |
| *AK055272* | -2.504439266 | 6.74E-22 | 9.32E-21 |  |  |  |  |
| *AK123582* | 3.767767264 | 6.15E-38 | 2.01E-36 |  |  |  |  |
| *AK127191* | 2.322024053 | 4.69E-08 | 2.09E-07 |  |  |  |  |
| *ANKS4B* | -7.46522157 | 6.66E-187 | 7.24E-184 |  |  |  |  |
| *ARHGDIG* | -2.275714142 | 7.72E-14 | 6.05E-13 |  |  |  |  |
| *ASPHD1* | -2.787004876 | 6.07E-53 | 3.56E-51 |  |  |  |  |
| *BC068290* | 2.139980524 | 5.60E-05 | 0.000165196 |  |  |  |  |
| *C16orf74* | 3.155007666 | 2.14E-66 | 2.01E-64 |  |  |  |  |
| *CALB2* | 2.067658663 | 1.04E-10 | 6.12E-10 |  |  |  |  |
| *CAPNS2* | 2.996191806 | 1.39E-15 | 1.25E-14 |  |  |  |  |
| *CDH3* | 2.207902699 | 2.49E-36 | 7.56E-35 |  |  |  |  |
| *CDH8* | 3.194588279 | 1.63E-36 | 4.98E-35 |  |  |  |  |
| *CES1P1* | 3.66622197 | 3.46E-13 | 2.56E-12 |  |  |  |  |
| *CES1P2* | 4.637437613 | 1.01E-26 | 1.89E-25 |  |  |  |  |
| *CES1* | 4.43178705 | 3.26E-31 | 7.59E-30 |  |  |  |  |
| *CHST4* | -3.569874723 | 6.43E-27 | 1.22E-25 |  |  |  |  |
| *CHST5* | -4.476073163 | 4.14E-71 | 4.52E-69 |  |  |  |  |
| *CLDN6* | -4.783304566 | 6.56E-33 | 1.68E-31 |  |  |  |  |
| *CLDN9* | -3.057778706 | 3.02E-27 | 5.81E-26 |  |  |  |  |
| *CNGB1* | 5.51038504 | 1.13E-69 | 1.18E-67 |  |  |  |  |
| *CRYM* | -2.002698259 | 6.16E-19 | 7.00E-18 |  |  |  |  |
| *DPEP1* | -6.380156042 | 4.75E-85 | 8.55E-83 |  |  |  |  |
| *DQ573285* | 4.00129236 | 7.83E-09 | 3.81E-08 |  |  |  |  |
| *DYNLRB2* | 2.205413536 | 5.35E-17 | 5.38E-16 |  |  |  |  |
| *ERN2* | -5.168686592 | 2.92E-99 | 7.07E-97 |  |  |  |  |
| *FA2H* | -3.494899947 | 1.15E-72 | 1.36E-70 |  |  |  |  |
| *FOXC2* | 2.640365666 | 3.00E-26 | 5.43E-25 |  |  |  |  |
| *GP2* | -8.02457106 | 1.85E-78 | 2.65E-76 |  |  |  |  |
| *GPRC5B* | -2.136808485 | 2.69E-27 | 5.20E-26 |  |  |  |  |
| *HAS3* | 2.519607264 | 5.24E-46 | 2.43E-44 |  |  |  |  |
| *HS3ST6* | 3.461691981 | 9.30E-20 | 1.12E-18 |  |  |  |  |
| *HSD17B2* | -3.531447069 | 6.97E-39 | 2.38E-37 |  |  |  |  |
| *IGFALS* | -3.884485589 | 3.51E-46 | 1.65E-44 |  |  |  |  |
| *IGH.1* | 2.053377964 | 0.00076084 | 0.001866441 |  |  |  |  |
| *IL17C* | -2.830998103 | 8.47E-21 | 1.09E-19 |  |  |  |  |
| *IRF8* | -2.536475681 | 5.52E-45 | 2.44E-43 |  |  |  |  |
| *IRX3* | 2.708248214 | 5.15E-36 | 1.54E-34 |  |  |  |  |
| *IRX6* | 5.179122882 | 2.15E-50 | 1.18E-48 |  |  |  |  |
| *KCNG4* | 2.11463823 | 0.000570182 | 0.001424851 |  |  |  |  |
| *KREMEN2* | 3.339675492 | 1.58E-45 | 7.12E-44 |  |  |  |  |
| *LOC146336* | -2.711820226 | 1.51E-10 | 8.77E-10 |  |  |  |  |
| *LOC283914* | 3.722147111 | 6.61E-08 | 2.88E-07 |  |  |  |  |
| *LOC440335* | -2.468355443 | 1.55E-23 | 2.37E-22 |  |  |  |  |
| *LOC643714* | -3.402329716 | 8.83E-19 | 9.95E-18 |  |  |  |  |
| *LRRC36* | -2.573741435 | 1.08E-33 | 2.89E-32 |  |  |  |  |
| *MAF* | 2.09645321 | 6.02E-41 | 2.26E-39 |  |  |  |  |
| *MIR3182* | 2.810568021 | 3.39E-06 | 1.18E-05 |  |  |  |  |
| *MIR548AE2* | 2.639056825 | 3.27E-06 | 1.14E-05 |  |  |  |  |
| *MIR662* | -2.877496688 | 9.79E-11 | 5.79E-10 |  |  |  |  |
| *MSLNL* | -2.449030167 | 3.47E-13 | 2.56E-12 |  |  |  |  |
| *MSLN* | -3.162707864 | 3.43E-21 | 4.52E-20 |  |  |  |  |
| *MT1G* | -2.427844256 | 8.73E-22 | 1.20E-20 |  |  |  |  |
| *MT1H* | -2.579878968 | 7.25E-22 | 1.00E-20 |  |  |  |  |
| *Metazoa_SRP.41* | 2.695427178 | 0.000187134 | 0.000508053 |  |  |  |  |
| *NDRG4* | 3.291784718 | 1.36E-54 | 8.41E-53 |  |  |  |  |
| *NECAB2* | 3.622805883 | 3.39E-37 | 1.07E-35 |  |  |  |  |
| *NOD2* | 2.131658575 | 6.03E-28 | 1.20E-26 |  |  |  |  |
| *OR1F1* | 2.40848281 | 5.80E-11 | 3.50E-10 |  |  |  |  |
| *OR1F2P* | 2.505497308 | 2.11E-10 | 1.20E-09 |  |  |  |  |
| *PDIA2* | -3.357326723 | 8.50E-28 | 1.68E-26 |  |  |  |  |
| *PLA2G10* | -3.794673132 | 4.40E-68 | 4.32E-66 |  |  |  |  |
| *PRSS30P* | -3.746817783 | 1.59E-43 | 6.63E-42 |  |  |  |  |
| *PRSS33* | -5.862028785 | 1.31E-68 | 1.31E-66 |  |  |  |  |
| *PYDC1* | 2.507824233 | 2.28E-09 | 1.17E-08 |  |  |  |  |
| *QPRT* | -2.186089743 | 6.16E-19 | 7.01E-18 |  |  |  |  |
| *RPL3L* | 4.619490882 | 9.88E-70 | 1.04E-67 |  |  |  |  |
| *SHISA9* | 2.143769025 | 1.15E-08 | 5.50E-08 |  |  |  |  |
| *SLC6A2* | 5.165372258 | 2.42E-59 | 1.79E-57 |  |  |  |  |
| *SMPD3* | -3.430352786 | 1.93E-71 | 2.16E-69 |  |  |  |  |
| *SULT1A2* | -2.020911188 | 1.48E-20 | 1.88E-19 |  |  |  |  |
| *TMC5* | -5.756090058 | 4.86E-137 | 2.45E-134 |  |  |  |  |
| *TMEM114* | 2.21166091 | 3.07E-05 | 9.41E-05 |  |  |  |  |
| *TOX3* | -4.790216121 | 5.14E-52 | 2.94E-50 |  |  |  |  |
| *TRNA_Lys.14* | 2.422741817 | 0.000676178 | 0.001671586 |  |  |  |  |
| *TRNA_Pseudo.41* | 2.678380717 | 0.000185219 | 0.000503166 |  |  |  |  |
| *VAT1L* | -2.074720548 | 4.91E-32 | 1.20E-30 |  |  |  |  |
| *ZG16B* | -3.76226784 | 7.73E-33 | 1.97E-31 |  |  |  |  |
| *ZG16* | -3.820936032 | 6.90E-20 | 8.37E-19 |  |  |  |  |
| *tRNA_Pro.15* | 2.709122706 | 1.78E-06 | 6.48E-06 |  |  |  |  |
| *AATK* | -2.657456959 | 3.72E-52 | 2.15E-50 |  |  |  |  |
| *AB062083.2* | 2.486813059 | 0.002081926 | 0.004704508 |  |  |  |  |
| *ABCC3* | -2.146478152 | 7.94E-29 | 1.65E-27 |  |  |  |  |
| *AF289551* | -2.564636242 | 5.71E-84 | 9.88E-82 |  |  |  |  |
| *AIPL1* | 2.324485673 | 6.33E-11 | 3.80E-10 |  |  |  |  |
| *AK055254* | 2.35682959 | 1.91E-28 | 3.90E-27 |  |  |  |  |
| *AK097500* | 2.606994258 | 1.43E-23 | 2.19E-22 |  |  |  |  |
| *AK127460* | 4.351581195 | 2.40E-86 | 4.46E-84 |  |  |  |  |
| *AK127974* | 2.600156205 | 9.46E-06 | 3.11E-05 |  |  |  |  |
| *AK296148* | 3.722423376 | 4.79E-42 | 1.88E-40 |  |  |  |  |
| *AK301679* | 2.574590672 | 2.30E-13 | 1.73E-12 |  |  |  |  |
| *ALDH3A1* | 3.725659347 | 1.40E-31 | 3.35E-30 |  |  |  |  |
| *ALOX12B* | 2.456923751 | 1.12E-16 | 1.09E-15 |  |  |  |  |
| *ALOX12P2* | 2.374106301 | 1.80E-27 | 3.51E-26 |  |  |  |  |
| *ALOX15B* | 2.195709793 | 1.32E-13 | 1.01E-12 |  |  |  |  |
| *ALOXE3* | 2.631184594 | 7.64E-24 | 1.19E-22 |  |  |  |  |
| *APOH* | -4.685843057 | 2.60E-30 | 5.77E-29 |  |  |  |  |
| *ARHGAP44* | -2.051386911 | 3.60E-33 | 9.30E-32 |  |  |  |  |
| *ARL4D* | 2.491402521 | 2.80E-30 | 6.21E-29 |  |  |  |  |
| *ASGR1* | -2.373551354 | 1.29E-25 | 2.26E-24 |  |  |  |  |
| *ATP2A3* | -2.57337596 | 1.20E-48 | 6.24E-47 |  |  |  |  |
| *AX747630* | -3.244181496 | 1.84E-15 | 1.64E-14 |  |  |  |  |
| *AX748345* | -2.596624119 | 3.75E-41 | 1.42E-39 |  |  |  |  |
| *AXIN2* | -2.734705313 | 8.79E-35 | 2.49E-33 |  |  |  |  |
| *BC037347* | 3.403885116 | 1.08E-33 | 2.89E-32 |  |  |  |  |
| *BC043554* | -3.813622449 | 6.36E-19 | 7.23E-18 |  |  |  |  |
| *BC044939* | -4.690801837 | 1.67E-93 | 3.66E-91 |  |  |  |  |
| *BC046191* | 2.160752463 | 2.12E-17 | 2.19E-16 |  |  |  |  |
| *BC084573* | 2.413957701 | 9.36E-09 | 4.51E-08 |  |  |  |  |
| *C17orf110* | -5.521911049 | 9.29E-189 | 1.10E-185 |  |  |  |  |
| *C17orf28* | -3.612493864 | 5.59E-100 | 1.41E-97 |  |  |  |  |
| *C1QL1* | 2.217822448 | 2.11E-22 | 3.02E-21 |  |  |  |  |
| *CA4* | -6.32772963 | 2.81E-61 | 2.26E-59 |  |  |  |  |
| *CDK5R1* | 2.063052306 | 2.96E-39 | 1.03E-37 |  |  |  |  |
| *COPZ2* | 2.420794819 | 8.74E-58 | 6.10E-56 |  |  |  |  |
| *CORO6* | 3.200815869 | 3.26E-58 | 2.33E-56 |  |  |  |  |
| *DLX3* | 2.056917466 | 1.19E-11 | 7.66E-11 |  |  |  |  |
| *DNAH17* | 3.907530793 | 7.20E-103 | 1.98E-100 |  |  |  |  |
| *DQ570973* | -2.326164711 | 1.92E-22 | 2.76E-21 |  |  |  |  |
| *EFNB3* | 2.128073081 | 5.66E-25 | 9.48E-24 |  |  |  |  |
| *ENPP7* | -2.758045657 | 8.24E-14 | 6.45E-13 |  |  |  |  |
| *ERBB2* | -2.532984287 | 1.05E-29 | 2.28E-28 |  |  |  |  |
| *EVPLL* | 2.028246181 | 5.67E-13 | 4.12E-12 |  |  |  |  |
| *FAM211A* | -2.814876242 | 4.48E-98 | 1.06E-95 |  |  |  |  |
| *FGF11* | 2.449355433 | 2.46E-34 | 6.76E-33 |  |  |  |  |
| *FOXJ1* | -2.597555512 | 4.62E-12 | 3.09E-11 |  |  |  |  |
| *FOXN1* | 3.500143192 | 2.36E-31 | 5.57E-30 |  |  |  |  |
| *GDPD1* | -2.083346673 | 1.28E-39 | 4.53E-38 |  |  |  |  |
| *GSDMB* | -3.241444482 | 2.67E-60 | 2.07E-58 |  |  |  |  |
| *HAP1* | 3.201729424 | 9.15E-43 | 3.73E-41 |  |  |  |  |
| *HCRT* | -2.05221745 | 9.02E-08 | 3.86E-07 |  |  |  |  |
| *HES7* | 2.131497307 | 3.73E-21 | 4.91E-20 |  |  |  |  |
| *HNF1B* | -7.961234686 | 1.06E-202 | 1.60E-199 |  |  |  |  |
| *HOXB-AS3* | -3.097676088 | 4.70E-76 | 6.20E-74 |  |  |  |  |
| *HOXB-AS5* | -2.025471608 | 1.24E-08 | 5.87E-08 |  |  |  |  |
| *HOXB13* | -2.331213011 | 1.50E-13 | 1.14E-12 |  |  |  |  |
| *HOXB5* | -2.770522565 | 6.95E-63 | 5.94E-61 |  |  |  |  |
| *HOXB6* | -3.333341793 | 4.40E-76 | 5.83E-74 |  |  |  |  |
| *HOXB8* | -3.210235652 | 8.63E-31 | 1.97E-29 |  |  |  |  |
| *HOXB9* | -2.520042178 | 6.88E-22 | 9.51E-21 |  |  |  |  |
| *HS3ST3A1* | 2.63796751 | 6.41E-37 | 1.99E-35 |  |  |  |  |
| *KCNH6* | -3.652719876 | 1.66E-27 | 3.25E-26 |  |  |  |  |
| *KCNJ16* | -2.247496117 | 2.31E-08 | 1.06E-07 |  |  |  |  |
| *KIF2B.2* | 2.02213619 | 0.016451646 | 0.030739763 |  |  |  |  |
| *KRT14* | 3.684362987 | 2.83E-19 | 3.29E-18 |  |  |  |  |
| *KRT15* | 3.380692135 | 1.62E-30 | 3.63E-29 |  |  |  |  |
| *KRT16P3* | 2.205684597 | 1.19E-08 | 5.65E-08 |  |  |  |  |
| *KRT16* | 3.052904162 | 9.17E-17 | 9.01E-16 |  |  |  |  |
| *KRT17* | 3.708351993 | 5.31E-43 | 2.17E-41 |  |  |  |  |
| *KRT20* | -9.00066883 | 1.22E-116 | 4.21E-114 |  |  |  |  |
| *KRT27* | 2.061800221 | 2.03E-06 | 7.32E-06 |  |  |  |  |
| *KRT31* | 5.083792401 | 6.96E-42 | 2.71E-40 |  |  |  |  |
| *KRT32* | 2.912098908 | 3.22E-16 | 3.03E-15 |  |  |  |  |
| *KRT33B* | 2.997126688 | 1.15E-18 | 1.29E-17 |  |  |  |  |
| *KRT34* | 3.844158106 | 9.14E-38 | 2.96E-36 |  |  |  |  |
| *KRT35* | 3.075867686 | 1.74E-14 | 1.44E-13 |  |  |  |  |
| *KRT37* | 3.331783456 | 4.21E-19 | 4.85E-18 |  |  |  |  |
| *KRT38* | 3.17737256 | 2.33E-14 | 1.91E-13 |  |  |  |  |
| *KRT40* | -2.44824239 | 2.01E-10 | 1.15E-09 |  |  |  |  |
| *KRT42P* | 4.301257894 | 2.29E-48 | 1.18E-46 |  |  |  |  |
| *KRT9* | 3.246570059 | 5.78E-31 | 1.34E-29 |  |  |  |  |
| *KRTAP3-3* | -2.671597436 | 7.05E-06 | 2.36E-05 |  |  |  |  |
| *LHX1* | 3.05268029 | 1.36E-16 | 1.32E-15 |  |  |  |  |
| *LINC00483* | -6.464130932 | 4.39E-120 | 1.63E-117 |  |  |  |  |
| *LLGL2* | -2.256299647 | 4.27E-79 | 6.34E-77 |  |  |  |  |
| *LOC100289255* | -4.803669082 | 2.30E-56 | 1.54E-54 |  |  |  |  |
| *LOC100505782* | 2.409889157 | 9.48E-11 | 5.61E-10 |  |  |  |  |
| *LOC339240* | 4.535154234 | 7.61E-45 | 3.35E-43 |  |  |  |  |
| *MARCH10* | 2.262196866 | 5.12E-19 | 5.87E-18 |  |  |  |  |
| *MFSD6L* | -2.331594884 | 1.12E-19 | 1.34E-18 |  |  |  |  |
| *MIEN1* | -2.030021861 | 3.07E-22 | 4.35E-21 |  |  |  |  |
| *MIR2117* | 4.82700043 | 6.11E-35 | 1.74E-33 |  |  |  |  |
| *MIR3185* | -2.143587642 | 4.23E-10 | 2.35E-09 |  |  |  |  |
| *MIR4728* | -2.707647479 | 2.25E-32 | 5.60E-31 |  |  |  |  |
| *MIR497* | 2.409227722 | 8.52E-05 | 0.000244694 |  |  |  |  |
| *MIR657* | -2.736865834 | 6.57E-16 | 6.04E-15 |  |  |  |  |
| *MYADML2* | -2.679457006 | 2.58E-30 | 5.72E-29 |  |  |  |  |
| *MYH4* | -2.961887614 | 9.76E-13 | 6.94E-12 |  |  |  |  |
| *MYO15B* | -2.90117954 | 7.62E-79 | 1.12E-76 |  |  |  |  |
| *Metazoa_SRP.46* | 2.427576568 | 1.30E-07 | 5.47E-07 |  |  |  |  |
| *NGFR* | 3.917163404 | 7.18E-46 | 3.29E-44 |  |  |  |  |
| *NOS2* | -3.695302925 | 1.31E-41 | 5.03E-40 |  |  |  |  |
| *NPTX1* | 2.460692675 | 1.16E-17 | 1.22E-16 |  |  |  |  |
| *OR1G1* | 2.787439753 | 2.26E-09 | 1.16E-08 |  |  |  |  |
| *OR3A3* | 2.125062508 | 0.002368148 | 0.005294907 |  |  |  |  |
| *OSBPL7* | -2.160027048 | 4.02E-59 | 2.97E-57 |  |  |  |  |
| *PNMT* | -2.78766609 | 1.62E-20 | 2.05E-19 |  |  |  |  |
| *PPP1R1B* | -6.782682036 | 4.53E-78 | 6.41E-76 |  |  |  |  |
| *PRR15L* | -4.761748505 | 2.67E-103 | 7.55E-101 |  |  |  |  |
| *PYY2* | 3.334901327 | 1.65E-33 | 4.36E-32 |  |  |  |  |
| *RNF157* | -2.419790528 | 9.00E-34 | 2.41E-32 |  |  |  |  |
| *RTN4RL1* | 2.57439552 | 2.30E-21 | 3.07E-20 |  |  |  |  |
| *SDK2* | 2.465723597 | 3.92E-26 | 7.06E-25 |  |  |  |  |
| *SLC13A2* | -5.220494313 | 1.04E-46 | 5.02E-45 |  |  |  |  |
| *SLC35G3* | 3.602178677 | 1.69E-15 | 1.51E-14 |  |  |  |  |
| *SLC47A1* | 2.773790835 | 1.81E-40 | 6.64E-39 |  |  |  |  |
| *SLC47A2* | 3.742223054 | 4.81E-56 | 3.17E-54 |  |  |  |  |
| *SLC52A1* | 2.531692099 | 4.54E-35 | 1.30E-33 |  |  |  |  |
| *SNORA59B.2* | 2.44612499 | 0.000284003 | 0.000748108 |  |  |  |  |
| *SOST* | 7.178070011 | 1.44E-78 | 2.08E-76 |  |  |  |  |
| *SOX15* | 3.838969708 | 1.18E-61 | 9.61E-60 |  |  |  |  |
| *SPEM1* | 2.411425715 | 2.25E-34 | 6.20E-33 |  |  |  |  |
| *SPNS3* | -2.551288885 | 5.01E-39 | 1.72E-37 |  |  |  |  |
| *ST6GALNAC1* | -2.950552654 | 3.96E-30 | 8.72E-29 |  |  |  |  |
| *ST6GALNAC2* | 2.671056069 | 7.27E-63 | 6.19E-61 |  |  |  |  |
| *TM4SF5* | -9.092408174 | 4.08E-177 | 3.96E-174 |  |  |  |  |
| *TRIM16L* | 2.397352439 | 1.03E-32 | 2.60E-31 |  |  |  |  |
| *TSPAN10* | 2.187457437 | 2.37E-35 | 6.87E-34 |  |  |  |  |
| *TTLL6* | -5.190371891 | 9.13E-63 | 7.75E-61 |  |  |  |  |
| *USH1G* | 2.313360126 | 3.52E-11 | 2.17E-10 |  |  |  |  |
| *VTN* | -3.159113088 | 3.52E-46 | 1.65E-44 |  |  |  |  |
| *WNK4* | -2.403951832 | 3.43E-21 | 4.52E-20 |  |  |  |  |
| *ZNF750* | 2.29636929 | 1.93E-13 | 1.46E-12 |  |  |  |  |
| *ACAA2* | -2.064934053 | 4.18E-40 | 1.51E-38 |  |  |  |  |
| *AK093940* | 2.3658966 | 1.43E-16 | 1.39E-15 |  |  |  |  |
| *BC037384* | 2.220013131 | 0.002947995 | 0.006476767 |  |  |  |  |
| *BC042382* | 3.677402285 | 1.62E-34 | 4.51E-33 |  |  |  |  |
| *BC047599* | -2.288606995 | 2.69E-10 | 1.52E-09 |  |  |  |  |
| *BOD1L2* | -2.55475233 | 0.001016094 | 0.002441035 |  |  |  |  |
| *C18orf23* | 3.798730241 | 2.63E-24 | 4.22E-23 |  |  |  |  |
| *C18orf62* | 2.309905902 | 7.80E-05 | 0.000225281 |  |  |  |  |
| *CABLES1* | -2.042573215 | 1.15E-35 | 3.40E-34 |  |  |  |  |
| *CABYR* | 2.32422093 | 1.76E-22 | 2.53E-21 |  |  |  |  |
| *CCDC68* | -2.587105001 | 3.52E-38 | 1.16E-36 |  |  |  |  |
| *CNDP1* | -3.883541079 | 1.64E-41 | 6.29E-40 |  |  |  |  |
| *CYP4F35P* | -2.004680978 | 5.16E-08 | 2.28E-07 |  |  |  |  |
| *DCC* | 2.294431225 | 1.36E-13 | 1.04E-12 |  |  |  |  |
| *DQ590589.8* | -2.068972165 | 0.001976427 | 0.004483642 |  |  |  |  |
| *DSC1* | 4.678710912 | 2.25E-48 | 1.16E-46 |  |  |  |  |
| *DSC3* | 3.744674702 | 9.55E-33 | 2.42E-31 |  |  |  |  |
| *DSG1* | 3.69730587 | 6.60E-21 | 8.58E-20 |  |  |  |  |
| *DSG3* | 2.519454899 | 1.17E-13 | 9.02E-13 |  |  |  |  |
| *FHOD3* | 2.348935659 | 4.12E-28 | 8.27E-27 |  |  |  |  |
| *GATA6* | -3.962030634 | 6.64E-109 | 2.00E-106 |  |  |  |  |
| *GRP* | 2.031265568 | 7.72E-08 | 3.34E-07 |  |  |  |  |
| *KC6* | 2.471547941 | 8.29E-10 | 4.46E-09 |  |  |  |  |
| *LAMA1* | 2.548578163 | 6.66E-19 | 7.55E-18 |  |  |  |  |
| *LOC100130480* | 2.986853488 | 1.67E-05 | 5.33E-05 |  |  |  |  |
| *LOC284215* | -2.509801994 | 5.69E-09 | 2.82E-08 |  |  |  |  |
| *LOC339298* | 2.2046219 | 0.000281823 | 0.000742797 |  |  |  |  |
| *LOC729950* | -2.764258982 | 3.10E-36 | 9.35E-35 |  |  |  |  |
| *LPIN2* | -2.355444954 | 2.04E-63 | 1.80E-61 |  |  |  |  |
| *MEP1B* | -2.912618936 | 5.03E-28 | 1.00E-26 |  |  |  |  |
| *NETO1* | 3.547637659 | 3.85E-27 | 7.34E-26 |  |  |  |  |
| *ONECUT2* | -4.432529369 | 1.01E-81 | 1.63E-79 |  |  |  |  |
| *PARD6G* | 2.755453959 | 1.18E-71 | 1.34E-69 |  |  |  |  |
| *RNF165* | 4.040356037 | 1.12E-61 | 9.10E-60 |  |  |  |  |
| *SALL3* | 5.008246079 | 1.48E-20 | 1.87E-19 |  |  |  |  |
| *SERPINB12* | 3.091476155 | 3.47E-13 | 2.56E-12 |  |  |  |  |
| *SERPINB13* | 2.029085335 | 5.30E-06 | 1.81E-05 |  |  |  |  |
| *SIGLEC15* | -2.041164663 | 1.11E-17 | 1.17E-16 |  |  |  |  |
| *SNORA73* | 3.285607232 | 1.05E-13 | 8.17E-13 |  |  |  |  |
| *ST8SIA3* | -2.528773396 | 1.59E-07 | 6.59E-07 |  |  |  |  |
| *TNFRSF11A* | -2.966294373 | 2.44E-56 | 1.63E-54 |  |  |  |  |
| *TTR* | -5.963856148 | 8.11E-44 | 3.44E-42 |  |  |  |  |
| *AK097493* | 2.02188543 | 0.004682612 | 0.009867957 |  |  |  |  |
| *B3GNT3* | -3.151030667 | 4.06E-56 | 2.69E-54 |  |  |  |  |
| *BC034929* | -3.188628506 | 2.78E-57 | 1.91E-55 |  |  |  |  |
| *BC062328* | 2.195749696 | 5.47E-10 | 3.01E-09 |  |  |  |  |
| *BSPH1* | 2.089048771 | 0.011520017 | 0.022333033 |  |  |  |  |
| *C19orf21* | -3.767840552 | 8.32E-80 | 1.26E-77 |  |  |  |  |
| *C19orf45* | -2.086722351 | 1.71E-22 | 2.46E-21 |  |  |  |  |
| *C19orf69* | -3.393423182 | 4.11E-25 | 6.94E-24 |  |  |  |  |
| *C19orf77* | -7.662989278 | 4.22E-256 | 1.64E-252 |  |  |  |  |
| *C3P1* | -6.384930073 | 5.21E-49 | 2.75E-47 |  |  |  |  |
| *CACNG6* | -2.936702845 | 1.37E-11 | 8.77E-11 |  |  |  |  |
| *CACNG8* | -2.101398768 | 1.58E-15 | 1.41E-14 |  |  |  |  |
| *CASP14* | 7.885521299 | 1.11E-63 | 9.78E-62 |  |  |  |  |
| *CCDC105* | 2.223050654 | 0.000735351 | 0.001807669 |  |  |  |  |
| *CCL25* | -7.830874876 | 2.58E-77 | 3.60E-75 |  |  |  |  |
| *CDC42EP5* | -3.140017976 | 7.78E-67 | 7.39E-65 |  |  |  |  |
| *CEACAM18* | -5.77358002 | 1.06E-33 | 2.83E-32 |  |  |  |  |
| *CEACAM20* | -4.18222767 | 4.70E-26 | 8.44E-25 |  |  |  |  |
| *CEACAM5* | -3.491682955 | 2.22E-27 | 4.30E-26 |  |  |  |  |
| *CEACAM6* | -2.627361563 | 3.56E-19 | 4.12E-18 |  |  |  |  |
| *CEACAM7* | -2.673008581 | 4.29E-12 | 2.88E-11 |  |  |  |  |
| *CGB8* | 2.236087941 | 5.23E-26 | 9.37E-25 |  |  |  |  |
| *CREB3L3* | -7.427846046 | 4.03E-128 | 1.74E-125 |  |  |  |  |
| *CRLF1* | 2.667505968 | 4.00E-23 | 5.97E-22 |  |  |  |  |
| *CYP2B6* | -7.553008668 | 1.08E-90 | 2.23E-88 |  |  |  |  |
| *CYP2B7P1* | -3.142769825 | 3.25E-27 | 6.23E-26 |  |  |  |  |
| *CYP4F11* | 3.405041573 | 1.21E-28 | 2.49E-27 |  |  |  |  |
| *CYP4F22* | 2.077197652 | 7.61E-09 | 3.71E-08 |  |  |  |  |
| *CYP4F2* | -4.023560847 | 2.38E-29 | 5.04E-28 |  |  |  |  |
| *DPF1* | 2.596976715 | 2.99E-36 | 9.04E-35 |  |  |  |  |
| *DQ590318* | -2.242456895 | 4.21E-09 | 2.11E-08 |  |  |  |  |
| *EFNA2* | -4.438201984 | 2.83E-71 | 3.14E-69 |  |  |  |  |
| *FAM83E* | -2.76064648 | 8.20E-40 | 2.94E-38 |  |  |  |  |
| *FBXO27* | 2.474919091 | 3.58E-30 | 7.91E-29 |  |  |  |  |
| *FCGBP* | -3.481654649 | 1.71E-38 | 5.70E-37 |  |  |  |  |
| *FOXA3* | -6.222973077 | 8.45E-189 | 1.04E-185 |  |  |  |  |
| *GDF15* | -3.25492271 | 3.17E-42 | 1.25E-40 |  |  |  |  |
| *HPN* | -3.170749315 | 2.32E-18 | 2.54E-17 |  |  |  |  |
| *IGFL1* | 3.401943199 | 3.65E-17 | 3.71E-16 |  |  |  |  |
| *IGFL2* | 3.991541334 | 5.87E-39 | 2.01E-37 |  |  |  |  |
| *IGFL3* | 5.889018978 | 1.66E-51 | 9.32E-50 |  |  |  |  |
| *IGSF23* | -3.070025331 | 5.90E-29 | 1.23E-27 |  |  |  |  |
| *IZUMO2* | -2.191991628 | 4.72E-06 | 1.62E-05 |  |  |  |  |
| *KCTD15* | 2.038458988 | 4.45E-59 | 3.28E-57 |  |  |  |  |
| *KLC3* | 2.583810595 | 6.90E-49 | 3.61E-47 |  |  |  |  |
| *KLK14* | 2.553136454 | 7.89E-17 | 7.81E-16 |  |  |  |  |
| *KLK15* | -3.027471064 | 3.10E-14 | 2.51E-13 |  |  |  |  |
| *KLK1* | -2.405389395 | 5.06E-22 | 7.07E-21 |  |  |  |  |
| *KLK3* | -2.653990765 | 4.89E-09 | 2.44E-08 |  |  |  |  |
| *KLK9* | 2.364157637 | 6.92E-13 | 4.99E-12 |  |  |  |  |
| *KRTDAP* | 4.371908076 | 9.05E-23 | 1.32E-21 |  |  |  |  |
| *LAIR2* | -2.021295364 | 1.76E-16 | 1.69E-15 |  |  |  |  |
| *LGALS4* | -9.113397069 | 0 | 0 |  |  |  |  |
| *LGALS7B* | 3.189096672 | 4.52E-17 | 4.57E-16 |  |  |  |  |
| *LGALS7* | 3.31052518 | 2.13E-14 | 1.74E-13 |  |  |  |  |
| *LIM2* | 2.943558391 | 1.21E-10 | 7.08E-10 |  |  |  |  |
| *LOC100507003* | -2.038690211 | 9.50E-10 | 5.08E-09 |  |  |  |  |
| *LOC284344* | 2.394553096 | 1.18E-10 | 6.94E-10 |  |  |  |  |
| *LOC390940* | 2.153502879 | 1.27E-27 | 2.49E-26 |  |  |  |  |
| *MBD3L1* | 2.375336845 | 0.003443286 | 0.007449256 |  |  |  |  |
| *MIA* | -2.917094165 | 2.98E-16 | 2.82E-15 |  |  |  |  |
| *MIR3189* | -4.117956853 | 5.93E-35 | 1.69E-33 |  |  |  |  |
| *MIR517A* | 2.151592317 | 0.016200901 | 0.03032339 |  |  |  |  |
| *MIR7-3HG* | -2.51634562 | 2.41E-07 | 9.78E-07 |  |  |  |  |
| *Mir_324.4* | -3.63015718 | 1.13E-13 | 8.75E-13 |  |  |  |  |
| *NANOS3* | -2.134229219 | 1.14E-21 | 1.56E-20 |  |  |  |  |
| *NKPD1* | 2.375982649 | 7.84E-32 | 1.89E-30 |  |  |  |  |
| *NLRP13* | 2.556985136 | 7.83E-05 | 0.000225972 |  |  |  |  |
| *NPHS1* | -2.345743324 | 3.14E-13 | 2.33E-12 |  |  |  |  |
| *NTF4* | 3.018262909 | 6.14E-59 | 4.47E-57 |  |  |  |  |
| *OLFM2* | 2.153336067 | 1.08E-27 | 2.12E-26 |  |  |  |  |
| *ONECUT3* | -5.376565763 | 9.62E-38 | 3.10E-36 |  |  |  |  |
| *OR1M1* | 2.867909997 | 0.000164765 | 0.000451484 |  |  |  |  |
| *OR7A10* | 2.476026377 | 0.004136685 | 0.008813207 |  |  |  |  |
| *OR7A17* | 2.8609302 | 0.00149902 | 0.003484416 |  |  |  |  |
| *OR7A5* | 5.443078171 | 5.38E-41 | 2.02E-39 |  |  |  |  |
| *OR7C1* | 3.030576194 | 7.48E-13 | 5.37E-12 |  |  |  |  |
| *OR7C2* | 2.067033348 | 0.010524898 | 0.020599379 |  |  |  |  |
| *OR7D4* | 3.078115148 | 0.000132735 | 0.000370022 |  |  |  |  |
| *OR7E24* | 2.400144281 | 0.002616213 | 0.005803221 |  |  |  |  |
| *OR7G3* | 2.663803853 | 0.000260401 | 0.000689881 |  |  |  |  |
| *PALM3* | -3.04925743 | 1.85E-33 | 4.86E-32 |  |  |  |  |
| *PAPL* | 2.336545301 | 3.51E-12 | 2.38E-11 |  |  |  |  |
| *PDE4C* | -2.788843533 | 6.54E-32 | 1.58E-30 |  |  |  |  |
| *PLAC2* | 3.27734568 | 7.20E-36 | 2.14E-34 |  |  |  |  |
| *PNMAL1* | 2.091604791 | 5.89E-13 | 4.27E-12 |  |  |  |  |
| *PRG1* | 2.529488192 | 2.36E-11 | 1.48E-10 |  |  |  |  |
| *PRKCG* | -4.867961357 | 4.78E-58 | 3.38E-56 |  |  |  |  |
| *PRODH2* | -2.816726419 | 3.36E-08 | 1.52E-07 |  |  |  |  |
| *PSG5* | 2.759844591 | 1.69E-11 | 1.07E-10 |  |  |  |  |
| *PTPRH* | -3.655443438 | 6.11E-61 | 4.82E-59 |  |  |  |  |
| *PTPRS* | 2.250844803 | 1.32E-41 | 5.06E-40 |  |  |  |  |
| *RDH8* | -3.009587619 | 4.17E-17 | 4.23E-16 |  |  |  |  |
| *RGL3* | -2.865516541 | 1.29E-33 | 3.44E-32 |  |  |  |  |
| *RHPN2* | -3.779737241 | 7.43E-128 | 3.11E-125 |  |  |  |  |
| *RYR1* | 3.363612434 | 1.78E-59 | 1.33E-57 |  |  |  |  |
| *S1PR5* | 3.798666642 | 7.52E-80 | 1.15E-77 |  |  |  |  |
| *SBSN* | 2.11400799 | 1.90E-06 | 6.88E-06 |  |  |  |  |
| *SELV* | 3.377069976 | 3.80E-12 | 2.57E-11 |  |  |  |  |
| *SGK110* | -2.07082189 | 2.96E-22 | 4.20E-21 |  |  |  |  |
| *SHD* | -3.583146503 | 8.03E-39 | 2.72E-37 |  |  |  |  |
| *SHISA7* | 2.147919652 | 2.15E-23 | 3.25E-22 |  |  |  |  |
| *SLC1A6* | 4.489533532 | 9.94E-22 | 1.36E-20 |  |  |  |  |
| *SLC5A5* | -3.582891789 | 2.26E-26 | 4.13E-25 |  |  |  |  |
| *SLC7A9* | -3.207589878 | 3.75E-38 | 1.23E-36 |  |  |  |  |
| *SNAR-C4* | 2.003046866 | 0.024353766 | 0.043660953 |  |  |  |  |
| *SPACA4* | -2.445855125 | 1.63E-35 | 4.76E-34 |  |  |  |  |
| *SULT2A1* | -4.833858579 | 6.70E-19 | 7.60E-18 |  |  |  |  |
| *THEG* | 2.093115626 | 1.41E-08 | 6.68E-08 |  |  |  |  |
| *TJP3* | -3.386191388 | 2.11E-51 | 1.18E-49 |  |  |  |  |
| *TM6SF2* | -2.682813562 | 1.06E-25 | 1.86E-24 |  |  |  |  |
| *TMEM150B* | -3.601570314 | 3.07E-71 | 3.38E-69 |  |  |  |  |
| *TNNT1* | 2.226734287 | 5.04E-16 | 4.69E-15 |  |  |  |  |
| *TSKS* | 3.857137892 | 1.09E-32 | 2.76E-31 |  |  |  |  |
| *TUBB4A* | 2.008495432 | 2.85E-11 | 1.77E-10 |  |  |  |  |
| *UCA1* | -2.912059515 | 2.33E-20 | 2.91E-19 |  |  |  |  |
| *ZIM3* | 2.566193674 | 5.16E-05 | 0.000153 |  |  |  |  |
| *ZNF69* | -3.125585914 | 1.10E-94 | 2.44E-92 |  |  |  |  |
| *ZNF729* | 2.544730148 | 0.0002346 | 0.00062586 |  |  |  |  |
| *ABCG5* | -3.579766504 | 3.20E-45 | 1.42E-43 |  |  |  |  |
| *ABCG8* | -4.533138379 | 2.83E-46 | 1.33E-44 |  |  |  |  |
| *ADAM23* | 4.023704281 | 4.14E-56 | 2.73E-54 |  |  |  |  |
| *ADD2* | 3.637717702 | 7.35E-45 | 3.24E-43 |  |  |  |  |
| *AGXT* | -3.449128132 | 9.80E-21 | 1.26E-19 |  |  |  |  |
| *AK125871* | -2.395337056 | 1.89E-38 | 6.31E-37 |  |  |  |  |
| *AK127124* | 2.753614496 | 2.99E-31 | 6.99E-30 |  |  |  |  |
| *AK311291* | 2.576554038 | 5.81E-16 | 5.37E-15 |  |  |  |  |
| *ALK* | 2.114171627 | 1.84E-21 | 2.47E-20 |  |  |  |  |
| *ALPI* | -8.022958125 | 9.54E-75 | 1.22E-72 |  |  |  |  |
| *ALPPL2* | -5.831652544 | 5.56E-49 | 2.93E-47 |  |  |  |  |
| *ALPP* | -3.84677024 | 5.43E-23 | 8.04E-22 |  |  |  |  |
| *APOB* | -6.075251797 | 2.28E-58 | 1.64E-56 |  |  |  |  |
| *AQP12A* | -4.05440101 | 1.02E-16 | 9.97E-16 |  |  |  |  |
| *AQP12B* | -3.828022666 | 6.16E-15 | 5.27E-14 |  |  |  |  |
| *ARHGEF4* | 2.779216198 | 1.78E-41 | 6.77E-40 |  |  |  |  |
| *AX746677* | -3.82108372 | 8.25E-24 | 1.28E-22 |  |  |  |  |
| *AX746725* | 2.085231765 | 1.03E-18 | 1.15E-17 |  |  |  |  |
| *AX747402* | 2.053016489 | 0.003224286 | 0.007019069 |  |  |  |  |
| *AX747413* | 2.065821704 | 7.42E-23 | 1.09E-21 |  |  |  |  |
| *B3GNT7* | -3.221181631 | 1.36E-69 | 1.42E-67 |  |  |  |  |
| *BC008215* | 2.095007831 | 2.43E-21 | 3.23E-20 |  |  |  |  |
| *BC016143.5* | 2.453658396 | 1.37E-05 | 4.41E-05 |  |  |  |  |
| *BC016143.6* | 7.286651527 | 3.90E-42 | 1.54E-40 |  |  |  |  |
| *BC016831* | -4.363923105 | 4.90E-16 | 4.56E-15 |  |  |  |  |
| *BC017935* | 2.837325928 | 2.58E-08 | 1.18E-07 |  |  |  |  |
| *BC040311* | 2.511720715 | 1.99E-05 | 6.27E-05 |  |  |  |  |
| *BC040861* | 2.191324153 | 1.49E-12 | 1.04E-11 |  |  |  |  |
| *BC051759* | 3.561596142 | 3.35E-36 | 1.01E-34 |  |  |  |  |
| *BMP10* | 2.043454999 | 0.000321204 | 0.000838457 |  |  |  |  |
| *C1QL2* | 2.031670335 | 0.000109893 | 0.000310488 |  |  |  |  |
| *C2orf70* | -2.109148778 | 6.75E-20 | 8.20E-19 |  |  |  |  |
| *C2orf71* | 3.654674532 | 6.40E-33 | 1.64E-31 |  |  |  |  |
| *C2orf72* | -5.10485429 | 2.43E-119 | 8.80E-117 |  |  |  |  |
| *C2orf82* | -2.018155957 | 3.59E-43 | 1.47E-41 |  |  |  |  |
| *C2orf89* | -2.927767958 | 4.33E-34 | 1.17E-32 |  |  |  |  |
| *CCDC108* | -3.493531123 | 6.58E-33 | 1.68E-31 |  |  |  |  |
| *CCDC140* | 6.691170691 | 2.28E-20 | 2.86E-19 |  |  |  |  |
| *CFC1B* | -2.608108643 | 0.002326111 | 0.005209921 |  |  |  |  |
| *CIB4* | -3.848990584 | 3.17E-25 | 5.39E-24 |  |  |  |  |
| *CPS1-IT1* | -3.783768595 | 1.87E-08 | 8.72E-08 |  |  |  |  |
| *CPS1* | -4.794668306 | 9.30E-46 | 4.22E-44 |  |  |  |  |
| *CXCR7* | 2.394904074 | 1.69E-34 | 4.71E-33 |  |  |  |  |
| *CYP27C1* | 2.587019782 | 2.47E-22 | 3.51E-21 |  |  |  |  |
| *DAPL1* | 3.384851481 | 2.45E-21 | 3.25E-20 |  |  |  |  |
| *DES* | 2.48546002 | 3.04E-12 | 2.08E-11 |  |  |  |  |
| *DIRC3* | 2.536810335 | 2.05E-35 | 5.95E-34 |  |  |  |  |
| *DLX1* | 3.20803478 | 1.52E-30 | 3.41E-29 |  |  |  |  |
| *DLX2* | 2.307392991 | 5.06E-16 | 4.71E-15 |  |  |  |  |
| *DNAH6* | -2.266803098 | 8.16E-28 | 1.61E-26 |  |  |  |  |
| *DNAJB3* | 2.605415134 | 5.79E-16 | 5.35E-15 |  |  |  |  |
| *DNER* | 2.233728002 | 5.04E-14 | 4.01E-13 |  |  |  |  |
| *DPP4* | -2.641717003 | 7.48E-38 | 2.43E-36 |  |  |  |  |
| *DPYSL5* | 3.010368847 | 8.28E-16 | 7.56E-15 |  |  |  |  |
| *DQ571479.6* | -2.085262771 | 3.78E-19 | 4.36E-18 |  |  |  |  |
| *ECEL1P2* | -2.602534351 | 6.64E-10 | 3.61E-09 |  |  |  |  |
| *EN1* | 7.756941259 | 3.20E-149 | 1.89E-146 |  |  |  |  |
| *EPCAM* | -2.353117871 | 2.16E-49 | 1.16E-47 |  |  |  |  |
| *FABP1* | -9.208832485 | 6.47E-84 | 1.11E-81 |  |  |  |  |
| *FAM123C* | -3.06239598 | 1.95E-14 | 1.60E-13 |  |  |  |  |
| *FEV* | -3.344827657 | 6.05E-20 | 7.37E-19 |  |  |  |  |
| *FOXI3* | 7.834259015 | 6.68E-67 | 6.39E-65 |  |  |  |  |
| *FSHR* | 2.412097044 | 7.49E-05 | 0.000216783 |  |  |  |  |
| *FZD5* | -2.262946622 | 2.58E-77 | 3.60E-75 |  |  |  |  |
| *GAL3ST2* | -3.169660998 | 8.37E-30 | 1.82E-28 |  |  |  |  |
| *GALM* | -2.326525207 | 1.78E-74 | 2.26E-72 |  |  |  |  |
| *GALNT13* | 2.606231099 | 6.48E-16 | 5.96E-15 |  |  |  |  |
| *GALNT14* | 2.646042705 | 4.20E-28 | 8.43E-27 |  |  |  |  |
| *GCKR* | -3.210392843 | 1.10E-38 | 3.70E-37 |  |  |  |  |
| *GKN1* | -4.422510129 | 2.11E-24 | 3.40E-23 |  |  |  |  |
| *GKN2* | -4.307093206 | 5.90E-20 | 7.20E-19 |  |  |  |  |
| *GLI2* | 2.352873964 | 6.29E-24 | 9.85E-23 |  |  |  |  |
| *GPC1* | 2.789595805 | 3.03E-71 | 3.35E-69 |  |  |  |  |
| *GPR1* | 2.081626585 | 2.61E-14 | 2.12E-13 |  |  |  |  |
| *GPR35* | -5.203626051 | 0 | 0 |  |  |  |  |
| *GRB14* | -2.118636797 | 2.20E-20 | 2.76E-19 |  |  |  |  |
| *HOXD-AS2* | 2.06068412 | 8.59E-15 | 7.25E-14 |  |  |  |  |
| *HOXD10* | 2.90025328 | 7.36E-23 | 1.08E-21 |  |  |  |  |
| *HOXD11* | 2.953828658 | 1.66E-14 | 1.37E-13 |  |  |  |  |
| *HOXD13* | 3.575517026 | 3.68E-18 | 3.98E-17 |  |  |  |  |
| *IHH* | -8.831926526 | 3.75E-232 | 1.02E-228 |  |  |  |  |
| *IL1A* | 2.78195657 | 9.94E-22 | 1.36E-20 |  |  |  |  |
| *IL1F10* | 4.041983558 | 2.65E-21 | 3.51E-20 |  |  |  |  |
| *IL36B* | 2.3439644 | 4.33E-08 | 1.93E-07 |  |  |  |  |
| *IL36G* | 3.00575156 | 4.40E-16 | 4.11E-15 |  |  |  |  |
| *IL36RN* | 2.279879094 | 1.08E-07 | 4.60E-07 |  |  |  |  |
| *IQCA1* | 2.854726194 | 1.47E-38 | 4.91E-37 |  |  |  |  |
| *KCNJ3* | -5.427072433 | 7.64E-52 | 4.36E-50 |  |  |  |  |
| *LINC00486* | 3.2396285 | 5.90E-19 | 6.73E-18 |  |  |  |  |
| *LOC100286922* | 2.559261347 | 5.25E-14 | 4.17E-13 |  |  |  |  |
| *LOC100287010* | 2.528763144 | 0.008854362 | 0.017589586 |  |  |  |  |
| *LOC100505964* | 2.384266785 | 0.000651203 | 0.001612926 |  |  |  |  |
| *LOC150622* | -2.719578355 | 7.49E-08 | 3.24E-07 |  |  |  |  |
| *LOC200726* | 4.285570316 | 1.24E-17 | 1.30E-16 |  |  |  |  |
| *LOC200772* | -2.430383048 | 4.39E-11 | 2.68E-10 |  |  |  |  |
| *LOC285000* | -3.418263796 | 5.28E-14 | 4.20E-13 |  |  |  |  |
| *LOC285084* | 3.960795706 | 1.13E-39 | 4.02E-38 |  |  |  |  |
| *LOC339807* | 2.1386952 | 1.92E-16 | 1.84E-15 |  |  |  |  |
| *LOC348761* | -3.062167774 | 1.26E-42 | 5.11E-41 |  |  |  |  |
| *LOC388942* | -3.351740584 | 3.23E-12 | 2.20E-11 |  |  |  |  |
| *LOC389023* | 2.186772132 | 0.000249001 | 0.000661552 |  |  |  |  |
| *LOC440925* | -2.833917436 | 6.48E-25 | 1.08E-23 |  |  |  |  |
| *LOC727982* | 3.323874074 | 4.92E-09 | 2.45E-08 |  |  |  |  |
| *LOC730811* | 2.148900342 | 0.026746916 | 0.04751548 |  |  |  |  |
| *LRP1B* | 3.901401007 | 2.06E-40 | 7.54E-39 |  |  |  |  |
| *MGAT4A* | -2.361371726 | 6.69E-55 | 4.19E-53 |  |  |  |  |
| *MIR3131* | -7.873127715 | 9.84E-56 | 6.44E-54 |  |  |  |  |
| *MIR559* | -2.984067269 | 4.01E-50 | 2.19E-48 |  |  |  |  |
| *MLPH* | -3.740898033 | 5.11E-63 | 4.43E-61 |  |  |  |  |
| *MOGAT1* | -2.176734915 | 6.00E-09 | 2.96E-08 |  |  |  |  |
| *MPP4* | 2.224473748 | 3.78E-24 | 6.03E-23 |  |  |  |  |
| *MSGN1* | 5.324806632 | 2.19E-34 | 6.06E-33 |  |  |  |  |
| *MYADML* | 2.786377952 | 7.42E-10 | 4.01E-09 |  |  |  |  |
| *MYO3B* | 2.203600652 | 2.76E-17 | 2.82E-16 |  |  |  |  |
| *MYO7B* | -6.060687845 | 4.54E-143 | 2.47E-140 |  |  |  |  |
| *NAT8B* | -2.465550145 | 2.25E-18 | 2.46E-17 |  |  |  |  |
| *NEU2* | 3.500048868 | 9.85E-16 | 8.94E-15 |  |  |  |  |
| *NEU4* | -3.939199898 | 1.23E-47 | 6.13E-46 |  |  |  |  |
| *NEUROD1* | -2.710562398 | 7.28E-09 | 3.56E-08 |  |  |  |  |
| *NOSTRIN* | -3.152465016 | 6.65E-123 | 2.54E-120 |  |  |  |  |
| *NPPC* | 3.948356469 | 6.41E-28 | 1.27E-26 |  |  |  |  |
| *NRXN1* | 2.356884183 | 1.05E-12 | 7.41E-12 |  |  |  |  |
| *NXPH2* | 4.779365431 | 3.19E-20 | 3.96E-19 |  |  |  |  |
| *OR6B3* | 2.602927821 | 0.000417557 | 0.001069623 |  |  |  |  |
| *OSBPL6* | 2.470811805 | 1.30E-40 | 4.77E-39 |  |  |  |  |
| *OTOS* | 2.066690626 | 0.001293375 | 0.003041799 |  |  |  |  |
| *PABL* | -2.608216752 | 2.36E-25 | 4.05E-24 |  |  |  |  |
| *PAX3* | 6.193075818 | 1.62E-39 | 5.75E-38 |  |  |  |  |
| *PCDP1* | -3.405089653 | 6.31E-32 | 1.53E-30 |  |  |  |  |
| *PDE11A* | -2.865404206 | 3.01E-26 | 5.45E-25 |  |  |  |  |
| *PKDCC* | -3.174985634 | 1.32E-48 | 6.86E-47 |  |  |  |  |
| *POMC* | 3.832384131 | 1.29E-43 | 5.39E-42 |  |  |  |  |
| *PRKAG3* | 2.266880964 | 1.90E-12 | 1.31E-11 |  |  |  |  |
| *PRLH* | -2.454119824 | 1.33E-06 | 4.93E-06 |  |  |  |  |
| *RAB17* | -3.019080308 | 2.28E-56 | 1.53E-54 |  |  |  |  |
| *RAD51AP2* | 2.047304565 | 5.06E-19 | 5.80E-18 |  |  |  |  |
| *REG1A* | -10.41535321 | 1.16E-127 | 4.79E-125 |  |  |  |  |
| *REG1B* | -8.238501941 | 1.01E-50 | 5.60E-49 |  |  |  |  |
| *REG3A* | -9.980376588 | 6.28E-95 | 1.40E-92 |  |  |  |  |
| *REG3G* | -6.044895692 | 5.35E-22 | 7.46E-21 |  |  |  |  |
| *RN7SK.48* | 2.294911778 | 1.25E-06 | 4.64E-06 |  |  |  |  |
| *RN7SK.51* | 2.483818713 | 8.41E-09 | 4.07E-08 |  |  |  |  |
| *RN7SK.55* | 2.141223926 | 0.020709944 | 0.037774026 |  |  |  |  |
| *SCN9A* | 3.157288055 | 6.62E-27 | 1.25E-25 |  |  |  |  |
| *SCTR* | -2.38882191 | 9.29E-17 | 9.12E-16 |  |  |  |  |
| *SERPINE2* | 2.020200852 | 2.00E-24 | 3.23E-23 |  |  |  |  |
| *SLC19A3* | -4.669902002 | 2.54E-69 | 2.62E-67 |  |  |  |  |
| *SLC40A1* | -2.311834164 | 1.17E-46 | 5.66E-45 |  |  |  |  |
| *SLC9A4* | -2.393386641 | 1.90E-11 | 1.20E-10 |  |  |  |  |
| *SP5* | -4.341563879 | 3.78E-53 | 2.24E-51 |  |  |  |  |
| *SP9* | 3.397924815 | 8.38E-19 | 9.46E-18 |  |  |  |  |
| *ST6GAL2* | 4.084633242 | 4.78E-45 | 2.12E-43 |  |  |  |  |
| *SULT1C2P1* | -4.771189616 | 1.51E-47 | 7.50E-46 |  |  |  |  |
| *SULT1C2* | -6.77179546 | 4.61E-157 | 3.13E-154 |  |  |  |  |
| *SULT1C3* | -4.994806028 | 4.50E-33 | 1.16E-31 |  |  |  |  |
| *SULT1C4* | -2.292121821 | 6.57E-22 | 9.10E-21 |  |  |  |  |
| *TEKT4* | 2.05546715 | 6.50E-12 | 4.29E-11 |  |  |  |  |
| *TM4SF20* | -9.270386174 | 2.59E-192 | 3.35E-189 |  |  |  |  |
| *TRIM54* | -3.619680213 | 2.50E-30 | 5.56E-29 |  |  |  |  |
| *TRNA_Pseudo.54* | 2.040815601 | 0.011790945 | 0.022801311 |  |  |  |  |
| *VAX2* | 2.085739047 | 2.98E-18 | 3.24E-17 |  |  |  |  |
| *VIL1* | -7.507579048 | 1.12E-151 | 7.22E-149 |  |  |  |  |
| *VIT* | 2.686355431 | 1.25E-15 | 1.13E-14 |  |  |  |  |
| *VSNL1* | 2.327724403 | 1.30E-21 | 1.76E-20 |  |  |  |  |
| *VWA3B* | -2.135162566 | 3.63E-21 | 4.78E-20 |  |  |  |  |
| *XIRP2* | 2.404891453 | 1.64E-08 | 7.69E-08 |  |  |  |  |
| *Y_RNA.50* | 3.186585056 | 1.73E-10 | 9.99E-10 |  |  |  |  |
| *AF143870* | -4.872835119 | 5.01E-64 | 4.47E-62 |  |  |  |  |
| *AK056267* | -2.714654842 | 0.000147577 | 0.000407757 |  |  |  |  |
| *AK125594* | -3.949087098 | 1.08E-17 | 1.14E-16 |  |  |  |  |
| *APCDD1L* | 2.986806572 | 1.10E-26 | 2.04E-25 |  |  |  |  |
| *AX746683* | -2.056321197 | 6.10E-23 | 9.00E-22 |  |  |  |  |
| *AX747070* | 2.477331121 | 6.62E-07 | 2.55E-06 |  |  |  |  |
| *AX747171* | 4.620614433 | 4.56E-60 | 3.50E-58 |  |  |  |  |
| *AX747649* | -2.559439064 | 4.42E-17 | 4.47E-16 |  |  |  |  |
| *BANF2* | -2.285038666 | 6.06E-06 | 2.05E-05 |  |  |  |  |
| *BC027448* | 3.824764184 | 7.17E-41 | 2.67E-39 |  |  |  |  |
| *BC043288* | 2.037870281 | 0.015205158 | 0.028653116 |  |  |  |  |
| *BC071794* | -5.648828761 | 8.93E-82 | 1.46E-79 |  |  |  |  |
| *BC141903* | -2.473859051 | 3.78E-14 | 3.04E-13 |  |  |  |  |
| *BCAS1* | -4.165572914 | 1.06E-55 | 6.90E-54 |  |  |  |  |
| *BIRC7* | -2.256735449 | 3.58E-16 | 3.36E-15 |  |  |  |  |
| *BPIFA2* | -3.36372527 | 1.21E-14 | 1.01E-13 |  |  |  |  |
| *BPIFA3* | 2.918415655 | 0.000435836 | 0.001112565 |  |  |  |  |
| *BPIFB1* | -4.509887555 | 3.99E-23 | 5.96E-22 |  |  |  |  |
| *BPIFB2* | -3.442752424 | 2.75E-31 | 6.45E-30 |  |  |  |  |
| *C20orf141* | 2.573892685 | 5.92E-11 | 3.57E-10 |  |  |  |  |
| *C20orf197* | 2.989049548 | 1.82E-29 | 3.89E-28 |  |  |  |  |
| *C20orf85* | -2.887893154 | 6.04E-10 | 3.30E-09 |  |  |  |  |
| *CDH26* | 2.594990654 | 8.27E-24 | 1.28E-22 |  |  |  |  |
| *CHRNA4* | 2.614268499 | 2.13E-09 | 1.10E-08 |  |  |  |  |
| *CYP24A1* | 3.851651923 | 1.11E-34 | 3.14E-33 |  |  |  |  |
| *DEFB118* | 2.607181347 | 2.78E-06 | 9.81E-06 |  |  |  |  |
| *DEFB122.1* | 2.52895582 | 0.001501413 | 0.003489382 |  |  |  |  |
| *DEFB122.2* | 2.356766899 | 0.0021716 | 0.004892088 |  |  |  |  |
| *DEFB127* | 2.632557626 | 0.004740837 | 0.009972865 |  |  |  |  |
| *DZANK1-AS1* | 3.977107066 | 2.11E-10 | 1.21E-09 |  |  |  |  |
| *EDN3* | -2.759614476 | 1.10E-12 | 7.75E-12 |  |  |  |  |
| *FAM83C* | 2.742682176 | 1.18E-14 | 9.89E-14 |  |  |  |  |
| *FER1L4.1* | -3.10489618 | 5.46E-55 | 3.45E-53 |  |  |  |  |
| *FER1L4.2* | -3.010039384 | 1.15E-51 | 6.56E-50 |  |  |  |  |
| *FOXA2* | -4.640164465 | 3.13E-61 | 2.51E-59 |  |  |  |  |
| *GATA5* | -3.953630068 | 5.18E-26 | 9.29E-25 |  |  |  |  |
| *HNF4A* | -8.029603019 | 4.16E-282 | 2.26E-278 |  |  |  |  |
| *INSM1* | -3.869163861 | 4.72E-26 | 8.48E-25 |  |  |  |  |
| *KCNG1* | 2.201173073 | 9.46E-19 | 1.06E-17 |  |  |  |  |
| *KCNK15* | -2.336430246 | 7.27E-24 | 1.13E-22 |  |  |  |  |
| *KCNQ2.1* | -3.209716367 | 1.04E-08 | 4.98E-08 |  |  |  |  |
| *LINC00261* | -7.645954932 | 4.35E-98 | 1.04E-95 |  |  |  |  |
| *LINC00494* | -3.646192811 | 1.08E-36 | 3.32E-35 |  |  |  |  |
| *LOC100127888* | -2.199040257 | 1.69E-29 | 3.62E-28 |  |  |  |  |
| *LOC100131208* | 2.435267174 | 1.57E-11 | 1.00E-10 |  |  |  |  |
| *LOC149773* | 2.923443481 | 1.79E-26 | 3.31E-25 |  |  |  |  |
| *LOC200261* | 2.335282044 | 1.23E-12 | 8.64E-12 |  |  |  |  |
| *LOC339593* | 3.503089536 | 5.70E-15 | 4.90E-14 |  |  |  |  |
| *LOC63930* | -2.380933424 | 1.08E-09 | 5.75E-09 |  |  |  |  |
| *LOC643406* | -2.443184527 | 7.05E-09 | 3.45E-08 |  |  |  |  |
| *LRRN4* | -2.090591959 | 1.87E-18 | 2.05E-17 |  |  |  |  |
| *MACROD2* | -3.006558033 | 1.41E-35 | 4.14E-34 |  |  |  |  |
| *MIR1257* | -2.389473719 | 1.17E-08 | 5.59E-08 |  |  |  |  |
| *MIR3646* | -6.112728425 | 1.94E-38 | 6.44E-37 |  |  |  |  |
| *MIR4756* | -3.334680011 | 4.51E-11 | 2.75E-10 |  |  |  |  |
| *MYT1* | -3.097689609 | 1.77E-22 | 2.55E-21 |  |  |  |  |
| *Metazoa_SRP.60* | 2.32878509 | 0.001089678 | 0.002602847 |  |  |  |  |
| *NKX2-4* | 4.432607346 | 1.64E-09 | 8.58E-09 |  |  |  |  |
| *NTSR1* | -3.554326525 | 4.39E-24 | 6.96E-23 |  |  |  |  |
| *PAK7* | 3.198655423 | 3.21E-15 | 2.80E-14 |  |  |  |  |
| *PAX1* | 3.670487167 | 1.31E-13 | 1.01E-12 |  |  |  |  |
| *PCK1* | -4.983235662 | 6.26E-45 | 2.77E-43 |  |  |  |  |
| *PCSK2* | 2.382590976 | 3.28E-10 | 1.85E-09 |  |  |  |  |
| *PRNP* | 2.12704406 | 2.40E-74 | 2.99E-72 |  |  |  |  |
| *PTPRT* | 2.232130074 | 1.25E-09 | 6.61E-09 |  |  |  |  |
| *ProSAPiP1* | -2.065947744 | 8.27E-49 | 4.32E-47 |  |  |  |  |
| *R3HDML* | -4.496837422 | 9.53E-42 | 3.69E-40 |  |  |  |  |
| *SEMG2* | -5.598765933 | 1.70E-48 | 8.81E-47 |  |  |  |  |
| *SGK2* | -3.834294991 | 1.91E-74 | 2.41E-72 |  |  |  |  |
| *SLC17A9* | -2.939856885 | 2.58E-48 | 1.32E-46 |  |  |  |  |
| *SRMS* | -2.062743229 | 4.97E-18 | 5.34E-17 |  |  |  |  |
| *SSTR4* | 2.549651259 | 8.25E-08 | 3.55E-07 |  |  |  |  |
| *TCF15* | 2.531105048 | 1.81E-24 | 2.94E-23 |  |  |  |  |
| *TFAP2C* | 2.785819011 | 8.43E-39 | 2.85E-37 |  |  |  |  |
| *THBD* | 2.413521461 | 4.95E-39 | 1.70E-37 |  |  |  |  |
| *TMEM239* | 2.425305491 | 8.10E-06 | 2.69E-05 |  |  |  |  |
| *TMEM74B* | -2.168467424 | 6.35E-38 | 2.07E-36 |  |  |  |  |
| *TNNC2* | -2.086903661 | 2.70E-18 | 2.95E-17 |  |  |  |  |
| *WFDC12* | 2.156483954 | 8.74E-08 | 3.75E-07 |  |  |  |  |
| *WFDC5* | 2.388398305 | 8.35E-10 | 4.48E-09 |  |  |  |  |
| *XKR7* | -2.446584957 | 8.48E-09 | 4.11E-08 |  |  |  |  |
| *AX747935* | 2.837002821 | 6.07E-17 | 6.06E-16 |  |  |  |  |
| *B3GALT5* | -3.322904268 | 8.87E-30 | 1.93E-28 |  |  |  |  |
| *BAGE* | 2.06710828 | 0.000146736 | 0.000405764 |  |  |  |  |
| *CBR1* | 2.102614925 | 5.04E-41 | 1.90E-39 |  |  |  |  |
| *CBR3* | 2.142885997 | 3.31E-37 | 1.05E-35 |  |  |  |  |
| *CHODL-AS1* | 3.111680906 | 1.92E-13 | 1.45E-12 |  |  |  |  |
| *CHODL* | 4.915270194 | 3.62E-91 | 7.62E-89 |  |  |  |  |
| *CLDN8* | 3.637859257 | 7.04E-18 | 7.50E-17 |  |  |  |  |
| *CLIC6* | -2.296788419 | 1.00E-19 | 1.20E-18 |  |  |  |  |
| *D21S2088E* | 2.384616854 | 0.002854005 | 0.006285521 |  |  |  |  |
| *DQ588725* | -3.893121202 | 2.38E-15 | 2.10E-14 |  |  |  |  |
| *DQ590668* | -3.691471283 | 2.21E-09 | 1.14E-08 |  |  |  |  |
| *DQ601137* | -3.459375319 | 1.64E-07 | 6.81E-07 |  |  |  |  |
| *DSCAM* | 3.631894567 | 7.36E-28 | 1.46E-26 |  |  |  |  |
| *FAM3B* | -2.179836135 | 7.92E-13 | 5.68E-12 |  |  |  |  |
| *KCNE2* | -3.297107042 | 2.54E-31 | 5.99E-30 |  |  |  |  |
| *KCNJ15* | 2.535793273 | 2.00E-26 | 3.68E-25 |  |  |  |  |
| *KRTAP10-4* | -2.867184675 | 1.57E-05 | 5.00E-05 |  |  |  |  |
| *KRTAP11-1* | 4.385925327 | 6.65E-12 | 4.39E-11 |  |  |  |  |
| *KRTAP13-1* | 3.68680044 | 1.84E-07 | 7.58E-07 |  |  |  |  |
| *KRTAP19-1* | 7.526712614 | 1.29E-30 | 2.92E-29 |  |  |  |  |
| *KRTAP19-5* | 3.294272353 | 3.15E-05 | 9.63E-05 |  |  |  |  |
| *KRTAP21-2* | 3.457286225 | 1.47E-05 | 4.72E-05 |  |  |  |  |
| *KRTAP24-1* | 2.077181368 | 0.00640574 | 0.013140401 |  |  |  |  |
| *KRTAP7-1* | 2.558978128 | 0.002408272 | 0.005374887 |  |  |  |  |
| *LINC00114* | -3.906325478 | 5.03E-29 | 1.05E-27 |  |  |  |  |
| *LINC00161* | 2.153470802 | 3.51E-16 | 3.30E-15 |  |  |  |  |
| *LINC00163* | 2.242805946 | 8.30E-10 | 4.46E-09 |  |  |  |  |
| *LINC00307* | 2.637817163 | 0.000606497 | 0.001508714 |  |  |  |  |
| *LINC00317* | 2.761635161 | 0.003872466 | 0.008296484 |  |  |  |  |
| *LINC00320* | 2.918574425 | 4.93E-06 | 1.69E-05 |  |  |  |  |
| *LOC100133286* | 2.103027463 | 2.43E-39 | 8.49E-38 |  |  |  |  |
| *LOC284837* | 2.485412943 | 1.71E-53 | 1.02E-51 |  |  |  |  |
| *MIR125B2* | 2.79277511 | 7.46E-06 | 2.49E-05 |  |  |  |  |
| *MIR3197* | -2.285750685 | 8.57E-13 | 6.13E-12 |  |  |  |  |
| *Mir_548.44* | 2.217941181 | 0.006172391 | 0.01269583 |  |  |  |  |
| *RSPH1* | -3.573681416 | 6.81E-87 | 1.29E-84 |  |  |  |  |
| *SLC37A1* | -2.414174905 | 1.24E-85 | 2.25E-83 |  |  |  |  |
| *TFF1* | -8.233260642 | 4.85E-101 | 1.25E-98 |  |  |  |  |
| *TFF2* | -8.236973636 | 5.22E-81 | 8.30E-79 |  |  |  |  |
| *TFF3* | -3.567212237 | 2.09E-26 | 3.83E-25 |  |  |  |  |
| *TMPRSS2* | -3.9111729 | 1.34E-56 | 9.02E-55 |  |  |  |  |
| *TMPRSS3* | -4.512738697 | 2.13E-74 | 2.67E-72 |  |  |  |  |
| *TPTE* | 3.239444441 | 2.91E-11 | 1.80E-10 |  |  |  |  |
| *U3.6* | -3.209871355 | 1.85E-19 | 2.18E-18 |  |  |  |  |
| *AK097787* | -2.749722167 | 6.83E-11 | 4.09E-10 |  |  |  |  |
| *AK123632* | 2.234773512 | 0.002804583 | 0.006186204 |  |  |  |  |
| *BAIAP2L2* | -2.4097526 | 1.89E-43 | 7.85E-42 |  |  |  |  |
| *BC035867* | 4.425459618 | 2.21E-31 | 5.23E-30 |  |  |  |  |
| *BPIFC* | 2.412047177 | 2.64E-08 | 1.21E-07 |  |  |  |  |
| *CACNG2* | 2.309842342 | 8.93E-07 | 3.37E-06 |  |  |  |  |
| *CECR2* | 3.25883916 | 3.80E-34 | 1.04E-32 |  |  |  |  |
| *CECR3* | -2.163650586 | 9.45E-08 | 4.03E-07 |  |  |  |  |
| *CR936633* | -2.209754992 | 0.000111071 | 0.000313654 |  |  |  |  |
| *DQ571461.1* | 2.658345282 | 4.22E-05 | 0.000126685 |  |  |  |  |
| *ENTHD1* | 2.208866535 | 7.35E-16 | 6.74E-15 |  |  |  |  |
| *FLJ46257* | 3.161533526 | 1.47E-21 | 1.98E-20 |  |  |  |  |
| *GAL3ST1* | -4.932917172 | 1.93E-118 | 6.90E-116 |  |  |  |  |
| *GGT2* | 3.088051969 | 2.40E-07 | 9.74E-07 |  |  |  |  |
| *GGT3P* | 3.083261056 | 1.77E-17 | 1.84E-16 |  |  |  |  |
| *GSC2* | 4.573950694 | 1.84E-17 | 1.91E-16 |  |  |  |  |
| *IL17REL* | -2.870181168 | 3.47E-23 | 5.22E-22 |  |  |  |  |
| *ISX* | -7.212125175 | 4.38E-54 | 2.68E-52 |  |  |  |  |
| *KIAA1644* | 2.356886575 | 2.97E-26 | 5.38E-25 |  |  |  |  |
| *KLHDC7B* | 3.080478745 | 3.05E-24 | 4.89E-23 |  |  |  |  |
| *LGALS2* | -3.962944433 | 3.83E-60 | 2.95E-58 |  |  |  |  |
| *LOC284865* | -3.050978568 | 1.39E-18 | 1.54E-17 |  |  |  |  |
| *LOC284912* | 2.307577953 | 3.58E-10 | 2.00E-09 |  |  |  |  |
| *MGAT3* | -2.388805442 | 1.14E-29 | 2.46E-28 |  |  |  |  |
| *MN1* | 2.163420573 | 4.33E-29 | 9.09E-28 |  |  |  |  |
| *MPPED1* | 5.422688587 | 1.78E-37 | 5.68E-36 |  |  |  |  |
| *P712P.3* | 2.221869475 | 0.006519546 | 0.013352158 |  |  |  |  |
| *PANX2* | 3.16318386 | 7.54E-35 | 2.14E-33 |  |  |  |  |
| *PHF21B* | 2.446813236 | 4.12E-15 | 3.57E-14 |  |  |  |  |
| *PNPLA5* | 2.943746454 | 3.25E-08 | 1.47E-07 |  |  |  |  |
| *POM121L4P* | 3.897487186 | 4.40E-18 | 4.75E-17 |  |  |  |  |
| *PVALB* | 2.284445583 | 9.33E-13 | 6.65E-12 |  |  |  |  |
| *RFPL1* | 2.418679191 | 3.34E-20 | 4.14E-19 |  |  |  |  |
| *SGSM1* | -2.323420227 | 8.53E-27 | 1.60E-25 |  |  |  |  |
| *SLC5A1* | -2.300029133 | 5.76E-20 | 7.03E-19 |  |  |  |  |
| *SOX10* | 2.572903856 | 7.20E-18 | 7.68E-17 |  |  |  |  |
| *TBX1* | 3.984788072 | 7.25E-76 | 9.47E-74 |  |  |  |  |
| *UPK3A* | -4.139535919 | 1.04E-41 | 4.03E-40 |  |  |  |  |
| *WNT7B* | 2.878034991 | 7.38E-33 | 1.88E-31 |  |  |  |  |
| *A4GNT* | -4.771087719 | 1.26E-37 | 4.05E-36 |  |  |  |  |
| *AADACL2* | 4.100286849 | 1.20E-27 | 2.35E-26 |  |  |  |  |
| *ABCC5* | 2.461859619 | 1.38E-51 | 7.76E-50 |  |  |  |  |
| *AK056252* | 2.932793615 | 3.56E-23 | 5.34E-22 |  |  |  |  |
| *AK091265* | -5.174753742 | 3.04E-83 | 5.10E-81 |  |  |  |  |
| *AK092143* | 2.926162788 | 3.25E-21 | 4.28E-20 |  |  |  |  |
| *AK092619* | 3.1238002 | 6.26E-26 | 1.11E-24 |  |  |  |  |
| *AK098763* | 2.342168943 | 8.13E-05 | 0.000234101 |  |  |  |  |
| *AK124973* | 3.26742436 | 5.10E-55 | 3.24E-53 |  |  |  |  |
| *AK128202* | 3.709877549 | 1.42E-25 | 2.48E-24 |  |  |  |  |
| *AK304483* | 3.278209798 | 3.43E-21 | 4.52E-20 |  |  |  |  |
| *ANKUB1* | 4.88366962 | 1.38E-31 | 3.31E-30 |  |  |  |  |
| *ARHGEF26-AS1* | 2.536836675 | 1.51E-17 | 1.57E-16 |  |  |  |  |
| *ARL14* | -3.571301017 | 1.31E-34 | 3.67E-33 |  |  |  |  |
| *ARPP21.1* | 2.308403738 | 2.41E-06 | 8.56E-06 |  |  |  |  |
| *ATP13A5* | 4.88608965 | 6.45E-41 | 2.42E-39 |  |  |  |  |
| *AX746877.1* | 2.769380455 | 4.81E-25 | 8.09E-24 |  |  |  |  |
| *AX746877.2* | 2.90499567 | 2.26E-31 | 5.34E-30 |  |  |  |  |
| *BC032918* | 3.16662359 | 0.001097995 | 0.002620181 |  |  |  |  |
| *BC036236* | 3.561631185 | 3.43E-22 | 4.85E-21 |  |  |  |  |
| *BC038725* | -2.140145407 | 7.27E-05 | 0.000210827 |  |  |  |  |
| *BC042737* | 2.050939786 | 4.82E-05 | 0.000143436 |  |  |  |  |
| *BCHE* | 2.951930876 | 5.04E-24 | 7.94E-23 |  |  |  |  |
| *C3orf32* | -2.643841476 | 1.22E-43 | 5.13E-42 |  |  |  |  |
| *C3orf43* | 2.898590882 | 2.21E-25 | 3.79E-24 |  |  |  |  |
| *C3orf72* | 3.153783115 | 4.36E-23 | 6.48E-22 |  |  |  |  |
| *C3orf74* | 2.788063683 | 2.40E-21 | 3.21E-20 |  |  |  |  |
| *C3orf79* | 2.912304974 | 6.05E-17 | 6.04E-16 |  |  |  |  |
| *CACNA1D* | -2.893755359 | 5.13E-39 | 1.76E-37 |  |  |  |  |
| *CACNA2D3* | 2.248012671 | 1.05E-29 | 2.29E-28 |  |  |  |  |
| *CADPS* | -4.545300314 | 7.40E-67 | 7.05E-65 |  |  |  |  |
| *CCK* | -3.377455331 | 4.67E-22 | 6.54E-21 |  |  |  |  |
| *CCR9* | -2.178195086 | 1.79E-19 | 2.12E-18 |  |  |  |  |
| *CCRL2* | -2.513043222 | 6.70E-63 | 5.76E-61 |  |  |  |  |
| *CEP19* | 2.008128855 | 4.06E-52 | 2.34E-50 |  |  |  |  |
| *CHDH* | -3.253980807 | 7.92E-71 | 8.60E-69 |  |  |  |  |
| *CHST13* | -3.192054088 | 1.53E-34 | 4.29E-33 |  |  |  |  |
| *CHST2* | 2.259901811 | 3.56E-28 | 7.15E-27 |  |  |  |  |
| *CIDEC* | -2.162889588 | 1.06E-39 | 3.78E-38 |  |  |  |  |
| *CLDN18* | -8.868644229 | 2.13E-184 | 2.22E-181 |  |  |  |  |
| *COL7A1* | 2.321861885 | 7.38E-37 | 2.29E-35 |  |  |  |  |
| *DQ571917* | 4.230078298 | 6.41E-23 | 9.46E-22 |  |  |  |  |
| *EPHA6* | 2.296466684 | 1.49E-12 | 1.04E-11 |  |  |  |  |
| *ESRG* | 7.446402398 | 8.69E-68 | 8.49E-66 |  |  |  |  |
| *EU250752* | 2.430760024 | 1.70E-05 | 5.40E-05 |  |  |  |  |
| *FAM3D* | -3.128776343 | 2.71E-22 | 3.85E-21 |  |  |  |  |
| *FETUB* | 4.076320936 | 2.25E-22 | 3.22E-21 |  |  |  |  |
| *FLJ22763* | -4.090668963 | 5.78E-15 | 4.96E-14 |  |  |  |  |
| *FLJ46066* | 3.858725032 | 2.54E-22 | 3.61E-21 |  |  |  |  |
| *FOXL2* | 4.0552598 | 2.49E-37 | 7.89E-36 |  |  |  |  |
| *GADL1* | 2.263597534 | 1.76E-07 | 7.25E-07 |  |  |  |  |
| *GAP43* | 2.190572184 | 2.88E-19 | 3.35E-18 |  |  |  |  |
| *GLYCTK* | -2.555888501 | 2.88E-109 | 8.80E-107 |  |  |  |  |
| *GP9* | -2.244808026 | 3.03E-08 | 1.38E-07 |  |  |  |  |
| *GPR128* | -6.330409945 | 6.97E-82 | 1.15E-79 |  |  |  |  |
| *GPR149* | 7.132044019 | 1.34E-41 | 5.14E-40 |  |  |  |  |
| *GPR156* | 2.959683427 | 3.78E-37 | 1.19E-35 |  |  |  |  |
| *GPR160* | -2.537873854 | 1.01E-49 | 5.46E-48 |  |  |  |  |
| *GPR27* | 2.423769705 | 2.24E-17 | 2.31E-16 |  |  |  |  |
| *GPR87* | 3.55263629 | 2.46E-30 | 5.48E-29 |  |  |  |  |
| *GUCA1C* | 3.215608947 | 2.09E-08 | 9.70E-08 |  |  |  |  |
| *HGD* | -5.881189772 | 7.68E-125 | 3.02E-122 |  |  |  |  |
| *HHLA2* | -6.555643192 | 5.55E-117 | 1.96E-114 |  |  |  |  |
| *HTR3D* | 2.046111173 | 1.76E-05 | 5.58E-05 |  |  |  |  |
| *IGSF11* | 3.22671133 | 1.34E-34 | 3.76E-33 |  |  |  |  |
| *IL17RB* | -3.183398135 | 2.96E-90 | 6.00E-88 |  |  |  |  |
| *IL20RB* | 3.021263709 | 1.00E-32 | 2.53E-31 |  |  |  |  |
| *IQCF2* | 2.754264193 | 0.000170123 | 0.000465228 |  |  |  |  |
| *ITIH1* | -2.820624771 | 5.27E-15 | 4.54E-14 |  |  |  |  |
| *JA611300* | 2.396847635 | 0.000256313 | 0.000679848 |  |  |  |  |
| *KBTBD12* | -3.957993045 | 3.69E-42 | 1.46E-40 |  |  |  |  |
| *KNG1* | -2.063019941 | 6.88E-12 | 4.53E-11 |  |  |  |  |
| *LEPREL1* | 2.284490594 | 9.46E-24 | 1.46E-22 |  |  |  |  |
| *LINC00606* | 3.769164187 | 6.10E-08 | 2.68E-07 |  |  |  |  |
| *LIPH* | -2.732677594 | 1.03E-37 | 3.33E-36 |  |  |  |  |
| *LOC201617* | -3.015701389 | 1.38E-26 | 2.55E-25 |  |  |  |  |
| *LOC285326* | 2.156344252 | 0.005020776 | 0.010518577 |  |  |  |  |
| *LOC285375* | 2.704490477 | 0.000352362 | 0.000912949 |  |  |  |  |
| *LOC344887* | 5.245341991 | 3.93E-75 | 5.03E-73 |  |  |  |  |
| *LOC401109* | 4.383868584 | 2.72E-62 | 2.28E-60 |  |  |  |  |
| *LRRC2* | -2.523586885 | 1.82E-25 | 3.14E-24 |  |  |  |  |
| *LRRC31* | -5.634068599 | 5.87E-65 | 5.34E-63 |  |  |  |  |
| *LRRIQ4* | -2.835669939 | 2.04E-30 | 4.57E-29 |  |  |  |  |
| *LSAMP-AS3* | 3.203934216 | 8.87E-05 | 0.000254147 |  |  |  |  |
| *LYZL4* | -2.126220601 | 9.90E-06 | 3.25E-05 |  |  |  |  |
| *MECOM* | -2.412712239 | 1.03E-42 | 4.17E-41 |  |  |  |  |
| *MED12L* | 2.158850099 | 4.80E-27 | 9.15E-26 |  |  |  |  |
| *MIR135A1* | -2.27705021 | 1.41E-35 | 4.14E-34 |  |  |  |  |
| *MIR4273.1* | -2.58601179 | 0.000143877 | 0.000398589 |  |  |  |  |
| *MIR4791* | -2.141846155 | 0.002558623 | 0.005685688 |  |  |  |  |
| *MIR944* | 4.858928939 | 1.17E-29 | 2.52E-28 |  |  |  |  |
| *MLF1* | 2.049609665 | 2.06E-19 | 2.42E-18 |  |  |  |  |
| *MORC1* | 2.073911618 | 4.55E-05 | 0.000136007 |  |  |  |  |
| *MUC13* | -8.723081566 | 3.56E-223 | 7.48E-220 |  |  |  |  |
| *MUC4* | -2.151107489 | 3.51E-11 | 2.16E-10 |  |  |  |  |
| *MYRIP* | -2.237998014 | 1.38E-20 | 1.75E-19 |  |  |  |  |
| *Mir_340.5* | -2.206317145 | 5.11E-06 | 1.75E-05 |  |  |  |  |
| *Mir_384.4* | 3.161191891 | 1.15E-06 | 4.27E-06 |  |  |  |  |
| *Mir_584.29* | -2.596770647 | 0.000666048 | 0.001647593 |  |  |  |  |
| *NR1I2* | -6.455602131 | 3.50E-110 | 1.09E-107 |  |  |  |  |
| *P2RY1* | 2.529278767 | 3.10E-26 | 5.61E-25 |  |  |  |  |
| *PAQR9* | 2.321556749 | 1.33E-06 | 4.93E-06 |  |  |  |  |
| *PHLDB2* | 2.348641806 | 7.32E-42 | 2.85E-40 |  |  |  |  |
| *PLA1A* | -2.333165992 | 9.88E-26 | 1.74E-24 |  |  |  |  |
| *PLCH1* | -2.463923962 | 3.48E-34 | 9.51E-33 |  |  |  |  |
| *PLS1* | -2.95665464 | 8.16E-80 | 1.24E-77 |  |  |  |  |
| *PLSCR5* | 2.28169559 | 1.16E-05 | 3.78E-05 |  |  |  |  |
| *PPARG* | -2.968778781 | 1.19E-49 | 6.41E-48 |  |  |  |  |
| *PPP2R3A* | 2.150783934 | 1.65E-52 | 9.52E-51 |  |  |  |  |
| *PTX3* | 2.296111667 | 8.71E-17 | 8.58E-16 |  |  |  |  |
| *PVRL3* | -2.085678453 | 1.08E-30 | 2.47E-29 |  |  |  |  |
| *RBP2* | -4.479109872 | 7.06E-46 | 3.25E-44 |  |  |  |  |
| *RETNLB* | -3.342651179 | 1.47E-19 | 1.74E-18 |  |  |  |  |
| *RN7SK.68* | 2.036412753 | 0.02383603 | 0.042848703 |  |  |  |  |
| *RN7SK.70* | 3.858763184 | 6.86E-08 | 2.99E-07 |  |  |  |  |
| *ROPN1* | 3.269570231 | 4.61E-13 | 3.37E-12 |  |  |  |  |
| *RTP1* | 3.547287089 | 3.67E-18 | 3.98E-17 |  |  |  |  |
| *RTP3* | 4.500176584 | 5.30E-21 | 6.91E-20 |  |  |  |  |
| *SI* | -9.841064649 | 3.36E-114 | 1.09E-111 |  |  |  |  |
| *SLC2A2* | -3.281392209 | 7.64E-17 | 7.58E-16 |  |  |  |  |
| *SLC38A3* | -2.02516232 | 4.70E-11 | 2.86E-10 |  |  |  |  |
| *SLC6A11* | 3.009626385 | 7.89E-14 | 6.17E-13 |  |  |  |  |
| *SLC6A20* | -6.007489437 | 1.00E-144 | 5.54E-142 |  |  |  |  |
| *SOX14* | -3.023529738 | 1.12E-08 | 5.34E-08 |  |  |  |  |
| *SOX2-OT* | 4.47108643 | 8.91E-57 | 6.01E-55 |  |  |  |  |
| *SOX2* | 3.189131433 | 1.26E-26 | 2.34E-25 |  |  |  |  |
| *SPSB4* | 3.714861634 | 1.08E-48 | 5.65E-47 |  |  |  |  |
| *SST* | -3.636014809 | 4.60E-15 | 3.98E-14 |  |  |  |  |
| *STXBP5L* | 2.025765169 | 4.77E-10 | 2.64E-09 |  |  |  |  |
| *SYNPR* | -5.010005019 | 1.34E-38 | 4.50E-37 |  |  |  |  |
| *TDGF1* | -5.798826451 | 4.07E-62 | 3.38E-60 |  |  |  |  |
| *TGM4* | 3.096023029 | 3.04E-44 | 1.31E-42 |  |  |  |  |
| *TM4SF19* | 3.112668295 | 2.63E-64 | 2.36E-62 |  |  |  |  |
| *TM4SF4* | -8.390475122 | 2.75E-128 | 1.20E-125 |  |  |  |  |
| *TMEM45A* | 2.891805611 | 8.86E-40 | 3.17E-38 |  |  |  |  |
| *TNNC1* | -4.85282578 | 1.76E-95 | 4.02E-93 |  |  |  |  |
| *TP63* | 5.475683208 | 2.64E-80 | 4.05E-78 |  |  |  |  |
| *U3.8* | 2.514278901 | 0.00981595 | 0.019327397 |  |  |  |  |
| *U6.102* | 2.632247398 | 0.00380152 | 0.008156709 |  |  |  |  |
| *UCN2* | 2.114092591 | 3.89E-23 | 5.81E-22 |  |  |  |  |
| *VEPH1* | -2.027489936 | 3.60E-19 | 4.16E-18 |  |  |  |  |
| *VILL* | -3.629537386 | 2.37E-101 | 6.26E-99 |  |  |  |  |
| *WNT7A* | 2.671458931 | 5.50E-12 | 3.65E-11 |  |  |  |  |
| *ZBED2* | 3.139806189 | 5.26E-36 | 1.57E-34 |  |  |  |  |
| *ZIC1* | 2.173514742 | 4.34E-06 | 1.50E-05 |  |  |  |  |
| *ZIC4* | 2.369444177 | 1.08E-06 | 4.04E-06 |  |  |  |  |
| *AB059369* | -4.031703851 | 7.04E-32 | 1.70E-30 |  |  |  |  |
| *ADH4* | -4.154953011 | 4.82E-44 | 2.06E-42 |  |  |  |  |
| *ADH6* | -3.722461815 | 2.05E-62 | 1.73E-60 |  |  |  |  |
| *ADH7* | 3.464308425 | 1.20E-14 | 1.01E-13 |  |  |  |  |
| *AGXT2L1* | -2.368106399 | 3.33E-08 | 1.51E-07 |  |  |  |  |
| *AK001394* | 2.022721857 | 1.48E-32 | 3.71E-31 |  |  |  |  |
| *AK026379* | -2.348238992 | 1.22E-38 | 4.09E-37 |  |  |  |  |
| *AK056196* | 2.388031624 | 1.31E-10 | 7.65E-10 |  |  |  |  |
| *AK091889* | 2.250719296 | 6.47E-06 | 2.18E-05 |  |  |  |  |
| *AK093205* | 2.023239285 | 0.001011312 | 0.002430191 |  |  |  |  |
| *AK094909* | -2.524249115 | 6.30E-27 | 1.19E-25 |  |  |  |  |
| *AK124272* | -2.221748526 | 9.26E-07 | 3.49E-06 |  |  |  |  |
| *ALB* | -2.171150904 | 2.08E-13 | 1.56E-12 |  |  |  |  |
| *AMTN* | 3.430582839 | 1.82E-14 | 1.50E-13 |  |  |  |  |
| *ANXA10* | -3.00820868 | 4.17E-15 | 3.61E-14 |  |  |  |  |
| *ARHGEF38* | -5.393786037 | 2.57E-132 | 1.24E-129 |  |  |  |  |
| *ATOH1* | -8.153265725 | 1.56E-86 | 2.92E-84 |  |  |  |  |
| *ATP8A1* | -2.131082129 | 1.91E-27 | 3.72E-26 |  |  |  |  |
| *AX748249* | -3.784102723 | 5.29E-44 | 2.25E-42 |  |  |  |  |
| *BC016361* | 2.797956087 | 1.18E-21 | 1.61E-20 |  |  |  |  |
| *BC025350* | 2.895833206 | 9.57E-05 | 0.000272611 |  |  |  |  |
| *BC031092* | 2.790667565 | 3.19E-16 | 3.01E-15 |  |  |  |  |
| *BC033991* | 2.134715539 | 0.005099868 | 0.010660434 |  |  |  |  |
| *BC034799* | 3.396935675 | 4.32E-05 | 0.000129526 |  |  |  |  |
| *BC038746* | 2.120570998 | 1.53E-13 | 1.17E-12 |  |  |  |  |
| *BC038750* | 2.140545553 | 0.016969955 | 0.031584392 |  |  |  |  |
| *BC040219* | 2.910894458 | 2.94E-10 | 1.66E-09 |  |  |  |  |
| *BC042433* | 2.089769705 | 0.00301163 | 0.006597899 |  |  |  |  |
| *BC042823* | -2.693329608 | 5.50E-26 | 9.82E-25 |  |  |  |  |
| *BC043280* | 2.066164717 | 0.001587983 | 0.00367173 |  |  |  |  |
| *BC070495* | 3.002390391 | 2.93E-18 | 3.19E-17 |  |  |  |  |
| *BC137484* | 2.020970278 | 3.18E-10 | 1.79E-09 |  |  |  |  |
| *C4orf17* | 5.015863825 | 2.61E-25 | 4.45E-24 |  |  |  |  |
| *C4orf19* | -3.428118593 | 3.94E-53 | 2.32E-51 |  |  |  |  |
| *CABS1* | 2.085097775 | 0.000762877 | 0.001870932 |  |  |  |  |
| *CHRNA9* | 3.524765978 | 8.69E-24 | 1.35E-22 |  |  |  |  |
| *CLDN24* | 2.879617055 | 3.37E-26 | 6.09E-25 |  |  |  |  |
| *CPLX1* | -2.003688627 | 1.16E-33 | 3.08E-32 |  |  |  |  |
| *CXCL3* | -3.507640561 | 4.67E-65 | 4.26E-63 |  |  |  |  |
| *CXCL5* | -2.766128101 | 1.99E-16 | 1.91E-15 |  |  |  |  |
| *DOK7* | -2.133042448 | 9.41E-24 | 1.46E-22 |  |  |  |  |
| *DQ266889* | 2.053020046 | 9.40E-07 | 3.54E-06 |  |  |  |  |
| *DQ590589.20* | -2.050482372 | 0.008944239 | 0.017748663 |  |  |  |  |
| *DRD5* | 2.747799023 | 3.59E-23 | 5.39E-22 |  |  |  |  |
| *DSPP* | 2.133548478 | 9.24E-13 | 6.59E-12 |  |  |  |  |
| *F11* | -3.851235325 | 4.37E-21 | 5.73E-20 |  |  |  |  |
| *FABP2* | -7.205387014 | 9.02E-82 | 1.47E-79 |  |  |  |  |
| *FGA* | -4.087864911 | 1.50E-19 | 1.78E-18 |  |  |  |  |
| *FGB* | -3.364785927 | 1.73E-11 | 1.10E-10 |  |  |  |  |
| *FGFBP1* | 2.464115127 | 2.06E-16 | 1.98E-15 |  |  |  |  |
| *FGFBP2* | 5.273414876 | 2.83E-49 | 1.51E-47 |  |  |  |  |
| *FLJ35424* | -3.320674225 | 1.01E-08 | 4.86E-08 |  |  |  |  |
| *FRG2.1* | 2.421853195 | 0.000366234 | 0.000946542 |  |  |  |  |
| *GABRA2* | -2.931157277 | 5.32E-16 | 4.93E-15 |  |  |  |  |
| *GALNTL6* | -2.462664462 | 6.38E-16 | 5.87E-15 |  |  |  |  |
| *GBA3* | -4.082055984 | 2.52E-31 | 5.92E-30 |  |  |  |  |
| *GC* | -6.875623192 | 5.49E-53 | 3.23E-51 |  |  |  |  |
| *GPM6A* | 2.77775868 | 1.75E-14 | 1.44E-13 |  |  |  |  |
| *GPRIN3* | -2.506301316 | 2.70E-39 | 9.42E-38 |  |  |  |  |
| *HAND2* | 2.882049383 | 3.67E-17 | 3.73E-16 |  |  |  |  |
| *HELT* | 2.758785086 | 8.92E-08 | 3.82E-07 |  |  |  |  |
| *HMX1* | 7.190439096 | 1.29E-51 | 7.28E-50 |  |  |  |  |
| *HSD17B11* | -2.012567414 | 2.36E-61 | 1.90E-59 |  |  |  |  |
| *HTN3* | 2.644913833 | 0.000106432 | 0.000301242 |  |  |  |  |
| *IGJ* | -2.365307966 | 2.38E-15 | 2.10E-14 |  |  |  |  |
| *KCNIP4-IT1* | 4.357485018 | 1.11E-22 | 1.61E-21 |  |  |  |  |
| *KIAA1211* | -2.328523956 | 2.02E-39 | 7.11E-38 |  |  |  |  |
| *LOC100505989* | -6.032134948 | 7.84E-60 | 5.92E-58 |  |  |  |  |
| *LOC285419* | -2.956435111 | 3.29E-72 | 3.87E-70 |  |  |  |  |
| *LOC285548* | 2.068239063 | 4.38E-08 | 1.96E-07 |  |  |  |  |
| *LOC401134* | 2.93815545 | 1.67E-06 | 6.09E-06 |  |  |  |  |
| *LOC644145* | -2.107010751 | 5.18E-05 | 0.00015345 |  |  |  |  |
| *LRAT* | 4.001681692 | 3.04E-29 | 6.42E-28 |  |  |  |  |
| *LRRC66* | -4.989776885 | 2.79E-125 | 1.11E-122 |  |  |  |  |
| *MGC45800* | 2.900386185 | 5.33E-20 | 6.52E-19 |  |  |  |  |
| *MGC4836* | 2.928305001 | 8.98E-19 | 1.01E-17 |  |  |  |  |
| *MIR1305* | 2.074203099 | 0.013588254 | 0.02591355 |  |  |  |  |
| *MIR4275* | 2.027165862 | 0.016936555 | 0.031535192 |  |  |  |  |
| *MIR4799* | 2.209879689 | 0.001292788 | 0.003041195 |  |  |  |  |
| *MIR548I2* | -3.965015053 | 2.07E-38 | 6.86E-37 |  |  |  |  |
| *MTNR1A* | -2.875722844 | 8.93E-23 | 1.30E-21 |  |  |  |  |
| *MTTP* | -5.379981729 | 2.45E-53 | 1.45E-51 |  |  |  |  |
| *Metazoa_SRP.78* | 2.008500554 | 0.00324561 | 0.007059267 |  |  |  |  |
| *NBLA00301* | 2.762295078 | 7.33E-14 | 5.75E-13 |  |  |  |  |
| *NEUROG2* | 2.587491693 | 4.32E-09 | 2.16E-08 |  |  |  |  |
| *NKX3-2* | 2.354866654 | 5.97E-17 | 5.96E-16 |  |  |  |  |
| *NR3C2* | -2.081856958 | 5.63E-21 | 7.34E-20 |  |  |  |  |
| *ODAM* | -3.813714879 | 2.68E-23 | 4.05E-22 |  |  |  |  |
| *ODZ3* | 2.582804733 | 4.03E-24 | 6.40E-23 |  |  |  |  |
| *PCDH10* | 2.190159165 | 5.88E-10 | 3.22E-09 |  |  |  |  |
| *PCNAP1* | 2.165689239 | 1.38E-05 | 4.45E-05 |  |  |  |  |
| *PF4* | -4.822112586 | 3.03E-60 | 2.34E-58 |  |  |  |  |
| *PHOX2B* | 3.769379636 | 8.47E-11 | 5.03E-10 |  |  |  |  |
| *PITX2* | 3.283995556 | 5.89E-49 | 3.09E-47 |  |  |  |  |
| *PLAC8* | -2.579977031 | 1.03E-24 | 1.71E-23 |  |  |  |  |
| *PPP2R2C* | 2.586593958 | 6.09E-17 | 6.07E-16 |  |  |  |  |
| *PROM1* | -4.367007463 | 2.24E-46 | 1.06E-44 |  |  |  |  |
| *PTPN13* | 2.127137434 | 4.37E-23 | 6.49E-22 |  |  |  |  |
| *QRFPR* | 4.546970978 | 5.08E-38 | 1.66E-36 |  |  |  |  |
| *RBM46* | 2.519809105 | 6.44E-11 | 3.87E-10 |  |  |  |  |
| *RN7SK.76* | 2.407719886 | 0.001905471 | 0.004337162 |  |  |  |  |
| *RN7SK.78* | 2.280330131 | 0.014686282 | 0.027777452 |  |  |  |  |
| *RN7SK.86* | 2.232186987 | 0.000814382 | 0.001987023 |  |  |  |  |
| *RN7SK.87* | -2.361617018 | 0.00064438 | 0.001597193 |  |  |  |  |
| *RNF175* | 2.035725734 | 5.39E-27 | 1.02E-25 |  |  |  |  |
| *S100P* | -2.553595822 | 3.16E-20 | 3.93E-19 |  |  |  |  |
| *SHROOM3* | -2.383619673 | 5.60E-46 | 2.60E-44 |  |  |  |  |
| *SLC10A4* | 2.077368091 | 1.42E-14 | 1.18E-13 |  |  |  |  |
| *SLC4A4* | -4.95716178 | 5.29E-87 | 1.01E-84 |  |  |  |  |
| *SLIT2-IT1* | 2.303874875 | 0.000128723 | 0.000359393 |  |  |  |  |
| *SPINK2* | 2.609388835 | 3.39E-18 | 3.68E-17 |  |  |  |  |
| *SPOCK3* | 4.589515685 | 7.90E-35 | 2.24E-33 |  |  |  |  |
| *STATH* | 2.266073963 | 1.70E-06 | 6.20E-06 |  |  |  |  |
| *STOX2* | 2.059521296 | 2.07E-34 | 5.73E-33 |  |  |  |  |
| *SULT1B1* | -4.325817586 | 2.08E-46 | 9.86E-45 |  |  |  |  |
| *TRIML1* | 2.625219553 | 3.58E-10 | 2.00E-09 |  |  |  |  |
| *U6.122* | -3.126459783 | 4.38E-11 | 2.68E-10 |  |  |  |  |
| *UCHL1* | 3.771548874 | 1.69E-33 | 4.45E-32 |  |  |  |  |
| *UGT2A3* | -7.994785356 | 2.04E-74 | 2.56E-72 |  |  |  |  |
| *UGT2B11* | -2.133154832 | 1.24E-05 | 4.02E-05 |  |  |  |  |
| *UGT2B15* | -6.340300799 | 2.74E-70 | 2.92E-68 |  |  |  |  |
| *UGT2B17* | -3.10340549 | 1.81E-13 | 1.37E-12 |  |  |  |  |
| *UGT2B7* | -5.033412102 | 5.69E-48 | 2.88E-46 |  |  |  |  |
| *AB074188* | -2.55838005 | 7.24E-18 | 7.71E-17 |  |  |  |  |
| *ADRB2* | 2.194761891 | 3.34E-21 | 4.41E-20 |  |  |  |  |
| *AF086294* | 3.480185045 | 2.43E-08 | 1.12E-07 |  |  |  |  |
| *AK123816* | 2.463565557 | 9.76E-10 | 5.22E-09 |  |  |  |  |
| *AK128486* | 2.164863494 | 7.43E-13 | 5.35E-12 |  |  |  |  |
| *ANKRD33B* | 2.213442733 | 4.41E-24 | 6.98E-23 |  |  |  |  |
| *ANKRD34B* | 2.143219695 | 3.63E-08 | 1.64E-07 |  |  |  |  |
| *ARSI* | 2.562008916 | 2.87E-32 | 7.12E-31 |  |  |  |  |
| *ATP10B* | -3.079639343 | 9.70E-36 | 2.86E-34 |  |  |  |  |
| *AX746723* | 2.607292751 | 1.87E-06 | 6.80E-06 |  |  |  |  |
| *AX746964* | -2.155713062 | 3.12E-09 | 1.59E-08 |  |  |  |  |
| *AX747345* | 2.793334907 | 5.25E-15 | 4.51E-14 |  |  |  |  |
| *BASP1* | 2.286118784 | 3.93E-34 | 1.07E-32 |  |  |  |  |
| *BC028204* | 2.76892333 | 1.68E-08 | 7.86E-08 |  |  |  |  |
| *BC032469* | -5.456365561 | 5.69E-46 | 2.63E-44 |  |  |  |  |
| *BC032795* | -3.421167536 | 2.04E-38 | 6.75E-37 |  |  |  |  |
| *BC034636* | 4.447490615 | 2.40E-44 | 1.03E-42 |  |  |  |  |
| *BC038535* | 4.135867683 | 3.88E-21 | 5.10E-20 |  |  |  |  |
| *BC127870* | -2.567988625 | 4.65E-32 | 1.14E-30 |  |  |  |  |
| *BTNL3* | -6.873372675 | 1.68E-87 | 3.28E-85 |  |  |  |  |
| *BTNL8* | -7.350535239 | 1.16E-172 | 9.52E-170 |  |  |  |  |
| *C5orf46* | 2.030307101 | 1.43E-10 | 8.34E-10 |  |  |  |  |
| *C5orf52* | -4.063294602 | 3.74E-30 | 8.27E-29 |  |  |  |  |
| *C6* | -3.866613795 | 4.12E-23 | 6.14E-22 |  |  |  |  |
| *CARTPT* | 3.583901151 | 4.59E-11 | 2.80E-10 |  |  |  |  |
| *CCL28* | -2.704070186 | 8.18E-36 | 2.43E-34 |  |  |  |  |
| *CCNI2* | -3.199928716 | 2.10E-97 | 4.91E-95 |  |  |  |  |
| *CDH18* | 2.061757802 | 5.86E-05 | 0.000172375 |  |  |  |  |
| *CDHR2* | -6.41615121 | 1.12E-192 | 1.60E-189 |  |  |  |  |
| *CDX1* | -5.392256934 | 3.59E-74 | 4.45E-72 |  |  |  |  |
| *CTXN3* | 3.105864576 | 1.11E-16 | 1.09E-15 |  |  |  |  |
| *CXCL14* | 2.362180178 | 7.86E-23 | 1.15E-21 |  |  |  |  |
| *CYSTM1* | -3.133773353 | 4.72E-103 | 1.31E-100 |  |  |  |  |
| *DQ576909* | -2.42957734 | 2.13E-08 | 9.87E-08 |  |  |  |  |
| *DRD1* | -3.3117532 | 5.12E-28 | 1.02E-26 |  |  |  |  |
| *ENC1* | -2.291258698 | 3.13E-59 | 2.32E-57 |  |  |  |  |
| *FAM134B* | -2.17251529 | 8.09E-33 | 2.06E-31 |  |  |  |  |
| *FAT2* | 4.060324664 | 5.29E-43 | 2.16E-41 |  |  |  |  |
| *FBN2* | 2.845816508 | 1.20E-30 | 2.72E-29 |  |  |  |  |
| *FBXL21* | 3.383033339 | 7.96E-10 | 4.29E-09 |  |  |  |  |
| *FGF18* | -2.176956387 | 1.98E-26 | 3.65E-25 |  |  |  |  |
| *FGFR4* | -3.513909338 | 1.75E-99 | 4.27E-97 |  |  |  |  |
| *FSTL4* | 3.842713684 | 1.37E-52 | 7.94E-51 |  |  |  |  |
| *FST* | 2.743314832 | 7.32E-31 | 1.69E-29 |  |  |  |  |
| *GABRP* | -2.032988631 | 7.60E-09 | 3.71E-08 |  |  |  |  |
| *GHR.1* | 2.238550014 | 2.53E-27 | 4.90E-26 |  |  |  |  |
| *GHR.2* | 2.574586429 | 1.26E-07 | 5.31E-07 |  |  |  |  |
| *HAVCR1* | -4.912510226 | 5.19E-46 | 2.42E-44 |  |  |  |  |
| *HSPB3* | 3.800975819 | 6.38E-33 | 1.64E-31 |  |  |  |  |
| *HTR4* | -2.19623531 | 8.15E-09 | 3.96E-08 |  |  |  |  |
| *IL31RA* | 3.501792241 | 2.81E-42 | 1.12E-40 |  |  |  |  |
| *IQGAP2* | -2.975102183 | 1.10E-63 | 9.78E-62 |  |  |  |  |
| *IRX1* | 3.820328313 | 5.68E-17 | 5.69E-16 |  |  |  |  |
| *IRX4* | 4.87468933 | 9.26E-45 | 4.06E-43 |  |  |  |  |
| *ISL1* | -2.151302505 | 2.11E-09 | 1.09E-08 |  |  |  |  |
| *JAKMIP2* | 2.152191275 | 6.66E-15 | 5.69E-14 |  |  |  |  |
| *JF824130* | 2.01940462 | 4.27E-09 | 2.14E-08 |  |  |  |  |
| *LOC100505841* | 2.545915347 | 3.95E-13 | 2.91E-12 |  |  |  |  |
| *LOC153469* | 2.242719224 | 2.55E-14 | 2.08E-13 |  |  |  |  |
| *LOC285577* | 2.583978545 | 6.58E-06 | 2.21E-05 |  |  |  |  |
| *LOC285696* | 2.377886372 | 1.99E-17 | 2.06E-16 |  |  |  |  |
| *LOC340074* | 2.799623967 | 1.32E-09 | 6.95E-09 |  |  |  |  |
| *LOC340094* | 2.06689768 | 0.010945758 | 0.021335471 |  |  |  |  |
| *LOC340107* | 2.784233777 | 0.000863982 | 0.002100119 |  |  |  |  |
| *LOC389332* | -4.827833219 | 2.24E-57 | 1.54E-55 |  |  |  |  |
| *LOC401177* | 2.143522556 | 5.88E-06 | 1.99E-05 |  |  |  |  |
| *LOC442132* | 2.866082138 | 4.59E-11 | 2.80E-10 |  |  |  |  |
| *LOC643201* | -2.728848823 | 1.87E-23 | 2.84E-22 |  |  |  |  |
| *MCC* | 2.432799865 | 6.67E-69 | 6.81E-67 |  |  |  |  |
| *MEGF10* | 2.057226327 | 5.97E-17 | 5.96E-16 |  |  |  |  |
| *MIR4277* | 2.062662552 | 0.000211065 | 0.000567983 |  |  |  |  |
| *MIR4803* | 2.350909533 | 0.000127503 | 0.000356389 |  |  |  |  |
| *Metazoa_SRP.85* | -3.665181038 | 9.21E-41 | 3.42E-39 |  |  |  |  |
| *Mir_544.26* | -2.549826468 | 4.94E-08 | 2.19E-07 |  |  |  |  |
| *NEUROG1* | 6.437881355 | 8.38E-27 | 1.57E-25 |  |  |  |  |
| *NIPAL4* | 3.088412236 | 8.28E-26 | 1.46E-24 |  |  |  |  |
| *NKX2-5* | 3.864115528 | 4.72E-17 | 4.77E-16 |  |  |  |  |
| *NMUR2* | -4.460950352 | 1.60E-30 | 3.58E-29 |  |  |  |  |
| *NPR3* | 3.065442793 | 7.11E-35 | 2.02E-33 |  |  |  |  |
| *OCLN.1* | -2.208660556 | 2.90E-41 | 1.10E-39 |  |  |  |  |
| *ODZ2* | 5.06849804 | 2.07E-69 | 2.15E-67 |  |  |  |  |
| *OR2V2* | 2.743668899 | 0.000111427 | 0.000314594 |  |  |  |  |
| *OR2Y1* | 2.531108452 | 0.004754433 | 0.009998368 |  |  |  |  |
| *PLEKHG4B* | 2.949012259 | 8.99E-27 | 1.68E-25 |  |  |  |  |
| *PPP2R2B* | 2.039508186 | 1.86E-17 | 1.92E-16 |  |  |  |  |
| *RAB3C* | -3.059132628 | 1.04E-26 | 1.94E-25 |  |  |  |  |
| *RN7SK.91* | 2.081680978 | 0.015814324 | 0.029681631 |  |  |  |  |
| *RN7SK.92* | 2.287296137 | 0.000452602 | 0.001152442 |  |  |  |  |
| *SLC12A2* | -2.487333203 | 1.25E-53 | 7.51E-52 |  |  |  |  |
| *SLC25A48* | 2.012894243 | 7.25E-13 | 5.22E-12 |  |  |  |  |
| *SLC34A1* | 2.359351038 | 7.13E-24 | 1.11E-22 |  |  |  |  |
| *SLC36A3* | 2.127969824 | 2.30E-13 | 1.73E-12 |  |  |  |  |
| *SLC6A18* | -3.063752457 | 1.23E-09 | 6.49E-09 |  |  |  |  |
| *SLC6A19* | -8.863258874 | 1.78E-106 | 5.32E-104 |  |  |  |  |
| *SLC6A7* | -3.264042419 | 1.10E-35 | 3.24E-34 |  |  |  |  |
| *SLC9A3* | -2.768771349 | 1.58E-24 | 2.57E-23 |  |  |  |  |
| *SNCB* | 3.412673442 | 1.44E-25 | 2.50E-24 |  |  |  |  |
| *SNORA40.4* | 4.908308082 | 1.11E-18 | 1.24E-17 |  |  |  |  |
| *SOWAHA* | -3.713615255 | 9.86E-70 | 1.04E-67 |  |  |  |  |
| *SPINK1* | -8.388321 | 5.10E-208 | 8.67E-205 |  |  |  |  |
| *SPINK6* | 4.234665821 | 3.93E-20 | 4.85E-19 |  |  |  |  |
| *TLX3* | 3.509754658 | 7.65E-09 | 3.73E-08 |  |  |  |  |
| *TRNA_Pseudo.77* | 2.349161584 | 0.007183378 | 0.014567306 |  |  |  |  |
| *TRPC7* | -2.449725802 | 3.10E-14 | 2.51E-13 |  |  |  |  |
| *TSSK1B* | 3.316325917 | 1.53E-56 | 1.03E-54 |  |  |  |  |
| *U6.137* | 2.295590384 | 0.009692616 | 0.019108099 |  |  |  |  |
| *UGT3A1* | 2.610165044 | 8.65E-07 | 3.27E-06 |  |  |  |  |
| *ZNF474* | 2.011433193 | 6.34E-24 | 9.92E-23 |  |  |  |  |
| *AF520419* | 2.822321078 | 2.16E-06 | 7.75E-06 |  |  |  |  |
| *AK024936* | -2.031581007 | 9.20E-22 | 1.26E-20 |  |  |  |  |
| *AK024998* | -3.929190979 | 1.06E-60 | 8.37E-59 |  |  |  |  |
| *AK056584* | 2.447799975 | 2.44E-21 | 3.25E-20 |  |  |  |  |
| *AK090788* | 2.524303218 | 5.86E-08 | 2.58E-07 |  |  |  |  |
| *AK123416* | -4.724431011 | 9.05E-33 | 2.30E-31 |  |  |  |  |
| *AK124950* | 2.086864867 | 5.69E-31 | 1.32E-29 |  |  |  |  |
| *AK126334* | 5.194073268 | 2.05E-32 | 5.11E-31 |  |  |  |  |
| *AL832096* | 2.182213135 | 0.002002983 | 0.004537445 |  |  |  |  |
| *BC040308* | 5.151763609 | 6.85E-54 | 4.16E-52 |  |  |  |  |
| *BC040898* | 2.282254601 | 1.11E-05 | 3.63E-05 |  |  |  |  |
| *BC047626* | -2.899460819 | 9.93E-30 | 2.16E-28 |  |  |  |  |
| *BC067243* | -2.034409255 | 0.01153028 | 0.022349738 |  |  |  |  |
| *BC070061* | 4.444196194 | 1.55E-48 | 8.05E-47 |  |  |  |  |
| *BEND6* | 2.293357482 | 1.83E-36 | 5.57E-35 |  |  |  |  |
| *BTN1A1* | -2.694918207 | 1.60E-22 | 2.31E-21 |  |  |  |  |
| *BVES-AS1* | 2.445022349 | 3.14E-24 | 5.03E-23 |  |  |  |  |
| *C6orf10* | 4.670713934 | 3.11E-34 | 8.51E-33 |  |  |  |  |
| *C6orf123* | -3.408847846 | 3.82E-67 | 3.68E-65 |  |  |  |  |
| *C6orf222* | -9.127621569 | 9.26E-285 | 6.29E-281 |  |  |  |  |
| *C6orf223* | -2.368963717 | 3.54E-18 | 3.84E-17 |  |  |  |  |
| *C6orf58* | -3.990645776 | 1.11E-23 | 1.71E-22 |  |  |  |  |
| *CAGE1* | 2.399180526 | 1.40E-34 | 3.92E-33 |  |  |  |  |
| *CD109* | 2.430133968 | 5.15E-40 | 1.85E-38 |  |  |  |  |
| *CDSN* | 3.803029887 | 6.71E-34 | 1.80E-32 |  |  |  |  |
| *CFB* | -2.790113951 | 2.64E-60 | 2.05E-58 |  |  |  |  |
| *CGA* | -2.843747054 | 1.81E-13 | 1.37E-12 |  |  |  |  |
| *CLDN20* | 2.150501673 | 3.60E-23 | 5.39E-22 |  |  |  |  |
| *CLIC5* | -3.621049113 | 3.24E-62 | 2.71E-60 |  |  |  |  |
| *COL9A1* | -2.960928641 | 5.54E-29 | 1.15E-27 |  |  |  |  |
| *DCDC2* | -5.303560713 | 1.94E-78 | 2.76E-76 |  |  |  |  |
| *DLK2* | 2.290707875 | 1.31E-42 | 5.29E-41 |  |  |  |  |
| *DPCR1* | -6.853297619 | 6.73E-62 | 5.56E-60 |  |  |  |  |
| *ENPP4* | -2.298586039 | 1.14E-39 | 4.05E-38 |  |  |  |  |
| *EPHA7* | 2.880484907 | 1.16E-17 | 1.22E-16 |  |  |  |  |
| *FAXC* | 2.087316085 | 5.38E-22 | 7.48E-21 |  |  |  |  |
| *FRMD1* | -2.487334863 | 1.92E-11 | 1.21E-10 |  |  |  |  |
| *FUT9* | -6.236248679 | 1.12E-52 | 6.50E-51 |  |  |  |  |
| *GJA1* | 2.924103084 | 8.99E-62 | 7.40E-60 |  |  |  |  |
| *GMDS* | -2.421674143 | 2.49E-75 | 3.20E-73 |  |  |  |  |
| *GSTA2* | -2.917566868 | 6.70E-12 | 4.42E-11 |  |  |  |  |
| *GSTA3* | 2.679208211 | 2.04E-12 | 1.41E-11 |  |  |  |  |
| *GSTA7P* | 2.816189937 | 6.21E-08 | 2.72E-07 |  |  |  |  |
| *HCRTR2* | 2.018930032 | 5.31E-08 | 2.35E-07 |  |  |  |  |
| *HDGFL1* | 5.150152657 | 2.63E-17 | 2.69E-16 |  |  |  |  |
| *HMGA1P7* | 2.456524351 | 9.65E-06 | 3.17E-05 |  |  |  |  |
| *IL17A* | -2.634011752 | 1.06E-13 | 8.22E-13 |  |  |  |  |
| *IL22RA2* | 2.297149685 | 7.37E-17 | 7.32E-16 |  |  |  |  |
| *IYD* | -6.722652107 | 5.49E-162 | 3.92E-159 |  |  |  |  |
| *KAAG1* | -4.177698575 | 7.12E-39 | 2.42E-37 |  |  |  |  |
| *KCNK16* | -2.673259586 | 5.92E-05 | 0.000174008 |  |  |  |  |
| *KCNK5* | -3.191765065 | 3.97E-73 | 4.82E-71 |  |  |  |  |
| *KCNQ5* | 4.242497822 | 8.19E-83 | 1.36E-80 |  |  |  |  |
| *KHDC1L* | 3.735549758 | 4.90E-30 | 1.08E-28 |  |  |  |  |
| *KIAA1244* | -2.650838969 | 2.94E-43 | 1.22E-41 |  |  |  |  |
| *KLHL32* | -2.253161237 | 3.59E-23 | 5.39E-22 |  |  |  |  |
| *LGSN* | -2.568585831 | 9.37E-09 | 4.51E-08 |  |  |  |  |
| *LINC00574* | -2.057535571 | 7.34E-24 | 1.15E-22 |  |  |  |  |
| *LOC100422737* | -4.430325188 | 2.35E-32 | 5.84E-31 |  |  |  |  |
| *LOC100507203* | -4.829239363 | 1.41E-46 | 6.78E-45 |  |  |  |  |
| *LOC154092* | 3.536644565 | 6.50E-48 | 3.28E-46 |  |  |  |  |
| *LOC285740* | -2.105892525 | 4.91E-36 | 1.47E-34 |  |  |  |  |
| *LOC285758* | -2.450650277 | 1.96E-28 | 4.00E-27 |  |  |  |  |
| *LY6G6C* | 2.62410426 | 1.85E-15 | 1.65E-14 |  |  |  |  |
| *MDGA1* | 2.403540785 | 5.10E-27 | 9.70E-26 |  |  |  |  |
| *MEP1A* | -7.461151306 | 5.22E-127 | 2.12E-124 |  |  |  |  |
| *Metazoa_SRP.91* | 2.751081391 | 3.06E-33 | 7.94E-32 |  |  |  |  |
| *Metazoa_SRP.92* | 2.026206046 | 0.028141649 | 0.049636153 |  |  |  |  |
| *NR2E1* | 2.251817706 | 7.20E-12 | 4.73E-11 |  |  |  |  |
| *OR11A1* | 3.042825647 | 7.03E-06 | 2.35E-05 |  |  |  |  |
| *PAQR8* | -2.75315328 | 3.99E-49 | 2.11E-47 |  |  |  |  |
| *PBOV1* | -2.448362662 | 2.23E-15 | 1.97E-14 |  |  |  |  |
| *PERP* | 2.045911605 | 3.98E-37 | 1.25E-35 |  |  |  |  |
| *PGC* | -9.643397193 | 1.40E-102 | 3.82E-100 |  |  |  |  |
| *PNLDC1* | 2.611187535 | 1.30E-13 | 1.00E-12 |  |  |  |  |
| *POPDC3* | 3.299461217 | 3.30E-25 | 5.59E-24 |  |  |  |  |
| *POU3F2* | 2.356588454 | 7.77E-10 | 4.19E-09 |  |  |  |  |
| *POU5F1.2* | -2.284556113 | 2.77E-18 | 3.02E-17 |  |  |  |  |
| *PPP1R14C* | 2.660847188 | 1.31E-29 | 2.82E-28 |  |  |  |  |
| *PRDM13* | 3.804013858 | 2.46E-18 | 2.69E-17 |  |  |  |  |
| *PSORS1C3* | -2.174375134 | 3.52E-17 | 3.58E-16 |  |  |  |  |
| *RFX6* | -4.560028599 | 3.21E-30 | 7.10E-29 |  |  |  |  |
| *RIPPLY2* | 2.999394516 | 2.30E-11 | 1.44E-10 |  |  |  |  |
| *RN7SK.98* | 2.281543624 | 6.73E-05 | 0.000196097 |  |  |  |  |
| *RN7SK.100* | -2.055722372 | 0.000326147 | 0.000850707 |  |  |  |  |
| *RNF217* | 2.999764666 | 4.13E-63 | 3.59E-61 |  |  |  |  |
| *SAMD5* | -2.367076009 | 3.88E-23 | 5.81E-22 |  |  |  |  |
| *SCGN* | -4.676998693 | 1.31E-22 | 1.89E-21 |  |  |  |  |
| *SCML4* | -2.019944976 | 1.02E-19 | 1.23E-18 |  |  |  |  |
| *SFTA2* | -3.263032635 | 1.64E-16 | 1.58E-15 |  |  |  |  |
| *SH3BGRL2* | -2.44440111 | 1.91E-38 | 6.34E-37 |  |  |  |  |
| *SIM1* | 2.745318611 | 8.79E-08 | 3.77E-07 |  |  |  |  |
| *SLC17A1* | -2.246627312 | 3.57E-06 | 1.24E-05 |  |  |  |  |
| *SLC17A4* | -8.292202297 | 8.80E-101 | 2.26E-98 |  |  |  |  |
| *SLC25A51P1* | 2.084485311 | 0.002966059 | 0.006511481 |  |  |  |  |
| *SLC26A8* | -2.301595466 | 9.98E-35 | 2.82E-33 |  |  |  |  |
| *SLC35D3* | -4.268985912 | 7.04E-41 | 2.63E-39 |  |  |  |  |
| *SLC44A4* | -6.354367114 | 2.42E-137 | 1.26E-134 |  |  |  |  |
| *SNORD45* | 2.303674223 | 0.010897567 | 0.021255262 |  |  |  |  |
| *SPDEF* | -3.191516484 | 1.75E-23 | 2.66E-22 |  |  |  |  |
| *STL* | 3.244980793 | 2.69E-71 | 3.00E-69 |  |  |  |  |
| *TAAR1* | -5.12924797 | 1.65E-24 | 2.69E-23 |  |  |  |  |
| *TAAR3* | -4.102336867 | 2.82E-31 | 6.61E-30 |  |  |  |  |
| *TAAR5* | 2.546143231 | 5.41E-05 | 0.000159987 |  |  |  |  |
| *TCP10L2* | -4.335617665 | 3.23E-22 | 4.57E-21 |  |  |  |  |
| *TCP10* | -3.616116044 | 2.21E-17 | 2.28E-16 |  |  |  |  |
| *TFAP2B* | 3.068925391 | 1.16E-09 | 6.15E-09 |  |  |  |  |
| *TFAP2D* | 2.10306696 | 0.001281104 | 0.003016595 |  |  |  |  |
| *TINAG* | -3.233284126 | 7.66E-17 | 7.58E-16 |  |  |  |  |
| *TRDN* | 2.518796416 | 1.75E-07 | 7.24E-07 |  |  |  |  |
| *TREML3P* | 2.213080041 | 2.60E-11 | 1.62E-10 |  |  |  |  |
| *TRIM10* | -4.533536311 | 2.37E-67 | 2.30E-65 |  |  |  |  |
| *TRIM15* | -5.577775947 | 1.65E-83 | 2.81E-81 |  |  |  |  |
| *TRIM31* | -5.875475108 | 1.03E-105 | 3.04E-103 |  |  |  |  |
| *TRIM40* | -5.4751964 | 5.32E-58 | 3.75E-56 |  |  |  |  |
| *TRNA_Ala.25* | 2.01016372 | 0.025749003 | 0.045901792 |  |  |  |  |
| *TRNA_Ala.33* | 2.356137348 | 0.008077662 | 0.016210222 |  |  |  |  |
| *TRNA_Arg.15* | 3.014859688 | 5.33E-05 | 0.000157788 |  |  |  |  |
| *TRNA_Ile.10* | 2.102054853 | 0.009119529 | 0.018075377 |  |  |  |  |
| *TRNA_Lys.30* | 2.216185015 | 0.010586731 | 0.020709955 |  |  |  |  |
| *TRNA_Met.16* | 2.611864353 | 0.000478701 | 0.001211967 |  |  |  |  |
| *TRNA_Phe.11* | 2.224110819 | 0.025709111 | 0.045836695 |  |  |  |  |
| *TSG1* | 2.648483914 | 1.10E-11 | 7.08E-11 |  |  |  |  |
| *TSPO2* | -2.861247492 | 2.63E-54 | 1.61E-52 |  |  |  |  |
| *TTLL2* | -2.829075139 | 4.62E-25 | 7.79E-24 |  |  |  |  |
| *T* | 2.343821713 | 1.61E-07 | 6.68E-07 |  |  |  |  |
| *UBD* | -2.529624945 | 8.13E-23 | 1.19E-21 |  |  |  |  |
| *UNC5CL* | -3.824157863 | 9.32E-130 | 4.15E-127 |  |  |  |  |
| *VN1R10P* | 2.302628636 | 0.00158379 | 0.0036633 |  |  |  |  |
| *VNN1* | -4.104104156 | 7.05E-47 | 3.44E-45 |  |  |  |  |
| *VNN2* | -2.810603765 | 3.34E-32 | 8.25E-31 |  |  |  |  |
| *Y_RNA.77* | 4.004734329 | 3.43E-19 | 3.96E-18 |  |  |  |  |
| *ABCA13* | 3.19554438 | 8.92E-35 | 2.52E-33 |  |  |  |  |
| *ABHD11-AS1* | -3.597033786 | 4.94E-72 | 5.77E-70 |  |  |  |  |
| *ABP1* | -4.596447619 | 5.00E-66 | 4.68E-64 |  |  |  |  |
| *ACHE* | -3.87760349 | 3.08E-58 | 2.20E-56 |  |  |  |  |
| *ADAP1* | -2.529183085 | 1.80E-87 | 3.50E-85 |  |  |  |  |
| *AGMO* | -2.579729199 | 1.87E-21 | 2.51E-20 |  |  |  |  |
| *AGR2* | -4.497894961 | 4.02E-56 | 2.66E-54 |  |  |  |  |
| *AGR3* | -7.564312474 | 2.37E-145 | 1.37E-142 |  |  |  |  |
| *AK094915* | 2.541488324 | 2.01E-06 | 7.24E-06 |  |  |  |  |
| *AK096803* | -6.315603633 | 2.02E-131 | 9.44E-129 |  |  |  |  |
| *AK097470* | 3.371181435 | 1.72E-19 | 2.03E-18 |  |  |  |  |
| *AKR1B10* | 2.124933508 | 3.81E-12 | 2.57E-11 |  |  |  |  |
| *AKR1B15* | 2.780480176 | 1.37E-19 | 1.63E-18 |  |  |  |  |
| *ANKRD7* | 2.786494605 | 4.50E-22 | 6.31E-21 |  |  |  |  |
| *ASB4* | -3.744763122 | 3.75E-23 | 5.61E-22 |  |  |  |  |
| *BC018166* | 4.867687074 | 4.32E-34 | 1.17E-32 |  |  |  |  |
| *BC034444* | -4.400350485 | 1.69E-22 | 2.43E-21 |  |  |  |  |
| *BC036261* | 2.877091166 | 0.000234368 | 0.0006253 |  |  |  |  |
| *BC038570* | -2.368207023 | 7.86E-31 | 1.80E-29 |  |  |  |  |
| *BC040865* | -2.584588143 | 3.65E-17 | 3.72E-16 |  |  |  |  |
| *BC087859* | 2.179981118 | 3.67E-20 | 4.54E-19 |  |  |  |  |
| *BC150495* | 2.660343126 | 0.0001744 | 0.000476 |  |  |  |  |
| *BX538274* | -6.646044429 | 1.04E-80 | 1.64E-78 |  |  |  |  |
| *C7orf10* | 2.32557936 | 1.13E-41 | 4.35E-40 |  |  |  |  |
| *C7orf65* | 2.068406274 | 1.05E-06 | 3.94E-06 |  |  |  |  |
| *C7orf66* | 3.366078397 | 7.46E-09 | 3.64E-08 |  |  |  |  |
| *CALN1* | 3.207921938 | 1.14E-13 | 8.82E-13 |  |  |  |  |
| *CFTR* | -6.317764903 | 3.63E-100 | 9.21E-98 |  |  |  |  |
| *CHN2* | -2.734384558 | 2.76E-65 | 2.54E-63 |  |  |  |  |
| *CLDN15* | -2.555012736 | 1.74E-39 | 6.13E-38 |  |  |  |  |
| *CLDN3* | -5.034922926 | 9.87E-87 | 1.86E-84 |  |  |  |  |
| *COBL* | -2.09524633 | 8.26E-23 | 1.21E-21 |  |  |  |  |
| *CPA2* | -2.936472031 | 1.05E-14 | 8.80E-14 |  |  |  |  |
| *CPA4* | 3.474326496 | 1.81E-25 | 3.11E-24 |  |  |  |  |
| *CYP2W1* | -4.710722344 | 4.42E-52 | 2.54E-50 |  |  |  |  |
| *CYP3A4* | -3.744449062 | 1.62E-35 | 4.74E-34 |  |  |  |  |
| *CYP3A7* | -3.913299296 | 6.47E-70 | 6.83E-68 |  |  |  |  |
| *DDC* | -4.995106374 | 1.14E-60 | 8.93E-59 |  |  |  |  |
| *DFNA5* | 2.24995779 | 9.44E-31 | 2.15E-29 |  |  |  |  |
| *DJ051769* | -3.725977745 | 1.15E-25 | 2.01E-24 |  |  |  |  |
| *DLX5* | 3.888747768 | 1.18E-55 | 7.69E-54 |  |  |  |  |
| *DLX6-AS1* | 2.203095532 | 6.03E-10 | 3.29E-09 |  |  |  |  |
| *DLX6* | 2.485878614 | 4.25E-11 | 2.60E-10 |  |  |  |  |
| *DPY19L2P1* | 3.160383506 | 3.35E-33 | 8.67E-32 |  |  |  |  |
| *DQ578920* | -3.615185659 | 4.62E-07 | 1.81E-06 |  |  |  |  |
| *DQ583756* | -4.663905359 | 1.04E-57 | 7.23E-56 |  |  |  |  |
| *EN2* | 2.099898437 | 2.92E-07 | 1.18E-06 |  |  |  |  |
| *EPO* | 4.145550959 | 1.79E-54 | 1.10E-52 |  |  |  |  |
| *EVX1* | -5.202752255 | 5.21E-44 | 2.22E-42 |  |  |  |  |
| *Evf1_1* | 2.114005086 | 3.04E-08 | 1.38E-07 |  |  |  |  |
| *FAM221A* | -2.387610632 | 1.66E-47 | 8.18E-46 |  |  |  |  |
| *FAM71F1* | 2.122243094 | 1.23E-11 | 7.93E-11 |  |  |  |  |
| *FLJ42280* | 2.571666462 | 1.05E-16 | 1.03E-15 |  |  |  |  |
| *FOXP2* | 2.176859519 | 7.78E-22 | 1.07E-20 |  |  |  |  |
| *FSCN1* | 2.992382807 | 5.81E-76 | 7.62E-74 |  |  |  |  |
| *GHRHR* | -4.104483628 | 5.97E-24 | 9.36E-23 |  |  |  |  |
| *GLI3* | 2.9261303 | 1.35E-75 | 1.75E-73 |  |  |  |  |
| *GNAT3* | 2.021117701 | 0.000414559 | 0.0010625 |  |  |  |  |
| *GPNMB* | 3.270033043 | 5.93E-55 | 3.72E-53 |  |  |  |  |
| *GU228584* | 2.332615731 | 3.83E-33 | 9.87E-32 |  |  |  |  |
| *HEPACAM2* | -5.570981483 | 1.08E-52 | 6.27E-51 |  |  |  |  |
| *HOTTIP* | -5.342885749 | 4.12E-81 | 6.58E-79 |  |  |  |  |
| *HOXA11-AS* | -2.768771385 | 2.15E-27 | 4.18E-26 |  |  |  |  |
| *HOXA13* | -3.426488234 | 1.43E-40 | 5.26E-39 |  |  |  |  |
| *HPVC1* | 2.490422803 | 0.001874437 | 0.0042728 |  |  |  |  |
| *HTR5A* | 2.068322304 | 0.002462807 | 0.0054889 |  |  |  |  |
| *HYALP1* | 3.275084768 | 3.85E-06 | 1.34E-05 |  |  |  |  |
| *ICA1* | -3.109893819 | 3.46E-68 | 3.41E-66 |  |  |  |  |
| *IGFBP1* | -4.861808275 | 5.09E-47 | 2.49E-45 |  |  |  |  |
| *INHBA-AS1* | 2.040819535 | 2.02E-17 | 2.09E-16 |  |  |  |  |
| *KIAA0087* | -2.089566893 | 1.29E-09 | 6.81E-09 |  |  |  |  |
| *KLF14* | 2.121032657 | 1.30E-09 | 6.89E-09 |  |  |  |  |
| *KLRG2* | 4.404300435 | 4.51E-55 | 2.87E-53 |  |  |  |  |
| *LAMB4* | 2.014556616 | 2.92E-15 | 2.56E-14 |  |  |  |  |
| *LEP* | 2.055541236 | 1.28E-10 | 7.49E-10 |  |  |  |  |
| *LHFPL3* | -3.556038389 | 1.49E-24 | 2.43E-23 |  |  |  |  |
| *LOC100124692* | -7.554460968 | 4.95E-128 | 2.10E-125 |  |  |  |  |
| *LOC100129427* | -7.584390213 | 1.58E-114 | 5.17E-112 |  |  |  |  |
| *LOC100131257* | -2.734370443 | 9.35E-23 | 1.36E-21 |  |  |  |  |
| *LOC100506895* | 3.206386237 | 1.32E-54 | 8.19E-53 |  |  |  |  |
| *LOC285889* | -2.141869233 | 6.00E-09 | 2.96E-08 |  |  |  |  |
| *LOC401296* | 2.619281066 | 2.77E-09 | 1.41E-08 |  |  |  |  |
| *LOC723809* | -3.801802809 | 4.26E-29 | 8.95E-28 |  |  |  |  |
| *LOC93432* | -7.133496315 | 2.04E-93 | 4.44E-91 |  |  |  |  |
| *LRRC17* | 2.199491189 | 1.41E-21 | 1.91E-20 |  |  |  |  |
| *LRRC4* | 2.916909629 | 4.01E-29 | 8.44E-28 |  |  |  |  |
| *MACC1* | -2.564677846 | 7.03E-50 | 3.83E-48 |  |  |  |  |
| *MGAM* | -2.602637255 | 5.22E-32 | 1.27E-30 |  |  |  |  |
| *MIR3147* | -2.169752594 | 0.001972862 | 0.0044763 |  |  |  |  |
| *MIR3666* | 3.16728645 | 5.29E-07 | 2.06E-06 |  |  |  |  |
| *MIR490* | 2.642089308 | 7.29E-05 | 0.0002114 |  |  |  |  |
| *MIR595* | -4.416037872 | 3.44E-15 | 3.00E-14 |  |  |  |  |
| *MLXIPL* | -2.958335488 | 2.05E-34 | 5.66E-33 |  |  |  |  |
| *MNX1* | -2.338300334 | 3.40E-23 | 5.11E-22 |  |  |  |  |
| *MOGAT3* | -7.073055258 | 1.67E-104 | 4.88E-102 |  |  |  |  |
| *MOXD2P* | -3.824151303 | 9.31E-19 | 1.05E-17 |  |  |  |  |
| *MUC12* | -6.357826867 | 4.58E-102 | 1.23E-99 |  |  |  |  |
| *MUC17* | -10.43355161 | 7.94E-173 | 6.74E-170 |  |  |  |  |
| *MUC3A* | -6.435160299 | 2.81E-141 | 1.49E-138 |  |  |  |  |
| *Metazoa_SRP.96* | 2.200722166 | 0.002820487 | 0.0062162 |  |  |  |  |
| *Metazoa_SRP.97* | 2.20983214 | 0.024774319 | 0.0443418 |  |  |  |  |
| *Mir_340.10* | -2.434064807 | 1.20E-09 | 6.34E-09 |  |  |  |  |
| *NOBOX* | 2.854316377 | 4.26E-07 | 1.67E-06 |  |  |  |  |
| *NPC1L1* | -5.039996367 | 2.71E-83 | 4.58E-81 |  |  |  |  |
| *NPSR1* | -2.055410864 | 4.77E-08 | 2.12E-07 |  |  |  |  |
| *PAX4* | -5.998576506 | 1.77E-33 | 4.65E-32 |  |  |  |  |
| *PDK4* | -2.51894078 | 7.61E-24 | 1.19E-22 |  |  |  |  |
| *PPP1R9A* | -2.471068187 | 1.40E-17 | 1.47E-16 |  |  |  |  |
| *PRR15* | -3.075330528 | 1.07E-49 | 5.77E-48 |  |  |  |  |
| *PRRT4* | 2.355516692 | 1.06E-21 | 1.45E-20 |  |  |  |  |
| *PRSS1* | -5.597271502 | 3.18E-39 | 1.10E-37 |  |  |  |  |
| *PRSS3P2* | -6.291861998 | 3.92E-39 | 1.36E-37 |  |  |  |  |
| *PTPRN2* | -3.674532059 | 1.91E-68 | 1.91E-66 |  |  |  |  |
| *PTPRZ1* | 3.602208458 | 2.10E-42 | 8.40E-41 |  |  |  |  |
| *RAB19* | -2.402462254 | 1.87E-37 | 5.94E-36 |  |  |  |  |
| *RBSG3* | 2.790351821 | 0.000909649 | 0.0022020 |  |  |  |  |
| *RPL13AP17* | 2.360055216 | 8.45E-06 | 2.80E-05 |  |  |  |  |
| *SHH* | -2.296632864 | 1.28E-12 | 8.99E-12 |  |  |  |  |
| *SLC26A3* | -7.434997106 | 8.00E-79 | 1.17E-76 |  |  |  |  |
| *SMO* | 2.427697316 | 2.25E-39 | 7.90E-38 |  |  |  |  |
| *SNORA25.4* | 2.63153605 | 0.001722926 | 0.0039565 |  |  |  |  |
| *SNORD93* | 2.091215459 | 1.49E-25 | 2.59E-24 |  |  |  |  |
| *SOSTDC1* | 2.425506315 | 2.67E-14 | 2.17E-13 |  |  |  |  |
| *STEAP1B* | 2.819156981 | 5.78E-26 | 1.03E-24 |  |  |  |  |
| *TBX20* | 2.42789008 | 6.13E-08 | 2.69E-07 |  |  |  |  |
| *TMEM139* | -4.013092799 | 7.18E-66 | 6.71E-64 |  |  |  |  |
| *TMEM176A* | -2.443946107 | 1.37E-46 | 6.57E-45 |  |  |  |  |
| *TMEM229A* | -4.984050382 | 3.77E-40 | 1.37E-38 |  |  |  |  |
| *TRNA_Cys.16* | 2.606490807 | 1.34E-05 | 4.31E-05 |  |  |  |  |
| *TSPAN12* | -2.492052497 | 7.75E-59 | 5.60E-57 |  |  |  |  |
| *U6.151* | 2.821466413 | 0.001175439 | 0.0027872 |  |  |  |  |
| *VSTM2A* | -2.375515026 | 3.72E-06 | 1.30E-05 |  |  |  |  |
| *VWC2* | 2.36476246 | 3.36E-13 | 2.49E-12 |  |  |  |  |
| *ZAN* | 4.792368527 | 8.27E-58 | 5.79E-56 |  |  |  |  |
| *ADAM28* | -2.284740456 | 1.94E-28 | 3.95E-27 |  |  |  |  |
| *ADAM7* | 3.427928919 | 2.74E-13 | 2.05E-12 |  |  |  |  |
| *ADCY8* | 4.198620448 | 8.07E-18 | 8.57E-17 |  |  |  |  |
| *AK001351* | 3.95595774 | 5.24E-42 | 2.05E-40 |  |  |  |  |
| *AK024242* | 2.428070534 | 2.03E-19 | 2.39E-18 |  |  |  |  |
| *AK057332* | -2.287505289 | 2.43E-11 | 1.51E-10 |  |  |  |  |
| *AK307207* | -3.613815798 | 4.92E-37 | 1.54E-35 |  |  |  |  |
| *AK308605* | 4.128455553 | 2.05E-31 | 4.85E-30 |  |  |  |  |
| *ANXA13* | -6.737005347 | 6.97E-130 | 3.16E-127 |  |  |  |  |
| *AX747124.1* | 4.086217255 | 6.54E-08 | 2.86E-07 |  |  |  |  |
| *AX747124.2* | 2.865051028 | 2.35E-05 | 7.33E-05 |  |  |  |  |
| *AX747544* | -2.149720105 | 5.68E-13 | 4.13E-12 |  |  |  |  |
| *AX748380* | -2.781382562 | 1.24E-19 | 1.48E-18 |  |  |  |  |
| *BAALC* | 4.117724811 | 2.91E-55 | 1.87E-53 |  |  |  |  |
| *BAI1* | 3.8981903 | 5.20E-50 | 2.84E-48 |  |  |  |  |
| *BC017578* | -3.332112107 | 6.62E-30 | 1.45E-28 |  |  |  |  |
| *BC037250* | 2.106613687 | 0.006034817 | 0.0124443 |  |  |  |  |
| *BC038546* | 3.771990306 | 1.15E-25 | 2.01E-24 |  |  |  |  |
| *BC042029* | 2.686415909 | 0.000222383 | 0.0005960 |  |  |  |  |
| *BC045738* | 2.176211463 | 4.64E-07 | 1.82E-06 |  |  |  |  |
| *BC048982* | 2.792789438 | 3.87E-15 | 3.36E-14 |  |  |  |  |
| *BC052578* | 2.795952864 | 3.67E-18 | 3.98E-17 |  |  |  |  |
| *C8orf31* | -2.29991068 | 2.13E-32 | 5.32E-31 |  |  |  |  |
| *C8orf42* | 2.169021402 | 4.88E-28 | 9.77E-27 |  |  |  |  |
| *C8orf49* | -4.430192463 | 2.45E-36 | 7.43E-35 |  |  |  |  |
| *C8orf56* | 3.44200882 | 5.31E-30 | 1.16E-28 |  |  |  |  |
| *CA13* | -2.256282893 | 1.25E-51 | 7.10E-50 |  |  |  |  |
| *CA1* | -6.438485649 | 7.40E-72 | 8.52E-70 |  |  |  |  |
| *CA3* | -2.445063225 | 5.92E-32 | 1.44E-30 |  |  |  |  |
| *CA8* | -2.881147485 | 4.27E-23 | 6.35E-22 |  |  |  |  |
| *CALB1* | 3.130406325 | 1.34E-14 | 1.12E-13 |  |  |  |  |
| *CCDC26* | 2.172508365 | 1.58E-07 | 6.56E-07 |  |  |  |  |
| *CDH17* | -7.709246841 | 4.61E-209 | 8.35E-206 |  |  |  |  |
| *CLDN23* | -3.168419106 | 9.44E-83 | 1.56E-80 |  |  |  |  |
| *DEFA5* | -2.830536312 | 0.003098471 | 0.0067707 |  |  |  |  |
| *DEFA6* | -6.913874225 | 2.84E-29 | 6.01E-28 |  |  |  |  |
| *DEPTOR* | -2.485424954 | 3.84E-53 | 2.27E-51 |  |  |  |  |
| *DQ580489* | 3.044102047 | 2.51E-15 | 2.21E-14 |  |  |  |  |
| *DQ589437* | 2.497101135 | 0.0003477 | 0.0009015 |  |  |  |  |
| *DUSP4* | -2.793860996 | 2.88E-45 | 1.29E-43 |  |  |  |  |
| *EFCAB1* | 4.831658586 | 1.09E-96 | 2.53E-94 |  |  |  |  |
| *FABP4* | 4.363110886 | 2.04E-39 | 7.16E-38 |  |  |  |  |
| *FABP5* | 2.904020489 | 3.17E-34 | 8.66E-33 |  |  |  |  |
| *FAM83A* | 2.217373721 | 2.16E-13 | 1.63E-12 |  |  |  |  |
| *FER1L6-AS1* | -2.833162402 | 2.63E-11 | 1.64E-10 |  |  |  |  |
| *FER1L6* | -2.751790062 | 2.57E-17 | 2.64E-16 |  |  |  |  |
| *FGF20* | -4.396083349 | 5.83E-23 | 8.62E-22 |  |  |  |  |
| *FGL1* | -2.644432376 | 3.16E-11 | 1.95E-10 |  |  |  |  |
| *GATA4* | -4.579211599 | 1.66E-44 | 7.17E-43 |  |  |  |  |
| *GDF6* | 2.047667118 | 7.30E-12 | 4.79E-11 |  |  |  |  |
| *GPT* | -2.745990495 | 1.20E-35 | 3.51E-34 |  |  |  |  |
| *GSDMC* | 3.227012628 | 1.18E-29 | 2.54E-28 |  |  |  |  |
| *HEY1* | 2.249725431 | 1.15E-30 | 2.62E-29 |  |  |  |  |
| *HHLA1* | 2.687329893 | 4.48E-05 | 0.0001339 |  |  |  |  |
| *HNF4G* | -4.60014043 | 5.88E-91 | 1.23E-88 |  |  |  |  |
| *KBTBD11* | -3.072106824 | 5.36E-59 | 3.92E-57 |  |  |  |  |
| *KCNB2* | 2.037113107 | 7.25E-09 | 3.54E-08 |  |  |  |  |
| *LGI3* | 4.112021879 | 6.10E-31 | 1.41E-29 |  |  |  |  |
| *LOC100130155* | 2.332098572 | 0.022680619 | 0.0410023 |  |  |  |  |
| *LOC100131726* | 2.688502086 | 2.20E-19 | 2.58E-18 |  |  |  |  |
| *LOC100505659* | -4.345723974 | 3.73E-15 | 3.24E-14 |  |  |  |  |
| *LOC100505718* | 2.659768983 | 6.71E-15 | 5.73E-14 |  |  |  |  |
| *LOC157381* | 3.22949988 | 3.26E-27 | 6.25E-26 |  |  |  |  |
| *LOC286083* | 2.025821654 | 7.90E-05 | 0.0002279 |  |  |  |  |
| *LOC286094* | 2.175208249 | 0.001918496 | 0.0043646 |  |  |  |  |
| *LOC286135* | 2.741345607 | 3.61E-05 | 0.0001095 |  |  |  |  |
| *LOC731779* | -2.183660207 | 4.64E-09 | 2.32E-08 |  |  |  |  |
| *LRP12* | 2.507265997 | 6.99E-59 | 5.08E-57 |  |  |  |  |
| *LY6D* | 2.633916609 | 7.07E-12 | 4.65E-11 |  |  |  |  |
| *MTSS1* | 2.34420982 | 4.66E-42 | 1.83E-40 |  |  |  |  |
| *Metazoa_SRP.103* | 3.266003649 | 6.14E-08 | 2.69E-07 |  |  |  |  |
| *Mir_548.68* | 2.713824437 | 5.57E-10 | 3.06E-09 |  |  |  |  |
| *NAT2* | -4.703379201 | 9.44E-45 | 4.14E-43 |  |  |  |  |
| *NDRG1* | 2.204039361 | 1.35E-33 | 3.57E-32 |  |  |  |  |
| *NEFL* | 6.079234932 | 3.76E-74 | 4.64E-72 |  |  |  |  |
| *NEFM* | 4.427798459 | 6.42E-40 | 2.31E-38 |  |  |  |  |
| *NKX2-6* | 3.547087487 | 1.09E-06 | 4.07E-06 |  |  |  |  |
| *NKX6-3* | -2.701771564 | 2.50E-05 | 7.75E-05 |  |  |  |  |
| *NPBWR1* | 5.428719464 | 3.27E-47 | 1.61E-45 |  |  |  |  |
| *NRG1* | 2.547107688 | 3.56E-26 | 6.43E-25 |  |  |  |  |
| *OC90* | 2.545717364 | 9.86E-06 | 3.24E-05 |  |  |  |  |
| *PMP2* | 2.086411346 | 1.63E-13 | 1.24E-12 |  |  |  |  |
| *PNMA2* | -2.744631415 | 3.81E-30 | 8.40E-29 |  |  |  |  |
| *PRSS55* | -2.036946746 | 1.10E-07 | 4.66E-07 |  |  |  |  |
| *PSCA* | -2.416626286 | 2.52E-13 | 1.88E-12 |  |  |  |  |
| *PSKH2* | 3.574576935 | 1.05E-06 | 3.93E-06 |  |  |  |  |
| *RGS20* | 2.998117028 | 5.78E-34 | 1.56E-32 |  |  |  |  |
| *RIMS2* | 2.327492113 | 1.42E-12 | 9.96E-12 |  |  |  |  |
| *RN7SK.112* | 2.047196162 | 0.010828831 | 0.0211409 |  |  |  |  |
| *SFRP1* | 2.721264906 | 1.25E-15 | 1.13E-14 |  |  |  |  |
| *SFTPC* | 2.217464511 | 2.03E-08 | 9.42E-08 |  |  |  |  |
| *SGCZ* | 2.447740176 | 7.16E-07 | 2.74E-06 |  |  |  |  |
| *SLC10A5* | -2.532007525 | 1.47E-42 | 5.90E-41 |  |  |  |  |
| *SLC18A1* | -4.80862119 | 1.35E-37 | 4.32E-36 |  |  |  |  |
| *SNAI2* | 3.219789433 | 3.08E-96 | 7.09E-94 |  |  |  |  |
| *SNORD112.17* | -2.22756818 | 7.94E-07 | 3.02E-06 |  |  |  |  |
| *SPAG11B.1* | 2.894185675 | 0.000183561 | 0.0004989 |  |  |  |  |
| *STAR* | 2.669793003 | 1.42E-43 | 5.95E-42 |  |  |  |  |
| *STMN4* | 2.344020783 | 1.38E-13 | 1.06E-12 |  |  |  |  |
| *SYBU* | -2.004559302 | 2.25E-22 | 3.21E-21 |  |  |  |  |
| *TNFRSF11B* | -3.024250983 | 1.66E-36 | 5.06E-35 |  |  |  |  |
| *TRPA1* | -2.056919073 | 2.27E-23 | 3.44E-22 |  |  |  |  |
| *TTPA* | -3.042230953 | 4.02E-25 | 6.79E-24 |  |  |  |  |
| *TUSC3* | 2.532141908 | 1.60E-44 | 6.95E-43 |  |  |  |  |
| *U3.12* | 2.49661906 | 1.24E-14 | 1.03E-13 |  |  |  |  |
| *U6.162* | 2.370112721 | 0.006519781 | 0.0133522 |  |  |  |  |
| *XKR5* | 2.621884847 | 3.15E-25 | 5.35E-24 |  |  |  |  |
| *XKR9* | -2.568383749 | 1.15E-36 | 3.53E-35 |  |  |  |  |
| *ZMAT4* | 3.834753754 | 8.59E-17 | 8.47E-16 |  |  |  |  |
| *ZNF705A.2* | 2.316869689 | 0.004849927 | 0.0101771 |  |  |  |  |
| *AK057188* | -2.041891915 | 1.58E-39 | 5.59E-38 |  |  |  |  |
| *AK093363* | 2.052388126 | 0.000160678 | 0.0004414 |  |  |  |  |
| *AK094342* | 2.753318848 | 1.92E-16 | 1.85E-15 |  |  |  |  |
| *AK128673* | 2.016930282 | 6.38E-19 | 7.24E-18 |  |  |  |  |
| *AK131516* | 3.127007687 | 4.03E-07 | 1.59E-06 |  |  |  |  |
| *AL390170* | 2.12632933 | 7.11E-10 | 3.85E-09 |  |  |  |  |
| *ALDOB* | -7.665822111 | 1.16E-174 | 1.08E-171 |  |  |  |  |
| *AMBP* | -4.086399969 | 6.91E-55 | 4.32E-53 |  |  |  |  |
| *AQP7* | -2.658097117 | 8.67E-29 | 1.80E-27 |  |  |  |  |
| *AX747119.1* | 2.002585027 | 0.000200653 | 0.0005417 |  |  |  |  |
| *AY343902* | -3.168842816 | 9.82E-06 | 3.23E-05 |  |  |  |  |
| *BAAT* | -4.596424779 | 5.86E-60 | 4.47E-58 |  |  |  |  |
| *BC016143.9* | 2.005598298 | 4.04E-14 | 3.24E-13 |  |  |  |  |
| *BC017988* | 2.269118074 | 2.66E-10 | 1.51E-09 |  |  |  |  |
| *BC035187* | 3.077939749 | 3.42E-09 | 1.73E-08 |  |  |  |  |
| *BC037833* | -3.063872981 | 1.53E-35 | 4.47E-34 |  |  |  |  |
| *BC039180* | 2.504825639 | 5.10E-21 | 6.67E-20 |  |  |  |  |
| *BC065763* | -3.216677898 | 5.00E-14 | 3.98E-13 |  |  |  |  |
| *BICD2* | 2.198947619 | 4.23E-68 | 4.17E-66 |  |  |  |  |
| *BX538226* | 2.86567599 | 1.07E-17 | 1.13E-16 |  |  |  |  |
| *C9orf135* | -2.949443511 | 1.45E-08 | 6.85E-08 |  |  |  |  |
| *C9orf152* | -4.730343535 | 8.49E-73 | 1.01E-70 |  |  |  |  |
| *C9orf170* | 2.384735092 | 8.43E-15 | 7.13E-14 |  |  |  |  |
| *CACNA1B* | 3.199688768 | 1.66E-23 | 2.54E-22 |  |  |  |  |
| *CNTNAP3B.2* | 2.188436884 | 3.33E-22 | 4.71E-21 |  |  |  |  |
| *CNTNAP3* | 2.875521562 | 3.26E-39 | 1.13E-37 |  |  |  |  |
| *CYLC2* | 2.211000071 | 0.00020658 | 0.0005567 |  |  |  |  |
| *DEC1* | 3.082666561 | 4.00E-24 | 6.35E-23 |  |  |  |  |
| *DIRAS2* | 3.402985178 | 8.12E-18 | 8.62E-17 |  |  |  |  |
| *DMRT1* | 2.997797464 | 4.32E-17 | 4.37E-16 |  |  |  |  |
| *DMRT2* | 6.30883919 | 4.74E-79 | 7.00E-77 |  |  |  |  |
| *DMRT3* | 3.991539407 | 6.89E-36 | 2.05E-34 |  |  |  |  |
| *DQ574810.2* | 2.793998146 | 2.71E-06 | 9.59E-06 |  |  |  |  |
| *DQ580140.4* | 2.665951181 | 6.99E-05 | 0.0002032 |  |  |  |  |
| *DQ585850.2* | 3.097860977 | 4.09E-07 | 1.61E-06 |  |  |  |  |
| *DQ587539.18* | 2.32373224 | 5.44E-06 | 1.85E-05 |  |  |  |  |
| *DQ587955* | 2.327775016 | 0.000153026 | 0.0004220 |  |  |  |  |
| *DQ594696.2* | 2.707755401 | 3.24E-05 | 9.88E-05 |  |  |  |  |
| *DQ597117.3* | 3.226371435 | 3.03E-07 | 1.22E-06 |  |  |  |  |
| *ELAVL2* | 3.39557751 | 2.29E-28 | 4.66E-27 |  |  |  |  |
| *ENTPD8* | -4.578308685 | 1.54E-89 | 3.11E-87 |  |  |  |  |
| *FAM205A* | 2.204375095 | 7.81E-14 | 6.11E-13 |  |  |  |  |
| *FAM75D3* | 2.221574647 | 0.011348871 | 0.022035839 |  |  |  |  |
| *FBP1* | -2.269820283 | 1.31E-29 | 2.81E-28 |  |  |  |  |
| *FBP2* | -2.047109136 | 2.47E-19 | 2.88E-18 |  |  |  |  |
| *FOXB2* | 3.222466044 | 9.37E-06 | 3.09E-05 |  |  |  |  |
| *FOXE1* | 4.414459096 | 7.70E-31 | 1.77E-29 |  |  |  |  |
| *FRRS1L* | 2.063288816 | 1.46E-08 | 6.88E-08 |  |  |  |  |
| *GCNT1* | -3.127094098 | 3.71E-63 | 3.25E-61 |  |  |  |  |
| *GDA* | -2.129069939 | 3.14E-12 | 2.14E-11 |  |  |  |  |
| *GFI1B* | -3.004792619 | 1.04E-22 | 1.51E-21 |  |  |  |  |
| *GLT6D1* | 2.436606194 | 0.000314895 | 0.00082278 |  |  |  |  |
| *GNA14* | -2.035241462 | 1.37E-29 | 2.95E-28 |  |  |  |  |
| *GOLM1* | -2.537773561 | 2.83E-72 | 3.34E-70 |  |  |  |  |
| *GPR144* | 2.170988377 | 1.12E-15 | 1.02E-14 |  |  |  |  |
| *IFNA10* | 2.138955633 | 0.016366925 | 0.030606725 |  |  |  |  |
| *IFNA13* | 3.05184607 | 0.001021468 | 0.002452425 |  |  |  |  |
| *KIAA1045* | 3.581763701 | 3.00E-42 | 1.19E-40 |  |  |  |  |
| *KIF12* | -3.882970314 | 6.86E-69 | 6.96E-67 |  |  |  |  |
| *LCN12* | -2.112861829 | 2.01E-20 | 2.52E-19 |  |  |  |  |
| *LCN15* | -3.623940388 | 4.75E-12 | 3.18E-11 |  |  |  |  |
| *LCN2* | -2.821683032 | 1.03E-26 | 1.93E-25 |  |  |  |  |
| *LCN9* | 2.126668481 | 0.01345506 | 0.025675763 |  |  |  |  |
| *LHX2* | 4.547886576 | 1.11E-84 | 1.97E-82 |  |  |  |  |
| *LINGO2* | 3.319210417 | 2.20E-22 | 3.14E-21 |  |  |  |  |
| *LOC100289019* | -2.263100923 | 2.53E-63 | 2.22E-61 |  |  |  |  |
| *LOC286238* | 3.780501888 | 8.87E-11 | 5.27E-10 |  |  |  |  |
| *LOC286359* | -2.307531987 | 1.12E-05 | 3.65E-05 |  |  |  |  |
| *LOC440173* | 2.109035582 | 1.33E-12 | 9.34E-12 |  |  |  |  |
| *LRRC19* | -2.724147363 | 1.16E-34 | 3.28E-33 |  |  |  |  |
| *LRRC26* | -2.830091263 | 1.12E-21 | 1.53E-20 |  |  |  |  |
| *MIR101-2* | 2.203069678 | 0.002140944 | 0.004828232 |  |  |  |  |
| *MIR126* | -2.094322329 | 1.47E-06 | 5.42E-06 |  |  |  |  |
| *MIR31* | 2.115428828 | 0.00039619 | 0.001018538 |  |  |  |  |
| *MIR3621* | -2.624419118 | 3.41E-10 | 1.92E-09 |  |  |  |  |
| *NTRK2* | 4.425351763 | 2.12E-49 | 1.14E-47 |  |  |  |  |
| *OBP2A* | 2.266385224 | 1.85E-13 | 1.40E-12 |  |  |  |  |
| *OLFM1* | 2.649370217 | 9.67E-26 | 1.70E-24 |  |  |  |  |
| *OR13C2* | 2.199830606 | 0.017481735 | 0.032441342 |  |  |  |  |
| *ORM1* | -5.986418302 | 2.73E-61 | 2.20E-59 |  |  |  |  |
| *PIP5K1B* | -4.52542409 | 4.35E-113 | 1.39E-110 |  |  |  |  |
| *PRKACG* | -2.492800373 | 3.15E-14 | 2.55E-13 |  |  |  |  |
| *PRSS3* | -2.868560984 | 3.26E-27 | 6.25E-26 |  |  |  |  |
| *PTPRD* | 2.551856322 | 8.18E-22 | 1.13E-20 |  |  |  |  |
| *RASEF* | -2.11790235 | 5.28E-30 | 1.16E-28 |  |  |  |  |
| *RN7SK.122* | 2.502652577 | 0.001311766 | 0.003080965 |  |  |  |  |
| *RNF183* | -2.554842346 | 2.55E-26 | 4.64E-25 |  |  |  |  |
| *SLC1A1* | -2.388630412 | 4.27E-37 | 1.34E-35 |  |  |  |  |
| *SPINK4* | -8.691900722 | 5.75E-145 | 3.25E-142 |  |  |  |  |
| *TMEM8C* | -2.516159464 | 0.000461007 | 0.001171536 |  |  |  |  |
| *TNC* | 3.585792704 | 2.27E-64 | 2.05E-62 |  |  |  |  |
| *TNFSF15* | -2.121665369 | 1.72E-46 | 8.20E-45 |  |  |  |  |
| *TOR4A* | -2.062055946 | 8.15E-28 | 1.61E-26 |  |  |  |  |
| *TXNDC8* | 2.204603688 | 1.57E-10 | 9.12E-10 |  |  |  |  |
| *AK091688.2* | 2.123536935 | 0.000182703 | 0.000496679 |  |  |  |  |
| *HYDIN.2* | 2.089662709 | 7.59E-18 | 8.07E-17 |  |  |  |  |
| *TRNA_Pseudo.97* | 2.671537354 | 2.91E-05 | 8.94E-05 |  |  |  |  |
| *MGC39584* | 2.3972838 | 0.000119654 | 0.000335936 |  |  |  |  |
| *ACE2* | -2.314841891 | 1.98E-18 | 2.18E-17 |  |  |  |  |
| *AFF2* | 2.922591231 | 1.10E-29 | 2.38E-28 |  |  |  |  |
| *AK123758* | -3.915747703 | 4.78E-32 | 1.17E-30 |  |  |  |  |
| *AKAP4* | -4.887233414 | 7.70E-25 | 1.28E-23 |  |  |  |  |
| *AMELX* | -2.870068052 | 5.52E-18 | 5.90E-17 |  |  |  |  |
| *ARHGAP36* | 2.306052678 | 1.45E-07 | 6.04E-07 |  |  |  |  |
| *ARSE* | -4.686293804 | 1.03E-116 | 3.59E-114 |  |  |  |  |
| *ARX* | -3.196918443 | 3.78E-21 | 4.97E-20 |  |  |  |  |
| *CDX4* | 3.923900781 | 1.65E-07 | 6.84E-07 |  |  |  |  |
| *CLCN4* | -2.270093294 | 2.47E-25 | 4.23E-24 |  |  |  |  |
| *CLDN2* | -7.02345034 | 3.00E-149 | 1.81E-146 |  |  |  |  |
| *CNGA2* | 2.211628138 | 0.01087693 | 0.02122263 |  |  |  |  |
| *COL4A5* | 2.741896527 | 1.03E-61 | 8.46E-60 |  |  |  |  |
| *COL4A6* | 3.524148448 | 1.56E-57 | 1.09E-55 |  |  |  |  |
| *CTAG1A.2* | 3.163185685 | 0.002457552 | 0.00547767 |  |  |  |  |
| *CXorf28* | 2.293148141 | 2.40E-16 | 2.30E-15 |  |  |  |  |
| *CXorf61* | -2.542930013 | 1.36E-07 | 5.68E-07 |  |  |  |  |
| *DCAF12L2* | 3.144412967 | 3.55E-20 | 4.40E-19 |  |  |  |  |
| *DQ580770.3* | 2.567908707 | 0.001947776 | 0.004425711 |  |  |  |  |
| *DQ595787.1* | 2.493287854 | 0.001333751 | 0.003128091 |  |  |  |  |
| *DQ595787.2* | 2.371308092 | 0.001182698 | 0.002802522 |  |  |  |  |
| *DQ595787.4* | 2.52926848 | 0.000425496 | 0.001088728 |  |  |  |  |
| *DQ595787.10* | 2.281431257 | 0.001585349 | 0.003666262 |  |  |  |  |
| *DQ597811.3* | 2.832971668 | 9.41E-05 | 0.00026821 |  |  |  |  |
| *DUSP9* | 2.715611937 | 1.46E-24 | 2.40E-23 |  |  |  |  |
| *FAM9C* | -2.113989977 | 1.82E-07 | 7.50E-07 |  |  |  |  |
| *FTHL17* | 3.141497175 | 0.000146275 | 0.000404737 |  |  |  |  |
| *GABRE* | 2.46185126 | 7.49E-23 | 1.10E-21 |  |  |  |  |
| *GABRQ* | 3.126633215 | 9.00E-26 | 1.58E-24 |  |  |  |  |
| *GJB1* | -7.683426038 | 1.43E-149 | 8.80E-147 |  |  |  |  |
| *GLOD5* | -3.317064945 | 3.62E-46 | 1.69E-44 |  |  |  |  |
| *GPR101* | 2.249630468 | 0.005876735 | 0.012152499 |  |  |  |  |
| *GPR50* | 5.229649537 | 1.73E-34 | 4.82E-33 |  |  |  |  |
| *H2BFM* | 2.302483742 | 3.82E-07 | 1.51E-06 |  |  |  |  |
| *HEPH* | -2.476480602 | 1.59E-38 | 5.33E-37 |  |  |  |  |
| *HS6ST2* | 3.442691255 | 3.35E-35 | 9.67E-34 |  |  |  |  |
| *IRS4* | 4.216129162 | 1.42E-17 | 1.48E-16 |  |  |  |  |
| *KIAA1210* | 2.563389491 | 1.42E-14 | 1.18E-13 |  |  |  |  |
| *KLHL4* | 2.508107205 | 1.33E-21 | 1.81E-20 |  |  |  |  |
| *LHFPL1* | 3.171087826 | 1.73E-20 | 2.19E-19 |  |  |  |  |
| *LINC00269* | 2.034194914 | 1.56E-05 | 4.99E-05 |  |  |  |  |
| *LONRF3* | -2.701608326 | 1.49E-45 | 6.70E-44 |  |  |  |  |
| *LUZP4* | 2.193509508 | 3.02E-06 | 1.06E-05 |  |  |  |  |
| *MAGEA11* | 3.096215398 | 1.99E-09 | 1.03E-08 |  |  |  |  |
| *MAGEB2* | 2.347549173 | 0.000338148 | 0.000878887 |  |  |  |  |
| *MAGEE1* | 2.062440134 | 7.78E-31 | 1.79E-29 |  |  |  |  |
| *MAP7D2* | 2.306179257 | 4.58E-13 | 3.35E-12 |  |  |  |  |
| *MUM1L1* | 2.430219437 | 4.98E-11 | 3.02E-10 |  |  |  |  |
| *Mir_548.81* | 2.232409745 | 0.001852607 | 0.004228171 |  |  |  |  |
| *NAP1L2* | 2.826315376 | 1.86E-34 | 5.18E-33 |  |  |  |  |
| *NOX1* | -4.588953769 | 9.24E-67 | 8.71E-65 |  |  |  |  |
| *NR0B1* | 6.102972217 | 1.51E-25 | 2.61E-24 |  |  |  |  |
| *NUDT10* | 3.107316166 | 1.48E-31 | 3.54E-30 |  |  |  |  |
| *NUDT11* | 4.195946796 | 1.32E-84 | 2.33E-82 |  |  |  |  |
| *NXF3* | -4.144444856 | 1.64E-42 | 6.58E-41 |  |  |  |  |
| *OTC* | -6.620326692 | 5.31E-55 | 3.36E-53 |  |  |  |  |
| *PCDH11X.1* | 2.002589299 | 3.32E-06 | 1.16E-05 |  |  |  |  |
| *PCDH19* | 3.229771216 | 4.44E-29 | 9.28E-28 |  |  |  |  |
| *PCYT1B* | 2.931355815 | 1.91E-27 | 3.72E-26 |  |  |  |  |
| *PNCK* | 3.60101509 | 9.99E-50 | 5.43E-48 |  |  |  |  |
| *POU3F4* | 2.096941927 | 0.000311981 | 0.000815872 |  |  |  |  |
| *REPS2* | -2.234729341 | 4.66E-42 | 1.83E-40 |  |  |  |  |
| *RGN* | -2.643009003 | 1.02E-27 | 2.02E-26 |  |  |  |  |
| *RNF128* | -2.08167425 | 2.19E-21 | 2.93E-20 |  |  |  |  |
| *RP1-177G6.2* | 2.772822367 | 1.09E-10 | 6.40E-10 |  |  |  |  |
| *RPS6KA6* | 2.012612219 | 1.05E-11 | 6.82E-11 |  |  |  |  |
| *SMEK3P* | 2.4464753 | 0.000223249 | 0.000597987 |  |  |  |  |
| *SOX3* | 2.466337591 | 0.004142923 | 0.008825112 |  |  |  |  |
| *SPANXC* | 4.046982882 | 2.52E-10 | 1.43E-09 |  |  |  |  |
| *SPANXE* | 3.51417752 | 2.81E-08 | 1.28E-07 |  |  |  |  |
| *SRD5A1P1* | 2.510863856 | 6.20E-10 | 3.38E-09 |  |  |  |  |
| *SSX4B* | -3.181085601 | 6.60E-05 | 0.000192764 |  |  |  |  |
| *SSX4* | -2.739362418 | 0.000336206 | 0.000874426 |  |  |  |  |
| *TBX22* | 2.596988327 | 0.000138466 | 0.000384971 |  |  |  |  |
| *TEX13B* | 2.514536933 | 1.42E-17 | 1.48E-16 |  |  |  |  |
| *TFDP3* | 2.917088656 | 5.63E-07 | 2.19E-06 |  |  |  |  |
| *TRNA_Ile.19* | 2.176036153 | 5.10E-06 | 1.74E-05 |  |  |  |  |
| *TRNA_Val.32* | -2.193734888 | 0.000162773 | 0.000446565 |  |  |  |  |
| *VSIG1* | -5.529578103 | 9.85E-77 | 1.35E-74 |  |  |  |  |
| *XAGE3* | -2.37560483 | 4.96E-06 | 1.70E-05 |  |  |  |  |
| *XK* | -2.394910874 | 8.17E-31 | 1.87E-29 |  |  |  |  |
| *XPNPEP2* | -3.388186721 | 1.21E-25 | 2.11E-24 |  |  |  |  |
| *ZC3H12B* | -2.022711018 | 2.00E-39 | 7.05E-38 |  |  |  |  |
| *ZIC3* | 4.431170553 | 7.98E-14 | 6.25E-13 |  |  |  |  |
| *AMELY* | 2.180375364 | 0.001263533 | 0.002977286 |  |  |  |  |
| *BC041884* | 3.298936673 | 4.21E-07 | 1.66E-06 |  |  |  |  |
| *DAZ1* | -5.879368493 | 1.23E-12 | 8.62E-12 |  |  |  |  |
| *DAZ2* | -5.78714973 | 3.31E-11 | 2.04E-10 |  |  |  |  |
| *DAZ4* | -4.544337685 | 1.05E-07 | 4.45E-07 |  |  |  |  |
| *RBMY1A3P* | 2.268849698 | 0.019747143 | 0.036202046 |  |  |  |  |
| *RBMY2EP* | -2.180822263 | 0.015155501 | 0.028565523 |  |  |  |  |
| *TGIF2LY* | 2.279150067 | 0.002728566 | 0.006033719 |  |  |  |  |
| *TSPY1* | 2.140765093 | 0.025388038 | 0.045332683 |  |  |  |  |
| *TTTY11* | 2.2595088 | 0.02053525 | 0.037478038 |  |  |  |  |
| *TTTY13* | -2.363438123 | 0.00114602 | 0.002723539 |  |  |  |  |
| *TTTY16* | 3.333296757 | 2.89E-07 | 1.16E-06 |  |  |  |  |
| **SNCA vs. EA** |  |  |  |  |  |  |  |
| **mRNAs** | **log2 Fold Change** | **p value** | **p adj** | **microRNAs** | **log2 Fold Change** | **p value** | **p adj** |
| *AK055631* | -2.211235314 | 2.15E-06 | 1.21E-05 | *hsa-mir-133a-2* | 2.209434464 | 6.32E-16 | 2.28E-14 |
| *ANGPTL1* | 2.402455644 | 1.29E-23 | 1.56E-21 | *hsa-mir-133b* | 2.214220783 | 4.20E-19 | 3.68E-17 |
| *APOA2* | 2.006838515 | 2.32E-13 | 6.05E-12 | *hsa-mir-205* | -2.644826264 | 4.74E-11 | 6.46E-10 |
| *ASTN1* | 2.267742135 | 2.54E-16 | 1.15E-14 | *hsa-mir-374c* | 2.909396163 | 1.51E-08 | 1.38E-07 |
| *ATP1A2* | 2.27866778 | 1.92E-17 | 1.06E-15 | *hsa-mir-483* | 2.380068632 | 2.09E-21 | 3.20E-19 |
| *BNIPL* | -3.363643815 | 1.81E-70 | 1.68E-67 | *hsa-mir-490* | 3.535281599 | 4.80E-29 | 2.94E-26 |
| *C1orf177* | -2.090806151 | 1.30E-30 | 2.44E-28 | *hsa-mir-944* | -2.565014479 | 5.26E-25 | 1.61E-22 |
| *CASQ1* | 2.059014773 | 9.81E-18 | 5.58E-16 |  |  |  |  |
| *CHIA* | 4.635356108 | 7.57E-26 | 1.15E-23 |  |  |  |  |
| *CLCA2* | -6.625130152 | 7.85E-106 | 1.57E-102 |  |  |  |  |
| *CLCA3P* | -2.181685741 | 3.20E-22 | 3.34E-20 |  |  |  |  |
| *CLCA4* | -6.624501915 | 1.63E-118 | 6.06E-115 |  |  |  |  |
| *CRCT1* | -6.162258712 | 7.76E-56 | 3.96E-53 |  |  |  |  |
| *CRNN* | -7.178867248 | 1.02E-56 | 5.67E-54 |  |  |  |  |
| *FLG* | -2.28015965 | 5.85E-24 | 7.40E-22 |  |  |  |  |
| *GBP6* | -5.474597757 | 1.62E-101 | 2.81E-98 |  |  |  |  |
| *GRHL3* | -2.48323397 | 2.12E-33 | 4.57E-31 |  |  |  |  |
| *GRIK3* | 2.486979776 | 6.82E-23 | 7.49E-21 |  |  |  |  |
| *HMGB4* | 2.70435082 | 2.39E-08 | 2.10E-07 |  |  |  |  |
| *HSPB7* | 3.088829279 | 9.48E-35 | 2.33E-32 |  |  |  |  |
| *IL19* | -2.022225091 | 2.27E-11 | 3.89E-10 |  |  |  |  |
| *IVL* | -6.60682851 | 3.19E-56 | 1.73E-53 |  |  |  |  |
| *KPRP* | -3.473282887 | 7.82E-15 | 2.77E-13 |  |  |  |  |
| *LCE3A* | -2.398170217 | 2.57E-06 | 1.41E-05 |  |  |  |  |
| *LCE3D* | -4.279808683 | 3.05E-23 | 3.51E-21 |  |  |  |  |
| *LCE3E* | -3.859097352 | 8.19E-17 | 4.04E-15 |  |  |  |  |
| *LHX8* | -2.540991445 | 2.92E-12 | 6.10E-11 |  |  |  |  |
| *LMOD1* | 2.390840042 | 1.93E-24 | 2.53E-22 |  |  |  |  |
| *LRRC7* | 3.127543223 | 6.92E-26 | 1.06E-23 |  |  |  |  |
| *MAB21L3* | -2.339615517 | 3.23E-22 | 3.36E-20 |  |  |  |  |
| *MIR205HG* | -3.695215629 | 1.78E-17 | 9.83E-16 |  |  |  |  |
| *OR10K2* | 2.193456643 | 3.69E-06 | 1.96E-05 |  |  |  |  |
| *OR2L13* | 2.027838675 | 2.08E-09 | 2.34E-08 |  |  |  |  |
| *PADI1* | -3.382926581 | 1.26E-30 | 2.38E-28 |  |  |  |  |
| *PGLYRP3* | -3.597437515 | 2.15E-29 | 3.77E-27 |  |  |  |  |
| *PKP1* | -3.947158778 | 4.25E-40 | 1.30E-37 |  |  |  |  |
| *PLD5* | 2.245000638 | 6.83E-12 | 1.32E-10 |  |  |  |  |
| *S100A12* | -2.165486903 | 1.19E-19 | 9.31E-18 |  |  |  |  |
| *S100A2* | -3.442550581 | 6.14E-36 | 1.61E-33 |  |  |  |  |
| *S100A7A* | -3.964537413 | 2.45E-21 | 2.30E-19 |  |  |  |  |
| *S100A7* | -4.046184476 | 2.46E-25 | 3.52E-23 |  |  |  |  |
| *S100A8* | -5.010924521 | 1.31E-89 | 1.89E-86 |  |  |  |  |
| *S100A9* | -4.164585173 | 2.98E-72 | 2.98E-69 |  |  |  |  |
| *SPRR1A* | -5.206545845 | 1.57E-45 | 5.68E-43 |  |  |  |  |
| *SPRR1B* | -4.574771315 | 5.51E-35 | 1.38E-32 |  |  |  |  |
| *SPRR2A* | -6.659028716 | 2.34E-63 | 1.56E-60 |  |  |  |  |
| *SPRR2B* | -5.937925362 | 3.50E-41 | 1.10E-38 |  |  |  |  |
| *SPRR2C* | -5.844937658 | 2.74E-34 | 6.47E-32 |  |  |  |  |
| *SPRR2D* | -6.067238836 | 5.55E-56 | 2.89E-53 |  |  |  |  |
| *SPRR2E* | -6.348044285 | 1.05E-50 | 4.54E-48 |  |  |  |  |
| *SPRR2F* | -4.783730627 | 2.01E-36 | 5.35E-34 |  |  |  |  |
| *SPRR2G* | -2.938525034 | 8.59E-13 | 1.99E-11 |  |  |  |  |
| *SPRR3* | -5.998405886 | 1.81E-59 | 1.05E-56 |  |  |  |  |
| *SPRR4* | -2.11624915 | 1.09E-05 | 5.18E-05 |  |  |  |  |
| *TRNA_Pseudo.19* | 2.337525599 | 3.70E-07 | 2.50E-06 |  |  |  |  |
| *TRNA_Val.4* | 2.145925375 | 3.52E-06 | 1.88E-05 |  |  |  |  |
| *ZNF683* | 2.112811672 | 1.17E-24 | 1.56E-22 |  |  |  |  |
| *AK128534* | -2.409491597 | 1.74E-18 | 1.12E-16 |  |  |  |  |
| *ANXA8L1.1* | -2.116937359 | 4.88E-06 | 2.52E-05 |  |  |  |  |
| *AX747977* | 2.327197838 | 3.25E-23 | 3.71E-21 |  |  |  |  |
| *BC051760* | -2.891495016 | 5.22E-20 | 4.31E-18 |  |  |  |  |
| *C10orf99* | -2.165811408 | 1.49E-07 | 1.10E-06 |  |  |  |  |
| *CALML3* | -5.919396398 | 8.27E-60 | 4.89E-57 |  |  |  |  |
| *CALML5* | -2.678070269 | 1.16E-10 | 1.70E-09 |  |  |  |  |
| *CTNNA3* | 2.158146394 | 7.49E-17 | 3.73E-15 |  |  |  |  |
| *FAM25A* | -2.826497564 | 7.89E-17 | 3.91E-15 |  |  |  |  |
| *KCNMA1* | 2.658931304 | 2.48E-30 | 4.55E-28 |  |  |  |  |
| *LDB3* | 2.572338269 | 3.91E-27 | 6.24E-25 |  |  |  |  |
| *LIPK* | -3.215807788 | 4.98E-19 | 3.55E-17 |  |  |  |  |
| *MKX* | 2.400973173 | 5.66E-33 | 1.18E-30 |  |  |  |  |
| *NKX6-2* | 2.098099103 | 5.58E-08 | 4.50E-07 |  |  |  |  |
| *PNLIPRP3* | -3.910887015 | 1.36E-20 | 1.20E-18 |  |  |  |  |
| *PNLIP* | 3.79208868 | 1.20E-18 | 8.00E-17 |  |  |  |  |
| *PSD* | 2.747077012 | 5.24E-34 | 1.21E-31 |  |  |  |  |
| *PTF1A* | 2.144055236 | 3.87E-07 | 2.61E-06 |  |  |  |  |
| *SORCS3* | 3.013097377 | 3.55E-23 | 4.04E-21 |  |  |  |  |
| *TACR2* | 3.405941548 | 1.08E-39 | 3.28E-37 |  |  |  |  |
| *AB231710* | 2.441276699 | 5.39E-13 | 1.30E-11 |  |  |  |  |
| *ADAMTS8* | 2.022153819 | 8.27E-20 | 6.61E-18 |  |  |  |  |
| *AK128059* | 2.541830689 | 8.41E-11 | 1.27E-09 |  |  |  |  |
| *AL833634* | 2.533406951 | 5.86E-24 | 7.40E-22 |  |  |  |  |
| *AX746604* | 2.266557628 | 4.11E-10 | 5.38E-09 |  |  |  |  |
| *BBOX1* | -2.551382893 | 1.93E-38 | 5.65E-36 |  |  |  |  |
| *BC027619* | 2.162179398 | 2.38E-06 | 1.32E-05 |  |  |  |  |
| *CHRDL2* | 2.200380092 | 1.42E-19 | 1.09E-17 |  |  |  |  |
| *FAT3* | 2.057757637 | 1.28E-18 | 8.51E-17 |  |  |  |  |
| *HEPHL1* | -3.681120912 | 2.06E-49 | 8.66E-47 |  |  |  |  |
| *KCNA4* | 2.944171453 | 6.58E-20 | 5.35E-18 |  |  |  |  |
| *LINC00301* | 2.178512948 | 2.34E-06 | 1.30E-05 |  |  |  |  |
| *LMO1* | 4.008279536 | 8.53E-34 | 1.95E-31 |  |  |  |  |
| *MRGPRF* | 2.302176565 | 1.30E-31 | 2.55E-29 |  |  |  |  |
| *MUC15* | -2.170951126 | 6.69E-09 | 6.68E-08 |  |  |  |  |
| *NCAM1* | 2.006987921 | 1.48E-21 | 1.43E-19 |  |  |  |  |
| *OR4D11* | 2.393990091 | 3.17E-07 | 2.18E-06 |  |  |  |  |
| *OR5A1* | 2.872437702 | 1.47E-09 | 1.72E-08 |  |  |  |  |
| *OR5AP2* | 2.389260432 | 2.35E-07 | 1.66E-06 |  |  |  |  |
| *OR8J1* | 2.020476742 | 5.77E-05 | 0.000229057 |  |  |  |  |
| *PGA3* | 2.202233324 | 3.08E-08 | 2.64E-07 |  |  |  |  |
| *PGA4* | 4.650565938 | 1.45E-33 | 3.18E-31 |  |  |  |  |
| *TAGLN* | 2.491720607 | 5.40E-35 | 1.36E-32 |  |  |  |  |
| *TP53AIP1* | -2.170657776 | 2.69E-25 | 3.82E-23 |  |  |  |  |
| *TRIM49B* | 2.342463776 | 2.90E-07 | 2.01E-06 |  |  |  |  |
| *TRIM64B* | 2.409768717 | 1.05E-06 | 6.34E-06 |  |  |  |  |
| *WT1-AS* | 2.545877329 | 6.89E-15 | 2.45E-13 |  |  |  |  |
| *A2ML1* | -4.943606041 | 7.08E-53 | 3.23E-50 |  |  |  |  |
| *AX748157* | 2.300256531 | 2.08E-12 | 4.47E-11 |  |  |  |  |
| *C12orf54* | -2.326589567 | 1.04E-15 | 4.29E-14 |  |  |  |  |
| *DQ591569* | 2.003733309 | 1.79E-05 | 8.08E-05 |  |  |  |  |
| *ENDOU* | -3.663110914 | 1.75E-63 | 1.20E-60 |  |  |  |  |
| *EPYC* | 2.463074654 | 2.45E-15 | 9.33E-14 |  |  |  |  |
| *FGF6* | 2.054089628 | 6.29E-06 | 3.16E-05 |  |  |  |  |
| *KCNA1* | 4.876588025 | 8.50E-48 | 3.40E-45 |  |  |  |  |
| *KERA* | 2.615938977 | 5.94E-24 | 7.47E-22 |  |  |  |  |
| *KRT3* | -2.37380922 | 6.71E-14 | 1.98E-12 |  |  |  |  |
| *KRT4* | -7.960785875 | 1.37E-118 | 5.92E-115 |  |  |  |  |
| *KRT5* | -6.254928127 | 1.68E-82 | 1.90E-79 |  |  |  |  |
| *KRT6A* | -5.940505767 | 1.12E-64 | 8.12E-62 |  |  |  |  |
| *KRT6B* | -4.205183318 | 1.70E-39 | 5.02E-37 |  |  |  |  |
| *KRT6C* | -7.097002734 | 9.79E-89 | 1.34E-85 |  |  |  |  |
| *KRT75* | -4.159455784 | 7.23E-27 | 1.13E-24 |  |  |  |  |
| *KRT78* | -7.301719583 | 2.51E-113 | 7.25E-110 |  |  |  |  |
| *LINC00477* | 2.01636617 | 5.33E-08 | 4.33E-07 |  |  |  |  |
| *LOC255480* | -2.708824062 | 9.18E-19 | 6.29E-17 |  |  |  |  |
| *LOC283392* | 2.181995092 | 3.00E-13 | 7.64E-12 |  |  |  |  |
| *OR6C65* | 2.412252063 | 4.38E-07 | 2.92E-06 |  |  |  |  |
| *OR8S1* | 2.087297797 | 2.51E-07 | 1.77E-06 |  |  |  |  |
| *PDZRN4* | 2.633877695 | 4.21E-22 | 4.33E-20 |  |  |  |  |
| *PPP1R1A* | 2.392472445 | 6.13E-20 | 5.02E-18 |  |  |  |  |
| *PRPH* | 2.248993763 | 3.20E-20 | 2.76E-18 |  |  |  |  |
| *SDR9C7* | -4.860155744 | 4.89E-44 | 1.67E-41 |  |  |  |  |
| *SYT10* | 2.452018509 | 1.33E-13 | 3.67E-12 |  |  |  |  |
| *WSCD2* | 2.185904243 | 3.32E-17 | 1.78E-15 |  |  |  |  |
| *Y_RNA.18* | 2.328650666 | 4.25E-08 | 3.54E-07 |  |  |  |  |
| *ATP12A* | -2.662584234 | 2.95E-18 | 1.84E-16 |  |  |  |  |
| *FAM123A* | 3.307964762 | 4.88E-17 | 2.51E-15 |  |  |  |  |
| *FAM194B* | 2.060980887 | 2.03E-06 | 1.15E-05 |  |  |  |  |
| *FGF14-IT1* | 2.011212127 | 7.55E-09 | 7.44E-08 |  |  |  |  |
| *GJB6* | -3.957017386 | 3.02E-42 | 9.95E-40 |  |  |  |  |
| *LINC00550* | 2.0784649 | 6.86E-06 | 3.42E-05 |  |  |  |  |
| *MLNR* | 2.518158796 | 1.87E-17 | 1.03E-15 |  |  |  |  |
| *Metazoa_SRP.20* | 2.046042864 | 5.40E-05 | 0.000215448 |  |  |  |  |
| *PCDH8* | 2.129541069 | 7.66E-12 | 1.46E-10 |  |  |  |  |
| *RXFP2* | 2.092882943 | 4.43E-07 | 2.94E-06 |  |  |  |  |
| *SCEL* | -3.524611501 | 5.38E-31 | 1.04E-28 |  |  |  |  |
| *SERTM1* | 2.906521811 | 2.67E-14 | 8.51E-13 |  |  |  |  |
| *SLITRK5* | 2.69777894 | 9.73E-22 | 9.66E-20 |  |  |  |  |
| *ASB2* | 2.151441391 | 9.91E-26 | 1.49E-23 |  |  |  |  |
| *AX746996.1* | 2.040763432 | 1.89E-05 | 8.46E-05 |  |  |  |  |
| *C14orf177* | 2.045421403 | 1.72E-05 | 7.80E-05 |  |  |  |  |
| *C14orf23* | 2.034093084 | 1.29E-06 | 7.64E-06 |  |  |  |  |
| *CMTM5* | 2.185951771 | 1.01E-14 | 3.52E-13 |  |  |  |  |
| *DLK1* | 3.532723301 | 8.33E-27 | 1.29E-24 |  |  |  |  |
| *NGB* | 2.088210425 | 7.85E-08 | 6.14E-07 |  |  |  |  |
| *NRXN3* | 2.273977104 | 4.55E-23 | 5.08E-21 |  |  |  |  |
| *OR4K2* | 2.012988539 | 9.83E-06 | 4.72E-05 |  |  |  |  |
| *PAX9* | -2.67447858 | 2.06E-38 | 5.94E-36 |  |  |  |  |
| *PRIMA1* | 2.221861582 | 1.84E-18 | 1.18E-16 |  |  |  |  |
| *RNASE11* | 2.156080288 | 1.58E-05 | 7.21E-05 |  |  |  |  |
| *RNASE7* | -3.217594757 | 1.66E-21 | 1.60E-19 |  |  |  |  |
| *SLC39A2* | -2.970069356 | 3.23E-21 | 3.01E-19 |  |  |  |  |
| *TGM1* | -5.989355039 | 1.99E-153 | 5.19E-149 |  |  |  |  |
| *TMEM179* | 2.026075065 | 1.14E-07 | 8.60E-07 |  |  |  |  |
| *TRD* | 2.001831382 | 9.73E-17 | 4.74E-15 |  |  |  |  |
| *BNC1* | -3.92911077 | 2.85E-46 | 1.06E-43 |  |  |  |  |
| *CERS3* | -5.638290557 | 2.03E-86 | 2.51E-83 |  |  |  |  |
| *CHRNA3* | 2.620845825 | 3.33E-32 | 6.72E-30 |  |  |  |  |
| *CTXN2* | 2.048706249 | 6.10E-07 | 3.92E-06 |  |  |  |  |
| *CYP1A2* | 2.102158097 | 3.48E-09 | 3.72E-08 |  |  |  |  |
| *DUOX1* | -2.41171162 | 8.55E-38 | 2.39E-35 |  |  |  |  |
| *FAM189A1* | 2.06668785 | 1.10E-14 | 3.83E-13 |  |  |  |  |
| *LOC283738* | 2.045093543 | 4.94E-06 | 2.54E-05 |  |  |  |  |
| *PLA2G4E* | -2.890815796 | 3.10E-33 | 6.56E-31 |  |  |  |  |
| *PWRN2* | 2.385569149 | 2.37E-08 | 2.09E-07 |  |  |  |  |
| *RBPMS2* | 2.069383199 | 2.76E-22 | 2.91E-20 |  |  |  |  |
| *RGMA* | 2.076782054 | 7.62E-20 | 6.14E-18 |  |  |  |  |
| *RHCG* | -7.504115257 | 9.93E-144 | 1.29E-139 |  |  |  |  |
| *SYNM* | 2.953084913 | 6.10E-30 | 1.10E-27 |  |  |  |  |
| *TGM5* | -4.48273125 | 7.74E-47 | 3.01E-44 |  |  |  |  |
| *AX747287* | 2.141995729 | 5.84E-06 | 2.96E-05 |  |  |  |  |
| *BC043527* | 2.17405363 | 1.61E-08 | 1.47E-07 |  |  |  |  |
| *CAPNS2* | -5.057868488 | 2.45E-69 | 2.12E-66 |  |  |  |  |
| *CBLN1* | 2.323301188 | 4.32E-17 | 2.26E-15 |  |  |  |  |
| *CTRB1* | 2.133060773 | 5.98E-09 | 6.03E-08 |  |  |  |  |
| *DKFZP434H168* | 2.200442955 | 4.83E-09 | 4.98E-08 |  |  |  |  |
| *GNAO1* | 2.51276838 | 1.67E-32 | 3.40E-30 |  |  |  |  |
| *HP* | 2.217154617 | 9.24E-19 | 6.31E-17 |  |  |  |  |
| *LOC100506172* | 2.358432505 | 2.10E-09 | 2.36E-08 |  |  |  |  |
| *LOC283856* | 2.223306808 | 2.27E-20 | 1.98E-18 |  |  |  |  |
| *LOC283914* | 2.09955681 | 1.99E-05 | 8.84E-05 |  |  |  |  |
| *MYH11* | 3.018324508 | 3.82E-29 | 6.59E-27 |  |  |  |  |
| *PPL* | -2.586069902 | 7.67E-55 | 3.69E-52 |  |  |  |  |
| *PRSS27* | -4.760767466 | 4.23E-129 | 2.20E-125 |  |  |  |  |
| *SCNN1B* | -2.341225334 | 8.85E-22 | 8.82E-20 |  |  |  |  |
| *AK301679* | -2.107228826 | 9.56E-07 | 5.85E-06 |  |  |  |  |
| *ALOX12B* | -2.010511302 | 1.31E-19 | 1.02E-17 |  |  |  |  |
| *FOXN1* | -2.558806865 | 5.10E-24 | 6.50E-22 |  |  |  |  |
| *GAST* | 3.11000031 | 2.50E-19 | 1.89E-17 |  |  |  |  |
| *GIP* | 2.530996826 | 6.69E-14 | 1.98E-12 |  |  |  |  |
| *GLP2R* | 2.714096297 | 2.18E-33 | 4.64E-31 |  |  |  |  |
| *KRT13* | -7.787237851 | 1.19E-116 | 3.87E-113 |  |  |  |  |
| *KRT14* | -7.098732708 | 1.94E-105 | 3.61E-102 |  |  |  |  |
| *KRT15* | -2.776174517 | 1.26E-37 | 3.50E-35 |  |  |  |  |
| *KRT16P2* | -2.570710554 | 1.46E-14 | 4.93E-13 |  |  |  |  |
| *KRT16P3* | -2.556420707 | 9.05E-10 | 1.10E-08 |  |  |  |  |
| *KRT16* | -4.956386927 | 5.29E-69 | 4.30E-66 |  |  |  |  |
| *KRT24* | -4.976999218 | 2.31E-40 | 7.16E-38 |  |  |  |  |
| *MYH2* | 2.227760787 | 6.67E-14 | 1.97E-12 |  |  |  |  |
| *MYOCD* | 2.065970174 | 3.71E-20 | 3.17E-18 |  |  |  |  |
| *NPTX1* | 3.22061602 | 1.40E-33 | 3.10E-31 |  |  |  |  |
| *OR1E2* | 2.041421026 | 7.41E-06 | 3.66E-05 |  |  |  |  |
| *OTOP2* | -2.976338664 | 5.21E-22 | 5.29E-20 |  |  |  |  |
| *PIRT* | 2.309802518 | 2.31E-19 | 1.76E-17 |  |  |  |  |
| *RNF222* | -4.374854725 | 4.46E-69 | 3.74E-66 |  |  |  |  |
| *SLC35G3* | 2.434369805 | 1.22E-11 | 2.21E-10 |  |  |  |  |
| *TMEM100* | 2.021094347 | 4.81E-21 | 4.41E-19 |  |  |  |  |
| *TUSC5* | 2.810990981 | 6.36E-18 | 3.76E-16 |  |  |  |  |
| *U1.5* | 2.144161537 | 6.31E-06 | 3.17E-05 |  |  |  |  |
| *USH1G* | -2.070218049 | 3.82E-11 | 6.23E-10 |  |  |  |  |
| *ZNF750* | -3.807949815 | 9.20E-52 | 4.06E-49 |  |  |  |  |
| *BC037384* | 2.230918358 | 2.84E-06 | 1.55E-05 |  |  |  |  |
| *C18orf26* | -4.113973645 | 9.45E-25 | 1.27E-22 |  |  |  |  |
| *DSC3* | -4.177384763 | 4.44E-56 | 2.36E-53 |  |  |  |  |
| *DSG1* | -2.868911004 | 1.87E-27 | 3.00E-25 |  |  |  |  |
| *DSG3* | -3.065994749 | 1.02E-23 | 1.25E-21 |  |  |  |  |
| *SERPINB11* | -4.081295297 | 1.62E-21 | 1.57E-19 |  |  |  |  |
| *SERPINB12* | -2.388443946 | 6.82E-10 | 8.52E-09 |  |  |  |  |
| *SERPINB13* | -7.0806189 | 2.03E-70 | 1.82E-67 |  |  |  |  |
| *SERPINB2* | -2.78066235 | 2.05E-21 | 1.96E-19 |  |  |  |  |
| *SERPINB3* | -3.433016544 | 2.06E-22 | 2.19E-20 |  |  |  |  |
| *SYT4* | 2.613605123 | 5.07E-17 | 2.59E-15 |  |  |  |  |
| *BSPH1* | 2.704952603 | 6.54E-10 | 8.22E-09 |  |  |  |  |
| *CABP5* | 2.065147979 | 3.50E-07 | 2.39E-06 |  |  |  |  |
| *CLC* | 3.238617077 | 2.74E-29 | 4.78E-27 |  |  |  |  |
| *CNFN* | -4.87663611 | 2.86E-108 | 6.19E-105 |  |  |  |  |
| *CNN1* | 3.856507427 | 5.32E-49 | 2.20E-46 |  |  |  |  |
| *CRX* | 2.200651002 | 2.57E-09 | 2.83E-08 |  |  |  |  |
| *CYP4F22* | -2.70722693 | 4.72E-42 | 1.54E-39 |  |  |  |  |
| *FLJ25758* | 2.044211221 | 9.76E-08 | 7.47E-07 |  |  |  |  |
| *HSPB6* | 3.561699732 | 6.86E-42 | 2.20E-39 |  |  |  |  |
| *IGFL1* | -2.321569688 | 7.05E-12 | 1.35E-10 |  |  |  |  |
| *IGFL2* | 2.214296412 | 5.22E-20 | 4.31E-18 |  |  |  |  |
| *KLK12* | -3.318899883 | 1.57E-28 | 2.64E-26 |  |  |  |  |
| *KLK13* | -4.747300058 | 3.34E-60 | 2.02E-57 |  |  |  |  |
| *KLK5* | -3.490158003 | 1.00E-18 | 6.81E-17 |  |  |  |  |
| *KRTDAP* | -5.353963709 | 3.89E-55 | 1.91E-52 |  |  |  |  |
| *LGALS7B* | -2.798026458 | 2.82E-20 | 2.44E-18 |  |  |  |  |
| *LGALS7* | -2.847077482 | 7.92E-13 | 1.85E-11 |  |  |  |  |
| *LOC646862* | -2.955570604 | 4.25E-25 | 5.91E-23 |  |  |  |  |
| *LYPD3* | -3.463136225 | 1.63E-53 | 7.58E-51 |  |  |  |  |
| *LYPD4* | 2.087034139 | 9.60E-10 | 1.16E-08 |  |  |  |  |
| *MBD3L1* | 2.314936194 | 1.13E-06 | 6.78E-06 |  |  |  |  |
| *MIMT1* | 2.076278754 | 1.67E-06 | 9.67E-06 |  |  |  |  |
| *MIR371A* | 2.547846002 | 2.44E-09 | 2.71E-08 |  |  |  |  |
| *NCCRP1* | -4.286709487 | 8.44E-56 | 4.22E-53 |  |  |  |  |
| *NLRP13* | 2.536297864 | 5.39E-09 | 5.50E-08 |  |  |  |  |
| *OR1M1* | 2.097072541 | 1.55E-05 | 7.08E-05 |  |  |  |  |
| *OR7A17* | 2.046550256 | 2.82E-05 | 0.000120765 |  |  |  |  |
| *OR7C2* | 2.005688921 | 2.94E-05 | 0.000125388 |  |  |  |  |
| *OR7D4* | 2.537524424 | 1.57E-07 | 1.15E-06 |  |  |  |  |
| *OR7E24* | 2.155393453 | 8.63E-06 | 4.20E-05 |  |  |  |  |
| *OR7G3* | 2.687216441 | 4.47E-09 | 4.65E-08 |  |  |  |  |
| *PLIN4* | 2.6395913 | 1.67E-25 | 2.42E-23 |  |  |  |  |
| *PSG4* | -2.273470532 | 1.60E-11 | 2.83E-10 |  |  |  |  |
| *SBSN* | -7.195009776 | 4.28E-110 | 1.01E-106 |  |  |  |  |
| *USP29* | 2.210283973 | 2.17E-07 | 1.55E-06 |  |  |  |  |
| *VSIG10L* | -2.355067868 | 3.45E-38 | 9.87E-36 |  |  |  |  |
| *ZIM3* | 2.733570041 | 6.03E-10 | 7.62E-09 |  |  |  |  |
| *ZNF812* | -3.009279243 | 2.09E-29 | 3.70E-27 |  |  |  |  |
| *ACTG2* | 3.991226728 | 1.93E-49 | 8.22E-47 |  |  |  |  |
| *BC048132* | 2.053118108 | 2.18E-09 | 2.44E-08 |  |  |  |  |
| *C2orf40* | 3.305425976 | 1.53E-30 | 2.85E-28 |  |  |  |  |
| *C2orf53* | 2.159004705 | 4.48E-09 | 4.66E-08 |  |  |  |  |
| *CAPN14* | -6.032577757 | 3.44E-131 | 2.24E-127 |  |  |  |  |
| *DES* | 3.847016311 | 1.39E-37 | 3.79E-35 |  |  |  |  |
| *DPP10* | 2.254368991 | 2.74E-11 | 4.61E-10 |  |  |  |  |
| *DPYSL5* | 2.595619788 | 4.69E-19 | 3.38E-17 |  |  |  |  |
| *GKN1* | 4.009210147 | 2.15E-25 | 3.11E-23 |  |  |  |  |
| *GKN2* | 3.496682805 | 1.47E-20 | 1.29E-18 |  |  |  |  |
| *IL36A* | -6.988227733 | 7.16E-61 | 4.43E-58 |  |  |  |  |
| *IL36B* | -2.416835476 | 2.28E-11 | 3.90E-10 |  |  |  |  |
| *IL36G* | -5.022775272 | 5.90E-64 | 4.15E-61 |  |  |  |  |
| *IL36RN* | -5.439630252 | 4.05E-52 | 1.82E-49 |  |  |  |  |
| *LOC100505964* | 2.514271782 | 1.40E-08 | 1.29E-07 |  |  |  |  |
| *MAL* | -5.00121479 | 2.96E-78 | 3.08E-75 |  |  |  |  |
| *MYADML* | 2.305074348 | 2.83E-13 | 7.27E-12 |  |  |  |  |
| *MYT1L* | 2.201995886 | 2.25E-13 | 5.91E-12 |  |  |  |  |
| *NRXN1* | 2.219881904 | 1.53E-16 | 7.14E-15 |  |  |  |  |
| *NXPH2* | 2.93797701 | 6.66E-17 | 3.32E-15 |  |  |  |  |
| *RN7SK.54* | 2.001390966 | 4.13E-06 | 2.17E-05 |  |  |  |  |
| *SLC5A7* | 2.450052057 | 8.86E-17 | 4.35E-15 |  |  |  |  |
| *SMYD1* | 5.579477605 | 3.45E-54 | 1.63E-51 |  |  |  |  |
| *SPHKAP* | 2.342144198 | 6.02E-12 | 1.18E-10 |  |  |  |  |
| *TCF23* | 2.828643493 | 1.57E-17 | 8.77E-16 |  |  |  |  |
| *TISP43.1* | 2.055540556 | 1.20E-05 | 5.67E-05 |  |  |  |  |
| *XIRP2* | 2.176207021 | 1.18E-10 | 1.72E-09 |  |  |  |  |
| *BPIFA3* | 2.353524639 | 1.30E-06 | 7.72E-06 |  |  |  |  |
| *C20orf166-AS1* | 2.611970625 | 3.69E-22 | 3.81E-20 |  |  |  |  |
| *C20orf166* | 2.81637077 | 1.80E-14 | 5.94E-13 |  |  |  |  |
| *DEFB118* | 2.52409895 | 8.52E-11 | 1.28E-09 |  |  |  |  |
| *DEFB122.1* | 2.502033454 | 4.73E-08 | 3.90E-07 |  |  |  |  |
| *DEFB122.2* | 2.404796109 | 1.07E-07 | 8.16E-07 |  |  |  |  |
| *DEFB127* | 2.006279006 | 6.04E-05 | 0.000238464 |  |  |  |  |
| *FAM83C* | -3.897922779 | 9.11E-31 | 1.74E-28 |  |  |  |  |
| *JPH2* | 2.050910768 | 6.41E-20 | 5.23E-18 |  |  |  |  |
| *KCNB1* | 2.091600404 | 3.96E-13 | 9.88E-12 |  |  |  |  |
| *LOC100287792* | 2.139446896 | 9.10E-09 | 8.77E-08 |  |  |  |  |
| *MYL9* | 2.493856077 | 2.45E-34 | 5.84E-32 |  |  |  |  |
| *Metazoa_SRP.60* | 2.097773 | 4.14E-06 | 2.18E-05 |  |  |  |  |
| *PCSK2* | 2.106380235 | 1.80E-13 | 4.81E-12 |  |  |  |  |
| *PRNT* | 2.039207646 | 2.27E-06 | 1.26E-05 |  |  |  |  |
| *SEL1L2* | 2.486955327 | 1.32E-18 | 8.72E-17 |  |  |  |  |
| *SUN5* | 2.102611201 | 4.70E-06 | 2.43E-05 |  |  |  |  |
| *TGM3* | -5.000438674 | 2.36E-82 | 2.56E-79 |  |  |  |  |
| *WFDC12* | -2.564727124 | 3.60E-20 | 3.08E-18 |  |  |  |  |
| *WFDC5* | -4.053198437 | 7.60E-35 | 1.88E-32 |  |  |  |  |
| *BC035642* | 2.056512828 | 5.29E-06 | 2.71E-05 |  |  |  |  |
| *C21orf54* | 2.166348204 | 1.13E-06 | 6.77E-06 |  |  |  |  |
| *CLDN17* | -2.707620683 | 9.61E-08 | 7.37E-07 |  |  |  |  |
| *DQ586768.5* | -2.130278512 | 2.74E-06 | 1.50E-05 |  |  |  |  |
| *LINC00320* | 2.207433096 | 4.24E-07 | 2.83E-06 |  |  |  |  |
| *AK123632* | 2.285894194 | 8.21E-07 | 5.11E-06 |  |  |  |  |
| *BC015159* | 2.172677277 | 4.01E-19 | 2.90E-17 |  |  |  |  |
| *CACNG2* | 2.957397166 | 6.27E-17 | 3.16E-15 |  |  |  |  |
| *CCT8L2* | 2.462088919 | 1.32E-08 | 1.22E-07 |  |  |  |  |
| *KIAA1644* | 2.325863623 | 7.69E-28 | 1.26E-25 |  |  |  |  |
| *PHF21B* | 2.062609762 | 4.19E-14 | 1.29E-12 |  |  |  |  |
| *SEZ6L* | 2.828706064 | 6.20E-36 | 1.61E-33 |  |  |  |  |
| *ADIPOQ* | 2.968316555 | 7.37E-14 | 2.15E-12 |  |  |  |  |
| *AGTR1* | 2.558037215 | 6.45E-22 | 6.53E-20 |  |  |  |  |
| *ARPP21.1* | 2.907725653 | 7.91E-16 | 3.32E-14 |  |  |  |  |
| *C3orf77* | 2.371807974 | 4.02E-09 | 4.23E-08 |  |  |  |  |
| *CPB1* | 2.283512678 | 2.84E-13 | 7.28E-12 |  |  |  |  |
| *CSTA* | -4.26891531 | 6.83E-87 | 8.89E-84 |  |  |  |  |
| *FAM19A4* | 3.701599739 | 8.95E-29 | 1.51E-26 |  |  |  |  |
| *IL20RB* | -2.129307883 | 7.60E-32 | 1.51E-29 |  |  |  |  |
| *IQCF2* | 2.263056158 | 2.34E-06 | 1.30E-05 |  |  |  |  |
| *LOC253573* | 2.015280186 | 1.75E-06 | 1.00E-05 |  |  |  |  |
| *LOC285375* | 2.339724046 | 7.26E-07 | 4.58E-06 |  |  |  |  |
| *MYLK* | 2.35867175 | 4.33E-29 | 7.41E-27 |  |  |  |  |
| *POPDC2* | 2.410469102 | 2.94E-34 | 6.88E-32 |  |  |  |  |
| *RBP2* | 2.116122268 | 6.96E-12 | 1.34E-10 |  |  |  |  |
| *SLC7A14* | 3.548940633 | 1.24E-34 | 3.00E-32 |  |  |  |  |
| *TMEM40* | -3.591096476 | 8.76E-34 | 1.98E-31 |  |  |  |  |
| *TP63* | -2.947628238 | 1.46E-34 | 3.52E-32 |  |  |  |  |
| *VENTXP7* | 2.106686507 | 5.38E-08 | 4.36E-07 |  |  |  |  |
| *WNT7A* | -2.702095777 | 2.09E-17 | 1.14E-15 |  |  |  |  |
| *ADH1A* | 2.349483582 | 2.91E-20 | 2.52E-18 |  |  |  |  |
| *ADH4* | 2.116517203 | 9.83E-14 | 2.79E-12 |  |  |  |  |
| *ADH7* | -4.032175543 | 6.04E-25 | 8.27E-23 |  |  |  |  |
| *AFP* | 3.034167555 | 6.57E-30 | 1.18E-27 |  |  |  |  |
| *AK093205* | 2.20084176 | 6.58E-07 | 4.19E-06 |  |  |  |  |
| *ALB* | 2.778638848 | 8.27E-28 | 1.34E-25 |  |  |  |  |
| *AMTN* | -2.318792309 | 2.34E-10 | 3.23E-09 |  |  |  |  |
| *ASB5* | 2.525561111 | 1.27E-11 | 2.29E-10 |  |  |  |  |
| *BC025350* | 2.435730304 | 1.51E-07 | 1.11E-06 |  |  |  |  |
| *BC034799* | 2.226831004 | 7.37E-06 | 3.64E-05 |  |  |  |  |
| *BC040219* | 2.50890506 | 1.03E-12 | 2.36E-11 |  |  |  |  |
| *BC041818* | 2.001599848 | 1.05E-05 | 5.00E-05 |  |  |  |  |
| *BC042378* | 2.22411654 | 1.19E-08 | 1.12E-07 |  |  |  |  |
| *BC043280* | 2.061956628 | 4.26E-06 | 2.23E-05 |  |  |  |  |
| *BC048420* | 2.019911252 | 2.47E-05 | 0.000107282 |  |  |  |  |
| *BMP3* | 3.202167905 | 5.22E-25 | 7.19E-23 |  |  |  |  |
| *CCKAR* | 3.59218478 | 4.36E-23 | 4.89E-21 |  |  |  |  |
| *CWH43* | -3.124088131 | 1.06E-13 | 2.97E-12 |  |  |  |  |
| *DEFB131* | 2.303881085 | 1.11E-06 | 6.65E-06 |  |  |  |  |
| *EPGN* | -4.711845483 | 7.58E-48 | 3.08E-45 |  |  |  |  |
| *FTLP10* | -2.762625089 | 4.39E-08 | 3.65E-07 |  |  |  |  |
| *GPR78* | -2.43039112 | 1.54E-11 | 2.73E-10 |  |  |  |  |
| *HAND2* | 5.292082924 | 1.53E-82 | 1.81E-79 |  |  |  |  |
| *KCTD8* | 2.432154748 | 5.96E-15 | 2.15E-13 |  |  |  |  |
| *LOC285547* | 3.583661005 | 1.16E-16 | 5.57E-15 |  |  |  |  |
| *NBLA00301* | 5.027280982 | 1.59E-67 | 1.25E-64 |  |  |  |  |
| *NKX3-2* | 4.043742127 | 5.95E-66 | 4.54E-63 |  |  |  |  |
| *NKX6-1* | -2.780792029 | 2.13E-23 | 2.50E-21 |  |  |  |  |
| *PCDH10* | 2.909903222 | 3.56E-22 | 3.68E-20 |  |  |  |  |
| *PHOX2B* | 4.791129514 | 7.73E-36 | 1.99E-33 |  |  |  |  |
| *SCRG1* | 3.798847269 | 1.16E-43 | 3.92E-41 |  |  |  |  |
| *SYNPO2* | 2.986827525 | 4.69E-32 | 9.38E-30 |  |  |  |  |
| *TMPRSS11A* | -7.923550484 | 1.22E-98 | 1.98E-95 |  |  |  |  |
| *TMPRSS11BNL* | -3.498325347 | 8.22E-14 | 2.37E-12 |  |  |  |  |
| *TMPRSS11B* | -7.436000374 | 1.20E-70 | 1.15E-67 |  |  |  |  |
| *TMPRSS11D* | -7.620620166 | 7.17E-134 | 6.22E-130 |  |  |  |  |
| *TMPRSS11E* | -6.707876996 | 3.16E-90 | 4.83E-87 |  |  |  |  |
| *TMPRSS11F* | -5.610968794 | 1.05E-46 | 4.02E-44 |  |  |  |  |
| *TRIM60* | 2.108214185 | 5.49E-06 | 2.80E-05 |  |  |  |  |
| *ADCY2* | 2.058545585 | 1.12E-22 | 1.20E-20 |  |  |  |  |
| *AK000840* | 2.666333765 | 6.37E-09 | 6.39E-08 |  |  |  |  |
| *AX748028* | 2.049077306 | 9.23E-07 | 5.67E-06 |  |  |  |  |
| *BC034636* | -2.352205738 | 2.65E-17 | 1.43E-15 |  |  |  |  |
| *C7* | 2.278935396 | 3.89E-17 | 2.05E-15 |  |  |  |  |
| *CARTPT* | 5.993713904 | 2.62E-58 | 1.48E-55 |  |  |  |  |
| *CTNND2* | 2.36819423 | 6.21E-19 | 4.37E-17 |  |  |  |  |
| *DL490294* | 2.004031058 | 9.33E-07 | 5.72E-06 |  |  |  |  |
| *FAT2* | -3.861753802 | 2.67E-62 | 1.73E-59 |  |  |  |  |
| *FBXL21* | 2.581130479 | 1.52E-10 | 2.17E-09 |  |  |  |  |
| *FGF10* | 2.036520852 | 1.84E-18 | 1.18E-16 |  |  |  |  |
| *GABRA1* | 2.157201569 | 1.83E-08 | 1.65E-07 |  |  |  |  |
| *HMP19* | 2.504591211 | 2.13E-22 | 2.26E-20 |  |  |  |  |
| *IRX4* | -2.429342284 | 7.69E-11 | 1.17E-09 |  |  |  |  |
| *KCNMB1* | 2.175459501 | 3.58E-25 | 5.00E-23 |  |  |  |  |
| *LOC340107* | 2.081790085 | 2.93E-05 | 0.000124834 |  |  |  |  |
| *NKX2-5* | 2.87769951 | 4.18E-17 | 2.19E-15 |  |  |  |  |
| *SPINK5* | -4.092884026 | 6.10E-66 | 4.54E-63 |  |  |  |  |
| *SPINK6* | -2.821002081 | 4.82E-18 | 2.91E-16 |  |  |  |  |
| *SPINK7* | -4.822158491 | 2.90E-46 | 1.06E-43 |  |  |  |  |
| *THBS4* | 2.175204547 | 3.96E-15 | 1.46E-13 |  |  |  |  |
| *AF086258* | 2.299363581 | 5.77E-20 | 4.75E-18 |  |  |  |  |
| *AK056584* | -2.121562534 | 8.58E-24 | 1.07E-21 |  |  |  |  |
| *CLVS2* | 2.920656539 | 4.10E-13 | 1.02E-11 |  |  |  |  |
| *HCG22* | -2.206612338 | 5.98E-19 | 4.21E-17 |  |  |  |  |
| *HMGA1P7* | 2.100067003 | 2.97E-07 | 2.05E-06 |  |  |  |  |
| *KHDRBS2* | 2.480709994 | 3.49E-20 | 3.00E-18 |  |  |  |  |
| *METTL24* | 2.275936007 | 4.74E-29 | 8.06E-27 |  |  |  |  |
| *MLN* | 2.564088206 | 7.03E-11 | 1.09E-09 |  |  |  |  |
| *MUC21* | -7.926362152 | 3.60E-112 | 9.38E-109 |  |  |  |  |
| *MUC22* | -4.487874013 | 1.63E-36 | 4.38E-34 |  |  |  |  |
| *NRSN1* | 2.446109328 | 1.29E-15 | 5.18E-14 |  |  |  |  |
| *OR11A1* | 2.273630432 | 1.75E-07 | 1.27E-06 |  |  |  |  |
| *PLN* | 2.344964442 | 3.89E-23 | 4.40E-21 |  |  |  |  |
| *RAET1E* | -2.95967242 | 4.64E-61 | 2.94E-58 |  |  |  |  |
| *RAET1L* | -2.238631499 | 8.80E-19 | 6.04E-17 |  |  |  |  |
| *RIPPLY2* | 2.062526983 | 2.52E-08 | 2.21E-07 |  |  |  |  |
| *SNAP91* | 2.077981078 | 4.81E-14 | 1.47E-12 |  |  |  |  |
| *TAAR5* | 2.14034525 | 1.10E-06 | 6.62E-06 |  |  |  |  |
| *TRNA_Arg.15* | 2.373024651 | 1.06E-06 | 6.41E-06 |  |  |  |  |
| *VIP* | 3.787459824 | 8.55E-45 | 2.96E-42 |  |  |  |  |
| *ADCYAP1R1* | 2.299481837 | 1.44E-21 | 1.41E-19 |  |  |  |  |
| *BC035176* | 2.1065592 | 2.88E-07 | 2.00E-06 |  |  |  |  |
| *C7orf66* | 2.330414715 | 4.11E-08 | 3.43E-07 |  |  |  |  |
| *C7orf72* | 2.114004415 | 8.49E-07 | 5.25E-06 |  |  |  |  |
| *CHRM2* | 3.86218738 | 9.83E-34 | 2.21E-31 |  |  |  |  |
| *CPA1* | 3.26151348 | 9.48E-24 | 1.17E-21 |  |  |  |  |
| *DPP6* | 3.693715932 | 1.40E-39 | 4.17E-37 |  |  |  |  |
| *EN2* | 2.034933893 | 7.89E-10 | 9.69E-09 |  |  |  |  |
| *FLNC* | 2.803845837 | 2.74E-30 | 4.99E-28 |  |  |  |  |
| *HPVC1* | 2.058856473 | 1.07E-05 | 5.09E-05 |  |  |  |  |
| *HTR5A* | 2.437090518 | 4.88E-08 | 4.01E-07 |  |  |  |  |
| *LOC100128264* | 2.028577032 | 3.13E-05 | 0.000132392 |  |  |  |  |
| *Metazoa_SRP.96* | 2.524798092 | 8.54E-09 | 8.29E-08 |  |  |  |  |
| *NPY* | 2.264570168 | 1.88E-13 | 4.98E-12 |  |  |  |  |
| *OR6W1P* | 2.200822367 | 6.76E-09 | 6.74E-08 |  |  |  |  |
| *SEPT14* | 2.129273851 | 1.91E-08 | 1.72E-07 |  |  |  |  |
| *TAC1* | 4.396927569 | 8.51E-38 | 2.39E-35 |  |  |  |  |
| *U6.151* | 2.133882271 | 1.90E-05 | 8.49E-05 |  |  |  |  |
| *VIPR2* | 2.074272143 | 4.27E-21 | 3.92E-19 |  |  |  |  |
| *VWC2* | 2.135143997 | 2.18E-15 | 8.42E-14 |  |  |  |  |
| *ADRB3* | 2.535329283 | 4.06E-20 | 3.41E-18 |  |  |  |  |
| *AX747062* | 2.038073129 | 3.18E-11 | 5.29E-10 |  |  |  |  |
| *BC042029* | 2.230173457 | 1.56E-06 | 9.05E-06 |  |  |  |  |
| *C8orf87* | 2.168371861 | 4.00E-07 | 2.69E-06 |  |  |  |  |
| *CCDC26* | 2.086640325 | 1.71E-10 | 2.42E-09 |  |  |  |  |
| *FABP12* | -2.074780845 | 3.39E-08 | 2.89E-07 |  |  |  |  |
| *FAM83A* | -2.14045345 | 4.38E-16 | 1.91E-14 |  |  |  |  |
| *GSDMC* | -2.393576991 | 9.46E-33 | 1.94E-30 |  |  |  |  |
| *LOC100127983* | 2.197884077 | 3.04E-26 | 4.67E-24 |  |  |  |  |
| *LOC100192378* | 3.046910836 | 2.15E-31 | 4.20E-29 |  |  |  |  |
| *LOC286094* | 2.347546331 | 3.07E-07 | 2.12E-06 |  |  |  |  |
| *LY6D* | -2.825566115 | 3.80E-17 | 2.02E-15 |  |  |  |  |
| *NKX6-3* | 2.645268319 | 5.01E-17 | 2.56E-15 |  |  |  |  |
| *SLURP1* | -4.790547769 | 2.57E-42 | 8.58E-40 |  |  |  |  |
| *SPAG11B.1* | 2.505478977 | 5.01E-08 | 4.10E-07 |  |  |  |  |
| *STMN2* | 2.742230626 | 6.48E-28 | 1.07E-25 |  |  |  |  |
| *STMN4* | 2.02224762 | 2.42E-13 | 6.28E-12 |  |  |  |  |
| *AK093363* | 2.142629279 | 2.53E-07 | 1.78E-06 |  |  |  |  |
| *AK128153* | -2.603549751 | 1.03E-35 | 2.64E-33 |  |  |  |  |
| *AK130904* | 2.240160332 | 3.43E-19 | 2.52E-17 |  |  |  |  |
| *AL390170* | 2.730573319 | 1.17E-19 | 9.20E-18 |  |  |  |  |
| *ANXA1* | -2.374921606 | 4.26E-45 | 1.52E-42 |  |  |  |  |
| *AX747119.1* | 2.054141017 | 1.62E-07 | 1.18E-06 |  |  |  |  |
| *AX747119.2* | 2.000851116 | 4.26E-08 | 3.54E-07 |  |  |  |  |
| *BARX1* | 3.360651057 | 3.92E-47 | 1.55E-44 |  |  |  |  |
| *BC035187* | 2.389193565 | 1.79E-08 | 1.61E-07 |  |  |  |  |
| *C9orf169* | -2.942660776 | 1.94E-41 | 6.16E-39 |  |  |  |  |
| *DQ582785* | 2.028423936 | 4.15E-05 | 0.000170792 |  |  |  |  |
| *FAM75D3* | 2.082516759 | 3.23E-05 | 0.000136183 |  |  |  |  |
| *FOXE1* | -3.923013851 | 4.72E-28 | 7.82E-26 |  |  |  |  |
| *FRRS1L* | 3.391468422 | 4.42E-28 | 7.38E-26 |  |  |  |  |
| *GLT6D1* | 2.345424999 | 8.64E-08 | 6.70E-07 |  |  |  |  |
| *LOC340515* | 2.088173271 | 9.03E-10 | 1.10E-08 |  |  |  |  |
| *LOC572558* | 2.811412775 | 6.28E-19 | 4.40E-17 |  |  |  |  |
| *MIR101-2* | 2.101640768 | 2.99E-06 | 1.63E-05 |  |  |  |  |
| *MORN5* | 3.722300276 | 1.21E-30 | 2.29E-28 |  |  |  |  |
| *OGN* | 2.149256669 | 2.66E-16 | 1.20E-14 |  |  |  |  |
| *PGM5* | 2.466142959 | 2.17E-25 | 3.12E-23 |  |  |  |  |
| *RMRP* | 2.728620259 | 8.33E-45 | 2.93E-42 |  |  |  |  |
| *TPM2* | 2.013519666 | 1.27E-25 | 1.87E-23 |  |  |  |  |
| *FLJ44838* | 2.989002716 | 1.06E-12 | 2.42E-11 |  |  |  |  |
| *AGTR2* | 2.469173957 | 1.21E-09 | 1.44E-08 |  |  |  |  |
| *AK056105* | 2.43881487 | 1.93E-11 | 3.35E-10 |  |  |  |  |
| *ARSF* | -2.761552123 | 1.50E-20 | 1.31E-18 |  |  |  |  |
| *CNKSR2* | 2.24123578 | 3.33E-19 | 2.45E-17 |  |  |  |  |
| *DCX* | 2.429868271 | 3.85E-19 | 2.80E-17 |  |  |  |  |
| *DKFZp686D0853* | 2.25974838 | 1.02E-20 | 9.17E-19 |  |  |  |  |
| *FAM48B2* | 2.516442489 | 5.08E-12 | 1.01E-10 |  |  |  |  |
| *FHL1* | 2.174120229 | 5.21E-25 | 7.19E-23 |  |  |  |  |
| *JA202350* | 2.197284419 | 1.45E-11 | 2.58E-10 |  |  |  |  |
| *JA202363* | 2.003919094 | 1.21E-10 | 1.76E-09 |  |  |  |  |
| *NRK* | 2.136064126 | 1.30E-18 | 8.61E-17 |  |  |  |  |
| *PLP1* | 2.305287998 | 2.16E-15 | 8.38E-14 |  |  |  |  |
| *PNCK* | 3.149621782 | 5.90E-33 | 1.22E-30 |  |  |  |  |
| *RP1-177G6.2* | 2.952571514 | 1.21E-19 | 9.43E-18 |  |  |  |  |
| *SMPX* | 3.086153686 | 8.64E-22 | 8.65E-20 |  |  |  |  |
| *SOX3* | 2.412933532 | 2.58E-07 | 1.81E-06 |  |  |  |  |
| *SRD5A1P1* | 2.113619736 | 2.76E-08 | 2.40E-07 |  |  |  |  |
| *SSX3* | 2.123447583 | 1.79E-06 | 1.03E-05 |  |  |  |  |
| *TBX22* | 2.432251496 | 7.59E-09 | 7.48E-08 |  |  |  |  |
| *TCEAL2* | 2.807569486 | 8.28E-20 | 6.61E-18 |  |  |  |  |
| *TCEAL6* | 2.483584961 | 7.00E-12 | 1.34E-10 |  |  |  |  |
| *TMEM35* | 2.012533035 | 8.22E-16 | 3.44E-14 |  |  |  |  |
| *ZCCHC13* | 2.149206303 | 2.77E-06 | 1.51E-05 |  |  |  |  |
| *ZCCHC5* | 2.531523726 | 8.79E-23 | 9.57E-21 |  |  |  |  |
| **(SNCA+SCA) vs. EA** | | | | | | | |
| **mRNAs** | **log2 Fold Change** | **p value** | **p adj** | **microRNAs** | **log2 Fold Change** | **p value** | **p adj** |
| *ANGPTL1* | 2.200307018 | 2.95E-21 | 5.83E-19 | *hsa-mir-133a-2* | 2.099963485 | 5.28E-15 | 1.86E-13 |
| *ASTN1* | 2.115138931 | 1.01E-15 | 6.25E-14 | *hsa-mir-133b* | 2.087377408 | 5.79E-18 | 5.11E-16 |
| *ATP1A2* | 2.142555478 | 1.59E-16 | 1.19E-14 | *hsa-mir-374c* | 3.169046553 | 5.58E-09 | 6.34E-08 |
| *BC039356* | 2.0049298 | 4.48E-08 | 4.11E-07 | *hsa-mir-483* | 2.375029488 | 2.09E-21 | 2.95E-19 |
| *BNIPL* | -2.253716366 | 5.00E-28 | 2.33E-25 | *hsa-mir-490* | 3.458624239 | 4.87E-28 | 3.43E-25 |
| *CHIA* | 4.285735603 | 7.06E-24 | 2.11E-21 |  |  |  |  |
| *CLCA2* | -2.079423824 | 9.96E-09 | 1.09E-07 |  |  |  |  |
| *CLCA4* | -2.918578018 | 5.72E-18 | 5.89E-16 |  |  |  |  |
| *CRCT1* | -2.823436838 | 4.38E-12 | 1.13E-10 |  |  |  |  |
| *CRNN* | -2.654396993 | 7.64E-09 | 8.57E-08 |  |  |  |  |
| *GBP6* | -2.538955066 | 1.12E-16 | 8.75E-15 |  |  |  |  |
| *GRIK3* | 2.310013967 | 3.67E-21 | 7.08E-19 |  |  |  |  |
| *HMGB4* | 2.395665602 | 1.43E-07 | 1.16E-06 |  |  |  |  |
| *HSPB7* | 2.87487785 | 1.73E-32 | 1.52E-29 |  |  |  |  |
| *IVL* | -2.406238802 | 2.95E-08 | 2.83E-07 |  |  |  |  |
| *KPRP* | -2.029546813 | 2.88E-06 | 1.66E-05 |  |  |  |  |
| *LHX8* | -2.111134753 | 1.50E-09 | 1.99E-08 |  |  |  |  |
| *LMOD1* | 2.232731202 | 5.51E-23 | 1.41E-20 |  |  |  |  |
| *LRRC7* | 2.883026262 | 4.47E-23 | 1.17E-20 |  |  |  |  |
| *OR10K2* | 2.143191726 | 1.28E-06 | 8.14E-06 |  |  |  |  |
| *PADI1* | -2.171239006 | 8.00E-13 | 2.47E-11 |  |  |  |  |
| *PGLYRP3* | -2.132873921 | 1.50E-10 | 2.59E-09 |  |  |  |  |
| *PKP1* | -2.434694194 | 3.31E-15 | 1.83E-13 |  |  |  |  |
| *PLD5* | 2.30245719 | 5.05E-13 | 1.62E-11 |  |  |  |  |
| *S100A2* | -2.075246378 | 6.72E-13 | 2.11E-11 |  |  |  |  |
| *S100A7A* | -2.468957968 | 1.92E-09 | 2.49E-08 |  |  |  |  |
| *S100A7* | -2.050063867 | 1.13E-07 | 9.35E-07 |  |  |  |  |
| *S100A8* | -2.839599715 | 7.05E-23 | 1.75E-20 |  |  |  |  |
| *S100A9* | -2.184211814 | 1.40E-15 | 8.47E-14 |  |  |  |  |
| *SPRR1A* | -2.950394449 | 4.06E-16 | 2.75E-14 |  |  |  |  |
| *SPRR1B* | -2.906831352 | 2.22E-15 | 1.28E-13 |  |  |  |  |
| *SPRR2A* | -3.155758461 | 1.01E-14 | 5.00E-13 |  |  |  |  |
| *SPRR2B* | -2.364692164 | 1.81E-07 | 1.43E-06 |  |  |  |  |
| *SPRR2C* | -2.592194182 | 2.88E-08 | 2.78E-07 |  |  |  |  |
| *SPRR2D* | -3.451889876 | 1.74E-18 | 2.00E-16 |  |  |  |  |
| *SPRR2E* | -2.268972342 | 2.74E-07 | 2.06E-06 |  |  |  |  |
| *SPRR3* | -3.10599613 | 2.81E-16 | 1.94E-14 |  |  |  |  |
| *TRNA_Pseudo.19* | 2.234618518 | 2.04E-07 | 1.59E-06 |  |  |  |  |
| *TRNA_Val.4* | 2.056019152 | 2.52E-06 | 1.48E-05 |  |  |  |  |
| *ZNF683* | 2.058933709 | 4.37E-24 | 1.36E-21 |  |  |  |  |
| *AX747408* | 2.003379377 | 8.55E-08 | 7.29E-07 |  |  |  |  |
| *AX747977* | 2.122109883 | 1.43E-20 | 2.51E-18 |  |  |  |  |
| *CALML3* | -2.27843735 | 4.38E-09 | 5.25E-08 |  |  |  |  |
| *CALML5* | -2.130737404 | 6.76E-08 | 5.93E-07 |  |  |  |  |
| *KCNMA1* | 2.498174651 | 1.78E-28 | 8.74E-26 |  |  |  |  |
| *LDB3* | 2.359414658 | 2.05E-24 | 6.50E-22 |  |  |  |  |
| *LIPK* | -2.20217005 | 1.77E-09 | 2.30E-08 |  |  |  |  |
| *MKX* | 2.236276113 | 2.23E-29 | 1.32E-26 |  |  |  |  |
| *NKX6-2* | 2.099494618 | 1.26E-08 | 1.33E-07 |  |  |  |  |
| *PNLIP* | 3.565292522 | 8.13E-18 | 8.05E-16 |  |  |  |  |
| *PSD* | 2.480418185 | 1.36E-29 | 8.63E-27 |  |  |  |  |
| *PTF1A* | 2.007064366 | 4.42E-07 | 3.16E-06 |  |  |  |  |
| *SORCS3* | 2.84651071 | 6.35E-23 | 1.61E-20 |  |  |  |  |
| *TACR2* | 3.232224544 | 1.71E-37 | 2.34E-34 |  |  |  |  |
| *AB231710* | 2.1499407 | 9.20E-11 | 1.70E-09 |  |  |  |  |
| *AK128059* | 2.51934328 | 4.65E-11 | 9.16E-10 |  |  |  |  |
| *AL833634* | 2.325784894 | 2.13E-21 | 4.34E-19 |  |  |  |  |
| *AX746604* | 2.246427598 | 2.93E-10 | 4.72E-09 |  |  |  |  |
| *BC027619* | 2.005147221 | 3.61E-06 | 2.03E-05 |  |  |  |  |
| *CHRDL2* | 2.051031023 | 1.69E-18 | 1.98E-16 |  |  |  |  |
| *FAT3* | 2.237513124 | 4.24E-23 | 1.12E-20 |  |  |  |  |
| *HEPHL1* | -3.191456164 | 3.57E-38 | 5.16E-35 |  |  |  |  |
| *KCNA4* | 2.752438804 | 1.71E-18 | 1.98E-16 |  |  |  |  |
| *LINC00301* | 2.062400532 | 1.80E-06 | 1.10E-05 |  |  |  |  |
| *LMO1* | 3.723626628 | 2.22E-31 | 1.65E-28 |  |  |  |  |
| *MRGPRF* | 2.152703004 | 1.49E-29 | 9.21E-27 |  |  |  |  |
| *OR4D11* | 2.327764213 | 1.03E-07 | 8.62E-07 |  |  |  |  |
| *OR5A1* | 2.617094965 | 4.33E-09 | 5.20E-08 |  |  |  |  |
| *OR5AP2* | 2.254319844 | 2.73E-07 | 2.05E-06 |  |  |  |  |
| *PGA3* | 3.194529706 | 1.46E-16 | 1.11E-14 |  |  |  |  |
| *PGA4* | 4.500635811 | 1.32E-32 | 1.27E-29 |  |  |  |  |
| *PGA5* | 4.035369169 | 4.81E-44 | 1.39E-40 |  |  |  |  |
| *SLC22A6* | 2.071154835 | 4.55E-08 | 4.16E-07 |  |  |  |  |
| *TAGLN* | 2.322253296 | 1.19E-32 | 1.19E-29 |  |  |  |  |
| *TRIM49B* | 2.404588819 | 3.43E-08 | 3.24E-07 |  |  |  |  |
| *TRIM64B* | 2.181133814 | 1.91E-06 | 1.16E-05 |  |  |  |  |
| *WT1-AS* | 2.389648463 | 3.27E-14 | 1.41E-12 |  |  |  |  |
| *A2ML1* | -2.657794265 | 7.17E-15 | 3.73E-13 |  |  |  |  |
| *AX748157* | 2.078733221 | 6.68E-11 | 1.28E-09 |  |  |  |  |
| *ENDOU* | -2.832708935 | 1.48E-38 | 2.41E-35 |  |  |  |  |
| *EPYC* | 2.283396219 | 9.33E-14 | 3.61E-12 |  |  |  |  |
| *KCNA1* | 4.338006518 | 8.47E-40 | 1.70E-36 |  |  |  |  |
| *KERA* | 2.330937442 | 8.91E-20 | 1.35E-17 |  |  |  |  |
| *KRT4* | -3.108127548 | 9.21E-16 | 5.79E-14 |  |  |  |  |
| *KRT5* | -2.359397822 | 6.02E-11 | 1.16E-09 |  |  |  |  |
| *KRT6A* | -3.421586657 | 1.58E-21 | 3.33E-19 |  |  |  |  |
| *KRT6B* | -2.938073239 | 3.66E-20 | 5.93E-18 |  |  |  |  |
| *KRT6C* | -4.156179348 | 2.18E-29 | 1.32E-26 |  |  |  |  |
| *KRT75* | -3.346774543 | 2.91E-18 | 3.17E-16 |  |  |  |  |
| *KRT78* | -2.857702019 | 3.83E-14 | 1.61E-12 |  |  |  |  |
| *LINC00477* | 2.064979413 | 9.25E-09 | 1.02E-07 |  |  |  |  |
| *LOC283392* | 2.154708936 | 4.65E-14 | 1.92E-12 |  |  |  |  |
| *OR6C65* | 2.213106077 | 6.83E-07 | 4.66E-06 |  |  |  |  |
| *PDZRN4* | 2.437044711 | 1.16E-20 | 2.05E-18 |  |  |  |  |
| *PPP1R1A* | 2.289389304 | 1.34E-19 | 1.96E-17 |  |  |  |  |
| *PRPH* | 2.111579773 | 4.85E-19 | 6.57E-17 |  |  |  |  |
| *SDR9C7* | -2.980791728 | 1.62E-15 | 9.59E-14 |  |  |  |  |
| *SYT10* | 2.397168056 | 2.90E-14 | 1.28E-12 |  |  |  |  |
| *WSCD2* | 2.047136109 | 3.10E-16 | 2.14E-14 |  |  |  |  |
| *Y_RNA.18* | 2.255424573 | 2.45E-08 | 2.42E-07 |  |  |  |  |
| *FAM123A* | 3.082793217 | 3.87E-16 | 2.62E-14 |  |  |  |  |
| *FAM194B* | 2.119458905 | 3.56E-07 | 2.60E-06 |  |  |  |  |
| *GJB6* | -2.106464506 | 1.15E-11 | 2.65E-10 |  |  |  |  |
| *MLNR* | 2.333218851 | 2.30E-16 | 1.64E-14 |  |  |  |  |
| *RXFP2* | 2.072289924 | 1.54E-07 | 1.23E-06 |  |  |  |  |
| *SERTM1* | 2.595368313 | 9.48E-13 | 2.88E-11 |  |  |  |  |
| *SLITRK5* | 2.500093246 | 4.40E-20 | 6.94E-18 |  |  |  |  |
| *BX247990* | 2.030579404 | 3.06E-10 | 4.90E-09 |  |  |  |  |
| *C14orf23* | 2.021257605 | 4.17E-07 | 3.00E-06 |  |  |  |  |
| *CMTM5* | 2.06950374 | 1.26E-14 | 6.06E-13 |  |  |  |  |
| *DLK1* | 4.23340989 | 2.54E-38 | 3.89E-35 |  |  |  |  |
| *NRXN3* | 2.068316313 | 1.30E-20 | 2.29E-18 |  |  |  |  |
| *OR4K2* | 2.124768128 | 7.62E-07 | 5.14E-06 |  |  |  |  |
| *PRIMA1* | 2.065377784 | 1.21E-17 | 1.15E-15 |  |  |  |  |
| *RNASE7* | -2.272736732 | 2.06E-11 | 4.45E-10 |  |  |  |  |
| *TGM1* | -3.099916525 | 8.61E-29 | 4.40E-26 |  |  |  |  |
| *CERS3* | -2.49808471 | 1.30E-13 | 4.87E-12 |  |  |  |  |
| *CHRNA3* | 2.394357594 | 7.29E-29 | 3.88E-26 |  |  |  |  |
| *CYP1A2* | 2.107002103 | 7.51E-10 | 1.08E-08 |  |  |  |  |
| *GABRA5* | 2.059442627 | 8.46E-11 | 1.58E-09 |  |  |  |  |
| *LOC283738* | 2.072606511 | 9.99E-07 | 6.54E-06 |  |  |  |  |
| *LOC390660.1* | 2.154951193 | 1.48E-08 | 1.54E-07 |  |  |  |  |
| *PWRN2* | 2.45037254 | 1.71E-09 | 2.24E-08 |  |  |  |  |
| *RHCG* | -2.905166451 | 5.54E-17 | 4.64E-15 |  |  |  |  |
| *SYNM* | 2.697473788 | 6.70E-27 | 2.77E-24 |  |  |  |  |
| *AX747287* | 2.033464088 | 3.90E-06 | 2.18E-05 |  |  |  |  |
| *BC043527* | 2.171404193 | 5.26E-09 | 6.19E-08 |  |  |  |  |
| *CAPNS2* | -2.70937174 | 8.00E-17 | 6.42E-15 |  |  |  |  |
| *CBLN1* | 2.221553461 | 1.91E-16 | 1.42E-14 |  |  |  |  |
| *CTRB1* | 3.234677161 | 3.52E-16 | 2.39E-14 |  |  |  |  |
| *CTRB2* | 3.154006159 | 2.49E-15 | 1.41E-13 |  |  |  |  |
| *DKFZP434H168* | 2.067866903 | 1.28E-08 | 1.35E-07 |  |  |  |  |
| *GNAO1* | 2.32742916 | 8.26E-30 | 5.38E-27 |  |  |  |  |
| *HP* | 2.347768458 | 3.85E-21 | 7.37E-19 |  |  |  |  |
| *KCNG4* | 2.07424356 | 1.46E-06 | 9.14E-06 |  |  |  |  |
| *LOC100506172* | 2.480805911 | 9.90E-11 | 1.81E-09 |  |  |  |  |
| *LOC283856* | 2.080481394 | 1.94E-18 | 2.22E-16 |  |  |  |  |
| *MYH11* | 2.703630886 | 1.00E-25 | 3.68E-23 |  |  |  |  |
| *PDILT* | 2.096925601 | 3.72E-08 | 3.49E-07 |  |  |  |  |
| *PRSS27* | -2.798976926 | 1.75E-32 | 1.52E-29 |  |  |  |  |
| *GAST* | 2.400103361 | 1.50E-13 | 5.54E-12 |  |  |  |  |
| *GIP* | 2.317719799 | 8.50E-13 | 2.62E-11 |  |  |  |  |
| *GLP2R* | 2.489952796 | 2.70E-29 | 1.56E-26 |  |  |  |  |
| *KRT13* | -2.23288352 | 7.89E-09 | 8.80E-08 |  |  |  |  |
| *KRT14* | -3.077624613 | 3.07E-17 | 2.67E-15 |  |  |  |  |
| *KRT16* | -3.190654443 | 1.41E-26 | 5.48E-24 |  |  |  |  |
| *KRT24* | -2.240412788 | 2.81E-08 | 2.72E-07 |  |  |  |  |
| *MYH2* | 2.075803365 | 4.83E-13 | 1.56E-11 |  |  |  |  |
| *NPTX1* | 3.074486364 | 1.37E-32 | 1.28E-29 |  |  |  |  |
| *NXPH3* | 2.067755503 | 1.66E-23 | 4.76E-21 |  |  |  |  |
| *OTOP2* | -2.248820125 | 1.30E-13 | 4.87E-12 |  |  |  |  |
| *PIRT* | 2.15301354 | 5.56E-18 | 5.75E-16 |  |  |  |  |
| *RNF222* | -2.926501458 | 4.73E-26 | 1.76E-23 |  |  |  |  |
| *SLC35G3* | 2.435517668 | 5.38E-12 | 1.35E-10 |  |  |  |  |
| *TUSC5* | 2.631608474 | 3.94E-17 | 3.40E-15 |  |  |  |  |
| *ZNF750* | -2.278939873 | 1.27E-16 | 9.79E-15 |  |  |  |  |
| *BC037384* | 2.006115505 | 7.94E-06 | 4.08E-05 |  |  |  |  |
| *DSC3* | -2.152245916 | 2.98E-13 | 1.03E-11 |  |  |  |  |
| *DSG1* | -2.358650983 | 5.91E-19 | 7.66E-17 |  |  |  |  |
| *SERPINB13* | -3.012468061 | 1.54E-12 | 4.48E-11 |  |  |  |  |
| *SERPINB3* | -2.118110303 | 1.32E-09 | 1.79E-08 |  |  |  |  |
| *SYT4* | 2.43169535 | 4.61E-16 | 3.07E-14 |  |  |  |  |
| *BSPH1* | 2.611244197 | 3.89E-10 | 6.07E-09 |  |  |  |  |
| *CABP5* | 2.071267908 | 7.42E-08 | 6.44E-07 |  |  |  |  |
| *CCDC105* | 2.019292362 | 1.21E-06 | 7.73E-06 |  |  |  |  |
| *CLC* | 3.063560998 | 1.16E-26 | 4.70E-24 |  |  |  |  |
| *CNFN* | -2.803098876 | 4.96E-30 | 3.31E-27 |  |  |  |  |
| *CNN1* | 3.514045192 | 4.07E-44 | 1.33E-40 |  |  |  |  |
| *CRX* | 2.259164366 | 1.65E-10 | 2.82E-09 |  |  |  |  |
| *FLJ25758* | 2.019777335 | 3.50E-08 | 3.30E-07 |  |  |  |  |
| *HSPB6* | 3.323337414 | 4.37E-39 | 7.60E-36 |  |  |  |  |
| *IGFL2* | 2.112293231 | 1.26E-19 | 1.86E-17 |  |  |  |  |
| *KLK12* | -2.73061154 | 4.25E-21 | 8.02E-19 |  |  |  |  |
| *KLK13* | -2.859737521 | 6.24E-21 | 1.14E-18 |  |  |  |  |
| *KLK5* | -2.02156498 | 1.72E-07 | 1.36E-06 |  |  |  |  |
| *KRTDAP* | -2.500512215 | 2.33E-11 | 4.96E-10 |  |  |  |  |
| *LYPD3* | -2.444727016 | 2.04E-24 | 6.50E-22 |  |  |  |  |
| *LYPD4* | 2.091410982 | 2.63E-10 | 4.29E-09 |  |  |  |  |
| *MBD3L1* | 2.193971593 | 8.60E-07 | 5.72E-06 |  |  |  |  |
| *MIR371A* | 2.550594597 | 5.94E-10 | 8.77E-09 |  |  |  |  |
| *NCCRP1* | -2.551571402 | 4.32E-18 | 4.50E-16 |  |  |  |  |
| *NLRP13* | 2.532407162 | 8.42E-10 | 1.19E-08 |  |  |  |  |
| *OR7A17* | 2.095148023 | 4.53E-06 | 2.48E-05 |  |  |  |  |
| *OR7D4* | 2.394260517 | 1.15E-07 | 9.50E-07 |  |  |  |  |
| *OR7E24* | 2.073740755 | 4.26E-06 | 2.36E-05 |  |  |  |  |
| *OR7G3* | 2.612420196 | 1.12E-09 | 1.54E-08 |  |  |  |  |
| *PLIN4* | 2.426919427 | 3.01E-23 | 8.16E-21 |  |  |  |  |
| *SBSN* | -3.77902475 | 1.42E-25 | 5.15E-23 |  |  |  |  |
| *USP29* | 2.272125191 | 2.68E-08 | 2.61E-07 |  |  |  |  |
| *ZIM3* | 2.683142128 | 1.03E-10 | 1.88E-09 |  |  |  |  |
| *ACTG2* | 3.50236302 | 3.68E-42 | 8.72E-39 |  |  |  |  |
| *BC048132* | 2.125659657 | 1.39E-10 | 2.42E-09 |  |  |  |  |
| *C2orf40* | 3.190035387 | 1.97E-30 | 1.39E-27 |  |  |  |  |
| *C2orf53* | 2.109607122 | 2.98E-09 | 3.69E-08 |  |  |  |  |
| *CAPN14* | -2.121146861 | 4.66E-12 | 1.19E-10 |  |  |  |  |
| *DES* | 3.545991287 | 3.28E-34 | 3.72E-31 |  |  |  |  |
| *DPYSL5* | 2.472919626 | 5.95E-18 | 6.09E-16 |  |  |  |  |
| *GKN1* | 3.803027548 | 5.01E-25 | 1.74E-22 |  |  |  |  |
| *GKN2* | 3.428875045 | 1.43E-21 | 3.09E-19 |  |  |  |  |
| *IL36A* | -2.74161093 | 1.04E-09 | 1.45E-08 |  |  |  |  |
| *IL36G* | -3.076073671 | 5.58E-21 | 1.04E-18 |  |  |  |  |
| *IL36RN* | -3.55918183 | 1.57E-21 | 3.32E-19 |  |  |  |  |
| *LOC100505964* | 2.501898057 | 1.73E-09 | 2.26E-08 |  |  |  |  |
| *MAL* | -2.517414333 | 4.98E-19 | 6.61E-17 |  |  |  |  |
| *MYADML* | 2.255422644 | 3.79E-13 | 1.26E-11 |  |  |  |  |
| *MYT1L* | 2.29439839 | 4.29E-14 | 1.78E-12 |  |  |  |  |
| *NRXN1* | 2.068936874 | 2.42E-15 | 1.38E-13 |  |  |  |  |
| *NXPH2* | 2.785569054 | 3.13E-16 | 2.15E-14 |  |  |  |  |
| *SLC5A7* | 2.299969873 | 1.54E-15 | 9.21E-14 |  |  |  |  |
| *SMYD1* | 4.957276883 | 4.09E-46 | 1.77E-42 |  |  |  |  |
| *SPHKAP* | 2.29374049 | 2.01E-12 | 5.63E-11 |  |  |  |  |
| *TCF23* | 2.649350266 | 1.28E-16 | 9.82E-15 |  |  |  |  |
| *TISP43.1* | 2.026321852 | 4.58E-06 | 2.51E-05 |  |  |  |  |
| *XIRP2* | 2.072647362 | 6.59E-10 | 9.63E-09 |  |  |  |  |
| *BPIFA3* | 2.412326387 | 1.10E-07 | 9.14E-07 |  |  |  |  |
| *C20orf166-AS1* | 2.418048571 | 1.97E-20 | 3.38E-18 |  |  |  |  |
| *C20orf166* | 2.56716235 | 6.03E-13 | 1.90E-11 |  |  |  |  |
| *DEFB118* | 2.547001644 | 9.66E-12 | 2.28E-10 |  |  |  |  |
| *DEFB122.1* | 2.400858299 | 3.06E-08 | 2.93E-07 |  |  |  |  |
| *DEFB122.2* | 2.322641408 | 6.09E-08 | 5.40E-07 |  |  |  |  |
| *FAM83C* | -2.548234078 | 9.49E-14 | 3.65E-12 |  |  |  |  |
| *LOC100287792* | 2.104147206 | 5.98E-09 | 6.92E-08 |  |  |  |  |
| *MYL9* | 2.327124783 | 3.18E-32 | 2.59E-29 |  |  |  |  |
| *Metazoa_SRP.60* | 2.028199349 | 2.40E-06 | 1.42E-05 |  |  |  |  |
| *PRNT* | 2.004756373 | 1.05E-06 | 6.83E-06 |  |  |  |  |
| *SEL1L2* | 2.365576464 | 1.55E-17 | 1.45E-15 |  |  |  |  |
| *SUN5* | 2.231013212 | 2.45E-07 | 1.86E-06 |  |  |  |  |
| *TGM3* | -3.738022404 | 3.32E-42 | 8.64E-39 |  |  |  |  |
| *WFDC12* | -2.000451521 | 1.42E-12 | 4.17E-11 |  |  |  |  |
| *WFDC5* | -2.847712499 | 1.62E-17 | 1.50E-15 |  |  |  |  |
| *ANKRD30BP2* | 2.014765786 | 1.51E-09 | 2.01E-08 |  |  |  |  |
| *C21orf54* | 2.198665914 | 2.18E-07 | 1.69E-06 |  |  |  |  |
| *AK123632* | 2.083902278 | 2.06E-06 | 1.24E-05 |  |  |  |  |
| *CACNG2* | 2.910893596 | 1.04E-16 | 8.15E-15 |  |  |  |  |
| *CCT8L2* | 2.393393535 | 5.99E-09 | 6.93E-08 |  |  |  |  |
| *KIAA1644* | 2.065256253 | 1.11E-23 | 3.24E-21 |  |  |  |  |
| *SEZ6L* | 2.523129811 | 1.29E-31 | 9.87E-29 |  |  |  |  |
| *VPREB3* | 2.022461265 | 8.64E-19 | 1.09E-16 |  |  |  |  |
| *ADIPOQ* | 2.687177656 | 2.00E-12 | 5.62E-11 |  |  |  |  |
| *AGTR1* | 2.38963542 | 2.57E-20 | 4.33E-18 |  |  |  |  |
| *ARPP21.1* | 2.755330004 | 7.58E-15 | 3.91E-13 |  |  |  |  |
| *C3orf77* | 2.272597938 | 4.08E-09 | 4.92E-08 |  |  |  |  |
| *CPB1* | 2.193596753 | 9.17E-13 | 2.80E-11 |  |  |  |  |
| *CSTA* | -2.537200591 | 3.73E-27 | 1.59E-24 |  |  |  |  |
| *FAM19A4* | 3.511885449 | 1.67E-27 | 7.38E-25 |  |  |  |  |
| *IQCF2* | 2.120325094 | 3.16E-06 | 1.80E-05 |  |  |  |  |
| *LOC285375* | 2.183229039 | 6.90E-07 | 4.70E-06 |  |  |  |  |
| *MYLK* | 2.165910319 | 1.37E-26 | 5.42E-24 |  |  |  |  |
| *POPDC2* | 2.069426236 | 9.00E-29 | 4.51E-26 |  |  |  |  |
| *SLC7A14* | 3.3049247 | 1.13E-31 | 8.91E-29 |  |  |  |  |
| *VENTXP7* | 2.013630253 | 5.49E-08 | 4.92E-07 |  |  |  |  |
| *ADH1A* | 2.108373764 | 7.47E-18 | 7.51E-16 |  |  |  |  |
| *AFP* | 3.560700431 | 5.14E-37 | 6.69E-34 |  |  |  |  |
| *AK093205* | 2.125810224 | 4.54E-07 | 3.24E-06 |  |  |  |  |
| *ALB* | 3.289002697 | 1.97E-35 | 2.34E-32 |  |  |  |  |
| *ASB5* | 2.232143007 | 3.81E-10 | 5.97E-09 |  |  |  |  |
| *BC025350* | 2.264175248 | 2.10E-07 | 1.63E-06 |  |  |  |  |
| *BC034799* | 2.116382552 | 4.27E-06 | 2.36E-05 |  |  |  |  |
| *BC040219* | 2.389999949 | 4.25E-12 | 1.10E-10 |  |  |  |  |
| *BC041818* | 2.001052962 | 1.99E-06 | 1.20E-05 |  |  |  |  |
| *BC042378* | 2.012559129 | 8.96E-08 | 7.58E-07 |  |  |  |  |
| *BMP3* | 2.964735423 | 2.33E-23 | 6.45E-21 |  |  |  |  |
| *CCKAR* | 3.177991963 | 6.66E-21 | 1.20E-18 |  |  |  |  |
| *DEFB131* | 2.111728161 | 1.94E-06 | 1.18E-05 |  |  |  |  |
| *EPGN* | -2.627266081 | 5.82E-14 | 2.37E-12 |  |  |  |  |
| *HAND2* | 4.930986822 | 5.98E-72 | 1.56E-67 |  |  |  |  |
| *KCTD8* | 2.39596809 | 1.93E-15 | 1.14E-13 |  |  |  |  |
| *LOC285547* | 3.051452149 | 4.95E-13 | 1.59E-11 |  |  |  |  |
| *NBLA00301* | 4.462954224 | 1.84E-55 | 2.39E-51 |  |  |  |  |
| *NKX3-2* | 3.685611758 | 1.88E-53 | 1.63E-49 |  |  |  |  |
| *PCDH10* | 2.61146682 | 6.86E-20 | 1.06E-17 |  |  |  |  |
| *PHOX2B* | 4.435511623 | 5.14E-33 | 5.58E-30 |  |  |  |  |
| *SCRG1* | 3.56582052 | 1.13E-40 | 2.45E-37 |  |  |  |  |
| *SYNPO2* | 2.723361337 | 7.74E-29 | 4.03E-26 |  |  |  |  |
| *TMPRSS11A* | -2.218858936 | 2.94E-07 | 2.19E-06 |  |  |  |  |
| *TMPRSS11D* | -3.004645296 | 7.01E-16 | 4.53E-14 |  |  |  |  |
| *TMPRSS11E* | -2.496280941 | 2.77E-11 | 5.84E-10 |  |  |  |  |
| *TMPRSS11F* | -2.211457803 | 1.79E-07 | 1.41E-06 |  |  |  |  |
| *TRIM60* | 2.005375014 | 4.34E-06 | 2.40E-05 |  |  |  |  |
| *AK000840* | 2.581460178 | 1.72E-09 | 2.25E-08 |  |  |  |  |
| *BC011998* | 2.157254134 | 3.01E-09 | 3.74E-08 |  |  |  |  |
| *C7* | 2.116485654 | 6.51E-16 | 4.22E-14 |  |  |  |  |
| *CARTPT* | 5.138666805 | 1.21E-44 | 4.50E-41 |  |  |  |  |
| *CDH10* | 2.203447052 | 4.02E-13 | 1.33E-11 |  |  |  |  |
| *CTNND2* | 2.276592328 | 4.38E-19 | 6.00E-17 |  |  |  |  |
| *FAT2* | -2.106479961 | 4.38E-15 | 2.37E-13 |  |  |  |  |
| *FBXL21* | 2.405515539 | 5.23E-10 | 7.87E-09 |  |  |  |  |
| *GABRA1* | 2.114464174 | 1.10E-08 | 1.18E-07 |  |  |  |  |
| *HMP19* | 2.417021379 | 8.78E-22 | 1.94E-19 |  |  |  |  |
| *KCNMB1* | 2.046987083 | 6.63E-24 | 2.03E-21 |  |  |  |  |
| *LOC340107* | 2.012118477 | 1.41E-05 | 6.83E-05 |  |  |  |  |
| *NKX2-5* | 2.533294432 | 1.39E-13 | 5.17E-12 |  |  |  |  |
| *SLC36A2* | 2.038434871 | 9.80E-11 | 1.79E-09 |  |  |  |  |
| *SPINK5* | -2.49608425 | 4.36E-21 | 8.17E-19 |  |  |  |  |
| *SPINK6* | -2.244003879 | 1.04E-12 | 3.16E-11 |  |  |  |  |
| *SPINK7* | -2.29028935 | 1.14E-10 | 2.04E-09 |  |  |  |  |
| *AF086258* | 2.148767343 | 5.48E-18 | 5.69E-16 |  |  |  |  |
| *CLVS2* | 2.450152846 | 2.39E-10 | 3.96E-09 |  |  |  |  |
| *HMGA1P7* | 2.073189331 | 1.16E-07 | 9.60E-07 |  |  |  |  |
| *KHDRBS2* | 2.372808314 | 4.72E-20 | 7.41E-18 |  |  |  |  |
| *METTL24* | 2.112718704 | 5.41E-27 | 2.27E-24 |  |  |  |  |
| *MLN* | 2.089292953 | 3.65E-08 | 3.43E-07 |  |  |  |  |
| *MUC21* | -2.434679814 | 1.74E-09 | 2.27E-08 |  |  |  |  |
| *MUC22* | -2.878350458 | 2.42E-15 | 1.38E-13 |  |  |  |  |
| *NRSN1* | 2.343927243 | 7.82E-15 | 3.99E-13 |  |  |  |  |
| *OR11A1* | 2.187599883 | 1.58E-07 | 1.26E-06 |  |  |  |  |
| *PLN* | 2.179250542 | 1.31E-21 | 2.86E-19 |  |  |  |  |
| *RAET1E* | -2.258695709 | 2.54E-30 | 1.74E-27 |  |  |  |  |
| *SNAP91* | 2.078711246 | 9.12E-15 | 4.56E-13 |  |  |  |  |
| *TAAR5* | 2.027522414 | 1.34E-06 | 8.51E-06 |  |  |  |  |
| *TRNA_Arg.15* | 2.374130545 | 1.17E-07 | 9.65E-07 |  |  |  |  |
| *VIP* | 3.496801171 | 9.44E-40 | 1.76E-36 |  |  |  |  |
| *ADCYAP1R1* | 2.077162707 | 3.31E-19 | 4.63E-17 |  |  |  |  |
| *C7orf66* | 2.198202601 | 6.33E-08 | 5.59E-07 |  |  |  |  |
| *C7orf72* | 2.019741997 | 6.33E-07 | 4.35E-06 |  |  |  |  |
| *CHRM2* | 3.614635435 | 2.96E-31 | 2.14E-28 |  |  |  |  |
| *CPA1* | 2.742911544 | 2.21E-17 | 2.01E-15 |  |  |  |  |
| *DPP6* | 3.432883599 | 1.54E-35 | 1.91E-32 |  |  |  |  |
| *FLNC* | 2.43293506 | 3.16E-25 | 1.11E-22 |  |  |  |  |
| *HTR5A* | 2.206383636 | 2.43E-07 | 1.85E-06 |  |  |  |  |
| *Metazoa_SRP.96* | 2.378253024 | 1.35E-08 | 1.42E-07 |  |  |  |  |
| *NPY* | 2.092348873 | 4.66E-12 | 1.19E-10 |  |  |  |  |
| *OR6W1P* | 2.099629068 | 1.21E-08 | 1.28E-07 |  |  |  |  |
| *PRSS1* | 2.130248374 | 7.06E-13 | 2.20E-11 |  |  |  |  |
| *PRSS3P2* | 2.131196674 | 9.05E-12 | 2.16E-10 |  |  |  |  |
| *SEPT14* | 2.289974709 | 4.63E-10 | 7.09E-09 |  |  |  |  |
| *TAC1* | 4.018602709 | 1.98E-32 | 1.66E-29 |  |  |  |  |
| *ADAM7* | 2.036777593 | 4.39E-07 | 3.15E-06 |  |  |  |  |
| *ADRB3* | 2.65087309 | 8.42E-23 | 2.02E-20 |  |  |  |  |
| *BC042029* | 2.118659308 | 1.60E-06 | 9.92E-06 |  |  |  |  |
| *C8orf87* | 2.099766713 | 1.66E-07 | 1.32E-06 |  |  |  |  |
| *CCDC26* | 2.056813682 | 5.77E-11 | 1.12E-09 |  |  |  |  |
| *LOC100127983* | 2.04543059 | 6.36E-25 | 2.15E-22 |  |  |  |  |
| *LOC100192378* | 2.78724303 | 1.60E-27 | 7.19E-25 |  |  |  |  |
| *LOC286094* | 2.291941468 | 9.13E-08 | 7.72E-07 |  |  |  |  |
| *NKX6-3* | 2.283606706 | 1.71E-12 | 4.89E-11 |  |  |  |  |
| *SLURP1* | -2.769180801 | 7.49E-14 | 2.96E-12 |  |  |  |  |
| *SPAG11B.1* | 2.398472123 | 3.17E-08 | 3.02E-07 |  |  |  |  |
| *STMN2* | 2.580308322 | 3.57E-26 | 1.35E-23 |  |  |  |  |
| *AK093363* | 2.019098113 | 5.90E-07 | 4.08E-06 |  |  |  |  |
| *AK130904* | 2.031599823 | 4.70E-17 | 3.96E-15 |  |  |  |  |
| *AL390170* | 2.583180487 | 2.14E-19 | 3.08E-17 |  |  |  |  |
| *AX747119.2* | 2.013606377 | 9.96E-09 | 1.09E-07 |  |  |  |  |
| *BARX1* | 3.234571418 | 3.57E-46 | 1.77E-42 |  |  |  |  |
| *BC035187* | 2.32205434 | 5.55E-09 | 6.47E-08 |  |  |  |  |
| *FOXE1* | -2.261802769 | 5.42E-10 | 8.10E-09 |  |  |  |  |
| *FRRS1L* | 3.206367215 | 3.42E-27 | 1.49E-24 |  |  |  |  |
| *GLT6D1* | 2.264638047 | 5.94E-08 | 5.28E-07 |  |  |  |  |
| *LOC340515* | 2.052539201 | 8.99E-10 | 1.27E-08 |  |  |  |  |
| *LOC572558* | 2.656541691 | 1.57E-18 | 1.84E-16 |  |  |  |  |
| *MIR101-2* | 2.114353391 | 8.50E-07 | 5.66E-06 |  |  |  |  |
| *MORN5* | 3.362365407 | 2.11E-28 | 1.02E-25 |  |  |  |  |
| *PGM5* | 2.281302302 | 3.18E-23 | 8.54E-21 |  |  |  |  |
| *RMRP* | 2.735562381 | 1.80E-46 | 1.17E-42 |  |  |  |  |
| *FLJ44838* | 2.854844405 | 1.15E-12 | 3.44E-11 |  |  |  |  |
| *AGTR2* | 2.077214916 | 8.72E-08 | 7.41E-07 |  |  |  |  |
| *AK056105* | 2.37724686 | 9.45E-12 | 2.24E-10 |  |  |  |  |
| *CNKSR2* | 2.074341801 | 6.20E-18 | 6.31E-16 |  |  |  |  |
| *CTAG2* | 2.185515943 | 5.66E-07 | 3.94E-06 |  |  |  |  |
| *DCX* | 2.043971445 | 2.28E-15 | 1.32E-13 |  |  |  |  |
| *DKFZp686D0853* | 2.025390901 | 2.88E-18 | 3.15E-16 |  |  |  |  |
| *FAM48B2* | 2.251941767 | 3.77E-10 | 5.91E-09 |  |  |  |  |
| *FHL1* | 2.016782198 | 3.49E-23 | 9.28E-21 |  |  |  |  |
| *PLP1* | 2.312279271 | 2.36E-17 | 2.11E-15 |  |  |  |  |
| *PNCK* | 2.847261709 | 3.00E-29 | 1.70E-26 |  |  |  |  |
| *RP1-177G6.2* | 2.790087436 | 3.91E-18 | 4.16E-16 |  |  |  |  |
| *SMPX* | 2.77687236 | 1.14E-18 | 1.39E-16 |  |  |  |  |
| *SOX3* | 2.307140893 | 1.50E-07 | 1.21E-06 |  |  |  |  |
| *SRD5A1P1* | 2.005187103 | 3.95E-08 | 3.68E-07 |  |  |  |  |
| *SSX3* | 2.026926649 | 1.65E-06 | 1.02E-05 |  |  |  |  |
| *TBX22* | 2.447658926 | 6.48E-10 | 9.48E-09 |  |  |  |  |
| *TCEAL2* | 2.993986118 | 1.65E-24 | 5.43E-22 |  |  |  |  |
| *TCEAL6* | 2.442372548 | 3.09E-12 | 8.31E-11 |  |  |  |  |
| *ZCCHC13* | 2.048123831 | 2.18E-06 | 1.30E-05 |  |  |  |  |
| *ZCCHC5* | 2.335146464 | 2.61E-20 | 4.35E-18 |  |  |  |  |
| **(SCA+EA) vs. SNCA** | | | | | | | |
| **mRNAs** | **log2 Fold Change** | **p value** | **p adj** | **microRNAs** | **log2 Fold Change** | **p value** | **p adj** |
| *BNIPL* | 2.821425792 | 3.44E-70 | 1.99E-67 | *hsa-mir-205* | 5.086762013 | 1.10E-81 | 7.27E-79 |
| *C1orf177* | 2.108949181 | 2.16E-42 | 5.92E-40 | *hsa-mir-944* | 2.816950149 | 7.94E-53 | 2.62E-50 |
| *CLCA2* | 6.916189641 | 9.15E-183 | 2.38E-178 |  |  |  |  |
| *CLCA4* | 5.814626075 | 4.19E-136 | 1.56E-132 |  |  |  |  |
| *CRCT1* | 5.349402719 | 2.93E-75 | 2.18E-72 |  |  |  |  |
| *CRNN* | 5.667598816 | 3.01E-63 | 1.48E-60 |  |  |  |  |
| *GBP6* | 4.784334481 | 2.69E-122 | 5.83E-119 |  |  |  |  |
| *GRHL3* | 2.348016816 | 7.12E-45 | 2.23E-42 |  |  |  |  |
| *HSPA6* | 2.022743904 | 1.51E-42 | 4.36E-40 |  |  |  |  |
| *IVL* | 5.50796906 | 2.11E-68 | 1.15E-65 |  |  |  |  |
| *KPRP* | 2.36306309 | 6.28E-13 | 2.99E-11 |  |  |  |  |
| *LCE3D* | 2.597240859 | 7.49E-16 | 5.58E-14 |  |  |  |  |
| *LCE3E* | 2.478837365 | 5.14E-13 | 2.48E-11 |  |  |  |  |
| *MIR205HG* | 3.168405159 | 2.06E-22 | 2.64E-20 |  |  |  |  |
| *PADI1* | 3.129064509 | 1.35E-41 | 3.68E-39 |  |  |  |  |
| *PGLYRP3* | 3.283597214 | 6.61E-39 | 1.67E-36 |  |  |  |  |
| *PKP1* | 3.214535522 | 8.78E-44 | 2.69E-41 |  |  |  |  |
| *S100A12* | 2.258352944 | 2.86E-33 | 5.77E-31 |  |  |  |  |
| *S100A2* | 3.787343501 | 8.01E-77 | 6.14E-74 |  |  |  |  |
| *S100A7A* | 3.157114923 | 1.24E-24 | 1.78E-22 |  |  |  |  |
| *S100A7* | 3.629514604 | 5.20E-36 | 1.14E-33 |  |  |  |  |
| *S100A8* | 4.947113703 | 6.26E-136 | 2.04E-132 |  |  |  |  |
| *S100A9* | 3.769313691 | 7.54E-85 | 7.28E-82 |  |  |  |  |
| *SPRR1A* | 3.992640491 | 4.71E-47 | 1.53E-44 |  |  |  |  |
| *SPRR1B* | 4.029865358 | 2.28E-48 | 7.72E-46 |  |  |  |  |
| *SPRR2A* | 4.963215227 | 1.37E-63 | 6.85E-61 |  |  |  |  |
| *SPRR2B* | 3.858237168 | 2.25E-31 | 4.38E-29 |  |  |  |  |
| *SPRR2C* | 3.862616621 | 7.67E-28 | 1.34E-25 |  |  |  |  |
| *SPRR2D* | 4.945269695 | 2.24E-68 | 1.19E-65 |  |  |  |  |
| *SPRR2E* | 5.046978947 | 7.25E-55 | 2.82E-52 |  |  |  |  |
| *SPRR2F* | 4.668316049 | 7.14E-58 | 2.95E-55 |  |  |  |  |
| *SPRR2G* | 2.101288844 | 7.67E-12 | 3.04E-10 |  |  |  |  |
| *SPRR3* | 4.735132369 | 9.45E-64 | 4.82E-61 |  |  |  |  |
| *AK128534* | 2.507968781 | 3.25E-31 | 6.22E-29 |  |  |  |  |
| *BC051760* | 2.676286467 | 1.68E-27 | 2.82E-25 |  |  |  |  |
| *CALML3* | 5.375916469 | 2.29E-83 | 2.13E-80 |  |  |  |  |
| *FAM25A* | 2.52705822 | 1.16E-21 | 1.41E-19 |  |  |  |  |
| *LDB3* | -2.274626211 | 4.52E-38 | 1.11E-35 |  |  |  |  |
| *LIPK* | 2.533515025 | 1.57E-19 | 1.67E-17 |  |  |  |  |
| *PNLIPRP3* | 2.864923575 | 1.49E-19 | 1.60E-17 |  |  |  |  |
| *PSD* | -2.093838082 | 2.42E-34 | 4.96E-32 |  |  |  |  |
| *AB231710* | -2.025570615 | 9.11E-15 | 5.85E-13 |  |  |  |  |
| *BBOX1* | 2.449955827 | 1.07E-49 | 3.76E-47 |  |  |  |  |
| *HEPHL1* | 3.142594232 | 1.14E-59 | 4.94E-57 |  |  |  |  |
| *LMO1* | -2.503850035 | 1.52E-23 | 2.08E-21 |  |  |  |  |
| *MUC15* | 2.01878599 | 6.23E-13 | 2.98E-11 |  |  |  |  |
| *PGA5* | -3.680378341 | 2.88E-77 | 2.27E-74 |  |  |  |  |
| *SCGB1A1* | 2.369470198 | 1.36E-18 | 1.31E-16 |  |  |  |  |
| *A2ML1* | 4.401708351 | 3.18E-69 | 1.80E-66 |  |  |  |  |
| *ENDOU* | 3.186073022 | 9.04E-74 | 6.54E-71 |  |  |  |  |
| *GYS2* | 2.041254503 | 9.86E-25 | 1.43E-22 |  |  |  |  |
| *KCNA1* | -3.671834811 | 1.33E-46 | 4.27E-44 |  |  |  |  |
| *KERA* | -2.094661375 | 5.34E-26 | 8.18E-24 |  |  |  |  |
| *KRT4* | 6.419530018 | 8.22E-127 | 2.14E-123 |  |  |  |  |
| *KRT5* | 5.637499566 | 1.99E-106 | 2.47E-103 |  |  |  |  |
| *KRT6A* | 5.672372257 | 3.36E-104 | 3.98E-101 |  |  |  |  |
| *KRT6B* | 3.274617156 | 1.75E-42 | 4.90E-40 |  |  |  |  |
| *KRT6C* | 5.971652174 | 1.04E-112 | 1.56E-109 |  |  |  |  |
| *KRT75* | 2.882494715 | 2.69E-22 | 3.40E-20 |  |  |  |  |
| *KRT78* | 5.761535211 | 1.08E-112 | 1.56E-109 |  |  |  |  |
| *LOC255480* | 2.869242921 | 7.75E-41 | 2.04E-38 |  |  |  |  |
| *SDR9C7* | 3.666728546 | 2.90E-41 | 7.70E-39 |  |  |  |  |
| *ATP12A* | 2.46853653 | 9.87E-26 | 1.49E-23 |  |  |  |  |
| *GJB6* | 4.050813634 | 5.06E-72 | 3.22E-69 |  |  |  |  |
| *SCEL* | 3.226434339 | 3.63E-43 | 1.09E-40 |  |  |  |  |
| *PAX9* | 2.509713426 | 4.18E-49 | 1.43E-46 |  |  |  |  |
| *RNASE7* | 3.761576223 | 1.61E-54 | 6.06E-52 |  |  |  |  |
| *SLC39A2* | 2.838517907 | 5.75E-31 | 1.08E-28 |  |  |  |  |
| *TGM1* | 5.198806832 | 4.92E-172 | 6.40E-168 |  |  |  |  |
| *BNC1* | 3.564533852 | 5.73E-59 | 2.45E-56 |  |  |  |  |
| *CERS3* | 5.145334436 | 3.33E-110 | 4.33E-107 |  |  |  |  |
| *CHRNA3* | -2.043948161 | 1.13E-34 | 2.34E-32 |  |  |  |  |
| *DUOX1* | 2.104580083 | 1.09E-43 | 3.29E-41 |  |  |  |  |
| *PLA2G4E* | 2.277480381 | 1.85E-33 | 3.76E-31 |  |  |  |  |
| *RHCG* | 6.332747371 | 1.17E-155 | 7.60E-152 |  |  |  |  |
| *SYNM* | -2.301444906 | 6.18E-32 | 1.24E-29 |  |  |  |  |
| *TGM5* | 4.139137932 | 1.35E-61 | 6.37E-59 |  |  |  |  |
| *CAPNS2* | 4.356947654 | 2.06E-87 | 2.06E-84 |  |  |  |  |
| *MYH11* | -2.176681715 | 1.42E-26 | 2.30E-24 |  |  |  |  |
| *PPL* | 2.304082371 | 1.70E-60 | 7.64E-58 |  |  |  |  |
| *PRSS27* | 3.951608403 | 9.32E-121 | 1.87E-117 |  |  |  |  |
| *SCNN1B* | 2.485280837 | 3.69E-37 | 8.59E-35 |  |  |  |  |
| *FOXN1* | 2.224101696 | 3.24E-27 | 5.28E-25 |  |  |  |  |
| *GAST* | -2.532414196 | 5.12E-22 | 6.38E-20 |  |  |  |  |
| *KRT13* | 7.091887641 | 7.64E-153 | 3.98E-149 |  |  |  |  |
| *KRT14* | 5.901257287 | 2.80E-116 | 5.21E-113 |  |  |  |  |
| *KRT15* | 2.741822877 | 3.85E-53 | 1.41E-50 |  |  |  |  |
| *KRT16P2* | 2.433702798 | 1.08E-21 | 1.32E-19 |  |  |  |  |
| *KRT16P3* | 2.309781908 | 6.84E-14 | 3.77E-12 |  |  |  |  |
| *KRT16* | 3.662468613 | 3.31E-63 | 1.60E-60 |  |  |  |  |
| *KRT24* | 4.009067645 | 1.55E-42 | 4.39E-40 |  |  |  |  |
| *KRTAP3-2* | 2.02721332 | 1.11E-10 | 3.50E-09 |  |  |  |  |
| *OTOP2* | 2.439117666 | 1.80E-26 | 2.90E-24 |  |  |  |  |
| *RNF222* | 3.38982504 | 9.36E-64 | 4.82E-61 |  |  |  |  |
| *ZNF750* | 3.702174919 | 9.10E-73 | 6.08E-70 |  |  |  |  |
| *C18orf26* | 3.66413892 | 9.18E-32 | 1.82E-29 |  |  |  |  |
| *DSC3* | 3.816231903 | 1.12E-71 | 6.80E-69 |  |  |  |  |
| *DSG1* | 2.65646614 | 3.40E-37 | 7.98E-35 |  |  |  |  |
| *DSG3* | 2.709881606 | 1.05E-29 | 1.91E-27 |  |  |  |  |
| *SERPINB11* | 3.189836905 | 2.19E-23 | 2.98E-21 |  |  |  |  |
| *SERPINB12* | 2.003887599 | 1.51E-11 | 5.65E-10 |  |  |  |  |
| *SERPINB13* | 4.85330142 | 5.39E-58 | 2.26E-55 |  |  |  |  |
| *SERPINB2* | 2.567338786 | 2.21E-29 | 4.00E-27 |  |  |  |  |
| *SERPINB3* | 3.130570362 | 2.50E-31 | 4.83E-29 |  |  |  |  |
| *CNFN* | 3.770121275 | 9.73E-96 | 1.10E-92 |  |  |  |  |
| *CNN1* | -2.944268291 | 1.61E-49 | 5.58E-47 |  |  |  |  |
| *CYP4F22* | 2.221114544 | 1.95E-42 | 5.39E-40 |  |  |  |  |
| *HSPB6* | -2.537312709 | 1.99E-37 | 4.72E-35 |  |  |  |  |
| *IGFL1* | 2.596345185 | 1.07E-24 | 1.53E-22 |  |  |  |  |
| *KLK12* | 2.551580731 | 1.50E-27 | 2.53E-25 |  |  |  |  |
| *KLK13* | 3.961249959 | 1.32E-68 | 7.31E-66 |  |  |  |  |
| *KLK5* | 4.099662655 | 3.17E-47 | 1.04E-44 |  |  |  |  |
| *KRTDAP* | 4.020014369 | 1.05E-54 | 4.02E-52 |  |  |  |  |
| *LGALS7B* | 2.637362032 | 4.08E-29 | 7.33E-27 |  |  |  |  |
| *LGALS7* | 2.570186197 | 5.61E-19 | 5.70E-17 |  |  |  |  |
| *LOC646862* | 2.895053765 | 1.32E-37 | 3.15E-35 |  |  |  |  |
| *LYPD3* | 3.441890298 | 1.72E-82 | 1.54E-79 |  |  |  |  |
| *NCCRP1* | 4.021449924 | 5.06E-78 | 4.25E-75 |  |  |  |  |
| *PSG4* | 2.202835343 | 6.05E-17 | 5.18E-15 |  |  |  |  |
| *SBSN* | 5.75306933 | 2.07E-114 | 3.37E-111 |  |  |  |  |
| *VSIG10L* | 2.327648885 | 2.16E-56 | 8.65E-54 |  |  |  |  |
| *ZNF812* | 2.680733497 | 5.18E-37 | 1.18E-34 |  |  |  |  |
| *ACTG2* | -3.328596143 | 1.70E-60 | 7.64E-58 |  |  |  |  |
| *CAPN14* | 4.932365314 | 1.25E-126 | 2.97E-123 |  |  |  |  |
| *DES* | -2.648743334 | 5.22E-31 | 9.86E-29 |  |  |  |  |
| *IL36A* | 5.176476772 | 2.27E-57 | 9.23E-55 |  |  |  |  |
| *IL36G* | 4.242379913 | 4.89E-73 | 3.44E-70 |  |  |  |  |
| *IL36RN* | 3.573118556 | 4.34E-38 | 1.08E-35 |  |  |  |  |
| *MAL* | 4.013076191 | 1.12E-79 | 9.68E-77 |  |  |  |  |
| *SMYD1* | -4.399482167 | 3.31E-60 | 1.46E-57 |  |  |  |  |
| *FAM83C* | 3.217521565 | 3.88E-35 | 8.14E-33 |  |  |  |  |
| *TGM3* | 3.679698501 | 5.62E-73 | 3.85E-70 |  |  |  |  |
| *WFDC12* | 2.068000292 | 8.89E-21 | 1.03E-18 |  |  |  |  |
| *WFDC5* | 3.268610117 | 1.27E-39 | 3.25E-37 |  |  |  |  |
| *CSTA* | 3.517736151 | 5.50E-89 | 5.97E-86 |  |  |  |  |
| *GPR87* | 2.404970582 | 5.77E-22 | 7.09E-20 |  |  |  |  |
| *IL20RB* | 2.1184825 | 7.04E-44 | 2.18E-41 |  |  |  |  |
| *TMEM40* | 3.996089318 | 1.68E-72 | 1.09E-69 |  |  |  |  |
| *TP63* | 3.348918651 | 8.74E-72 | 5.42E-69 |  |  |  |  |
| *WNT7A* | 2.520018714 | 1.67E-24 | 2.36E-22 |  |  |  |  |
| *ADH7* | 3.726570661 | 5.52E-36 | 1.20E-33 |  |  |  |  |
| *BMP3* | -2.114446715 | 2.19E-19 | 2.31E-17 |  |  |  |  |
| *CWH43* | 2.751062328 | 1.66E-18 | 1.58E-16 |  |  |  |  |
| *EPGN* | 3.429863837 | 9.82E-41 | 2.56E-38 |  |  |  |  |
| *FTLP10* | 2.293656051 | 1.96E-10 | 5.79E-09 |  |  |  |  |
| *HAND2* | -2.793004838 | 1.56E-36 | 3.49E-34 |  |  |  |  |
| *NBLA00301* | -3.416007199 | 8.15E-52 | 2.95E-49 |  |  |  |  |
| *NKX3-2* | -3.018368683 | 7.43E-61 | 3.46E-58 |  |  |  |  |
| *NKX6-1* | 2.892121114 | 5.65E-43 | 1.65E-40 |  |  |  |  |
| *SYNPO2* | -2.102415833 | 1.06E-27 | 1.82E-25 |  |  |  |  |
| *TMPRSS11A* | 6.726858013 | 5.93E-111 | 8.12E-108 |  |  |  |  |
| *TMPRSS11BNL* | 2.684848238 | 6.87E-15 | 4.50E-13 |  |  |  |  |
| *TMPRSS11B* | 5.925293999 | 1.33E-77 | 1.09E-74 |  |  |  |  |
| *TMPRSS11D* | 6.810385635 | 1.44E-156 | 1.25E-152 |  |  |  |  |
| *TMPRSS11E* | 6.022916484 | 6.91E-115 | 1.20E-111 |  |  |  |  |
| *TMPRSS11F* | 4.707268106 | 1.20E-53 | 4.48E-51 |  |  |  |  |
| *BC034636* | 2.454105564 | 7.99E-29 | 1.42E-26 |  |  |  |  |
| *CARTPT* | -3.09385634 | 8.70E-26 | 1.32E-23 |  |  |  |  |
| *FAT2* | 4.370003392 | 2.65E-132 | 7.68E-129 |  |  |  |  |
| *IRX4* | 2.373215168 | 8.64E-17 | 7.28E-15 |  |  |  |  |
| *SPINK5* | 3.873075968 | 2.53E-88 | 2.64E-85 |  |  |  |  |
| *SPINK6* | 2.391826391 | 8.08E-22 | 9.88E-20 |  |  |  |  |
| *SPINK7* | 3.902698851 | 4.13E-50 | 1.47E-47 |  |  |  |  |
| *CRISP3* | 2.001431819 | 1.59E-13 | 8.22E-12 |  |  |  |  |
| *HCG22* | 2.311063394 | 6.66E-30 | 1.22E-27 |  |  |  |  |
| *MLN* | -2.014069338 | 1.49E-11 | 5.57E-10 |  |  |  |  |
| *MUC21* | 6.911097061 | 3.73E-137 | 1.62E-133 |  |  |  |  |
| *MUC22* | 3.628822302 | 1.41E-41 | 3.79E-39 |  |  |  |  |
| *RAET1E* | 2.738400727 | 1.34E-70 | 7.94E-68 |  |  |  |  |
| *VIP* | -2.587717343 | 1.34E-35 | 2.87E-33 |  |  |  |  |
| *DPP6* | -2.373550245 | 2.96E-27 | 4.86E-25 |  |  |  |  |
| *FLNC* | -2.363614039 | 4.84E-38 | 1.17E-35 |  |  |  |  |
| *TAC1* | -2.138202675 | 2.50E-15 | 1.74E-13 |  |  |  |  |
| *GSDMC* | 2.234128497 | 1.55E-42 | 4.39E-40 |  |  |  |  |
| *LY6D* | 2.677239929 | 8.62E-26 | 1.31E-23 |  |  |  |  |
| *NKX6-3* | -2.247694767 | 3.78E-19 | 3.91E-17 |  |  |  |  |
| *SLURP1* | 4.035640781 | 3.37E-48 | 1.12E-45 |  |  |  |  |
| *AK128153* | 2.337007763 | 4.94E-43 | 1.46E-40 |  |  |  |  |
| *ANXA1* | 2.192006691 | 5.01E-55 | 1.98E-52 |  |  |  |  |
| *C9orf169* | 2.496487438 | 1.05E-45 | 3.32E-43 |  |  |  |  |
| *FOXE1* | 3.40082925 | 3.87E-35 | 8.14E-33 |  |  |  |  |
| *MORN5* | -2.414199444 | 3.99E-23 | 5.32E-21 |  |  |  |  |
| *AGTR2* | -2.230354056 | 1.87E-13 | 9.52E-12 |  |  |  |  |
| *ARSF* | 2.410497156 | 4.82E-26 | 7.42E-24 |  |  |  |  |
| *DCX* | -2.183939653 | 3.71E-26 | 5.79E-24 |  |  |  |  |
| *SMPX* | -2.26163717 | 5.41E-20 | 5.90E-18 |  |  |  |  |
